# Synthesis and Evaluation of Antiplasmodial Activity of 2,2,2-Trifluoroethoxychalcones and 2-Fluoroethoxy Chalcones against *Plasmodium falciparum* in Culture

**DOI:** 10.3390/molecules23051174

**Published:** 2018-05-14

**Authors:** Kavita Devi, Vinoth Rajendran, Ayushee, T. M. Rangarajan, Rishi Pal Singh, Prahlad C. Ghosh, Manjula Singh

**Affiliations:** 1 Fluoroorganic Laboratory, Center for Fire, Explosive and Environment Safety, Delhi-110 054, India; kavik188@gmail.com; 2 Department of Biochemistry, University of Delhi South Campus, Benito Juarez Road, New Delhi 110 021, India; vinoth.avj@gmail.com (V.R.); pcghose@gmail.com (P.C.G.); 3 Department of Chemistry, Hansraj College, University of Delhi, Delhi 110 007, India; ayusheesingh27@yahoo.in; 4 Department of Chemistry, Sri Venkateswara College, University of Delhi, New Delhi 110 021, India; 5 Department of Chemistry, Shivaji College, University of Delhi, New Delhi 110 027, India; manjulasingh56@gmail.com

**Keywords:** fluoroethoxychalcones, antiplasmodial activity, *Plasmodium falciparum* (3D7), artemisinin combination therapy, cytotoxicity

## Abstract

A new class of compounds comprising two series of chalcones with 2,2,2-trifluoroethoxy group and 2-fluoroethoxy groups were synthesized and screened for in vitro antiplasmodial activity against *Plasmodium falciparum* (3D7) using the [^3^H] hypoxanthine incorporation inhibition assay. Chalcones with 2,2,2-trifluoroethoxy groups substituted on the *p*- and *m*-positions of the 1-phenyl ring showed weak antiplasmodial activity, while compounds substituted on the *o*-position of the 1-phenyl ring displayed enhanced antiplasmodial activity, thus indicating that 2,2,2-trifluoroethoxy groups on the 1-phenyl ring of chalcones show position-dependent antiplasmodial activity. Of the 34 compounds synthesized, chalcones **3a** and **3f** exhibited significant inhibitory effects, with IC_50_ values of 3.0 μg/mL and 2.2 μg/mL, respectively. Moreover, these compounds **3a** and **3f** showed profound antiplasmodial activity in combination with artemisinin in vitro. The most active molecules, **3a**, and **3f**, were further assessed for their cytotoxicity towards mammalian Vero cells and the selectivity index (SI) values are 8.6, and 8.2 respectively, being considered non-toxic. We also studied the antiplasmodial activity of 2-fluoroethoxychalcones to discern the effect of the number of fluorine atoms in the fluoroethoxy group. Our results showed that chalcones with 2-fluoroethoxy group on the 1-phenyl ring exhibited more enhanced inhibitory effects on the growth of parasites than their trifluoro analogues, which reveals that monofluoroethoxy group is generally more effective than trifluoroethoxy group in the inhibition of parasite growth. Thus *o*-2,2,2-trifluoroethoxychalcones (Series 3) and 2-fluoroethoxychalcones may serve as good antiplasmodial candidates for future further development.

## 1. Introduction

Chalcones (1,3-diaryl-2-propen-1-ones), or α,β-unsaturated carbonyl compounds, the bioprecursors of flavonoids, are an interesting class of compounds that engross medicinal [1,2], synthetic [3], and material chemists [4,5,6] with their diverse applications. Considerable evidence has shown that chalcones have immense chemotherapeutic importance, such as anti-inflammatory [7,8], antimicrobial [9,10], antileishmanial [11], anticancer [12,13], antimalarial [14,15,16] activities. Moreover, radiolabeled chalcones have been used as PET imaging agents for in vivo imaging of the β-amyloid plaques of Alzheimer’s disease [17,18]. After the advent of licochalcone A with its antiplasmodial activity [19], a large number ofnovel synthetic chalcone derivatives and chalcone hybrids have been designed, synthesized and evaluated for their antiplasmodial activity [20,21,22,23,24]. Several reports suggest that chalcones’ antiplasmodial potential is due the ability to inhibit both the plasmodial aspartate protease and cysteine protease targets [24,25].

Malaria is a parasitic disease caused by *Plasmodium*, which is transmitted to human by female anopheline mosquitoes and continues to be a major cause of the death, killing an estimated 1.5 to 2.7 million people per year [14,26,27]. The high mortality rate caused by *P. falciparum* strains is compounded by the fact the parasite has shown clinical resistance to currently used first-line antimalarial drugs like chloroquine and vaccines have proven ineffective. Even though Artemisinin is currently considered to be an effective drug for treating chloroquine-resistant *P. falciparum* infections the WHO has recommended artemisinin-based combination therapies (ACTs) due to the slow development of clinical resistance to this compound. This averts or slows down the resistance developed by *P. falciparum* to this compound. ACTs also mitigate the increasing demand for artemisinin, which is isolated from the plant *Artemisia annua*, due to the unavailability of commercially viable synthesis. Therefore, there is a state of emergency to develop new antimalarial agents or drug combinations which are effective and cost-effective [15,16,28]. 

The use of fluorine or fluorine-containing functional groups in organic compounds has shown spectacular growth in all branches of chemistry, such as medicinal chemistry [29,30,31], agrochemistry [32], material sciences [33], etc. Substitution of fluorine or fluorine-containing functional group in organic molecules exerts large electronic effects on neighboring carbon centers and greatly affects the physico-chemical properties of the resulting molecules. Lipophilicity is an important criterion for the molecules that increases fat solubility and bioavailability, and can be increased by a strategic transformation of organic molecules into fluoroorganic molecules [29,30]. Fluorine substitution at an appropriate site of the molecule can increase their biological half-life, extended activity, and potency by altering the rate of drug metabolism [30,34]. Interestingly, 2,2,2-trifluoroethoxy group-containing molecules appear to be one of the most attractive fluoro functionalities in pharmaceuticals, found in blockbuster drugs such as flecainide, lansoprazole, MF-310, etc. [35,36].

Fluoroorganic compounds and chalcones alone exhibit broad ranges of applications [1,2,3,4,5,6,29,30,31,32,33,37]. Therefore, incorporation of fluorine or fluorine containing groups into chalcones would be expected to have a strong effect on their physico-chemical properties which, in turn, could augment their biological activities. Moreover, biological evaluations of fluorochalcones have not been fully explored, except for some fluorochalcone derivatives, which have been reported for their anti-inflammatory [38,39], or anti-proliferative activity [40], or as inhibitors of monoamine oxidase-B [41,42]. Recently, we have reported a novel methodology for the coupling of bromochalcones with alcohols and fluoroalcohols by Pd/*t*BuXPhos catalyst systems [43,44,45,46,47]. Taking into consideration of the importance of fluoroethoxy groups and chalcones in biological activity, we pleased to synthesize fluoroethoxy chalcones and assess their antiplasmodial activity. Herein, we report the synthesis and antiplasmodial evaluation of 2,2,2-trifluoroethoxychalcones and 2-fluoroethoxychalcones on the growth of *P. falciparum* in culture. The most active compounds, **3a** and **3f**, were further tested for hemolytic activity, cytotoxicity on Vero cells and in combination with artemisinin as a partner antimalarial agent.

## 2. Results and Discussion

### 2.1. Chemistry

**Scheme 1:**
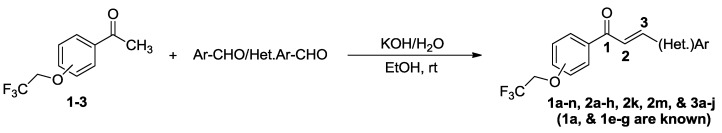
Synthesis of 2,2,2-trifluoroethoxychalcones.

A series of 2,2,2-trifluoroethoxychalcones **1a**–**n**, **2a**–**h**, **2k**, **2m**, and **3a**–**j** were synthesized in poor to excellent yields (40–94%) by base catalyzed Claisen−Schmidt condensation of 1-(4-(2,2,2-trifluoroethoxy)phenyl)ethanone (**1**), 1-(3-(2,2,2-trifluoroethoxy)phenyl)ethanone (**2**), and 1-(2-(2,2,2-trifluoroethoxy)phenyl)ethanone (**3**), with substituted benzaldehydes as shown in Scheme 1. All the reactions were carried out at room temperature for 30 min, except in the case of indole-2-carboxaldehyde which was the only aldehyde that required refluxing for 2 h as the reaction did not proceed at room temperature and gave the product **1n** in poor yield (40%).

**Scheme 2:**
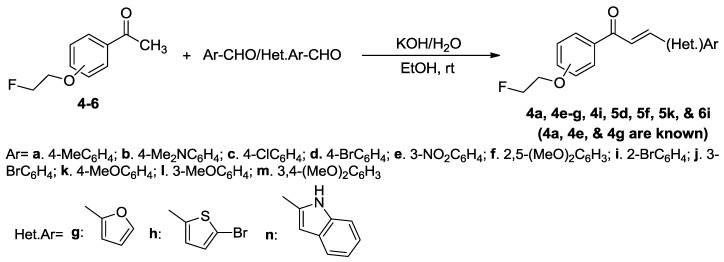

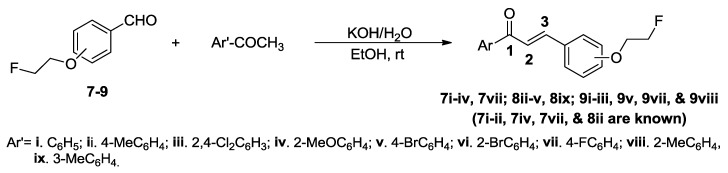
Synthesis of 2-fluoroethoxychalcones.

Similarly, 2-fluoroethoxychalcones, in which 2-fluoroethoxy group is substituted on both the 1-phenyl and 3-phenyl rings, were also synthesized in moderate to excellent yields (66–90%) by the same method from the corresponding 1-(4-(2-fluoroethoxy)phenyl)ethanone (**4**), 1-(3-(2-fluoroethoxy)phenyl)ethanone (**5**), and 1-(2-(2-trifluoroethoxy)phenyl)ethanone (**6**), with substituted benzaldehydes and 4-(2-fluoroethoxy)benzaldehyde (**7**), 3-(2-fluoroethoxy)benzaldehyde (**8**), and 2-(2-fluoroethoxy)benzaldehyde (**9**) with substituted acetophenones **i**–**ix** as shown in Scheme 2. Of the 59 chalcones synthesized, 12 chalcones (compounds **1a**, **1e**–**g**, **4a**, **4e**, **4g**, **7i**–**ii**, **7iv**, **7vii**, and **8ii**) have already been reported [43,44] and the rest of the chalcones reported here are novel. All the chalcones were characterized by ^1^H-, ^13^C- and ^19^F-NMR (NMR spectra of all the compounds can be found in the supplementary materials), IR and HRMS analysis and the data were consistent with the structure of the chalcones. The starting materials viz., ((2,2,2-trifluoroethoxy)phenyl)ethanones, ((2-fluoroethoxy)phenyl)ethanones, and 2-fluoroethoxy- benzaldehydes were synthesized by nucleophilic displacement reactions of 2,2,2-trifluoroethyl-4-methylbenzenesulphonate and 2-fluoroethyl-4-methylbenzenesulphonate with the corresponding hydroxyacetophenones and hydroxybenzaldehydes using sodium hydride base in dry DMF at 120 °C. The 2,2,2-trifluoroethyl-4-methylbenzenesulphonate and 2-fluoroethyl-4-methylbenzene- sulphonate were synthesized from the reaction of the corresponding fluoroalcohols with *p*-toluene-sulfonyl chloride in the presence of potassium hydroxide solution in water at room temperature.

### 2.2. Antiplasmodial Activity of 2,2,2-Trifluoroethoxychalcones

Initially, we intended to investigate the effect of the position of the 2,2,2-trifluoroethoxy group on the 1-phenyl ring of chalcones (Scheme 1) on the growth inhibition of *P. falciparum* (3D7) in culture. The 50% inhibitory concentration (IC_50_) values on parasite growth for all compound series are given in Table 1. The standard antimalarial drugs chloroquine (CQ) and artemisinin (ART) were used as positive controls. In the first series of fourteen chalcone derivatives **1a**–**n**, in which the 2,2,2-trifluoroethoxy group has been incorporated at the *p*-position of the 1-phenyl ring of chalcones (Scheme 1 and Table 1), showed very weak inhibitory effects on parasite growth, having IC_50_ values in the range of 32 to >50 µg/mL, except for the chalcone **1m** which showed good activity with an IC_50_ value of 10 µg/mL (27.3 µM). In the second series of ten chalcone derivatives **2a**–**h**, **2k**, and **2m**, in which 2,2,2-trifluoroethoxy group has been incorporated at the *m*-position of the 1-phenyl ring of chalcones (Scheme 1 and Table 1), six chalcones **2a**–**c**, **2h**, **2k**, and **2m** showed weak inhibitory effects with IC_50_ values of >46 µg/mL on parasite growth and other three chalcones **2d**–**f** showed moderate activity towards the parasite with IC_50_ values of 23–11 µg/mL. However, the chalcone **2g**, was found to be active against *P. falciparum* (3D7) parasite with an IC_50_ value of 6 μg/mL (20 µM). In the third series of ten chalcone derivatives **3a**–**j**, in which the 2,2,2-trifluoroethoxy group has been incorporated at the *o*-position of the 1-phenyl ring (Scheme 1 and Table 1), only two chalcones, **3e** and **3h**, showed weak activity and the other chalcones were found to display moderate (compounds **3b**, **3g**, and **3j**) and good activities (compounds **3a**, **3c**, **3d**, **3f**, and **3i**), with IC_50_ values less than 20 µg/mL and 10 µg/mL, respectively. 

**Figure 1:**
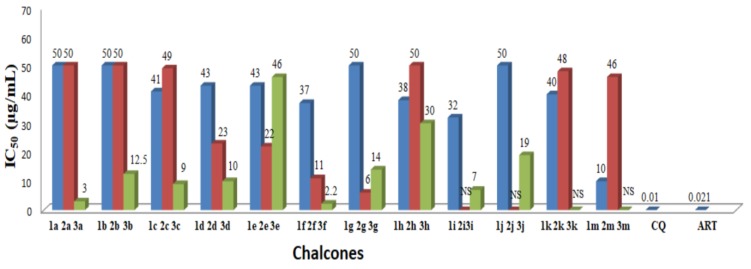
Results of in vitro antiplasmodial activity of some *para*-, *meta*-, and *ortho*-2,2,2-trifluoro ethoxychalcones with common substituent; NS: not synthesized.

Among the third series of chalcones, the chalcones **3a**–**d**, **3f**, and **3i**–**j** possessing diverse substituent(s) such as 4-methyl-, 4-dimethylamino-, 4-chloro-, 4-bromo-, 2,5-dimethoxy-, 2-bromo-, and 3-bromo- on the 3-phenyl ring and a 2,2,2-trifluoroethoxy group on the *o*-position of the 1-phenyl ring showed moderate to good antiplasmodial activities, whereas, the chalcones possessing the same substituents on the 3-phenyl ring, and a 2,2,2-trifluoroethoxy group on the *p*-(the first series) and *m*-positions (the second series) of the 1-phenyl ring (compounds **1a**–**d**, **1f**, **1i**–**j**; **2a**–**d**, and **2f**) showed weak antiplasmodial activities. Evidently, the chalcones with 2,2,2-trifluoroethoxy groups substituted on the *o*-position of the 1-phenyl ring displayed enhanced antiplasmodial activity (compounds **3a**–**d**, **3f**, and **3i**–**j**). Also, chalcone **2g**, with 3-furyl ring and a 2,2,2-trifluoroethoxy group on the *m*-position of the 1-phenyl ring showed significant activity in comparison with those of 2,2,2-trifluoroethoxy group on the *p*-(compound **1g**) and *o*-(compound **3g**) positions. This result indicates that the position of 2,2,2-trifluoroethoxy group on the 1-phenyl ring of chalcones plays an important role in the increase of parasite killing i.e., a 2,2,2-trifluoroethoxy group on the *o*-position in the 1-phenyl ring of chalcones exhibited an enhanced killing effect on parasites whereas, those on the *m*- and *p*-positions have little-to-no influence on parasite killing. Therefore, the antiplasmodial activity of chalcones with 2,2,2-trifluoroethoxy groups substituted on the 1-phenyl ring is highly position-dependent, which is graphically represented in Figure 1.

**Figure 2:**
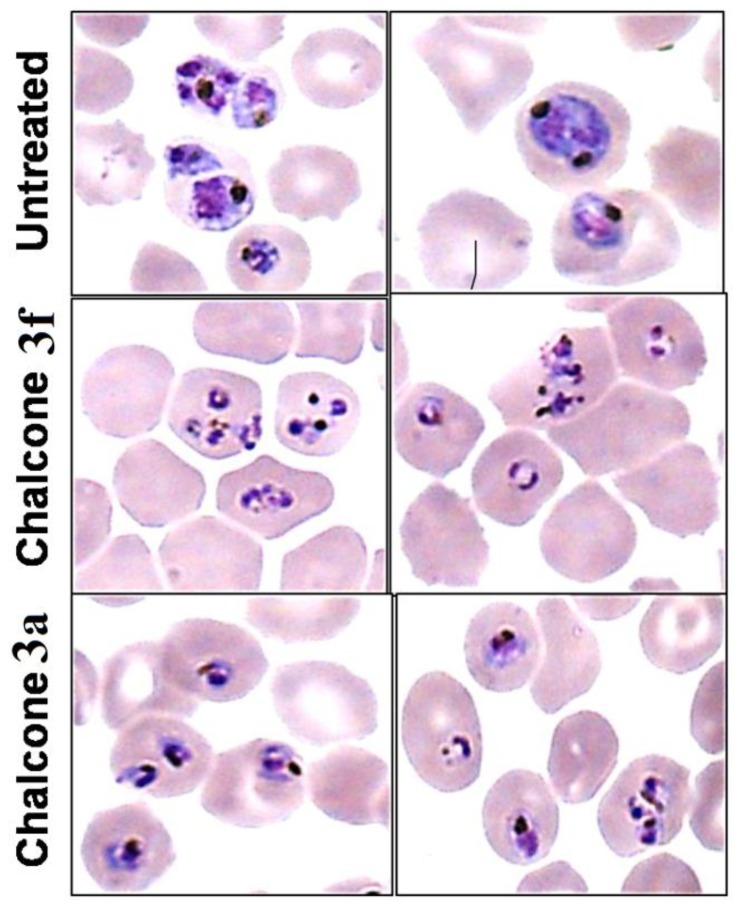
Effect of 2,2,2-trifluoroethoxychalcones (**3a** and **3f**) upon treatment causes developmental growth arrest of *P. falciparum* (3D7) on blood stages shown using Giemsa-stained light micrographs of highly synchronized ring-stage parasites in comparison to untreated parasites.

We further evaluated the stage-specific inhibitory effect of the most active chalcones **3a** and **3f** on the progression of developmental blood stages and survival of parasites. Parasites treated with chalcones displayed altered phenotypes with distorted morphological appearance according to microscopy studies as shown in Figure 2. Moreover, compounds **3a** and **3f** arrested the growth of mature blood stages (trophozoite) of parasites, leading to cell death. The most active compounds, **3a** and **3f** were found to be non-hemolytic at their effective concentrations (<20 µg/mL) indicating the non-toxicity towards the normal (uninfected) red blood cells.

Most importantly, identification ofnew synthetic derivatives as drug partners for standard combination therapy with artemisinin is now recommended for the treatment of *P. falciparum* malaria due to improved efficacy, fast action, and delaying the resistance development [15,16]. Therefore, the effect of the most active molecules **3a** and **3f** in combination with artemisinin on the various blood stages of parasites in culture were studied.

It is observed that chalcones **3a** and **3f** exhibited enhanced antiplasmodial activity with reduced IC_50_ values, as shown in Table 2. The drug combination study was carried out at a fixed concentration of artemisinin (5 ng/mL & 10 ng/mL) with varying concentrations of the chalcones **3a** and **3f** to find out the 50% inhibition concentration of parasite growth.

It is important to note that the IC_50_ values of chalcones **3a** and **3f** alone were found to be 3.0 µg/mL and 2.2 µg/mL (Table 1) respectively, and the parasite killing effect of artemisinin at 5 ng/mL and 10 ng/mL concentration was found to be 10–15% only. However, the combination study of chalcones **3a** and **3f** at a fixed concentration of artemisinin showed enhanced antiplasmodial activity with reduced IC_50_ values (Table 2). Therefore, the combined treatment of artemisinin with chalcones **3a** and **3f** exhibited positive interactions with synergetic effect on the parasites. 

As a result, these active molecules may find an effective means as strong chemotherapeutic agent in combination with other antimalarial drugs in combating clinical resistance against malaria infections. 

### 2.3. Antiplasmodial Activity of 2-fluoroethoxychalcones

Due to fluorine’s baffling nature, the effects of fluorine substitution on chemical reactivities and biological activities remains unpredictable [30,48]. Therefore, we attempted to explore the effect of the number of fluorine atoms in the fluoroethoxy group and its position in chalcones on antiplasmodial activity. Therefore, we synthesized a series of 2-fluoroethoxychalcone derivatives in which a 2-fluoroethoxy group substituted in both the 1-phenyl ring and 3-phenyl ring of chalcones (Scheme 2). The antiplasmodial activity results of these 2-fluoroethoxychalcones are given in Table 3. All the chalcones with 2-fluoroethoxy groups on the 1-phenyl ring (compounds **4a**, **4e**–**g**, **5d**, **5k**, and **6i**) exhibited moderate to good antiplasmodial activity, with IC_50_ values ranging from 20 µg/mL to 6.5 µg/mL.

**Figure 3:**
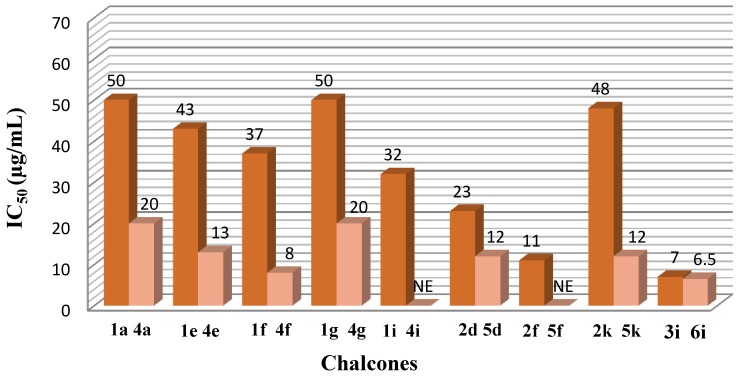
Graphical representation for the comparison of antiplasmodial activity of some 2,2,2-trifluoroethoxychalcones and 2-fluoroethoxychalcones with some common substituents; NE: not evaluated.

Surprisingly, it is observed that 2-fluoroethoxychalcones **4a**, **4e**–**g**, **5d**, **5k**, and **6i** showed enhanced parasite-killing activity, relative to their 2,2,2-trifluroethoxy analogues, irrespective of the position of the 2-fluoroethoxy group on the 1-phenyl ring (Figure 3).

As a result, the number of fluorine atoms on the ethoxy group in chalcones plays an important role in their antiplasmodial activity. The chalcones, **4f,** and **6i** showed good antiplasmodial activity with IC_50_ values of 8.0 µg/mL and 6.5 µg/mL, respectively. Similarly, chalcones **7i**–**iv**, **7vii**; **8i**–**v**; **9i**–**iii**, **9v**, and **9vii**–**viii** with 2-fluoroethoxy groups on the 3-phenyl ring showed moderate to good antiplasmodial activity. Among these chalcones, the chalcones **7iv**, **8iii**, **9ii**, **9iii**, and **9viii** showed significant antiplasmodial activities, with IC_50_ values of 3.5–6.0 µg/mL. Of all 2-fluoro-ethoxychalcones, only two, **8iii**, and **9ii**, were found to be most active towards *P. falciparum* parasite. Our result reveals that 2-fluoroethoxy substitution in chalcone derivatives are more effective than 2,2,2-trifluoroethoxy substitution, irrespective of the position, in inhibition of plasmodial growth.

Finally, the most active molecules **3a**, **3f**, **8iii**, and **9ii** were studied for their cytotoxic effect in Vero cell lines (kidney cells of African green monkey) and the results are given in Table 4. The selectivity index (SI) values for the most active molecules, **3a**, **3f**, **8iii**, and **9ii** were calculated using the formula of IC_50_ value fnormal cell line (Vero)/IC_50_ value *P. falciparum* (3D7), are 8.6, 8.2, 3.3 and 6.9, respectively. The most active molecules showed minimal cytotoxic effect towards Vero cells at therapeutic dosages with selective killing of parasites. Therefore, the active molecules are considered to be selective towards the inhibition of parasites.

## 3. Experimental 

### 3.1. General Information 

All substituted benzaldehydes, substituted acetophenones, *p*-toluenesulphonyl chloride and dry DMF were purchased from Spectrochem Pvt. Ltd. (Mumbai, India). 2′-Hydroxy-, 3′-hydroxy-, and 4′-hydroxyacetophenones, 2-hydroxy-, 3-hydroxy-, and 4-hydroxybenzaldehydes were purchased from Sigma Aldrich Chemicals pvt. Ltd. (Bangalore, India). Sodium hydride 60% dispersion in mineral oil was purchased from Thermo Fisher Scientific India Pvt. Ltd. (Mumbai, India) and purged withnitrogen after every use. Other common solvents & reagent were purchased from other common suppliers. Other starting materials such as 2,2,2-trifluoroethyl-4-methylbenzenesulphonate, 2-fluoroethyl-4-methylbenzenesulphonate, 4′-(2,2,2-trifluoroethoxy)-, 3′-(2,2,2-trifluoroethoxy)-, and 2′-(2,2,2-trifluoroethoxy)acetophenones, 4′-(2-fluoroethoxy)-, 3′-(2-fluoroethoxy)-, and 2′-(2-fluoroethoxy)acetophenones, 4-(2-fluoroethoxy)benzaldehyde, 3-(2-fluoroethoxy)benzaldehyde, and 2-(2-fluoroethoxy)benzaldehyde were synthesized in house and used for the preparation of chalcones. The chalcones were prepared by a Claisen-Schmidt condensation reported elsewhere in the literature. Reactions were monitored by thin layer chromatography (TLC) on pre-coated silica gel plates (silica gel 60, F_254,_ Merck, Darmstadt, Germany) and visualized under UV light.

### 3.2. Analytical Methods

NMR data were obtained on a Jeol 400 MHz spectrometers (Jeol USA, Inc., Peabody, MA, USA). All compounds were characterized by ^1^H-, ^13^C-, ^19^F-NMR (at 399.78 MHz, 100.5 MHz, 376.17 MHz respectively), IR and HRMS. All ^1^H- and ^13^C-NMR chemical shifts were reported in parts per million (ppm) and were measured relative to TMS or residual deuterated CDCl_3_ as solvent. ^19^F-NMR signals are reported in ppm units and were referenced to CFCl_3_ as external reference. FTIR spectra were recorded on a model RXI (Perkin-Elmer, Waltham, MA, USA) or model alphaT (Bruker, Billerica, MA, USA, untry) spectrometer, using KBr pellets and KBr films (DCM solvent) methods. HRMS (ESI) measurements were performed on an 1969A TOF (MS ES+) mass spectrometer (Agilent, Santa Clara, CA, USA) and an Orbitrap Elite Hybrid Ion Trap-Orbitrap Mass Spectrometer (Thermo Scientific, Waltham, MA, USA). Yields refer to isolated compounds.

### 3.3. Biological Evaluation

#### 3.3.1. In Vitro Culture of *Plasmodium falciparum*

We have tested a series of fluoroethoxychalcone derivatives against drug-susceptible *P. falciparum* (3D7) obtained from National Institute of Malaria Research (NIMR, New Delhi, India). The strain of *P. falciparum* was maintained by serial passages in human red blood cells (RBCs) cultured at 4–5% hematocrit in RPMI-1640 medium supplemented with 0.5% AlbuMAX II (complete RPMI) and incubated at 37 °C under the atmosphere of mixed gases (5% CO_2_, 5% O_2_, and 90% N_2_). The whole blood was collected from the Rotary Blood Bank (New Delhi, India). RBCs were separated under sterile conditions by centrifugation to remove plasma and peripheral blood mononuclear cells (PBCs) using Histopaque 1077 solution. The levels of parasitemia were routinely monitored by thin blood smear and staining with 5% Giemsa solution.

#### 3.3.2. Evaluation of In Vitro Antiplasmodial Activity

In vitro antiplasmodial activity was determined based on the [^3^H]-hypoxanthine incorporation inhibition assay. The level of radiolabelled precursor [^3^H]-hypoxanthine incorporation in the nucleic acid of parasites was used as direct measurement for determining the inhibitory growth concentration of parasites. Stock solutions of test compounds (chalcones) and artemisinin were dissolved in 100% DMSO and chloroquine diphosphate in sterile distilled water. The final concentration of DMSO is 0.4% (vehicle control) was found to be non-toxic to parasites. Briefly, different concentrations of test compounds ranging from 50 µg/mL to 0.1 µg/mL were serially diluted (2-fold dilution) and added to *P. falciparum* infected asynchronous erythrocyte suspension (4% final hematocrit and 2% parasitemia) in a 96-well micro dilution plate along with an untreated control. In another set, different concentration of standard antimalarial drugs chloroquine diphosphate (CQ) and artemisinin (ART) were added to infected erythrocyte suspension as positive control. After 30 h of incubation period at 37 °C, 20 µL of 0.2 µCi/well of [^3^H]-hypoxanthine (American Radiolabeled Chemicals, Inc., ST. Louis, MO, USA specific activity 25 Ci/mmol) was added to each well containing infected erythrocytes. After additional 18 h of incubation, the cells were harvested onto a glass-fiber filter mat (Whatman GF/A) using a semi-automated 96 well cell harvester (Skatron, Norway, MI, USA). The dried paper discs were placed in 5 mL toluene based scintillation cocktail and radioactive count was measured in a TriCarb 2900TR liquid scintillation analyzer beta-counter (PerkinElmer, Waltham, MA, USA). The 50% inhibitory concentration (IC_50_) values were determined by plotting the drug concentration versus the percent cell viability of the parasite after 48 h of incubation of growth assay period. All data points were collected in triplicate for each experiment conducted independently.

#### 3.3.3. Assessment of In Vitro Hemolytic Activity

The hemolytic activity was measured at different concentrations for highly potent 2,2,2-trifluoroethoxychalcones, **3a,** and **3f**, and 2-fluroethoxychalcones, **6i**, **7iv**, **8iii**, **9ii**, **9iii**, and **9viii**, in normal and infected erythrocytes by measuring the lysis of RBCs in culture medium at absorbance of 405 nm. Briefly, a series of concentrations 50 µg/mL−0.1 µg/mL were added to normal erythrocytes at 4% hematocrit in a 96-well plate for 48 h incubation period at 37 °C. After incubation, the plate was spun down and absorbance of supernatant was measured using spectrophotometric microplate reader (infinite 200 pro, Tecan trading AG, Switzerland). By mixing the erythrocytes with sterile distilled water (for 100% hemolysis) and PBS alone (for baseline values) served as positive and negative controls, respectively.

#### 3.3.4. Assessment of In Vitro Cytotoxicity on Vero Cells

The cytotoxic effect of most potent molecules (**3a**, **3f**, **8iii**, and **9ii**) was evaluated against Vero cells using MTT assay. In Brief, exponentially growing Vero cells were seeded in 96 well plates at a cell density of 1 × 10^4^ cells/well in 200 μL DMEM medium, 24 h prior to the experiment. Cells were incubated with different concentrations of compounds (100 µg/mL to 0.78 µg/mL) two-fold dilution and incubated further for 48 h at 37 °C, followed by incubating with 10 µL of 3-(4,5-dimethylthiazole-2-yl)-2,5-diphenyltetrazolium bromide) MTT reagent (5 mg/mL) for additional 3 h at 37 °C. The presence of formazan crystals were solubilized using 100 µL DMSO and the absorbance of each well optical density was measured using a microplate reader (BioRad, CA, USA) at 570 nm. The 50% inhibitory concentration (IC_50_ value) was determined by plotting the drug concentration versus the percentage cell viability of 48 h of a growth assay period. The relative cell viability (%) compared with control cells were calculated as follows:

(1) $$\mathrm{Cell}\;\mathrm{viability}\;\left(\%\right)=\frac{\mathrm{Abs}\left(\mathrm{sample}\right)-\left(\mathrm{Abs}\;\mathrm{blank}\right)}{\mathrm{Abs}\left(\mathrm{control}\right)-\left(\mathrm{Abs}\;\mathrm{blank}\right)}\times 100,$$

### 3.4. General Procedure for Fluoroethoxylation of Hydroxyacetophenones and Benzaldehydes

A round bottom flask was charged with sodium hydride (1.2 eq.). The appropriate hydroxyacetophenone or hydroxybenzaldehyde (1 eq.) in DMF (100 mL) was added dropwise over 10 min at rt. Then 2,2,2-trifluoroethyl-4-methylbenzenesulphonate (1.2 eq.) in DMF (50 mL) was added dropwise. The reaction mixture was then allowed to reflux at 130 °C for 15 h. After completion of reaction, monitored by TLC, the reaction mixture was poured into water and extracted with ethyl acetate. The extract was washed with water, brine solution and finally dried over sodium sulfate and evaporated to dryness. The crude reaction mixture was purified by column chromatography (silica gel 60–200 mesh) to afford desired compounds.

*1-(4-(2,2,2-Trifluoroethoxy)phenyl)ethanone* (**1**) [49]. White solid, Yield: 75%, m.p. 67–68 °C (lit. 67–68 °C); ^1^H-NMR (CDCl_3_) *δ* (ppm): 2.57 (s, 3H), 4.42 (q, 2H, ^3^*J* = 8.0 Hz), 6.98–7.00 (m, 2H), 7.97 (d, 2H, ^3^*J* = 8.8 Hz).

*1-(3-(2,2,2-Trifluoroethoxy)phenyl)ethanone* (**2**) [49]. White solid, Yield: 71%, m.p. 66–68 °C (lit. 67–68 °C); ^1^H-NMR (CDCl_3_) *δ* (ppm): 2.61 (s, 3H), 4.41 (q, 2H, ^3^*J* = 8.0 Hz), 7.17–7.19 (m, 1H), 7.41–7.45 (m, 1H), 7.51–7.52 (m, 1H), 7.63–7.65 (m, 1H)

*1-(2-(2,2,2-Trifluoroethoxy)phenyl)ethanone* (**3**) [50]. Yellow liquid, Yield: 68%, ^1^H-NMR (CDCl_3_) *δ* (ppm): 2.63 (s, 3H), 4.45 (q, 2H, ^3^*J* = 7.9 Hz), 6.88–6.90 (m, 1H), 7.10–7.14 (m, 1H), 7.47–7.52 (m, 1H), 7.79–7.81 (m, 1H)

*1-(4-(2-Fluoroethoxy)phenyl)ethanone* (**4**) [46]. Yellow solid, Yield: 75%, m.p. 52–54 °C (lit. 51–55 °C); ^1^H-NMR (CDCl_3_) *δ* (ppm): 2.56 (s, 3H), 4.28 (dt, 2H, ^3^*J*_H-H_ = 3.9 Hz, ^3^*J*_H-F_ = 27.5 Hz), 4.78 (dt, 2H, ^3^*J*_H-H_ = 4.2 Hz, ^2^*J*_H-F_ = 47.5 Hz), 6.95–6.97 (m, 2H), 7.93–7.95 (m, 2H)

*1-(3-(2-Fluoroethoxy)phenyl)ethanone* (**5**)**.** White solid, Yield: 69%, m.p. 66–68 °C; ^1^H-NMR (CDCl_3_) *δ* (ppm): 2.60 (s, 3H), 4.27 (dt, 2H, ^3^*J*_H-H_ = 3.90 Hz, ^3^*J*_H-F_ = 27.90 Hz), 4.78 (dt, 2H, ^3^*J*_H-H_ = 4.1 Hz, ^2^*J*_H-F_ = 47.9 Hz), 7.14–7.17 (m, 1H), 7.37–7.41 (m, 1H), 7.50 (s, 1H), 7.56–7.58 (m, 1H); ^13^C-NMR (CDCl_3_) *δ* (ppm): 26.86, 67.38 (d, ^2^*J*_C-F_ = 20.2 Hz), 81.91 (d, ^1^*J*_C-F_ = 171.1 Hz), 113.04, 120.41, 121.85, 129.86, 138.61, 158.76, 198.00; HRMS (ESI) calcd. for C_10_H_11_FO_2_: [M + H]^+^ 183.0821; Found: 183.0818.

*1-(2-(2-Fluoroethoxy)phenyl)ethanone)* (**6**)**.** Yellow liquid, Yield: 64%, ^1^H-NMR (CDCl_3_) *δ* (ppm): 2.66 (s, 3H), 4.32 (dt, 2H, ^3^*J*_H-H_ = 4.1 Hz, ^3^*J*_H-F_ = 27.8 Hz), 4.81 (dt, 2H, ^3^*J*_H-H_ = 4.7 Hz, ^2^*J*_H-F_ = 46.5 Hz), 6.92–6.94 (m, 1H), 7.02–7.06 (m, 1H), 7.44–7.48 (m, 1H), 7.75–7.78 (m, 1H); ^13^C-NMR (CDCl_3_) *δ* (ppm): 32.14, 67.73 (d, ^2^*J*_C-F_ = 20.1 Hz), 81.66 (d, ^1^*J*_C-F_ = 171.6 Hz), 112.49, 121.42, 128.73, 130.75, 133.79, 157.77, 199.94; HRMS(ESI) calcd. for C_10_H_11_FO_2_: [M + H]^+^ 183.08213 and [M + Na]^+^ 205.06408; Found: 183.0813 and 205.0634.

*4-(2-Fluoroethoxy)benzaldehyde* (**7**) [46]. Yellow liquid, Yield: 77%, ^1^H-NMR (CDCl_3_) *δ* (ppm): 4.30 (dt, 2H, ^3^*J*_H-H_ = 4.0 Hz, ^3^*J*_H-F_ = 28.3 Hz), 4.79 (dt, 2H, ^3^*J*_H-H_ = 3.9 Hz, ^2^*J*_H-F_ = 47.5 Hz), 7.02–7.05 (m, 2H), 7.84–7.86 (m, 2H), 9.90 (s, 1H)

*3-(2-Fluoroethoxy)benzaldehyde* (**8**)**.** Yellow oil, Yield: 71%, ^1^H-NMR (CDCl_3_) *δ* (ppm): 4.28 (dt, 2H, ^3^*J*_H-H_ = 4.0 Hz, ^3^*J*_H-F_ = 28 Hz), 4.78 (dt, 2H, ^3^*J*_H-H_ = 4.0 Hz, ^2^*J*_H-F_ = 47.5 Hz), 7.22–7.24 (m, 1H), 7.40–7.41 (m, 1H), 7.45–7.51 (m, 2H), 9.98 (s, 1H); ^13^C-NMR (CDCl_3_) *δ* (ppm): 67.43 (d, ^2^*J*_C-F_ = 20.7 Hz), 81.83 (d, ^1^*J*_C-F_ = 170.9 Hz), 112.60, 122.32, 124.34, 130.36, 137.92, 159.11, 192.14; ^19^F-NMR (CDCl_3_) *δ* (ppm): −223.44 to −223.84 (m, 1F).

*2-(2-Fluoroethoxy)benzaldehyde* (**9**) [51]. Yellow solid, Yield: 69%, m.p. 37–38 °C (lit. 37.5–38 °C); ^1^H-NMR (CDCl_3_) *δ* (ppm): 4.35 (dt, 2H, ^3^*J*_H-H_ = 3.9 Hz, ^3^*J*_H-F_ = 27.9 Hz), 4.82 (dt, 2H, ^3^*J*_H-H_ = 3.7 Hz, ^2^*J*_H-F_ = 47.8 Hz), 6.97–6.99 (m, 1H), 7.05–7.09 (m, 1H), 7.55 (t, 1H, ^3^*J* = 8.0 Hz), 7.86 (d, 1H, ^3^*J* = 7.8 Hz), 10.54 (s, 1H).

### 3.5. General Procedure for the Preparation of Fluoroethoxychalcones

An aqueous solution of KOH (1.2 eq.) was added at room temperature to a solution of the appropriate acetophenone (1.0 eq.), and benzaldehyde (1.2 eq.) in ethanol (10 mL). The reaction mixture was then stirred for 30 min. After completion of reaction, the reaction was judged by TLC. The reaction mixture was filtered through a Buckner funnel under vacuum. The solid was washed several times with a 1:1 ethanol-water mixture. The solid was finally dried under vacuum. The crude product was further purified by column chromatography or recrystallization.

*(E)-3-(4-Methylphenyl)-1-(4-(2,2,2-trifluoroethoxy)phenyl)prop-2-en-1-one* (**1a**) [43]. Yellow crystalline solid, Yield: 87%, m.p. 148–150 °C (lit. 148–151 °C); ^1^H-NMR (CDCl_3_) *δ* (ppm): 2.40 (s, 3H), 4.44 (q, 2H, ^3^*J* = 8.0 Hz), 7.03 (d, 2H, ^3^*J* = 8.7 Hz), 7.23 (d, 2H, ^3^*J* = 8.8 Hz) 7.48 (d, 1H, ^3^*J* = 15.2 Hz), 7.55 (d, 2H, ^3^*J* = 8.4 Hz), 7.80 (d, 1H, ^3^*J* = 15.6 Hz), 8.06 (d, 2H, ^3^*J* = 8.8 Hz).

*(E)-3-(4-(Dimethylamino)phenyl)-1-(4-(2,2,2-trifluoroethoxy)phenyl)prop-2-en-1-one* (**1b**)**.** Yellow solid, Yield: 77%, m.p. 130–135 °C; ^1^H-NMR (CDCl_3_) *δ* (ppm): 3.05 (s, 6H), 4.43 (q, 2H, ^3^*J* = 8.0 Hz), 6.70 (d, 2H, ^3^*J* = 8.4 Hz), 7.02 (d, 2H, ^3^*J* = 9.1 Hz), 7.33 (d, 1H, ^3^*J* = 15.2 Hz), 7.55 (d, 2H, ^3^*J* = 8.4 Hz), 7.79 (d, 1H, ^3^*J* = 15.2 Hz), 8.04 (d, 2H, ^3^*J* = 9.1 Hz); ^13^C-NMR (CDCl_3_) *δ* (ppm): 40.26, 65.65 (q, ^2^*J*_C-F_ = 36.2 Hz), 111.92, 114.53, 116.43, 122.71, 130.54, 130.75, 133.63, 145.75, 152.14, 160.36, 188.95; ^19^F-NMR (CDCl_3_) *δ* (ppm): −73.7(t, 3F,^3^*J* = 8.7 Hz); IR (KBr) *ν*: 3070, 2909, 1650, 1585, 1528, 1371, 1343, 1287, 1231, 1162, 1069, 1027, 969, 808, 676; HRMS (ESI) calcd. for C_19_H_18_F_3_NO_2_: [M + H]^+^ 350.1368 and [M + Na]^+^ 372.1187; Found: 350.1366 and 372.1163.

*(E)-3-(4-Chlorophenyl)-1-(4-(2,2,2-trifluoroethoxy)phenyl)prop-2-en-1-one* (**1c**)**.** Pale yellow solid, Yield: 90%, m.p. 139–140 °C; ^1^H-NMR (CDCl_3_) *δ* (ppm): 4.44 (q, 2H, ^3^*J* = 8.0 Hz), 7.04 (d, 2H, ^3^*J* = 8.8 Hz), 7.40 (d, 2H, ^3^*J* = 8.4 Hz), 7.49 (d, 1H, ^3^*J* = 15.4 Hz), 7.58 (d, 2H, ^3^*J* = 8.4 Hz), 7.76 (d, 1H, ^3^*J* = 15.4 Hz), 8.05 (d, 2H, ^3^*J* = 8.8 Hz); ^13^C-NMR (CDCl_3_) *δ* (ppm): 65.70 (q, ^2^*J*_C-F_ = 36.7 Hz), 114.79, 122.16, 129.41, 129.72, 131.06, 132.70, 133.54, 136.57, 143.23, 160.95, 188.50; ^19^F-NMR (CDCl_3_) *δ* (ppm): −73.67 (t, 3F, ^3^*J* = 8.6 Hz); IR (KBr) *ν*: 3066, 2947, 2815, 1663, 1609, 1510, 1490, 1406, 1343, 1293, 1162, 1029, 972, 811, 692, 657; HRMS (ESI) calc. for C_17_H_12_ClF_3_O_2_: [M + H]^+^ 341.0056; Found: 341.056.

*(E)-3-(4-Bromophenyl)-1-(4-(2,2,2-trifluoroethoxy)phenyl)prop-2-en-1-one* (**1d**)**.** Pale yellow solid, Yield: 77%, m.p. 145–146 °C; ^1^H-NMR (CDCl_3_) *δ* (ppm): 4.44 (q, 2H, ^3^*J* = 8.0 Hz), 7.04 (d, 2H, ^3^*J* = 9.1 Hz), 7.49–7.57 (m, 5H), 7.74 (m, 1H), 8.05 (d, 2H, ^3^*J* = 9.1 Hz); ^13^C-NMR (CDCl_3_) *δ* (ppm): 65.70 (q, ^2^*J*_C-F_ = 36.6 Hz), 114.79, 122.25, 124.93, 129.92, 131.06, 132.37, 132.68, 133.96, 143.30, 160.95, 188.49; ^19^F-NMR (CDCl_3_) *δ* (ppm): −73.67 (t, 3F, ^3^*J* = 8.7 Hz); IR (KBr) *ν*: 2946, 2812, 1668, 1609, 1511, 1487, 1349, 1305, 1293, 1254, 1180, 1161, 1079, 1028, 972, 808, 687; HRMS (ESI) calc. for C_17_H_12_BrF_3_O_2_: [M + H]^+^ 385.0051; Found: 385.0037.

*(E)-3-(3-Nitrophenyl)-1-(4-(2,2,2-trifluoroethoxy)phenyl)prop-2-en-1-one* (**1e**) [43]. Yellow solid, Yield: 94%, m.p. 169–171 °C (lit. 168–171 °C); ^1^H-NMR (CDCl_3_) *δ* (ppm): 4.46 (q, 2H,^3^*J* = 7.9 Hz), 7.07 (d, 2H, ^3^*J* = 9.1 Hz), 7.62 (d, 1H, ^3^*J* = 15.9 Hz), 7.84 (d, 1H, ^3^*J* = 15.9 Hz) 7.92 (d, 1H, ^3^*J* = 7.6 Hz), 8.09 (d, 2H, ^3^*J* = 8.5 Hz), 8.26 (d, 1H, ^3^*J* = 8.5 Hz), 8.52 (s, 1H).

*(E)-3-(2,5-Dimethoxyphenyl)-1-(4-(2,2,2-trifluoroethoxy)phenyl)prop-2-en-1-one* (**1f**) [43]. Yellow solid, Yield: 78%, m.p. 98–99 °C (lit. 97–99 °C); ^1^H-NMR (CDCl_3_) *δ* (ppm): 3.82 (s, 3H), 3.87 (s, 3H), 4.44 (q, 2H, ^3^*J* = 7.9 Hz), 6.87–6.89 (m, 1H), 6.93–6.96 (m, 1H), 7.03 (d, 2H, ^3^*J* = 8.8 Hz), 7.16–7.17 (m, 1H), 7.57 (d, 1H, ^3^*J* = 15.9 Hz), 8.04–8.09 (m, 3H).

*(E)-3-(Furan-2-yl)-1-(4-(2,2,2-trifluoroethoxy)phenyl)prop-2-en-1-one* (**1g**) [43]. Light yellow crystalline solid, Yield: 61%, m.p. 94–96 °C (lit. 94.5–96.5 °C); ^1^H-NMR (CDCl_3_) *δ* (ppm): 4.43 (q, 2H, ^3^*J* = 8.0 Hz), 6.52 (m, 1H), 6.72 (d, 1H, ^3^*J* = 3.5 Hz), 7.03 (d, 2H, ^3^*J* = 8.7 Hz), 7.45 (d, 1H, ^3^J = 15.3 Hz), 7.52 (s, 1H), 7.59 (d, 1H, ^3^*J* = 15.3 Hz), 8.07 (d, 2H, ^3^*J* = 8.7 Hz).

*(E)-3-(5-Bromothiophen-2-yl)-1-(4-(2,2,2-trifluoroethoxy)phenyl)prop-2-en-1-one* (**1h**)**.** Pale yellow solid, Yield: 93%, yield, m.p. 93–94 °C; ^1^H-NMR (CDCl_3_) *δ* (ppm): 4.44 (q, 2H, ^3^*J* = 8.0 Hz), 7.03 (d, 2H, ^3^*J* = 8.7 Hz), 7.06 (d, 1H, ^3^*J* = 4.2 Hz), 7.10 (d, 1H, ^3^*J* = 4.2 Hz), 7.21 (d, 1H, ^3^*J* = 15.6 Hz), 7.81 (d, 1H, ^3^*J* = 15.6 Hz), 8.02 (d, 2H, ^3^*J* = 8.7 Hz); ^13^C-NMR (CDCl_3_) *δ* (ppm): 65.71 (q, ^2^*J*_C-F_ = 36.0 Hz), 114.80, 116.48, 120.69, 130.96, 131.51, 132.49, 132.60, 136.20, 142.12, 160.96, 187.84; ^19^F-NMR (CDCl_3_) *δ* (ppm): −73.68 (t, 3F, ^3^*J* = 7.6 Hz); IR (KBr) *ν*: 3068, 2829, 1648, 1605, 1510, 1422, 1353, 1294, 1249, 1168, 1159, 1069, 1028, 972, 826, 796, 675 cm^−1^; HRMS (ESI) calcd. for C_15_H_10_BrF_3_O_2_S: [M + H]^+^ 390.9615; Found: 390.9608.

*(E)-3-(2-Bromophenyl)-1-(4-(2,2,2-trifluoroethoxy)phenyl)prop-2-en-1-one* (**1i**)**.** Pale yellow solid, Yield: 81%, m.p. 107–108 °C; ^1^H-NMR (CDCl_3_) *δ* (ppm): 4.43 (q, 2H, ^3^*J* = 7.9 Hz), 7.02 (d, 2H, ^3^*J* = 8.5 Hz), 7.24 (m, 1H), 7.34 (m, 1H), 7.39 (d, 1H, ^3^*J* = 15.7 Hz), 7.63 (d, 1H, ^3^*J* = 7.3 Hz), 7.71 (d, 1H,^3^*J* = 7.6 Hz), 8.04 (d, 2H, ^3^*J* = 8.5 Hz), 8.12 (d, 1H, ^3^*J* = 15.7 Hz); ^13^C-NMR (CDCl_3_) *δ* (ppm): 65.69 (q, ^2^*J*_C-F_ = 36.1 Hz), 114.77, 123.20 (q, ^1^*J*_C-F_ = 277.60 Hz), 124.88, 125.99, 127.85, 128.02, 131.21, 131.45, 132.55, 133.72, 135.25, 143.11, 160.94, 188.78; ^19^F-NMR (CDCl_3_) *δ* (ppm): −73.67 (t, 3F, ^3^*J* = 7.7 Hz); IR (KBr) *ν*: 3072, 2952, 2821, 1660, 1607, 1511, 1463, 1435, 1344, 1299, 1220, 1184, 1076, 1027, 970, 865, 830, 757, 739, 688, 657 cm^−1^; HRMS (ESI) calcd. for C_17_H_12_BrF_3_O_2_: [M + H]^+^ 385.0051; Found: 385.0051.

*(E)-3-(3-Bromophenyl)-1-(4-(2,2,2-trifluoroethoxy)phenyl)prop-2-en-1-one* (**1j**)**.** Pale yellow solid, Yield: 74%, m.p. 104–106 °C; ^1^H-NMR (CDCl_3_) *δ* (ppm): 4.45 (q, 2H, ^3^*J* = 7.9 Hz), 7.04 (d, 2H, ^3^*J* = 8.9 Hz), 7.28–7.32 (m, 1H), 7.50–7.55 (m, 3H), 7.73 (m, 1H), 7.80 (s, 1H), 8.07 (d, 2H,*^3^J* = 8.9 Hz); ^13^C-NMR (CDCl_3_) *δ* (ppm): 65.70 (q, ^2^*J*_C-F_ = 36.4 Hz), 114.80, 122.95, 123.24, 127.43, 130.63, 130.90, 131.11, 132.57, 133.38, 137.17, 142.87, 161.01, 188.32; ^19^F-NMR (CDCl_3_) *δ* (ppm): −73.63 (t, 3F, ^3^*J* = 8.8 Hz); IR (KBr) *ν*: 3064, 2948, 2894, 2361, 1663, 1611, 1511, 1418, 1341, 1255, 1220, 1182, 1127, 1078, 1030, 971, 830, 780, 669, 661 cm^−1^; HRMS (ESI) calcd. for C_17_H_12_BrF_3_O_2_: [M + H]^+^ 385.0051; Found: 385.0019.

*(E)-3-(4-Methoxyphenyl)-1-(4-(2,2,2-trifluoroethoxy)phenyl)prop-2-en-1-one* (**1k**)**.** Yellow solid, Yield: 72%, m.p. 100–102 °C; ^1^H-NMR (CDCl_3_) *δ* (ppm): 3.86 (s, 3H), 4.44 (q, 2H, ^3^*J* = 7.9 Hz), 6.94 (d, 2H, ^3^*J* = 8.8 Hz), 7.03 (d, 2H, ^3^*J* = 8.8 Hz),7.41 (d, 1H, ^3^*J* = 15.9 Hz), 7.61 (d, 2H, ^3^*J* = 8.8 Hz), 7.79 (d, 1H, ^3^*J* = 15.9 Hz), 8.05 (d, 2H, ^3^*J* = 8.8 Hz); ^13^C-NMR (CDCl_3_) *δ* (ppm): 55.56, 65.69 (q, ^2^*J*_C-F_ = 35.9 Hz), 114.57, 114.67, 119.41, 127.77, 130.36, 130.93, 133.15, 144.61, 160.68, 161.82, 188.85; ^19^F-NMR (CDCl_3_) *δ* (ppm): −73.69 (t, 3F, ^3^*J* = 7.7 Hz); IR (KBr) *ν*: 3042, 3073, 2957, 2839, 1657, 1603, 1572, 1510, 1423, 1344, 1295, 1249, 1168, 1033, 976, 813, 680 cm^−1^; HRMS(ESI) calcd. for C_18_H_15_F_3_O_3_: [M + H]^+^ 337.1051 and [M + Na]^+^ 359.0871; Found: 37.1051 and 359.0864.

*(E)-3-(3-Methoxyphenyl)-1-(4-(2,2,2-trifluoroethoxy)phenyl)prop-2-en-1-one* (**1l**)**.** Pale yellow solid, Yield: 91%, m.p. 78–80 °C; ^1^H-NMR (CDCl_3_) *δ* (ppm): 3.86 (s, 3H), 4.44 (q, 2H, ^3^*J* = 7.9 Hz), 6.96–6.98 (m, 1H), 7.04 (d, 2H, ^3^*J* = 8.7 Hz), 7.15–7.16 (m, 1H), 7.23 (s, 1H), 7.34 (t, 1H, ^3^*J* = 8.2 Hz), 7.54 (d, 1H, ^3^*J* = 15.7 Hz), 7.77 (d, 1H, ^3^*J* = 15.7 Hz), 8.06 (d, 2H, ^3^*J* = 8.7 Hz); ^13^C-NMR (CDCl_3_) *δ* (ppm): 55.51, 65.63 (q, ^2^*J*_C-F_ = 39.0 Hz), 113.63, 114.73, 116.37, 121.21, 122.06, 130.12, 131.07, 132.82, 136.41, 144.68, 160.09, 160.84, 188.82; ^19^F-NMR (CDCl_3_) *δ* (ppm): −73.66 (t, 3F, ^3^*J* = 8.8 Hz); IR (KBr) *ν*: 3079, 3004, 2941, 2833, 1650, 1606, 1510, 1426, 1314, 1264, 1217, 1158, 1026, 978, 818, 677 cm^−1^; HRMS (ESI) Calc. for C_18_H_15_F_3_O_3_: [M + H]^+^ 337.1051 and [M + Na]^+^ 359.0871; Found: 337.1048 and 359.0865.

*(E)-3-(3,4-Dimethoxyphenyl)-1-(4-(2,2,2-trifluoroethoxy)phenyl)prop-2-en-1-one* (**1m**)**.** Yellow solid, Yield: 70%, m.p. 116–118 °C; ^1^H-NMR (CDCl_3_) *δ* (ppm): 3.94 (s, 3H), 3.96 (s, 3H), 4.44 (q, 2H, ^3^*J* = 7.9 Hz), 6.90 (d, 1H, ^3^*J* = 8.9 Hz), 7.04 (d, 2H, ^3^*J* = 8.7 Hz), 7.16 (d, 1H, ^3^*J* = 1.9 Hz), 7.24 (dd, 1H, ^3^*J* = 8.3 Hz, ^4^*J* = 1.9 Hz) 7.38 (d, 1H, ^3^*J* = 15.7 Hz), 7.77 (d, 1H, ^3^*J* = 15.7 Hz), 8.05 (d, 2H, ^3^*J* = 8.7 Hz); ^13^C-NMR (CDCl_3_) *δ* (ppm): 56.12, 56.15, 65.69 (q, ^2^*J*_C-F_ = 36.0 Hz), 110.23, 111.26, 119.69, 123.28, 128.00, 130.96, 133.10, 144.95, 149.39, 151.58, 160.70, 188.89; ^19^F-NMR (CDCl_3_) *δ* (ppm): −73.67 (t, 3F, ^3^*J*= 7.6 Hz); IR (KBr) *ν*: 3079, 3003, 2941, 2833, 1651, 1606, 1590, 1510, 1425, 1361, 1236, 1217, 1180, 1141, 978, 817, 791, 677 cm^−1^; HRMS (ESI) calcd. for C_19_H_17_F_3_O_4_: [M + H]^+^ 367.1157 and [M + Na]^+^ 389.0977; Found: 367.1157 and 389.0987.

*(E)-3-(1H-Indol-2-yl)-1-(4-(2,2,2-trifluoroethoxy)phenyl)prop-2-en-1-one* (**1n**)**.** Yellow solid, Yield: 40%, m.p. 194–195 °C; ^1^H-NMR (CDCl_3_) *δ* (ppm): 4.44 (q, 2H, ^3^*J* = 8.0 Hz), 7.05 (d, 2H, ^3^*J* = 8.0 Hz), 7.31–7.33 (m, 2H), 7.45–7.47 (m, 1H), 7.57–7.62 (m, 2H), 8.03 (dd, 1H, ^3^*J* = 6.7 Hz, ^4^*J* = 3.0 Hz), 8.09–8.13 (m, 3H), 8.61 (s, 1H); ^13^C-NMR (CDCl_3_) *δ* (ppm): 65.68 (q, ^2^*J*_C-F_ **=** 35.9), 112.14, 114.64, 117.50, 120.84, 121.92, 123.72, 125.48, 130.46, 130.80, 133.55, 137.38, 138.83, 160.47, 189.26; ^19^F-NMR (CDCl_3_) *δ* (ppm): −73.68 (t, 3F, ^3^*J* = 8.7 Hz); IR (KBr) *ν*: 3219, 2929, 1647, 1604, 1587, 1554, 1365, 1246, 1170, 1041, 815, 735, 677 cm^−1^; HRMS (ESI) calcd. for C_19_H_14_F_3_NO_2_: [M + H]^+^ 346.1055; Found: 346.1051.

*(E)-3-(4-Methylphenyl)-1-(3-(2,2,2-trifluoroethoxy)phenyl)prop-2-en-1-one* (**2a**)**.** Yellow solid, Yield: 80%, m.p. 107–108 °C; ^1^H-NMR (CDCl_3_) *δ* (ppm): 2.42 (s, 3H), 4.45 (q, 2H, ^3^*J* = 8.1 Hz), 7.20 (dd, 1H, ^3^*J* = 8.2 Hz, ^4^*J* = 2.5 Hz), 7.24–7.27 (m, 2H), 7.46–7.50 (m, 2H), 7.56–7.58 (m, 3H), 7.72 (d, 1H, ^3^*J* = 8.0 Hz), 7.83 (d, 1H, ^3^*J* = 15.6 Hz); ^13^C-NMR (CDCl_3_) *δ* (ppm): 21.70, 65.99 (q, ^2^*J*_C-F_ = 36.1 Hz), 113.82, 120.10, 120.79, 122.84, 128.72, 129.90, 130.10, 132.12, 140.13, 141.50, 145.62, 157.76, 189.88; ^19^F-NMR (CDCl_3_) *δ* (ppm): −73.7 (t, 3F, ^3^*J* = 7.8 Hz); IR (KBr) *ν*: 3083, 3049, 2360, 1658, 1595, 1580, 1454, 1325, 1305, 1247, 1161, 1076, 1041, 967, 812, 800, 741, 674 cm^−1^; HRMS (ESI) Calc. for C_18_H_15_F_3_O_2_: [M + H]^+^ 321.1124; Found: 321.1093.

*(E)-3-(4-(Dimethylamino)phenyl)-1-(3-(2,2,2-trifluoroethoxy)phenyl)prop-2-en-1-on*e (**2b**)**.** Yellow solid, Yield: 71%, m.p. 134–136 °C; ^1^H-NMR (CDCl_3_) *δ* (ppm): 3.05 (s, 6H), 4.44 (q, 2H, ^3^*J* = 8.1 Hz), 6.69 (d, 2H, ^3^*J* = 9.2 Hz), 7.16 (dd, 1H, ^3^*J*= 8.3 Hz, ^4^*J* = 2.5 Hz), 7.30 (d, 1H, ^3^*J* = 15.6 Hz), 7.44 (t, 1H, ^3^*J* = 7.9 Hz), 7.54–7.57 (m, 3H), 7.69 (d, 1H, ^3^*J* = 7.9 Hz), 7.81 (d, 1H, ^3^*J* = 15.6 Hz); ^13^C-NMR (CDCl_3_) *δ* (ppm): 40.27, 66.00 (q, ^2^*J*_C-F_ = 35.8 Hz), 111.96, 113.69, 116.56, 119.62, 122.61, 122.67, 129.93, 130.71, 140.92, 146.54, 152.30, 157.69, 189.86; ^19^F-NMR (CDCl_3_) *δ* (ppm): −73.71 (t, 3F, ^3^*J* = 7.6Hz); HRMS (ESI calc. for C_19_H_18_F_3_NO_2_: [M + H]^+^ 350.1368; Found: 350.1362.

*(E)-3-(4-Chlorophenyl)-1-(3-(2,2,2-trifluoroethoxy)phenyl)prop-2-en-1-one* (**2c**)**.** Yellow solid, Yield: 73%, m.p. 117–118 °C; ^1^H-NMR (CDCl_3_) *δ* (ppm): 4.44 (q, 2H, ^3^*J* = 8.1 Hz), 7.20 (dd, 1H, ^3^*J* = 8.8 Hz, ^4^*J* = 2.7 Hz) 7.40–7.42 (m, 2H), 7.46–7.50 (m, 2H), 7.58–7.60 (m, 3H), 7.70 (d, 1H, ^3^*J* = 7.9 Hz), 7.78 (d, 1H, ^3^*J* = 15.7 Hz); ^13^C-NMR (CDCl_3_) *δ* (ppm): 65.99 (q, ^2^*J*_C-F_ = 35.8 Hz), 113.90, 120.31, 122.17, 122.86, 129.45, 129.81, 130.19, 133.34, 136.80, 139.80, 143.97, 157.82, 189.50; ^19^F-NMR (CDCl_3_) *δ* (ppm): −73.76 (t, 3F, ^3^*J* = 8.7 Hz); IR (KBr) *ν*: 3104, 3081, 2945, 2360, 1664, 1610, 1581, 1490, 1451, 1295, 1273, 1250, 1170, 1152, 1045, 973, 914, 868, 821, 789, 690 cm^−1^; HRMS (ESI) calc. for C_17_H_12_ClF_3_O_2_: [M + H]^+^ 341.0556 and [M + Na]^+^ 363.0376; Found: 341.0555 and 363.0375.

*(E)-3-(4-Bromophenyl)-1-(3-(2,2,2-trifluoroethoxy)phenyl)prop-2-en-1-one* (**2d**)**.** Pale yellow solid, Yield: 75%, m.p. 125–126 °C; ^1^H-NMR (CDCl_3_) *δ* (ppm): 4.44 (q, 2H, ^3^*J* = 8.0 Hz), 7.20 (dd, 1H, ^3^*J* = 8.4 Hz, ^4^*J* = 2.7 Hz), 7.45–7.58 (m, 7H), 7.70 (d, 1H, ^3^*J* = 8.4 Hz), 7.76 (d, 1H, ^3^*J* = 15.6 Hz); ^13^C-NMR (CDCl_3_) *δ* (ppm): 66.64 (q, ^2^*J*_C-F_ = 36.5 Hz), 113.96, 120.32, 122.39, 122.88, 125.19, 130.01, 130.20, 132.41, 133.79, 139.90, 144.03, 157.84, 189.75; ^19^F-NMR (CDCl_3_) *δ* (ppm): −73.7 (t, 3F, ^3^*J* = 7.7 Hz); IR (KBr) *ν*: 3076, 2945, 2360, 1667, 1599, 1486, 1450, 1401, 1314, 1295, 1170, 1152, 1080, 1045, 973, 867, 816, 788, 688 cm^−1^; HRMS (ESI) calc. for C_17_H_12_BrF_3_O_2_: [M + H]^+^ 385.0051 and [M + Na]^+^ 406.9871; Found: 385.0054 and 406.9879.

*(E)-3-(3-Nitrophenyl)-1-(3-(2,2,2-trifluoroethoxy)phenyl)prop-2-en-1-one* (**2e**)**.** Brown solid, Yield: 73%, m.p. 78–80 °C; ^1^H-NMR (CDCl_3_) *δ* (ppm): 4.45 (q, 2H, ^3^*J* = 8.0 Hz), 7.23 (dd, 1H, ^3^*J* = 8.5 Hz, ^4^*J* = 3.0 Hz), 7.50 (t, 1H, ^3^*J* = 8.1 Hz), 7.59–7.65 (m, 3H), 7.74 (d, 1H, ^3^*J* = 7.8 Hz), 7.85 (m, 1H), 7.93 (d, 1H, ^3^*J* = 7.2 Hz), 8.27 (dd, ^3^*J* = 8.0 Hz, ^4^J = 2.7 Hz), 8.51–8.8.52 (m, 1H); ^13^C-NMR (CDCl_3_) *δ* (ppm): 66.01 (q, ^2^*J*_C-F_ = 35.2 Hz), 113.95, 120.67, 122.58, 122.98, 124.36, 130.25, 130.34, 134.51, 136.62, 139.33, 142.28, 148.89, 157.91, 188.96; **^1^**^9^F-NMR (CDCl_3_) *δ* (ppm): −73.39 (t, 3F, ^3^*J* = 8.8 Hz); IR (KBr) *ν*: 3074, 2869, 2360, 1662, 1591, 528, 1444, 1357, 1309,1272, 1248, 1173, 1158, 1083, 1068, 974, 911, 857, 795, 780, 740, 675 cm^−1^; HRMS (ESI) calc. for C_17_H_12_F_3_NO_4_: [M + H]^+^ 352.0797; Found: 352.0789.

*(E)-3-(2,5-Dimethoxyphenyl)-1-(3-(2,2,2-trifluoroethoxy)phenyl)prop-2-en-1-one* (**2f**)**.** Yellow solid, Yield: 77%, m.p. 72–74 °C; ^1^H-NMR (CDCl_3_) *δ* (ppm): 3.83 (s, 3H), 3.88 (s, 3H), 4.44 (q, 2H, ^3^*J* = 8.1 Hz), 6.89 (m, 1H), 6.94–6.97 (m,1H), 7.16–7.18 (m, 1H), 7.20 (d, 1H, ^4^*J* = 2.7 Hz), 7.46 (t, 1H, ^3^*J* = 8.0 Hz), 7.53–7.57 (m, 2H), 7.69 (d, 1H, ^3^*J* = 8.4 Hz), 8.09 (d, 1H, ^3^*J* = 16 Hz); ^13^C-NMR (CDCl_3_) *δ* (ppm): 56.01, 56.25, 66.05 (q, ^2^*J*_C-F_ = 35.90 Hz), 112.64, 113.90, 113.99, 117.60, 120.10, 122.01, 122.96, 124.49, 130.06, 140.25, 140.86, 153.56, 153.70, 157.77, 190.40; ^19^F-NMR (CDCl_3_) *δ* (ppm): −73.7 (t, 3F, ^3^*J* = 7.7 Hz); IR (KBr) *ν*: 2948, 2838, 2357, 1659, 1587, 1494, 1442, 1323, 1248, 1172, 1123, 1047, 985, 974, 848, 791, 693 cm^−1^; HRMS (ESI) calc. for C_19_H_17_F_3_O_4_: [M + H]^+^ 367.1157; Found: 367.1158.

*(E)-3-(Furan-2-yl)-1-(3-(2,2,2-trifluoroethoxy)phenyl)prop-2-en-1-one* (**2g**)**.** Brown viscous liquid, Yield: 65%, ^1^H-NMR (CDCl_3_) *δ* (ppm): 4.39 (q, 2H, ^3^*J* = 7.90 Hz), 6.44–6.45 (m, 1H), 6.64 (d, 1H, ^3^*J* = 3.5 Hz), 6.9 (d, 1H, ^3^*J* = 8.4 Hz), 7.09–7.13 (m,1H), 7.21–7.22 (m, 1H), 7.36–7.46 (m, 3H), 7.64 (d, 1H, ^3^*J* = 8.0 Hz); ^13^C-NMR (CDCl_3_) *δ* (ppm): 65.58 (q, 2H, ^3^*J* = 36.2 Hz,) 112.94, 113.61, 116.87, 118.98, 120.29, 122.83, 130.14, 131.26, 139.89, 145.29, 157.76, 189.14; ^19^F-NMR (CDCl_3_) *δ* (ppm): −73.71 (t, ^3^*J* = 8.7 Hz); IR (KBr) *ν*: 3126, 1688, 1586, 1486, 1442, 1401, 1250, 1169, 1069, 1016, 973, 858, 789, 734, 680 cm^−1^; HRMS (ESI) calc. for C_15_H_11_F_3_O_3_: [M + H]^+^ 297.0739; Found: 297.0730.

*(E)-3-(5-Bromothiophen-2-yl)-1-(3-(2,2,2-trifluoroethoxy)phenyl)prop-2-en-1-one* (**2h**)**.** Yellow solid, Yield: 85%, m.p. 103–105 °C; ^1^H-NMR (CDCl_3_) *δ* (ppm): 4.44 (q, 2H, ^3^*J* = 8.1 Hz), 7.07 (m, 1H), 7.12 (m, 1H), 7.17–7.21 (m, 2H), 7.45–7.48 (m, 1H), 7.54–7.55 (m, 1H), 7.66 (d, 1H, ^3^*J* = 7.6 Hz), 7.83 (d, 1H, ^3^*J* = 15.7 Hz); ^13^C-NMR (CDCl_3_) *δ* (ppm): 65.94 (q, ^2^*J*_C-F_ = 36.1 Hz), 113.65, 116.90, 120.35, 120.59, 121.94, 122.74, 130.19, 131.55, 132.84, 136.85, 139.65, 141.89, 157.78, 188.82; ^19^F-NMR (CDCl_3_) *δ* (ppm): −73.75 (t, 3F, ^3^*J* = 8.8 Hz); IR (KBr) *ν*: 3073, 2941, 1658, 1579, 1488, 1423, 1346, 1264, 1247, 1162, 1070, 1057, 970, 917, 854, 814, 783, 716, 697 cm^−1^; HRMS (ESI) calc. for C_15_H_10_BrF_3_O_2_S: [M + H]^+^ 390.9615; Found: 390.961.

*(E)-3-(4-Methoxyphenyl)-1-(3-(2,2,2-trifluoroethoxy)phenyl)prop-2-en-1-one* (**2k**)**.** Yellow solid, Yield: 73%, m.p. 79–82 °C; ^1^H-NMR (CDCl_3_) *δ* (ppm): 3.86 (s, 3H) 4.44 (q, 2H, ^3^*J* = 8.0 Hz), 6.94 (d, 2H, ^3^*J* = 8.3 Hz), 7.18 (dd, 1H, ^3^*J* = 8.6 Hz, ^4^*J* = 2.6 Hz),7.38 (d, 1H, ^3^*J* = 15.6 Hz),7.46 (t, 1H, ^3^*J* = 8.2 Hz), 7.56–7.57 (m, 1H), 7.61 (d, 2H, ^3^*J* = 8.4 Hz), 7.70 (d, 1H, ^3^*J* = 8.0 Hz), 7.80 (d, 1H, ^3^*J* = 15.6 Hz); ^13^C-NMR (CDCl_3_) *δ* (ppm): 55.57, 65.95 (q, ^2^*J*_C-F_ = 35.40), 113.75, 114.59, 119.43, 119.98, 122.77, 127.57, 130.07, 130.51, 140.27, 145.38, 157.73, 161.98, 189.81; ^19^F-NMR (CDCl_3_) *δ* (ppm): −73.7 (t, 3F, ^3^*J* = 8.8 Hz); IR (KBr) *ν*: 3075, 3014, 2941, 2843, 1655, 1587, 1573, 1442, 1342, 1287, 1245, 1187, 1156, 1056, 1027, 972, 921, 872, 824, 798, 788, 669 cm^−1^; HRMS (ESI) calc. for C_18_H_15_F_3_O_3_: [M + H]^+^ 337.1052; Found: 337.1048.

*(E)-3-(3,4-Dimethoxyphenyl)-1-(3-(2,2,2-trifluoroethoxy)phenyl)prop-2-en-1-one* (**2m**)**.** Yellow solid, Yield: 64%, ^1^H-NMR (CDCl_3_) *δ* (ppm): 3.94 (s, 3H), 3.96 (s, 3H), 4.44 (q, 2H, ^3^*J* = 8.1 Hz), 6.91 (d, 1H, ^3^*J* = 8.0 Hz), 7.17–7.18 (m,1H), 7.19–7.24 (dd, 1H),7.36 (d, 1H, ^3^*J* = 15.6 Hz), 7.47 (t, 1H, ^3^*J* = 8.0 Hz), 7.57 (s, 1H), 7.70 (d, 1H, ^3^*J* = 8.0 Hz), 7.78 (d, 1H, ^3^*J* = 15.6 Hz); ^13^C-NMR (CDCl_3_) *δ* (ppm): 55.94, 56.11, 65.94 (q, ^2^*J*_C-F_ = 36.1 Hz), 110.14, 111.20, 113.85, 119.68, 119.89, 122.78, 123.53, 127.80, 130.06, 140.23, 145.73, 149.37, 151.71, 157.72, 189.85; ^19^F-NMR (CDCl_3_) *δ* (ppm): −73.75 (t, 3F, ^3^*J* = 8.8 Hz); IR (KBr) *ν*: 3128, 2840, 1656, 1576, 1517, 1421, 1256, 1165, 1080, 1026, 977, 919, 785, 724, 679 cm^−1^; HRMS (ESI) Calc. for C_19_H_17_F_3_O_4_: [M + H]^+^ 367.1157 and [M + Na]^+^ 389.0977; Found: 367.1154 and 389.0979.

*(E)-3-(4-Methylphenyl)-1-(2-(2,2,2-trifluoroethoxy)phenyl)prop-2-en-1-one* (**3a**)**.** Pale yellow solid, Yield: 82%, m.p. 80–82 °C; ^1^H-NMR (CDCl_3_) *δ* (ppm): 2.38 (s, 3H), 4.44 (q, 2H, ^3^*J* = 8.0 Hz), 6.96 (d, 1H, ^3^*J* = 8.0 Hz), 7.15–7.17 (m, 1H), 7.20 (d, 2H, ^3^*J* = 8.0 Hz), 7.38 (m, 1H), 7.49 (d, 2H, ^3^*J* = 7.6 Hz), 7.64 (m, 1H), 7.70 (d, 1H, ^3^*J* = 7.8 Hz), 7.78 (d, 1H, ^3^*J* = 7.8 Hz); ^13^C-NMR (CDCl_3_) *δ* (ppm): 21.66, 66.53 (q, ^2^*J*_C-F_ = 35.8 Hz), 113.21, 123.05, 125.55, 128.65, 129.78, 130.36, 131.19, 132.27, 133.02, 141.11, 144.30, 155.60, 192.08; ^19^F-NMR (CDCl_3_) *δ* (ppm): −73.51 (t, 3F, ^3^*J* = 8.8 Hz); IR (KBr) *ν*: 3115.70, 1648.15, 1604.14, 1484.69, 1449.97, 1403.88, 1330.59, 1265.53, 1163.65, 119.59, 978, 813.90, 761.55, 682.04 cm^−1^; HRMS (ESI) calc. for C_18_H_15_F_3_O_2_: [M + H]^+^ 321.1102; Found: 321.1095.

*(E)-3-(4-(Dimethylamino)phenyl)-1-(2-(2,2,2-trifluoroethoxy)phenyl)prop-2-en-1-one* (**3b**)**.** Yellow solid, Yield: 72%, m.p. 64–66 °C; ^1^H-NMR (CDCl_3_) *δ* (ppm): 3.05 (s, 6H), 4.44 (q, 2H, ^3^*J* = 8.1 Hz), 6.69–6.71 (m, 2H), 7.14–7.17 (m, 1H), 7.28–7.32 (m,1H), 7.44 (t, 1H), 7.54–7.57 (m, 3H), 7.68–7.70 (m, 1H), 7.79–7.83 (m, 1H); ^13^C-NMR (CDCl_3_) *δ* (ppm): 40.27, 65.65 (q, ^2^*J*_C-F_ = 36.20 Hz), 111.93, 114.53, 116.43, 121.87, 122.72, 124.63, 130.54, 130.75, 133.63, 145.75, 152.14, 160.36, 188.96; ^19^F-NMR (CDCl_3_) *δ* (ppm): −74.19 (t, 3F, ^3^*J* = 7.6 Hz); IR (KBr) *ν*: 2821, 2359, 1646, 1599, 1520, 1484, 1447, 1339, 1266, 1233, 1162, 1064, 1025, 970, 861, 815, 767, 753, 676 cm^−1^; HRMS (ESI) calc. for C_19_H_18_F_3_NO_2_: [M + H]^+^ 350.1368; Found: 350.1366.

*(E)-3-(4-Chlorophenyl)-1-(2-(2,2,2-trifluoroethoxy)phenyl)prop-2-en-1-one* (**3c**)**.** Yellow solid, Yield: 73%, m.p. 61–63 °C; ^1^H-NMR (CDCl_3_) *δ* (ppm): 4.45 (q, 2H, ^3^*J* = 8.0 Hz), 6.95 (d, 1H, ^3^*J* = 8.4 Hz), 7.17 (t, 1H, ^3^*J* = 7.7 Hz), 7.36–7.40 (m, 3H), 7.49–7.53 (m, 3H), 7.62 (m, 1H), 7.73 (dd, 1H, ^3^*J* = 7.6 Hz, ^4^*J* = 1.9 Hz); ^13^C-NMR (CDCl_3_) *δ* (ppm): 66.37 (q, ^2^*J*_C-F_ = 36.0 Hz), 112.94, 123.10, 126.91, 129.32, 129.73, 129.91, 131.40, 133.42, 133.58, 136.41, 142.31, 155.69, 191.49; ^19^F-NMR (CDCl_3_) *δ* (ppm): −73.70 (t, 3F, ^3^*J* = 7.6 Hz); IR (KBr) *ν*: 2884, 2361, 1651, 1602, 1588, 1485, 1447, 1330, 1260, 1227, 1196, 1069, 1024, 979, 863, 821, 769, 693 cm^−1^; HRMS (ESI) calc. for C_17_H_12_ClF_3_O_2_: [M + H]^+^ 341.0556 and [M + Na]^+^ 363.0376;Found: 341.0545 and 363.0363.

*(E)-3-(4-Bromophenyl)-1-(2-(2,2,2-trifluoroethoxy)phenyl)prop-2-en-1-one* (**3d**)**.** Yellow solid, Yield: 73%, m.p. 68–71 °C; ^1^H-NMR (CDCl_3_) *δ* (ppm): 4.45 (q, 2H, ^3^*J* = 8.0 Hz), 6.95 (d, 1H, ^3^*J* = 8.0 Hz), 7.17 (t, 1H, ^3^*J* = 7.4 Hz), 7.43 (d, 1H, ^3^*J* = 15.9 Hz), 7.45 (d, 2H, ^3^*J* = 8.0 Hz), 7.53 (d, 2H, ^3^*J* = 8.5 Hz), 7.61 (d, 1H, ^3^*J* = 15.9 Hz), 7.73 (d, 1H, ^3^*J* = 7.9 Hz); ^13^C-NMR (CDCl_3_) *δ* (ppm): 66.34 (q, ^2^*J*_C-F_ = 35.1 Hz), 112.89, 123.09, 124.62, 124.78, 126.98, 129.92, 131.40, 132.27, 133.44, 133.99, 142.33, 155.68, 191.46; ^19^F-NMR (CDCl_3_) *δ* (ppm): −73.54 (t, 3F, ^3^*J* = 7.6 Hz); IR (KBr) *ν*: 2855, 2361, 1652, 1603, 1586, 1486, 1450, 1329, 1276, 1198, 1071, 1022, 819, 764, 692 cm^−1^; HRMS (ESI) calc. for C_17_H_12_BrF_3_O_2_: [M + H]^+^ 385.0051; Found: 385.0042.

*(E)-3-(3-Nitrophenyl)-1-(2-(2,2,2-trifluoroethoxy)phenyl)prop-2-en-1-one* (**3e**)**.** Brown solid, Yield: 75%, m.p. 170–172 °C; ^1^H-NMR (CDCl_3_) *δ* (ppm): 4.48 (q, 2H, ^3^*J* = 7.9 Hz), 6.96 (d, 1H, ^3^*J* = 8.4 Hz), 7.17–7.21 (m, 1H), 7.53–7.61 (m, 3H), 7.69–7.72 (m, 1H), 7.78 (dd, 1H, ^3^*J* = 7.7 Hz, ^4^*J* = 1.9 Hz), 7.89 (d, 1H, ^3^*J* = 8.1 Hz), 8.24 (d, 1H, ^3^*J* = 8.8 Hz), 8.46 (s, 1H); ^13^C-NMR (CDCl_3_) *δ* (ppm): 66.21 (q, ^2^*J*_C-F_ = 35.7 Hz), 112.66, 122.72, 123.14, 124.64, 129.04, 129.33, 130.06, 131.59, 133.92, 134.12, 136.91, 140.25, 148.87, 155.83, 190.86; ^19^F-NMR (CDCl_3_) *δ* (ppm): −73.67 (t, 3F, ^3^*J* = 8.7 Hz); IR (KBr) *ν*: 3113, 3093, 2954, 2359, 1651, 1604, 1527, 1487, 1448, 1331, 1271, 1230, 1061, 1021, 979, 865, 769, 747, 670 cm^−1^; HRMS (ESI) calc. for C_17_H_12_F_3_NO_4_: [M + H]^+^ 352.0796 and [M + Na]^+^ 374.0616; Found: 352.0798 and 374.0616.

*(E)-3-(2,5-Dimethoxyphenyl)-1-(2-(2,2,2-trifluoroethoxy)phenyl)prop-2-en-1-one* (**3f**)**.** Yellow solid, Yield: 62%, 77–79 °C; ^1^H-NMR (CDCl_3_) *δ* (ppm): 3.79 (s, 3H), 3.82 (s, 3H), 4.43 (q, 2H, ^3^*J* = 8.1 Hz), 6.83–6.86 (m, 1H), 6.91–6.96 (m, 2H), 7.11–7.17 (m, 2H), 7.39–7.43 (m, 1H), 7.46–7.50 (m, 1H), 7.69 (dd, 1H, ^3^*J* = 7.7 Hz, ^4^*J* = 2.1 Hz), 8.02 (d, 1H, ^3^*J* = 16.0 Hz); ^13^C-NMR (CDCl_3_) *δ* (ppm): 55.84, 56.24, 66.64 (q, ^2^*J*_C-F_ = 35.8 Hz), 112.38, 112.69, 113.39, 118.18, 123.08, 124.51, 126.82, 130.61, 131.20, 132.91, 139.20, 153.43, 153.69, 155.63, 192.34; ^19^F-NMR (CDCl_3_) *δ* (ppm): −73.45 (t, 3F, ^3^*J* = 8.8 Hz); IR (KBr) *ν*: 2942, 2836, 2358, 1657, 1601, 1587, 1497, 1338, 1220, 1164, 1043, 978, 863, 820, 751, 680 cm^−1^; HRMS (ESI) calc. for C_19_H_17_F_3_O_4_: [M + H]^+^ 367.1157; Found: 367.1132.

*(E)-3-(Furan-2-yl)-1-(2-(2,2,2-trifluoroethoxy)phenyl)prop-2-en-1-one* (**3g**)**.** Brown solid, Yield: 59%, m.p. 72–74 °C, ^1^H-NMR (CDCl_3_) *δ* (ppm): 4.44 (q, 2H, ^3^*J* = 7.9 Hz), 6.48–6.50 (m, 1H), 6.69 (d, 1H, ^3^*J* = 3.5 Hz), 6.95 (d, 1H, ^3^J = 8.4 Hz), 7.14–7.18 (m, 1H), 7.27–7.31 (m, 1H), 7.41–7.51 (m, 3H), 7.69 (d, 1H, ^3^*J* = 8 Hz); ^13^C-NMR (CDCl_3_) *δ* (ppm): 66.63 (q, ^2^*J*_C-F_ = 36.0 Hz), 112.68, 113.41, 115.88, 123.05, 124.17, 130.15, 130.27, 131.11, 133.08, 145.17, 151.79, 155.73, 191.56; ^19^F-NMR (CDCl_3_) *δ* (ppm): −73.54 (t, 3F, ^3^*J* = 8.7 Hz); IR (KBr) *ν*: 3040, 2901, 2360, 2342, 1645, 1603, 1587, 1487, 1449, 1330, 1295, 1206, 1016, 969, 868, 771, 746, 675 cm^−1^; HRMS (ESI) calc. for C_15_H_11_F_3_O_3_: [M + H]^+^ 297.0738; Found: 297.0735.

*(E)-3-(5-Bromothiophen-2-yl)-1-(2-(2,2,2-trifluoroethoxy)phenyl)prop-2-en-1-one* (**3h**)**.** Yellow solid, Yield: 87%, m.p. 94–95 °C; ^1^H-NMR (CDCl_3_) *δ* (ppm): 4.45 (q, 2H, ^3^*J* = 8.0 Hz), 6.94 (d, 1H, ^3^*J* = 8.4 Hz), 7.03–7.07 (m, 2H), 7.14–7.18 (m, 2H), 7.48–7.53 (m, 1H), 7.67–7.73 (m, 2H); ^13^C-NMR (CDCl_3_) *δ* (ppm): 66.64 (q, ^2^*J*_C-F_ = 36.3 Hz), 113.00, 116.60, 123.08, 123.23 (q, ^1^*J*_C-F_ = 279.6 Hz), 125.52, 129.83, 131.39, 132.20, 133.41, 135.25, 142.20, 142.60, 155.73, 190.75; ^19^F-NMR (CDCl_3_) *δ* (ppm): −73.3 (t, 3F, ^3^*J* = 7.5 Hz); IR (KBr) *ν*: 3096, 2361, 1645, 1580, 1597, 1568, 1427, 1347, 1296, 1270, 1170, 1159, 1023, 979, 966, 867, 773, 675 cm^−1^; HRMS (ESI) calc. for C_18_H_15_F_3_O_2_: [M + H]^+^ 321.1102; Found: 321.1093.

*(E)-3-(2-Bromophenyl)-1-(2-(2,2,2-trifluoroethoxy)phenyl)prop-2-en-1-one* (**3i**)**.** Pale yellow solid, Yield: 74%, m.p. 76–77 °C; ^1^H-NMR (CDCl_3_) *δ* (ppm): 4.45 (q, 2H, ^3^*J* = 8.0 Hz), 6.96 (d, 1H, ^3^*J* = 8.4 Hz), 7.18 (t, 1H, ^3^*J* = 7.8 Hz), 7.20–7.23 (m, 1H), 7.32–7.37 (m, 2H), 7.49–7.54 (m, 1H), 7.61 (d, 1H, ^3^J = 7.4 Hz), 7.70–7.74 (m, 2H), 8.02 (d, 1H, ^3^*J* = 15.7 Hz); ^13^C-NMR (CDCl_3_) *δ* (ppm): 66.47 (q, ^2^*J*_C-F_ = 35.9 Hz), 113.03, 121.86, 123.12, 126.17, 127.83, 127.88, 128.99, 129.89, 131.37, 131.41, 133.36, 133.59, 142.33, 155.68, 191.73; ^19^F-NMR (CDCl_3_) *δ* (ppm): −73.48 (t, 3F, ^3^*J* = 6.5 Hz); IR (KBr) *ν*: 3060, 2902, 2342, 1646, 1604, 1486, 1450, 1329, 1278, 1201, 1149, 1120, 1023, 970, 864, 753, 693 cm^−1^; HRMS (ESI) calc. for C_17_H_12_BrF_3_O_2_: [M + H]^+^ 385.0051 and [M + Na]^+^ 406.9871; Found: 385.0042 and 406.9854.

*(E)-3-(3-Bromophenyl)-1-(2-(2,2,2-trifluoroethoxy)phenyl)prop-2-en-1-one* (**3j**)**.** Yellow solid, Yield: 74%, m.p. 97–98 °C; ^1^H-NMR (CDCl_3_) *δ* (ppm): 4.47 (q, 2H, ^3^*J* = 7.9 Hz), 6.96 (d, 1H, ^3^*J* = 8.4 Hz), 7.17–7.21 (m, 1H), 7.27–7.30 (m, 2H), 7.43–7.47 (m, 1H), 7.52 (d, 2H, ^3^*J* = 8.4 Hz), 7.58–7.62 (m, 1H), 7.73–7.76 (m, 2H); ^13^C-NMR (CDCl_3_) *δ* (ppm): 66.36 (q, ^2^*J*_C-F_ = 36.1 Hz), 112.90, 123.08, 127.14, 127.67, 129.76, 130.51, 131.20, 131.40, 133.23, 133.52, 137.20, 141.90, 155.72, 191.33; ^19^F-NMR (CDCl_3_) *δ* (ppm): −73.49 (t, 3F, ^3^*J* = 7.7 Hz); IR (KBr) *ν*: 3039, 2870, 2361, 2343, 1647, 1605, 1588, 1451, 1325, 1289, 1266, 1119, 1056, 1024, 969, 865, 767, 667 cm^−1^; HRMS (ESI) calc. for C_17_H_12_BrF_3_O_2_: [M + H]^+^ 385.0051 and [M + Na]^+^ 406.9871; Found: 385.0057 and 406.9874.

*(E)-1-(4-(2-Fluoroethoxy)phenyl)-3-(4-methylphenyl)-prop-2-en-1-one* (**4a**) [44]. Yellow solid, Yield: 78%, m.p. 110.1–110.7 °C; ^1^H-NMR (CDCl_3_) *δ* (ppm): 2.39 (s, 3H), 4.30 (dt, 2H, ^3^*J*_H-H_ = 3.8 Hz, ^3^*J*_H-F_ = 28.1 Hz), 4.80 (dt, 2H, ^3^*J*_H-H_ = 4.1 Hz, ^2^*J*_H-F_ = 47.2 Hz), 7.00–7.02 (m, 2H), 7.22–7.24 (m, 2H), 7.48–7.56 (m, 3H), 7.77–7.81(m,1H), 8.04(d, 2H, J = 8.4 Hz).

*(E)-1-(4-(2-Fluoroethoxy)phenyl)-3-(3-nitrophenyl)prop-2-en-1-one* (**4e**) [44]. Pale yellow solid, Yield: 66%, m.p. 116–118 °C (lit. 115–117 °C); ^1^H-NMR (CDCl_3_) *δ* (ppm): 4.32 (dt, 2H, ^3^*J*_H-H_ = 4.0 Hz, ^3^*J*_H-F_ = 27.1 Hz,), 4.81 (dt, 2H, ^3^*J*_H-H_ = 3.9 Hz,^2^*J*_H-F_ = 47.1 Hz,), 7.04 (m, 2H), 7.60–7.69 (m, 2H), 7.81–7.85 (m, 1H), 7.91 (d, 1H, ^3^*J* = 7.7 Hz), 8.08 (d, 2H, ^3^*J* = 8.7 Hz), 8.25 (d, 1H, ^3^*J* = 8.0 Hz), 8.52 (s, 1H).

*(E)-3-(2,5-Dimethoxyphenyl)-1-(4-(2-fluoroethoxy)phenyl)prop-2-en-1-one* (**4f**)**.** Yellow solid, Yield: 80%, m.p. 76–77 °C (lit. 75–77 °C); ^1^H-NMR (CDCl_3_) *δ* (ppm): 3.82 (s, 3H), 3.87 (s, 3H), 4.30 (dt, 2H, ^3^*J*_H-H_ = 4.9 Hz, ^3^*J*_H-F_ = 25.6 Hz), 4.80 (dt, 2H, ^3^*J*_H-H_ = 4.7 Hz, ^2^*J*_H-F_ = 46.4 Hz), 6.87–6.89 (m, 1H), 6.92–6.95 (m, 1H), 7.01 (d, 2H, ^3^*J* = 8.8 Hz), 7.16–7.17 (m, 1H), 7.57–7.61 (m, 1H), 8.02–8.09 (m, 3H); ^13^C-NMR (CDCl_3_) *δ* (ppm): 55.99, 56.26, 67.31 (d, ^2^*J*_C-F_ = 20.7 Hz), 81.82 (d, ^1^*J*_C-F_ = 170.9 Hz), 112.57, 113.92, 114.45, 117.13, 123.03, 124.78, 131.01, 131.43, 131.97, 139.67, 153.42, 153.62, 162.15, 189.46; ^19^F-NMR (CDCl_3_) *δ* (ppm): −223.52 to −223.92 (m, 1F); IR (KBr) *ν*: 2946, 2838, 1656, 1607, 1598, 1572, 1496, 1453, 1427, 1341, 1318, 1281, 1249, 1185, 1116, 1073, 1052, 974, 950 cm^−1^; HRMS (ESI) calcd. for C_19_H_19_FO_4_: [M + H]^+^ 331.1346 and [M + Na]^+^ 353.1165; Found: 331.1359 and 353.1171.

*(E)-1-(4-(2-Fluoroethoxy)phenyl)-3-(furan-2-yl)prop-2-en-1-one* (**4g**) [44]. Brown solid, Yield: 67%, m.p. 75–76 °C; ^1^H-NMR (CDCl_3_) *δ* (ppm): 4.30 (dt, 2H, ^3^*J*_H-H_ = 4.1 Hz, ^3^*J*_H-F_ = 26.9 Hz), 4.79 (dt, 2H, ^3^*J*_H-H_ = 3.5 Hz, ^2^*J*_H-F_ = 48.0 Hz), 6.51 (dd, 1H, ^3^*J* = 3.1 Hz, ^3^*J* = 1.7 Hz), 6.7 (d, 1H, ^3^*J* = 3.9 Hz), 6.99–7.02 (m, 2H), 7.44–7.60 (m, 3H), 8.04–8.06 (m, 2H). 

*(E)-3-(2-Bromophenyl)-1-(4-(2-fluoroethoxy)phenyl)prop-2-en-1-one* (**4i**)**.** Pale yellow solid, Yield: 76%, m.p. 115–116 °C; ^1^H-NMR (CDCl_3_) *δ* (ppm): 4.31 (dt, 2H, ^3^*J*_H-H_ = 3.8 Hz, ^3^*J*_H-F_ = 28.3 Hz), 4.80 (dt, 2H, ^3^*J*_H-H_ = 4.0 Hz, ^2^*J*_H- F_ = 47.5 Hz), 7.02 (d, 2H, ^3^*J* = 8.8 Hz), 7.32–7.45 (m, 3H), 7.64 (d, 1H, ^3^*J* = 8.1 Hz), 7.72–7.74 (m, 1H), 8.03–8.06 (m, 2H), 8.10–8.14 (m, 1H); ^13^C-NMR (CDCl_3_) *δ* (ppm): 67.35 (d, ^2^*J*_C-F_ = 20.6 Hz), 81.77 (d, ^1^J_C-F_ = 172.1 Hz), 114.57, 122.78, 125.03, 125.95, 127.82, 128.00, 131.17, 131.33, 133.68, 135.37, 142.69, 162.42; ^19^F-NMR (CDCl_3_) *δ* (ppm): −223.50 to −223.90 (m, 1F); IR (KBr) *ν*: 3696, 3311, 2360, 1657, 1599, 1575, 1509, 1464, 1368, 1332, 1316, 1273, 1222, 1187, 1092, 1017, 918, 886, 829, 757, 740, 671, 647, 579, 509 cm^−1^; HRMS (ESI) calcd. for C_17_H_14_BrFO_2_: [M + H]^+^ 349.0239; Found: 349.0233.

*(E)-3-(4-Bromophenyl)-1-(3-(2-fluoroethoxy)phenyl)prop-2-en-1-one* (**5d**)**.** Yellow solid, Yield: 69%, m.p. 122–124 °C; ^1^H-NMR (CDCl_3_) *δ* (ppm): 4.30 (dt, 2H, ^3^*J*_H-H_ = 4.0 Hz, ^3^*J*_H-F_ = 27.9 Hz), 4.8 (dt, 2H, ^3^*J*_H-H_ = 4.1 Hz, ^2^*J*_H-F_ = 47.4 Hz), 7.17–7.19 (m,1H), 7.43 (t, 1H, ^3^*J* = 8.0 Hz), 7.48–7.57 (m, 6H), 7.62–7.64 (m, 1H), 7.72–7.76 (m, 1H); ^13^C-NMR (CDCl_3_) *δ* (ppm): 67.45 (d, ^2^*J*_C-F_ = 20.60 Hz), 81.90 (d, ^1^*J*_C-F_ = 171.3 Hz), 113.65, 120.19, 121.75, 122.53, 125.03, 127.64, 129.97, 132.37, 133.86, 139.55, 143.67, 158.90, 189.88; ^19^F-NMR (CDCl_3_) *δ* (ppm): −223.49 to −223.89 (m, 1F); IR (KBr) *ν*: 2968, 1660, 1597, 1577, 1459, 1486, 1443, 1313, 1181, 1113, 1072, 1049, 1007, 975, 945, 893, 876, 834, 819, 793, 770, 691, 678, 612, 491 cm^−1^; HRMS (ESI) calcd. for C_17_H_14_BrFO_2_ : [M + H]^+^ 349.0239 and [M + Na]^+^ 371.0059; Found: 349.0233 and 371.0059.

*(E)-3-(2,5-Dimethoxyphenyl)-1-(3-(2-fluoroethoxy)phenyl)prop-2-en-1-one* (**5f**)**.** Yellow viscous solid, Yield: 75%, ^1^H-NMR (CDCl_3_) *δ* (ppm): 3.82 (s, 3H), 3.87 (s, 3H), 4.30 (dt, 2H, ^3^*J*_H-H_ = 4.2 Hz, ^3^*J*_H-F_ = 27.9 Hz), 4.79 (dt, 2H, ^3^*J*_H-H_ = 4.4 Hz, ^2^*J*_H-F_ = 47.0 Hz), 6.86–6.89 (m, 1H), 6.94–6.97 (m, 1H), 7.12–7.18 (m, 2H), 7.42 (t, 1H, ^3^*J* = 8.0 Hz), 7.54–7.58 (m, 2H), 7.62–7.64 (m, 1H), 8.07–8.10 (m, 1H); ^13^C-NMR (CDCl_3_) *δ* (ppm): 56.01, 56.26, 67.44 (d, ^2^*J*_C-F_ = 20.1 Hz), 81.97 (d, ^1^*J*_C-F_ = 171.20 Hz), 112.62, 113.62, 113.85, 117.51, 119.97, 121.86, 123.15, 124.58, 129.82, 140.04, 140.52, 153.52, 153.66, 158.81, 190.81; ^19^F-NMR (CDCl_3_) *δ* (ppm): −223.46 to −223.86 (m, 1F); IR (KBr) *ν*: 2952, 2836, 2066, 1866, 1660, 1585, 1495, 1438, 1326, 1259, 1180, 1118, 1046, 990, 946, 859, 865, 747, 710, 612, 564 cm^−1^; HRMS (ESI) calcd. for C_19_H_19_FO_4_:[M + H]^+^ 331.1346 and [M + Na]^+^ 353.1165; Found: 331.1338 and 353.1157.

*(E)-1-(3-(2-Fluoroethoxy)phenyl)-3-(4-methoxyphenyl)prop-2-en-1-one* (**5k**)**.** Pale yellow solid, Yield: 90%, m.p. 104–105 °C; ^1^H-NMR (CDCl_3_) *δ* (ppm): 3.86 (s, 3H), 4.30 (dt, 2H, ^3^*J*_H-H_ = 4.1 Hz,^3^*J*_H-F_ = 28.4 Hz,), 4.79 (dt, 2H, ^3^*J*_H-H_ = 3.9 Hz, ^2^*J*_H-F_ = 47.2 Hz), 6.93–6.95(m, 2H), 7.15–7.17 (m, 1H), 7.38–7.44(m, 2H), 7.56–7.64 (m, 4H), 7.77–7.81(m, 1H); ^13^C-NMR (CDCl_3_) *δ* (ppm): 55.57, 67.41 (d, ^2^*J*_C-F_ = 20.2 Hz), 81.97 (d, ^1^*J*_C-F_ = 170.9 Hz), 113.55, 114.57, 119.76, 119.86, 121.67, 127.69, 129.82, 130.44, 140.08, 145.04, 158.83, 161.88, 190.26; ^19^F-NMR (CDCl_3_) *δ* (ppm): −223.51 to −223.91 (m, 1F); IR (KBr) *ν*: 2972, 1657, 1594, 1575, 1511, 1486, 1418, 1326, 1309, 1286, 1254, 1182, 1172, 1112, 1074, 1046, 1030, 976, 890, 875, 830, 793, 739, 680, 536, 516 cm^−1^; HRMS (ESI) calcd. for C_18_H_17_FO_3_ :[M + H]^+^ 301.1240 and [M + Na]^+^ 323.1059; Found: 301.1233 and 323.1050.

*(E)-3-(2-Bromophenyl)-1-(2-(2-fluoroethoxy)phenyl)prop-2-en-1-one* (**6i**)**.** Yellow solid, Yield: 76%, m.p. 86–88 °C; ^1^H-NMR (CDCl_3_) *δ* (ppm): 4.33 (dt, 2H, ^3^*J*_H-H_ = 3.7 Hz, ^3^*J*_H-F_ = 28.6 Hz), 4.76 (dt, 2H, ^3^*J*_H-H_ = 4.0 Hz, ^2^*J*_H-F_ = 47.5 Hz), 6.96–6.98 (m, 1H), 7.08–7.11 (m, 1H), 7.19–7.24 (m, 1H), 7.30–7.34 (m, 1H), 7.46–7.50 (m, 2H), 7.60–7.62 (m, 1H), 7.71–7.78 (m, 2H), 8.01–8.05 (m, 1H); ^13^C-NMR (CDCl_3_) *δ* (ppm): 67.91 (d, ^2^*J*_C-F_ = 19.7 Hz), 81.77 (d, ^1^*J*_C-F_ = 171.40 Hz), 112.60, 121.75, 126.14, 127.82, 127.96, 127.98, 129.43, 131.17, 131.21, 133.43, 133.54, 135.22, 141.21, 157.17, 192.15; ^19^F-NMR (CDCl_3_) *δ* (ppm): −222.76 to 223.16 (m, 1F); IR (KBr) *ν*: 3331, 2893, 1646, 1606, 1484, 1464, 1450, 1329, 1281, 1262, 1166, 1119, 1064, 1042, 1026, 975, 922, 880, 814, 756, 670, 648, 616, 578, 530, 467, 446 cm^−1^; HRMS (ESI) calcd. for C_17_H_14_BrFO_2_: [M + H]^+^ 349.0239 and [M + Na]^+^ 371.0059; Found: 349.0243 and 371.0057.

*(E)-3-(4-(2-Fluoroethoxy)phenyl)-1-phenylprop-2-en-1-one* (**7i**) [44]. Yellow solid, Yield: 76%, m.p. 79–80 °C (lit. 78–80 °C); ^1^H-NMR (CDCl_3_) *δ* (ppm): 4.27 (dt, 2H, ^3^*J*_H-H_ = 4.2 Hz, ^3^*J*_H-F_ = 27.6 Hz), 4.79 (dt, 2H, ^3^*J*_H-H_ = 3.8 Hz, ^2^*J*_H-F_ = 47.6 Hz), 6.96–6.98 (m, 2H), 7.41–7.45 (m, 1H), 7.49–7.52 (m, 2H), 7.56–7.58 (m, 1H), 7.61–7.63 (m, 2H), 7.77–7.81 (m, 1H), 8.00–8.02 (m, 2H).

*(E)-3-(4-(2-Fluoroethoxy)phenyl)-1-(3-nitrophenyl)prop-2-en-1-one* (**7ii**) [44]. Pale yellow solid, Yield: 59%, 122–124 °C (lit. 122–124 °C); ^1^H-NMR (CDCl_3_) *δ* (ppm): 2.44 (s, 3H), 4.27 (dt, 2H, ^3^*J*_H-H_ = 4.1 Hz, ^3^*J*_H-F_ = 27.5 Hz), 4.78 (dt, 2H, ^3^*J*_H-H_ = 4.2 Hz, ^2^*J*_H-F_ = 47.4 Hz), 6.96 (d, 2H, ^3^*J* = 8.9 Hz), 7.30 (d, 2H, ^3^*J* = 8.0 Hz), 7.42–7.45 (m, 1H), 7.60–7.62 (m, 2H), 7.76–7.80 (m, 1H), 7.92–7.94 (m, 2H).

*(E)-1-(2,4-Dichlorophenyl)-3-(4-(2-fluoroethoxy)phenyl)prop-2-en-1-one* (**7iii**)**.** Pale yellow solid, Yield: 67%, m.p. 85–87 °C; ^1^H-NMR (CDCl_3_) *δ* (ppm): 4.26 (dt, 2H, ^3^*J*_H-H_ = 4.2 Hz, ^3^*J*_H-F_ = 27.6 Hz), 4.78 (dt, 2H, ^3^*J*_H-H_ = 4.1 Hz, ^2^*J*_H-F_ = 47.4 Hz), 6.94–6.97 (m, 2H), 7.01 (s, 1H), 7.33–7.36 (m, 1H), 7.39–7.41 (m, 1H), 7.43–7.48 (m, 2H), 7.52–7.54 (m, 2H); ^13^C-NMR (CDCl_3_) *δ* (ppm): 67.27 (d, ^2^*J*_C-F_ = 21.20 Hz), 81.76 (d, ^1^*J*_C-F_ = 171.5 Hz), 115.22, 124.17, 127.39, 127.64, 130.29, 130.47, 130.67, 132.42, 136.84, 137.80, 146.52, 160.98, 192.82; ^19^F-NMR (CDCl_3_) *δ* (ppm)**:** −223.92 to −223.52 (m, 1F); IR (KBr) *ν*: 3072, 2968, 1897, 1731, 1660, 1592, 1572,1516, 1456, 1427, 1374, 1337, 1296, 1253, 1267, 1208, 1180, 1048, 1025, 984, 921, 885, 863, 793, 682, 579, 519 cm^−1^; HRMS (ESI) calcd. for C_17_H_13_Cl_2_FO_2_: [M + H]^+^ 339.0355 and [M + Na]^+^ 361.0174; Found: 339.0342 and 361.0155.

*(E)-3-(4-(2-Fluoroethoxy)phenyl)-1-(2-methoxyphenyl)prop-2-en-1-one* (**7iv**) [44]. Yellow solid, Yield: 73%, m.p. 88–89 °C (lit. 87–89 °C); ^1^H-NMR (CDCl_3_) *δ* (ppm): 3.89 (s, 3H), 4.42 (dt, 2H, ^3^*J*_H-H_ = 3.9 Hz, ^3^*J*_H-F_ = 28.2 Hz), 4.77 (dt, 2H, ^3^*J*_H-H_ = 4.0 Hz, ^2^*J*_H-F_ = 47.6 Hz), 6.93 (d, 2H, ^3^*J* = 8.7 Hz), 6.98–7.05 (m, 2H), 7.22–7.25 (m, 1H), 7.46 (t, 1H, ^3^*J* = 8.0 Hz), 7.52–7.55 (m, 2H), 7.58–7.60 (m, 2H).

*(E)-1-(2-Bromophenyl)-3-(4-(2-fluoroethoxy)phenyl)prop-2-en-1-one* (**7vii**) [44]. Yellow solid, Yield: 71%, m.p. 107–108 °C (lit. 107–109 °C); ^1^H-NMR (CDCl_3_) *δ* (ppm): 4.27 (dt, 2H, ^3^*J*_H-H_ = 4.1 Hz, ^3^*J*_H-F_ = 27.7 Hz), 4.78 (dt, 2H, ^3^*J*_H-H_ = 3.9 Hz, ^2^*J*_H-F_ = 46.4 Hz), 6.97 (d, 2H, ^3^*J* = 7.5 Hz), 7.17 (m, 2H), 7.38–7.42 (m, 1H), 7.60–7.62 (m, 2H), 7.77–7.81 (m, 1H), 8.03–8.07 (m, 2H).

*(E)-3-(3-(2-Fluoroethoxy)phenyl)-1-(4-methylphenyl)-prop-2-en-1-one* (**8ii**) [44]. Yellow solid, Yield: 70%, m.p. 81–82 °C (lit. 80–82 °C); ^1^H-NMR (CDCl_3_) *δ* (ppm): 2.5 (s, 3H), 4.33 (dt, 2H, ^3^*J*_H-H_ = 3.9 Hz, ^3^*J*_H-F_ = 28.1 Hz), 4.85 (dt, 2H, ^3^*J*_H-H_ = 3.7 Hz, ^2^*J*_H-F_ = 47.7 Hz), 7.04–7.06 (m, 1H), 7.32–7.43 (m, 4H), 7.58–7.60 (m, 1H), 7.80–7.84 (m, 1H), 7.99–8.01 (m, 2H).

*(E)-1-(2,4-Dichlorophenyl)-3-(3-(2-fluoroethoxy)phenyl)prop-2-en-1-one* (**8iii**)**.** Pale yellow solid, Yield: 80%, m.p. 101–102 °C; ^1^H-NMR (CDCl_3_) *δ* (ppm): 4.28 (dt, 2H, ^3^*J*_H-H_ = 4.2 Hz, ^3^*J*_H-F_ = 27.6 Hz), 4.76 (dt, 2H, ^3^*J*_H-H_ = 4.4 Hz, ^2^*J*_H-F_ = 47.9 Hz), 6.91–6.93 (m, 1H), 7.01–7.04 (m, 1H), 7.24–7.28 (m, 1H), 7.33–7.48 (m, 4H), 7.58 (d, 1H, ^3^*J* = 7.4 Hz), 7.82–7.86 (m, 1H); ^13^C-NMR (CDCl_3_) *δ* (ppm): 67.87 (d, ^2^*J*_C-F_ = 20.6 Hz), 81.71 (d, ^1^*J*_C-F_ = 171.5 Hz), 112.59, 121.74, 123.91, 126.82, 127.30, 129.85, 130.28, 130.69, 132.40, 132.57, 136.89, 137.76, 142.10, 157.74, 193.12; ^19^F-NMR (CDCl_3_) *δ* (ppm): −223.56 to −223.95 (m, 1F); IR (KBr) *ν*: 2966, 1671, 1600, 1572, 1493, 1450, 1372, 1320, 1280, 1248, 1203, 1130, 1073, 1041, 1016, 987, 928, 887, 824, 762, 745, 575 cm^−1^; HRMS (ESI) calcd. for C_17_H_13_Cl_2_FO_2_: [M + H]^+^ 339.0355 and [M + Na]^+^ 361.0174; Found: 339.0350 and 361.0164.

*(E)-3-(3-(2-Fluoroethoxy)phenyl)-1-(2-methoxyphenyl)prop-2-en-1-one* (**8iv**)**.** Pale yellow paste, Yield: 86%, ^1^H-NMR (CDCl_3_) *δ* (ppm): 3.89 (s, 3H), 4.28 (dt, 2H, ^3^*J*_H-H_ = 4.3 Hz, ^3^*J*_H-F_ = 26.9 Hz), 4.78 (dt, 2H, ^3^*J*_H-H_ = 5.1 Hz, ^2^*J*_H-F_ = 45.7 Hz), 6.90–6.92 (m, 1H), 6.98–7.05 (m, 3H), 7.34 (t, ^3^*J* = 7.8 Hz), 7.43–7.48 (m, 2H), 7.61 (d, 2H, ^3^*J* = 8.0 Hz), 7.94–7.98 (m, 1H); ^13^C-NMR (CDCl_3_) *δ* (ppm): 55.81, 67.78 (d, ^2^*J*_C-F_ = 20.7 Hz), 81.80 (d, ^1^*J*_C-F_ = 171.8 Hz), 111.67, 112.44, 120.75, 121.56, 124.68, 128.02, 129.36, 129.65, 130.46, 131.54, 132.77, 138.77, 157.56, 158.17, 193.78; ^19^F-NMR (CDCl_3_) *δ* (ppm): −223.45 to −223.85 (m, 1F); IR (KBr) *ν*: 2962, 1658, 1604, 1485, 1438, 1403, 1281, 1246, 1179, 1163, 1114, 1055, 1028, 982, 886, 761, 678, 655 cm^−1^; HRMS (ESI) calcd. for C_18_H_17_FO_3_: [M + H]^+^ 301.1240 and [M + Na]^+^ 323.1059; Found: 301.1206 and 323.1029.

*(E)-1-(4-Bromophenyl)-3-(3-(2-fluoroethoxy)phenyl)prop-2-en-1-one* (**8v**)**.** Yellow solid, Yield: 74%, m.p. 122–123 °C; ^1^H-NMR (CDCl_3_) *δ* (ppm): 4.33 (dt, 2H, ^3^*J*_H-H_ = 4.0 Hz, ^3^*J*_H-F_ = 27.9 Hz), 4.85 (dt, 2H, ^3^*J*_H-H_ = 3.9 Hz, ^2^*J*_H-F_ = 47.7 Hz), 7.05–7.08 (m, 1H), 7.32–7.34 (m, 2H), 7.40–7.44 (m, 1H), 7.50–7.54 (m, 1H), 7.71–7.73 (m, 2H),7.82–7.86 (m, 1H), 7.94–7.96 (m, 2H); ^13^C-NMR (CDCl_3_) *δ* (ppm): 67.38 (d, ^2^*J*_C-F_ = 20.8 Hz), 81.97 (d, ^1^*J*_C-F_ = 170.50 Hz), 114.43, 117.08, 121.96, 122.01, 128.13, 130.17, 130.27, 132.10, 136.31, 136.95, 145.21, 158.95, 189.44; ^19^F-NMR (CDCl_3_) *δ* (ppm): −223.19 to −223.59 (m, 1F); IR (KBr) *ν*: 2928, 1659, 1604, 1491, 1310, 1262, 1213, 1178, 1048, 1027, 990, 883, 824, 778, 755, 666, 467 cm^−1^; HRMS (ESI) calcd. for C_17_H_14_BrFO_2_: [M + H]^+^ 349.0239; Found: 349.0232.

*(E)-3-(3-(2-Fluoroethoxy)phenyl)-1-(3-methylphenyl)-prop-2-en-1-one* (**8ix**). Pale yellow solid, Yield: 90%, m.p. 82–84 °C; ^1^H-NMR (CDCl_3_) *δ* (ppm): 2.44 (s, 3H), 4.27 (dt, 2H, ^3^*J*_H-H_ = 27.8 Hz, ^3^*J*_H-F_ = 3.9 Hz), 4.79 (dt, 2H, ^3^*J*_H-H_ = 47.3 Hz, ^2^*J*_H-F_ = 4.0 Hz), 6.98–7.00 (m,1H), 7.20 (s, 1H), 7.28–7.37 (m, 3H), 7.50–7.54 (m, 1H), 7.74–7.78 (m, 1H), 7.93–7.95 (m, 2H); ^13^C-NMR (CDCl_3_) *δ* (ppm): 21.82, 67.39 (d, ^2^*J*_C-F_ = 20.4 Hz), 81.99 (d, ^1^*J*_C-F_ = 171.90 Hz), 114.33, 116.82, 121.86, 122.71, 128.81, 129.50, 130.19, 135.70, 136.69, 143.88, 144.18, 158.95, 190.09; ^19^F-NMR (CDCl_3_) *δ* (ppm): −223.48 to −223.88 (m, 1F); IR (KBr) *ν*: 3055, 2920, 1658, 1600, 1576, 1494, 1439, 1372, 1311, 1205, 1182, 1096, 1078, 1055, 1030, 1016, 994, 884, 848 cm^−1^; HRMS (ESI) calcd. for C_18_H_17_FO_2_: [M + H]^+^ 285.1291 and [M + Na]^+^ 307.1110; Found: 285.1286 and 307.1104

*(E)-3-(2-(2-Fluoroethoxy)phenyl)-1-phenylprop-2-en-1-one* (**9i**). Pale yellow viscous liquid, Yield: 81%, ^1^H-NMR (CDCl_3_) *δ* (ppm): 4.33 (dt, 2H, ^3^*J*_H-H_ = 3.9 Hz, ^3^*J*_H-F_ = 27.5 Hz), 4.85 (dt, 2H, ^3^*J*_H-H_ = 4.2 Hz, ^2^*J*_H-F_ = 47.5 Hz), 6.93–6.95 (m, 1H), 7.02–7.06 (m, 1H), 7.37 (t, 1H, ^3^*J* = 8.3 Hz), 7.48–7.52 (m, 2H), 7.56–7.58 (m, 1H), 7.62–7.64 (m, 1H), 7.76–7.80 (m, 1H), 8.04–8.09 (m, 2H); ^13^C-NMR (CDCl_3_) *δ* (ppm): 67.68 (d, ^2^*J*_C-F_ = 21.2 Hz), 81.87 (d, ^1^*J*_C-F_ = 171.70 Hz), 112.36, 121.65, 123.68, 128.35, 128.71, 130.58, 131.70, 132.79, 133.09, 138.55, 140.49, 157.83, 191.21; ^19^F-NMR (CDCl_3_) *δ* (ppm): −223.86 to −223.46 (m, 1F); IR (KBr) *ν*: 2958, 2360, 1661, 1600, 1573, 1488, 1448, 1334, 1279, 1248, 1215, 1180, 1112, 1052, 1017, 927, 886, 753, 734, 694, 578 cm^−1^; HRMS (ESI) calcd. for C_17_H_15_FO_2_: [M + H]^+^ 271.1134 and [M + Na]^+^ 293.0954 ; Found: 271.1131 and 293.0945.

*(E)-3-(2-(2-Fluoroethoxy)phenyl)-1-(4-methylphenyl)-prop-2-en-1-one* (**9ii**). Yellow viscous liquid, Yield: 83%, m.p. 69–70 °C; ^1^H-NMR (CDCl_3_) *δ* (ppm): 2.43 (s, 3H), 4.33 (dt, 2H, ^3^*J*_H-H_ = 4.2 Hz, ^3^*J*_H-F_ = 27.5 Hz), 4.85 (dt, 2H, ^3^*J*_H-H_ = 4.0 Hz, ^2^*J*_H-F_ = 47.4 Hz), 6.93–6.95 (m, 1H), 7.02–7.06 (m, 1H), 7.29–7.31 (m, 2H), 7.34–7.38 (m, 1H), 7.62–7.64 (m, 1H), 7.77–7.80 (m, 1H), 7.95–7.97 (m, 2H), 8.04–8.08 (m, 1H); ^13^C-NMR (CDCl_3_) *δ* (ppm): 21.81, 67.67 (d, ^2^*J*_C-F_ = 20.6 Hz), 81.88(d, ^1^*J*_C-F_ = 171.90 Hz), 112.34, 121.62, 123.73, 124.59, 128.84, 129.42, 130.55, 131.55, 135.98, 140.02, 143.59, 157.79, 190.69; ^19^F-NMR (CDCl_3_) *δ* (ppm): −223.82 to −223.43 (m, 1F); IR (KBr) *ν*: 2860, 2361, 1659, 1598, 1490, 1449, 1280, 1251, 1181, 1126, 1040, 930, 884, 742, 572, 474 cm^−1^; HRMS (ESI) calcd. for C_18_H_17_FO_2_: [M + H]^+^ 285.1291 and [M + Na]^+^ 307.1110 ; Found: 285.1288 and 307.1105.

*(E)-1-(2,4-Dichlorophenyl)-3-(2-(2-fluoroethoxy)phenyl)prop-2-en-1-one* (**9iii**). Yellow solid, Yield: 89%, m.p. 93–94 °C; ^1^H-NMR (CDCl_3_) *δ* (ppm): 4.28 (dt, 2H, ^3^*J*_H-H_ = 4.3 Hz, ^3^*J*_H-F_ = 27.7 Hz), 4.77 (dt, 2H, ^3^*J*_H-H_ = 4.2 Hz, ^2^*J*_H-F_ = 47.0 Hz), 6.92 (m, 1H), 7.01–7.04 (m, 1H), 7.24 (s,1H), 7.28–7.48 (m, 4H), 7.57–7.59 (m, 1H), 7.84 (d, 1H, ^3^*J* = 16.5 Hz); ^13^C-NMR (CDCl_3_) *δ* (ppm): 67.86 (d, ^2^*J*_C-F_ = 20.6 Hz), 81.71 (d, ^1^*J*_C-F_ = 171.80 Hz), 112.58, 112.74,123.90, 126.82, 127.30, 129.86, 130.28, 130.69, 132.40, 132.57, 136.90, 137.76, 142.11, 157.74, 193.13; ^19^F-NMR (CDCl_3_) *δ* (ppm): −223.85 to −223.46 (m, 1F); IR (KBr) *ν*: 2966, 1671, 1600, 1584, 1572, 1493, 1450, 1371, 1334, 1320, 1280, 1247, 1202, 1167, 1130, 1111, 1072, 1042, 1016, 987, 928, 886, 824, 790, 762, 745, 609, 575, 520, 454 cm^−1^; HRMS (ESI) calcd. for C_17_H_13_Cl_2_FO_2_ : [M + H]^+^ 339.0355; Found: 339.0344.

*(E)-1-(4-Bromophenyl)-3-(2-(2-fluoroethoxy)phenyl)prop-2-en-1-one* (**9v**). Pale yellow solid, Yield: 78%, m.p. 84–86 °C; ^1^H-NMR (CDCl_3_) *δ* (ppm): 4.33 (dt, 2H, ^3^*J*_H-H_ = 6.2 Hz, ^3^*J*_H-F_ = 23.0 Hz), 4.86 (dt, 2H, ^3^*J*_H-H_ = 3.7 Hz, ^2^*J*_H-F_ = 48.5 Hz), 6.93–6.95 (m, 1H), 7.02–7.06 (m, 1H), 7.36–7.40 (m, 1H), 7.57–7.65 (m, 3H), 7.74–7.78 (m, 1H), 7.83–7.85 (m, 1H), 7.90–7.93 (m, 1H), 8.03–8.07 (m, 1H); ^13^C-NMR (CDCl_3_) *δ* (ppm): 67.60 (d, ^2^*J*_C-F_ = 20.7 Hz), 81.86 (d, ^1^*J*_C-F_ = 171.30 Hz), 112.34, 121.67, 123.16, 124.26, 129.88, 130.24, 130.95, 131.89, 131.99, 137.27, 141.12, 157.91, 190.08; ^19^F-NMR (CDCl_3_) *δ* (ppm): −223.51 to −223.91 (m, 1F); IR (KBr) *ν*: 2960, 1919, 1684, 1657, 1600, 1571, 1490, 1448, 1394, 1333, 1274, 1252, 1205, 1177, 1128, 1064, 1008, 983, 930, 884, 822, 740, 667, 608, 580, 540, 468 cm^−1^; HRMS (ESI) calcd. for C_17_H_14_BrFO_2_: [M + H]^+^ 349.0239 and [M + Na]^+^ 371.0053; Found: 349.0230 and 371.0044.

*(E)-3-(2-(2-Fluoroethoxy)phenyl)-1-(4-fluorophenyl)prop-2-en-1-one* (**9vii**). Yellow solid, Yield: 71%, m.p. 44–45 °C; ^1^H-NMR (CDCl_3_) *δ* (ppm): 4.33 (dt, 2H, ^3^*J*_H-H_ = 4.3 Hz, ^3^*J*_H-F_ = 27.6 Hz), 4.86 (dt, 2H, ^3^*J*_H-H_ = 3.9 Hz, ^2^*J*_H-F_ = 47.5 Hz), 6.93–6.95 (m, 1H), 7.02–7.06 (m, 1H), 7.10–7.19 (m, 2H), 7.36–7.40 (m, 1H), 7.60–7.62 (m, 1H), 7.76–7.80 (m, 1H), 8.03–8.10 (m, 2H); ^13^C-NMR (CDCl_3_) *δ* (ppm): 67.66 (d, ^2^*J*_C-F_ = 20.6 Hz), 81.87 (d, ^1^*J*_C-F_ = 172.4), 112.35, 115.69, 115.90, 121.67, 123.34, 130.88, 130.96, 131.05, 131.24, 131.33, 131.79, 140.79, 189.77; ^19^F-NMR (CDCl_3_) *δ* (ppm): −105.71 to −105.78 (m, 1H) and −223.46 to −223.86 (m, 1H); HRMS (ESI) calcd. for C_17_H_14_F_2_O_2_: [M + H]^+^ 289.1040 and [M + Na]^+^ 311.0860; Found: 289.1025 and 311.0843.

*(E)-3-(2-(2-Fluoroethoxy)phenyl)-1-(2-methylphenyl)-prop-2-en-1-one* (**9viii**). Pale yellow viscous liquid, Yield: 85%, ^1^H-NMR (CDCl_3_) *δ* (ppm): 2.46 (s, 3H), 4.27 (dt, 2H, ^3^*J*_H-H_ = 4.0 Hz, ^3^*J*_H-F_ = 27.1 Hz), 4.75 (dt, 2H, ^3^*J*_H-H_ = 4.1 Hz, ^2^*J*_H-F_ = 48.1 Hz), 6.90–6.92 (m, 1H), 7.00–7.04 (m, 1H), 7.25–7.29 (m, 3H), 7.34–7.39 (m, 2H), 7.52–7.54 (m, 1H), 7.58–7.60 (m, 1H), 7.83 (d, 1H, ^3^*J* =16.4 Hz); ^13^C-NMR (CDCl_3_) *δ* (ppm): 20.49, 67.79 (d, ^2^*J*_C-F_ = 20.40 Hz), 81.75 (d, ^1^*J*_C-F_ = 172.6 Hz), 112.48, 121.67, 124.27, 125.54, 127.70, 128.43, 129.53, 130.52, 131.39, 131.92, 137.16, 139.34, 141.36, 15s7.54, 197.15; ^19^F-NMR (CDCl_3_) *δ* (ppm): −223.53 to −223.93 (m, 1F); IR (KBr) *ν*: 3707, 3314, 2869, 1636, 1600, 1489, 1459, 1390, 1310, 1276, 1245, 1168, 1112, 1064, 994, 922, 882, 748, 607, 458 cm^−1^; HRMS (ESI) calcd. for C_18_H_17_FO_2_: [M + H]^+^ 285.1291 and [M + Na]^+^ 307.1110; Found: 285.1286 and 307.1102.

## 4. Conclusions

In summary, we have reported the synthesis of a series of 2,2,2-trifluoroethoxy- and 2-fluoroethoxychalcones and their in vitro antiplasmodial evaluation against *P. falciparum* (3D7). It was found for the first time that 2,2,2-trifluoroethoxychalcones exhibited position-dependent antiplasmodial activity and the compounds **3a** and **3f** exhibited good activity with negligible toxic effects on normal erythrocytes and mammalian cells. The influence of the number of fluorine atoms on antiplasmodial activity was also demonstrated with 2-fluoroethoxychalcones. The results showed that chalcones with 2,2,2-trifluoroethoxy groups on the *o*-position of the 1-phenyl ring and 2-fluoroethoxy chalcones could be considered as viable alternatives to current drugs of future interest. Based on this result, a detailed study on the antiplasmodial activity of 2,2,2-trifluoroethoxychalcones in which the 2,2,2-trifluoroethoxy group is substituted on the 3-phenyl ring and 2-fluoroethoxychalcones will be undertaken.

## Figures and Tables

**Table 1:** In vitro antiplasmodial activity of 2,2,2-trifluoroethoxychalcones against *P. falciparum* (3D7).

No.	Chalcone	Yield (%) ^a^	*P. falciparum* (3D7)	
			IC_50_ (µg/mL) ^b^	IC_50_ (µM)
1	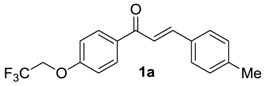	87	>50	>156.1
2	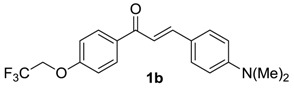	77	>50	>143.1
3	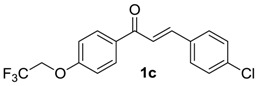	90	41	120.3
4	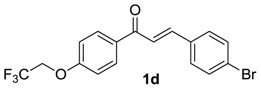	77	43	111.6
5	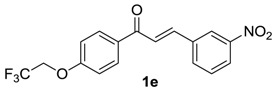	94	43	122.4
6	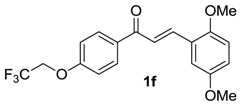	78	37	101.0
7	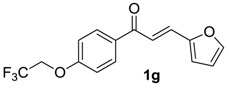	61	>50	>168.8
8	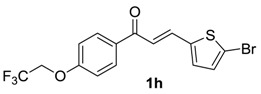	93	38	97.1
9	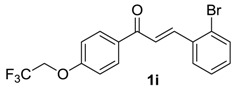	81	32	83.1
10	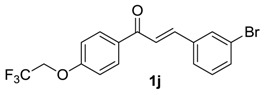	74	>50	>129.8
11	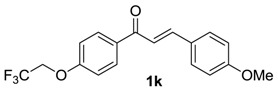	72	40	118.9
12	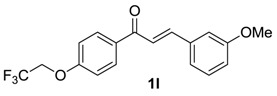	91	36	107.0
13	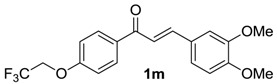	70	10	27.3
14	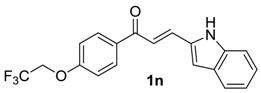	40	>50	>144.8
15	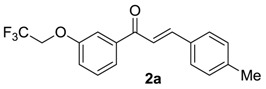	80	>50	>156.1
16	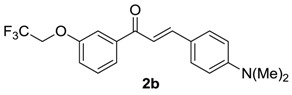	71	>50	>143.1
17	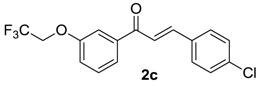	73	49	143.8
18	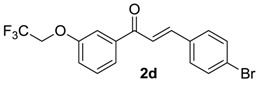	75	23	59.7
19	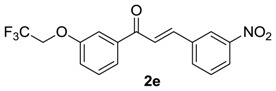	73	22	62.6
20	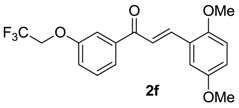	77	11	30.0
21	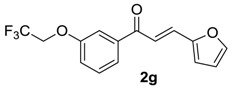	65	6	20.3
22	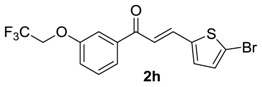	85	>50	>127.8
23	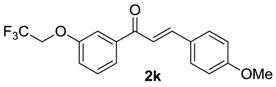	73	48	142.7
24	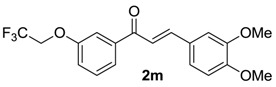	64	46	125.6
25	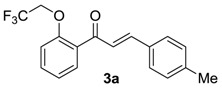	82	3	9.4
26	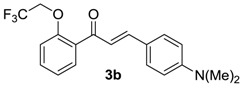	72	12.5	35.8
27	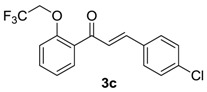	73	9	26.4
28	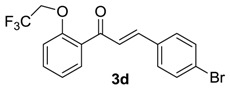	73	10	26.0
29	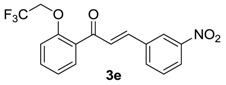	75	46	130.9
30	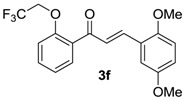	62	2.2	6.0
31	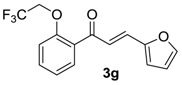	59	14	47.3
32	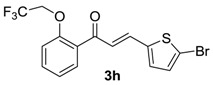	87	30	76.7
33	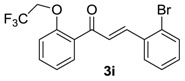	74	7	18.2
34	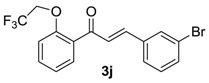	74	19	49.3
	Chloroquine (CQ) ^c^		0.010	0.031
	Artemisinin (ART) ^c^		0.021	0.074

^a^ Isolated yield; ^b^ IC_50_ (µg/mL): concentration corresponding to 50% growth inhibition of parasite; ^c^ Antimalarial control.

**Table 2:** Effect on antiplasmodial activity of chalcones **3a** and **3f** with artemisinin against *P. falciparum* (3D7) at two different concentrations.

Drug Combination	*P. falciparum* 3D7 (CQ Sensitive) IC_50_ Values µg/mL
**3a** + Artemisinin 5 ng/mL	1.04
**3a** + Artemisinin 10 ng/mL	0.6
**3f** + Artemisinin 5 ng/mL	2.15
**3f** + Artemisinin 10 ng/mL	0.89

**Table 3:** In vitro antiplasmodial activity of 2-fluoroethoxychalcones against *P. falciparum* (3D7).

No.	Entry	Yield (%) ^a^	*P. falciparum* (3D7)	
			IC_50_ (µg/mL) ^b^	IC_50_ (µM)
1	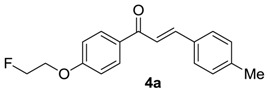	78	20	70.3
2	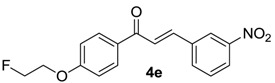	66	13	41.2
3	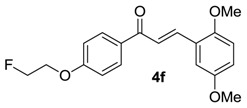	80	8	24.2
4	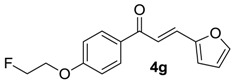	67	20	76.8
5	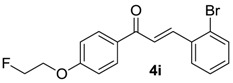	76	^c^	^c^
6	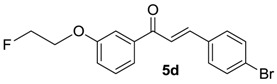	69	12	34.4
7	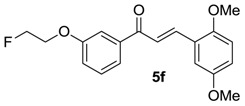	75	^c^	^c^
8	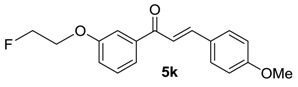	90	12	39.9
9	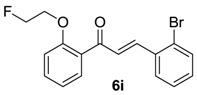	76	6.5	18.6
10	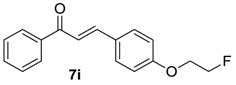	76	25	92.5
11	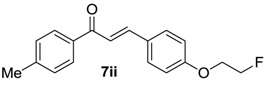	59	10	35.2
12	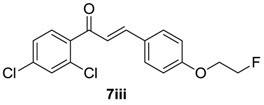	67	8	23.6
13	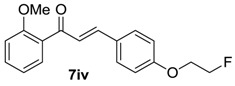	73	5	16.6
14	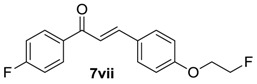	71	10	28.6
15	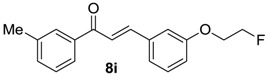	90	7	25.9
16	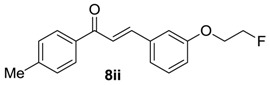	70	9	31.6
17	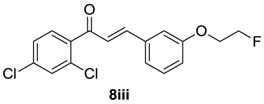	80	4.5	13.3
18	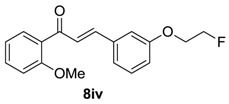	86	^c^	^c^
19	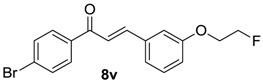	74	12.5	35.8
20	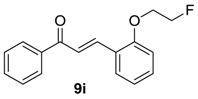	81	^c^	^c^
21	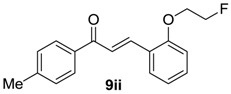	83	3.5	12.3
22	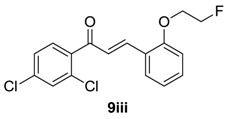	89	6	17.7
23	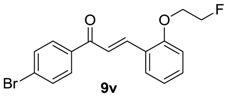	78	10	28.6
24	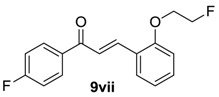	71	^c^	^c^
25	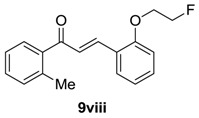	85	5	17.6
	Chloroquine (CQ)^d^		0.010	0.031
	Artemisinin (ART)^d^		0.021	0.074

^a^ Isolated yield; ^b^ IC_50_ (µg/mL): concentration corresponding to 50% growth inhibition of parasite; ^c^ Not tested; ^d^ Antimalarial control.

**Table 4:** Cytotoxic effect on Vero cells and selectivity index of most active molecules (**3a**, **3f**, **8iii**, and **9ii**) in vitro.

Chalcone	Cytotoxicity (Vero Cell)		Selectivity Index (SI)
	µg/mL	µM	
**3a**	26	81.2	8.6
**3f**	18	49.1	8.2
**8iii**	15	44.2	3.3
**9ii**	24	84.4	6.9

## Supplementary Materials

Supplementary materials are available online.

## References

[1] Gomes M.N., Muratov E.N., Pereira M., Peixoto J.C., Rosseto L.P., Cravo P.V.L., Andrade C.H., Neves B.J. (2017). Chalcone derivatives: Promising starting points for drug design. Molecules.

[2] Liu M., Wilairat P., Go M.L. (2001). Antimalarial alkoxylated and hydroxylated chalones: Structure-activity relationship analysis. J. Med. Chem..

[3] Singh N., Pandey S.K., Tripathi R.P. (2010). Regioselective [3+2] cycloaddition of chalcones with a sugar azide: Easy access to 1-(5-deoxy-d-xylofuranos-5-yl)-4,5-disubstituted-1*H*-1,2,3-triazoles. Carbohydr. Res..

[4] Rajesh Kumar P.C., Ravindrachary V., Janardhana K., Manjunath H.R., Karegouda P., Crasta V., Sridhar M.A. (2011). Optical and structural properties of chalcone NLO single crystals. J. Mol. Struct..

[5] Almeida L.R., Anjos M.M., Ribeiro G.C., Valverde C., Machado D.F.S., Oliveira G.R., Napolitano H.B., de Oliveira H.C.B. (2017). Synthesis, structural characterization and computational study of a novel amino chalcone: A potential nonlinear optical material. New J. Chem..

[6] Indira J., Karat P.P., Sarojini B. (2002). Growth, characterization and nonlinear optical property of chalcone derivative. J. Cryst. Growth.

[7] Zhang X.W., Zhao D.H., Quan Y.C., Sun L.P., Yin X.M., Guan L.P. (2010). Synthesis and evaluation of antiinflammatory activity of substituted chalcone derivatives. Med. Chem. Res..

[8] Shen-Jew W., Cheng-Tsung L., Lo-Ti T., Jing-Ru W., Horng-Huey K., Jih-Pyang W., Chun-Nan L. (2005). Synthetic chalcones as potential anti-inflammatory and cancer chemopreventive agents. Eur. J. Med. Chem..

[9] Sashidhara K.V., Rao K.B., Kushwaha P., Modukuri R.K., Singh P., Soni I., Shukla P.K., Chopra S., Pasupuleti M. (2015). Novel Chalcone-Thiazole Hybrids as Potent Inhibitors of Drug Resistant Staphylococcus aureus. ACS Med. Chem. Lett..

[10] Konduru N.K., Dey S., Sajid M., Owais M., Ahmed N. (2013). Synthesis and antibacterial and antifungal evaluation of some chalcone based sulfones and bisulfones. Eur. J. Med. Chem..

[11] Nielsen S.F., Christensen S.B., Cruciani G., Kharazmi A., Liljefors T. (1998). Antileishmanial Chalcones: Statistical Design, Synthesis, and Three-Dimensional Quantitative Structure−Activity Relationship Analysis. J. Med. Chem..

[12] Dimić D., Mercader A.G., Castro E.A. (2015). Chalcone derivative cytotoxicity activity against MCF-7 human breast cancer cell QSAR study. Chemom. Intell. Lab. Syst..

[13] Karthikeyan C., Narayana Moorthy N.S., Ramasamy S., Vanam U., Manivannan E., Karunagaran D., Trivedi P. (2014). Advances in Chalcones with Anticancer Activities. Recent Pat. Anticancer Drug Discov..

[14] Dominguez J.N., Charris J.E., Lobo G., Gamboa De Dominguez N., Moreno M.M., Riggione F., Sanchez E., Olson J., Rosenthal P.J. (2001). Synthesis of quinolinyl chalcones and evaluation of their antimalarial activity. Eur. J. Med. Chem..

[15] Bhattacharya A., Mishra L.C., Sharma M., Awasthi S.K., Bhasin V.K. (2009). Antimalarial pharmacodynamics of chalcone derivatives in combination with artemisinin against *Plasmodium falciparum* in vitro. Eur. J. Med. Chem..

[16] Sharma N., Mohanakrishnan D., Sharma U.K., Kumar R., Richa, Sinha A.K., Sahal D. (2014). Design, economical synthesis and antiplasmodial evaluation of vanillin derived allylated chalcones and their marked synergism with artemisinin against chloroquine resistant strains of *Plasmodium falciparum*. Eur. J. Med. Chem..

[17] Ono M., Haratake M., Mori H., Nakayama M. (2007). Novel chalcones as probes for in vivo imaging of β-amyloid plaques in Alzheimer’s brains. Bioorg. Med. Chem..

[18] Cui M., Ono M., Kimura H., Liu B., Saji H. (2011). Synthesis and structure-affinity relationships ofnovel dibenzylideneacetone derivatives as probes for β-Amyloid Plaques. J. Med. Chem..

[19] Chen M., Theander T.G., Christensen S.B., Hviid L., Zhai L., Kharazmi A. (1994). Licochalcone A, a new antimalarial agent, inhibits in vitro growth of the human malaria parasite *Plasmodium falciparum* and protects mice from P. yoelii infection. Antimicrob. Agents Chemother..

[20] Batagin-Neto A., Lavarda F.C. (2014). The correlation between electronic structure and antimalarial activity of alkoxylated and hydroxylated chalcones. Med. Chem. Res..

[21] Valla A., Valla B., Cartier D., Le Guillou R., Labia R., Florent L., Charneau S., Schrevel J., Potier P. (2006). New syntheses and potential antimalarial activities ofnew “retinoid-like chalcones”. Eur. J. Med. Chem..

[22] Mishra L.C., Bhattacharya A., Bhasin V.K. (2009). Phytochemical licochalcone A enhances antimalarial activity of artemisinin in vitro. Acta Trop..

[23] Gil A., Pabón A., Galiano S., Burguete A., Pérez-Silanes S., Deharo E., Monge A., Aldana I. (2014). Synthesis, biological evaluation and structure-activity relationships ofnew quinoxaline derivatives as anti-*Plasmodium falciparum* agents. Molecules.

[24] Hu Y.Q., Gao C., Zhang S., Xu L., Xu Z., Feng L.S., Wu X., Zhao F. (2017). Quinoline hybrids and their antiplasmodial and antimalarial activities. Eur. J. Med. Chem..

[25] Raj R., Saini A., Gut J., Rosenthal P.J., Kumar V. (2015). Synthesis and in vitro antiplasmodial evaluation of 7-chloroquinoline-chalcone and 7-chloroquinoline-ferrocenylchalcone conjugates. Eur. J. Med. Chem..

[26] Snow R.W., Guerra C.A., Noor A.M., Myint H.Y., Hay S.I. (2005). The global distribution of clinical episodes of *Plasmodium falciparum* malaria. Nature.

[27] Mital A. (2007). Recent advances in antimalarial compounds and their patents. Curr. Med. Chem..

[28] Shah F., Mukherjee P., Gut J., Legac J., Rosenthal P.J., Tekwani B.L., Avery M.A. (2011). Identification ofnovel malarial cysteine protease inhibitors using structure-based virtual screening of a focused cysteine protease inhibitor library. J. Chem. Inf. Model..

[29] Wang J., Sánchez-Roselló M., Aceña J.L., Del Pozo C., Sorochinsky A.E., Fustero S., Soloshonok V.A., Liu H. (2014). Fluorine in pharmaceutical industry: Fluorine-containing drugs introduced to the market in the last decade (2001–2011). Chem. Rev..

[30] Purser S., Moore P.R., Swallow S., Gouverneur V. (2008). Fluorine in medicinal chemistry. Chem. Soc. Rev..

[31] O’Neill P.M., Harrison A.C., Storr R.C., Hawley S.R., Ward S.A., Park B.K. (1994). The effect of fluorine substitution on the metabolism and antimalarial activity of Amodiaquine. J. Med. Chem..

[32] Jeschke P. (2004). The unique role of fluorine in the design of active ingredients for modern crop protection. ChemBioChem.

[33] Lee K.S., Lee J.S. (2006). Synthesis of highly fluorinated poly(arylene ether sulfide) for polymeric optical waveguides. Chem. Mater..

[34] Park B.K., Kitteringham N.R., O’Neill P.M. (2001). Metabolism of Fluorine-Containing Drugs. Annu. Rev. Pharmacol. Toxicol..

[35] Bégué J.-P., Bonnet-Delpon D. (2008). Effects of Fluorine Substitution on Biological Properties. Bioorganic and Medicinal Chemistry of Fluorine.

[36] Molinaro C., Gauvreau D., Hughes G., Lau S., Lauzon S., Angelaud R., O’Shea P.D., Janey J., Palucki M., Hoerrner S.R. (2009). Remote electronic control in the regioselective reduction of succinimides: A practical, scalable synthesis of EP4 antagonist MF-310. J. Org. Chem..

[37] Tressaud A., Haufe G. (2008). Fluorine and Health: Molecular Imaging, Biomedical Materials and Pharmaceuticals.

[38] Jadhav D.H., Ramaa C.S. (2007). Synthesis and anti-inflammatory activity of fluorinated chalcone derivatives. Indian J. Chem..

[39] Rojas J., Payá M., Dominguez J.N., Luisa Ferrándiz M. (2002). The synthesis and effect of fluorinated chalcone derivatives on nitric oxide production. Bioorg. Med. Chem. Lett..

[40] Burmaoglu S., Algul O., Anil D.A., Gobek A., Duran G.G., Ersan R.H., Duran N. (2016). Synthesis and anti-proliferative activity of fluoro-substituted chalcones. Bioorg. Med. Chem. Lett..

[41] Mathew B., Ucar G., Yabanogclu-Ciftci S., Baysal I., Suresh J., Elizabeth Mathew G., Kunjumon Vilapurathu J.M., Nadeena A., Nabeela P., Lakshmi V. (2018). Development of Fluorinated Thienylchalcones as Monoamine Oxidase-B Inhibitors: Design, Synthesis, Biological Evaluation and Molecular Docking Studies. Lett. Org. Chem..

[42] Mathew B., Mathew G.E., Uçar G., Baysal I., Suresh J., Vilapurathu J.K., Prakasan A., Suresh J.K., Thomas A. (2015). Development of fluorinated methoxylated chalcones as selective monoamine oxidase-B inhibitors: Synthesis, biochemistry and molecular docking studies. Bioorg. Chem..

[43] Rangarajan T.M., Devi K., Ayushee, Prasad A.K., Pal Singh R. (2015). A general, mild and efficient palladium-catalyzed 2,2,2-trifluoroethoxylation of activated aryl bromides and bromo-chalcones: Bromo-chalcones a new coupling partner in cross-coupling reaction. Tetrahedron.

[44] Rangarajan T.M., Devi K., Verma A.K., Singh R.P., Singh R.P. (2016). A general and efficient Pd-catalyzed rapid 2-fluoroethoxylation of bromo-chalcones. J. Fluorine Chem..

[45] Reeta, Rangarajan T.M., Ayushee, Singh R.P., Singh R.P. (2016). Palladium-catalysed rapid methoxylation and deuteriomethoxylation of bromo-chalcones: Uncovering the catalytic activity of the Pd/tBuXPhos catalyst system. Chem. Select.

[46] Rangarajan T.M., Singh R., Brahma R., Devi K., Singh R.P., Singh R.P., Prasad A.K. (2014). BrettPhos ligand supported palladium-catalyzed C–O bond formation through an electronic pathway of reductive elimination: Fluoroalkoxylation of activated aryl halides. Chem. Eur. J..

[47] Rangarajan T.M., Singh R., Brahma R., Devi K., Singh R.P. (2015). A process for the preparation of arylalkyl ether derivatives. Indian Patent Application.

[48] Seebach D. (2014). Joy and flustration with organofluorine compounds—A fluorous autobiography. Chimia (Aarau).

[49] Wang R., Wang L., Zhang K., Li J., Zou D., Wu Y., Wu Y. (2015). Facile synthesis of trifluoroethyl aryl ethers through copper-catalyzed coupling of CF_3_CH_2_OH with aryl- and heteroaryl boronic acids. Tetrahedron Lett..

[50] Mangawa S.K., Sharma C., Kumar Singh A., Awasthi S.K. (2015). Expedient and efficient one pot synthesis of trifluoroethyl ethers from metal free 2,4,6-tris-(2,2,2-trifluoro-ethoxy)-[1,3,5] triazene. RSC Adv..

[51] Yin J., Zarkowsky D.S., Thomas D.W., Zhao M.M., Huffman M.A. (2004). Direct and Convenient Conversion of Alcohols to Fluorides. Org. Lett..

