# Supplementary material for: Synthesis and Evaluation of Antiplasmodial Activity of 2,2,2-Trifluoroethoxychalcones and 2-Fluoroethoxy Chalcones against Plasmodium falciparum in Culture
Source: Molecules. 2018 May 14;23(5):1174. doi: 10.3390/molecules23051174 (PMC6099641; doi:10.3390/molecules23051174)
Supplement: Supplementary file 1 [file molecules-23-01174-s001.pdf]

Supporting information

Synthesis and Evaluation of Antiplasmodial Activity of 2,2,2-Trifluoroethoxychalcones and 2-Fluoroethoxy chalcones against *Plasmodium falciparum* in Culture

Kavita Devi [1], Vinoth Rajendran [2], Ayushee [3], T. M. Rangarajan [4,*], Rishi Pal Singh [4,*] Prahlad C. Ghosh [2], and Manjula Singh [5]

[1] Fluoroorganic Laboratory, Center for Fire, Explosive and Environment Safety, Delhi-110 054, India; kavik188@gmail.com

[2] Department of Biochemistry, University of Delhi South Campus, Benito Juarez Road, New Delhi-110 021, India; vinoth.avj@gmail.com (V.R.); pcghose@gmail.com (P.C.G.)

[3] Department of Chemistry, Hansraj College, University of Delhi, Delhi-110 007, India; ayusheesingh27@yahoo.in

[4] Department of Chemistry, Sri Venkateswara College, University of Delhi, New Delhi-110 021, India; rangarajan93150@gmail.com (T.M.R.); rpsingh54@gmail.com (R.P.S.)

[5] Department of Chemistry, Shivaji College, University of Delhi, New Delhi-110 027; manjulasingh56@gmail.com

* Correspondence: rangarajan93150@gmail.com (T.M.R.), rpsingh54@gmail.com (R.P.S.); Tel.:91-882-542-6596

^{1}H, ^{13}C and ^{19}F NMR Spectra of Isolated Compounds

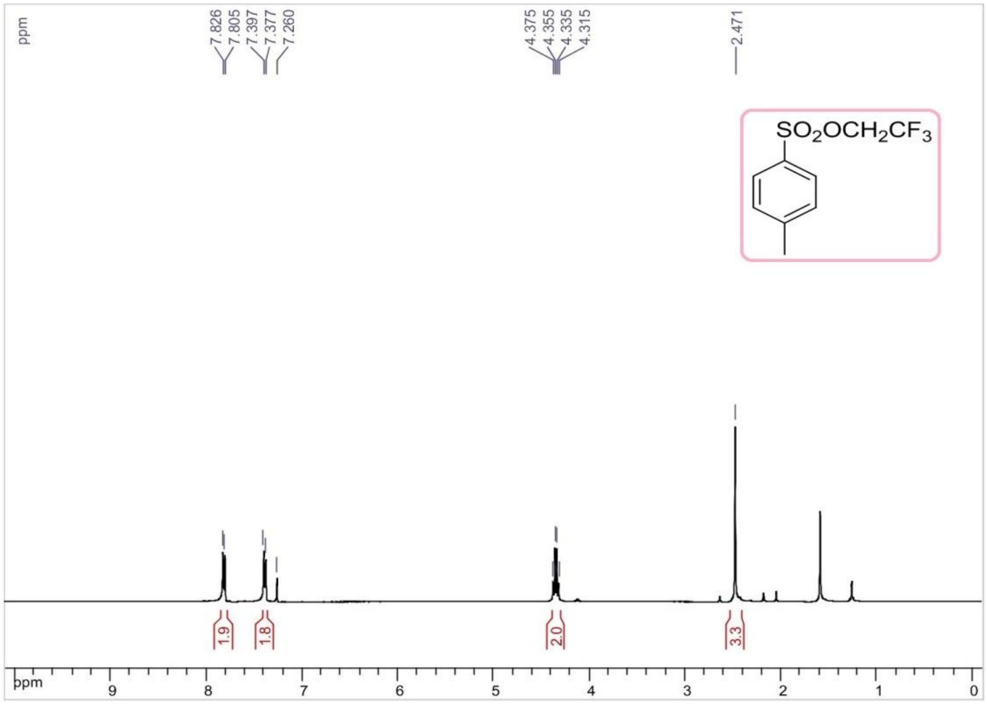
ppm
7.826
7.805
7.397
7.377
7.260
4.375
4.355
4.335
4.315
2.471
SO2OCH2CF3
1.9
1.8
2.0
3.3
ppm
9
8
7
6
5
4
3
2
1
0

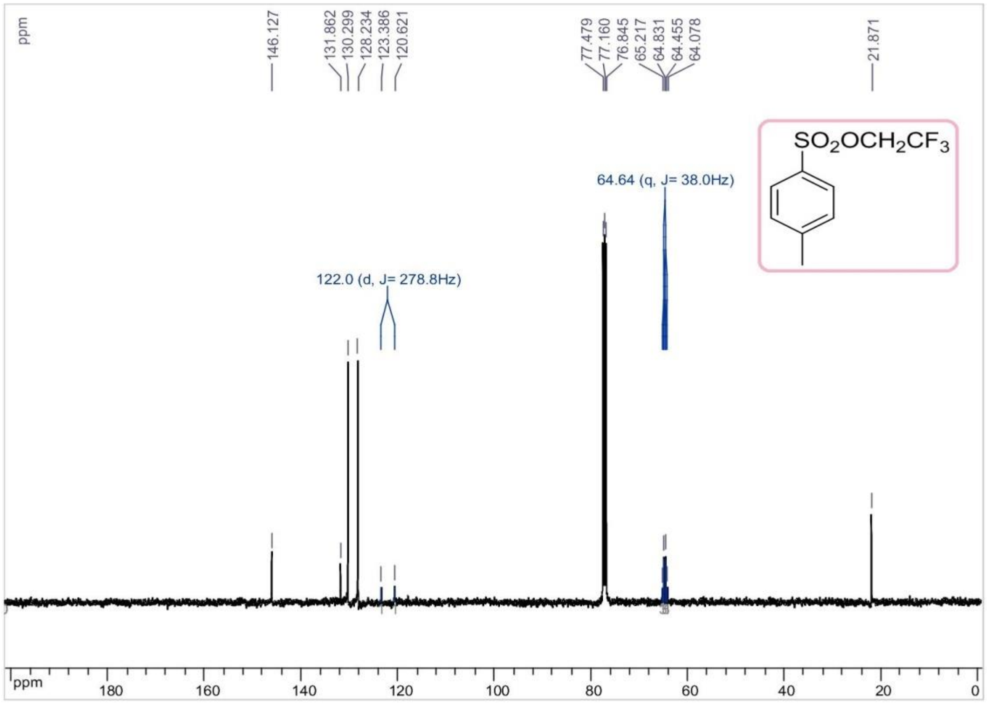
ppm
146.127
131.862
130.299
128.234
123.386
120.621
77.479
77.160
76.845
65.217
64.831
64.455
64.078
21.871
SO2OCH2CF3
64.64 (q, J= 38.0Hz)
122.0 (d, J= 278.8Hz)
ppm
180
160
140
120
100
80
60
40
20
0

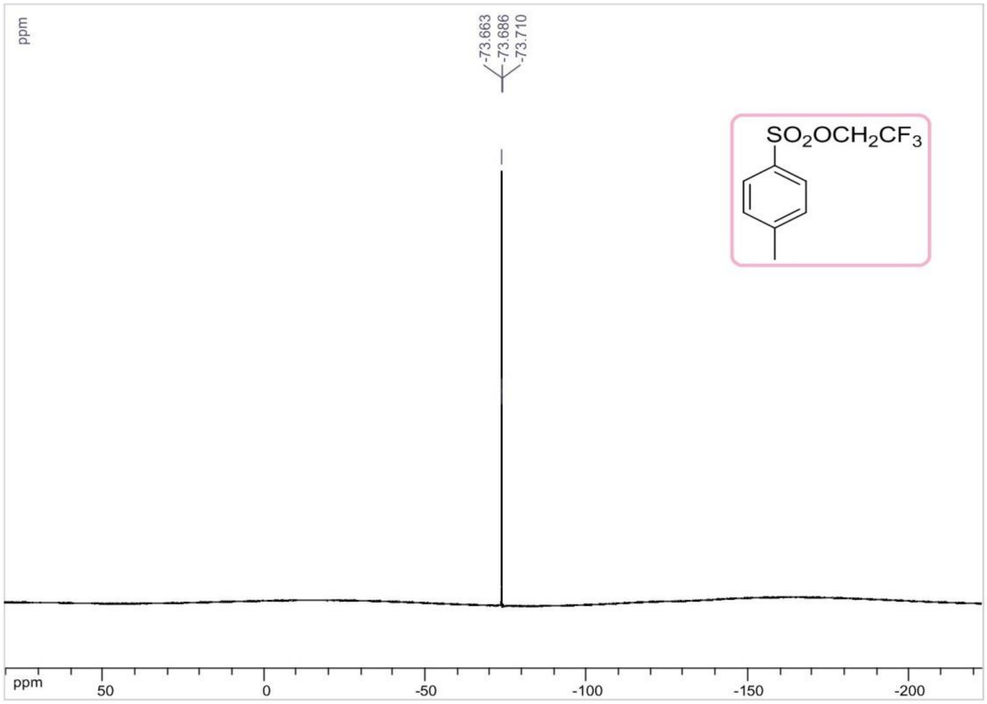
ppm
-73.663
-73.686
-73.710
SO2OCH2CF3
ppm
50
0
-50
-100
-150
-200

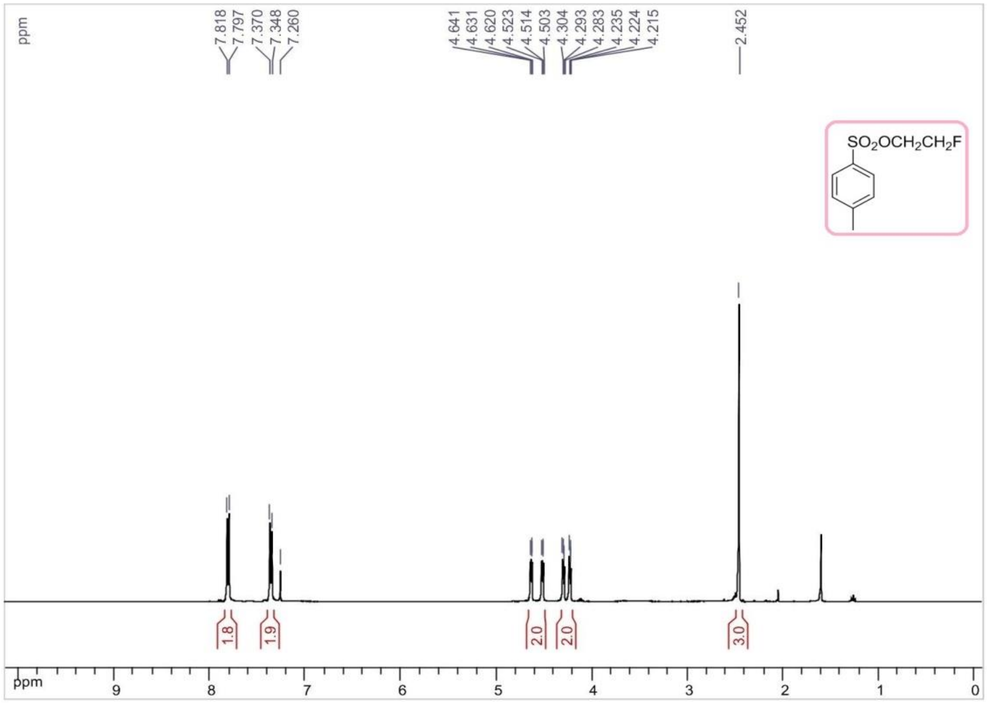
ppm
7.818
7.797
7.370
7.348
7.260
4.641
4.631
4.620
4.523
4.514
4.503
4.304
4.293
4.283
4.235
4.224
4.215
2.452
SO2OCH2CH2F
1.8
1.9
2.0
2.0
3.0
ppm
9
8
7
6
5
4
3
2
1
0

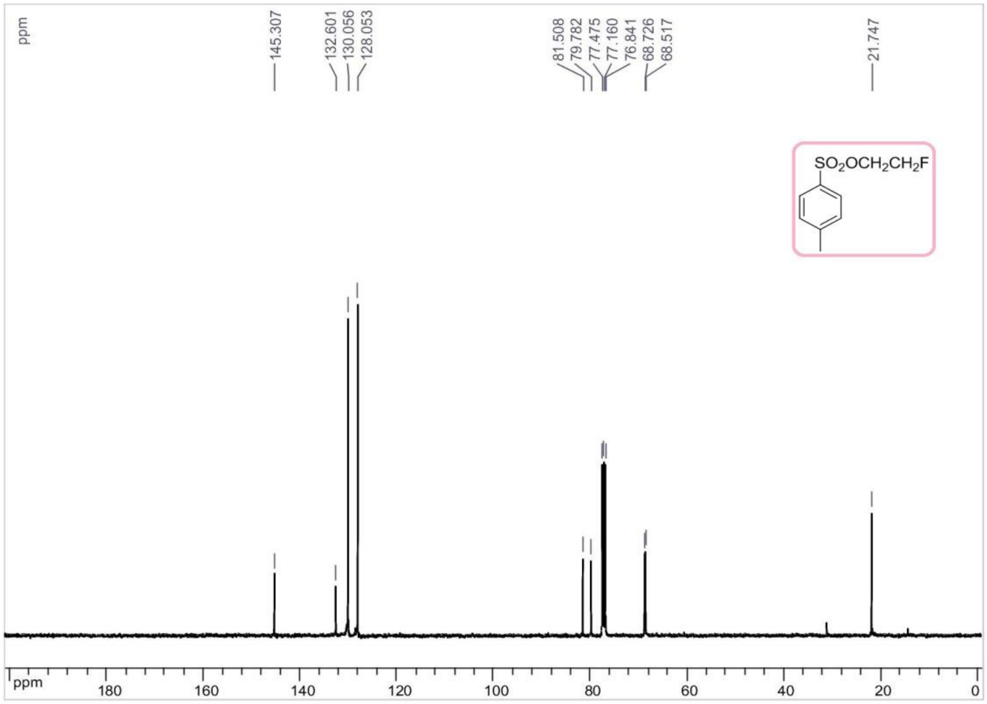
ppm
145.307
132.601
130.056
128.053
81.508
79.782
77.475
77.160
76.841
68.726
68.517
21.747
SO2OCH2CH2F
ppm 180 160 140 120 100 80 60 40 20 0

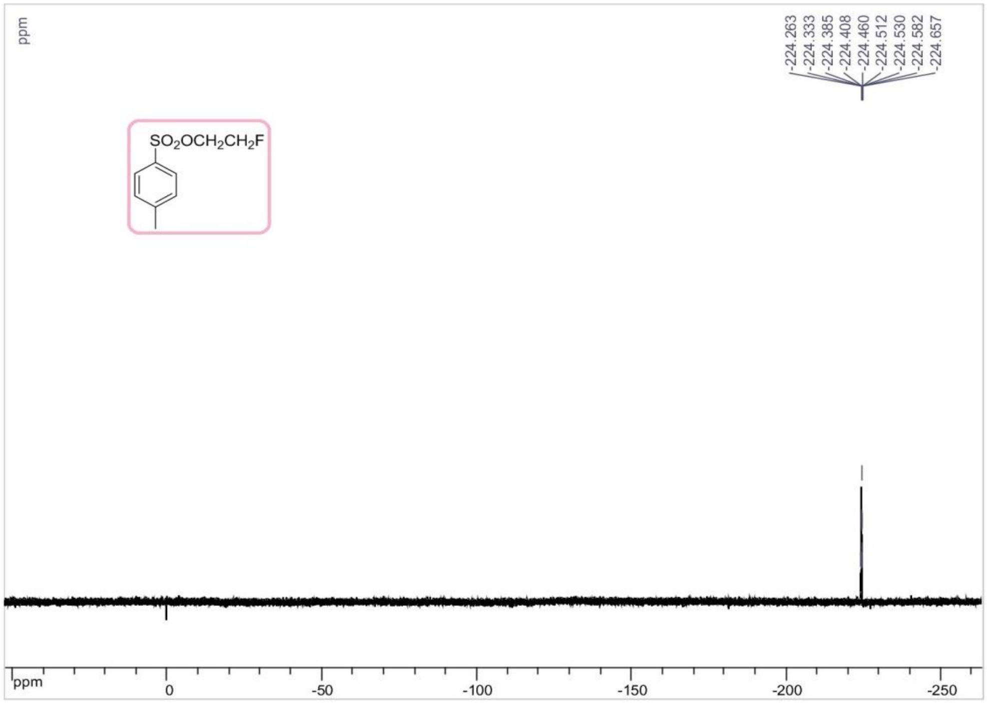
ppm
-224.263
-224.333
-224.385
-224.408
-224.460
-224.512
-224.530
-224.582
-224.657
SO2OCH2CH2F
ppm 0 -50 -100 -150 -200 -250

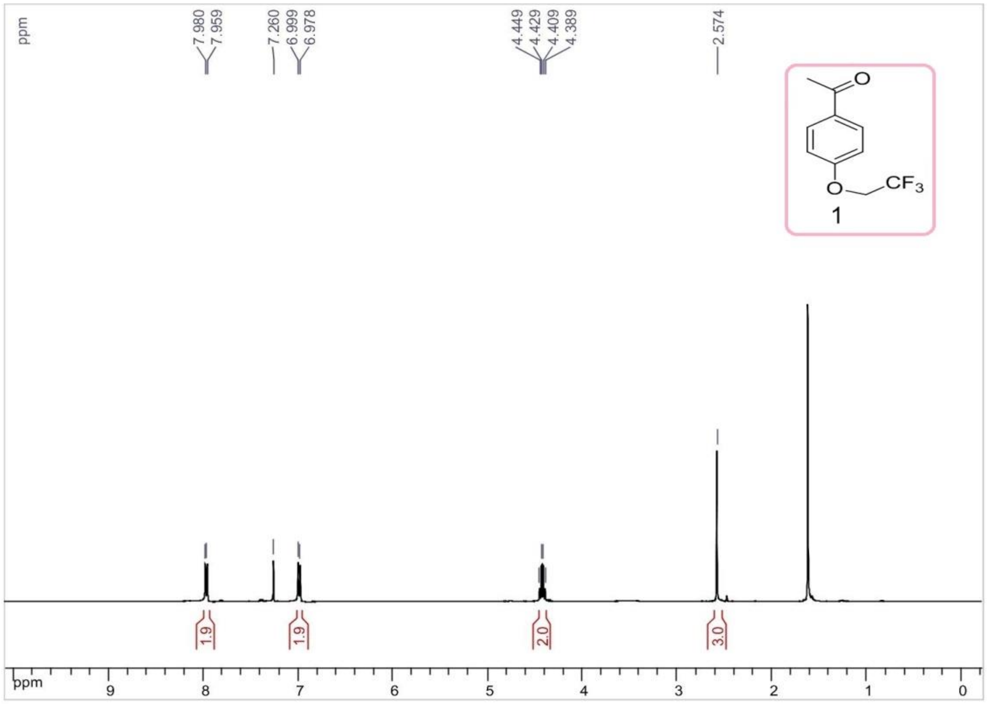
ppm
7.980
7.959
7.260
6.999
6.978
4.449
4.429
4.409
4.389
2.574
1
1.9
1.9
2.0
3.0
ppm 9 8 7 6 5 4 3 2 1 0

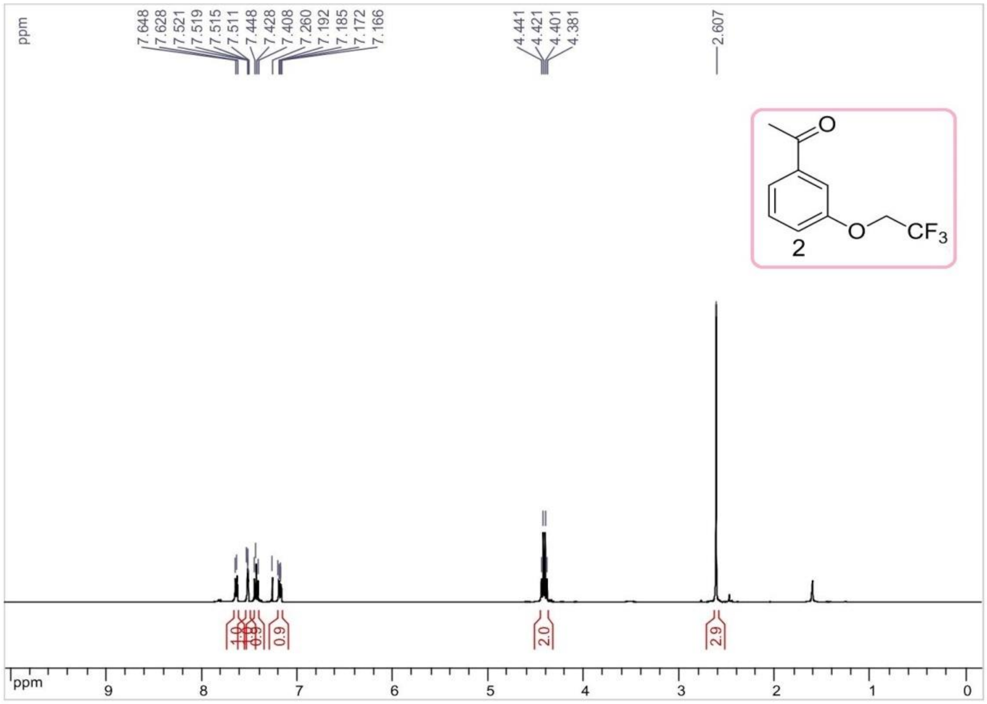
ppm
7.648
7.628
7.521
7.519
7.515
7.511
7.448
7.428
7.408
7.260
7.192
7.185
7.172
7.166
4.441
4.421
4.401
4.381
2.607
2
0.9
2.0
2.9
ppm 9 8 7 6 5 4 3 2 1 0

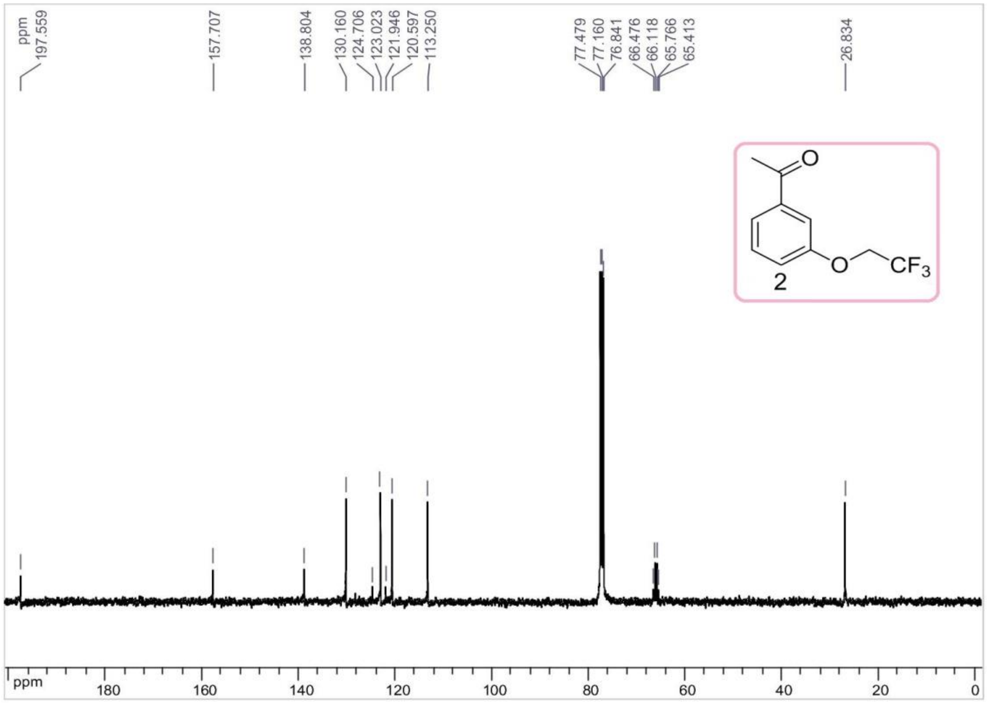
ppm
197.559
157.707
138.804
130.160
124.706
123.023
121.946
120.597
113.250
77.479
77.160
76.841
66.476
66.118
65.766
65.413
26.834
O
O
CF3
2
ppm
180
160
140
120
100
80
60
40
20
0

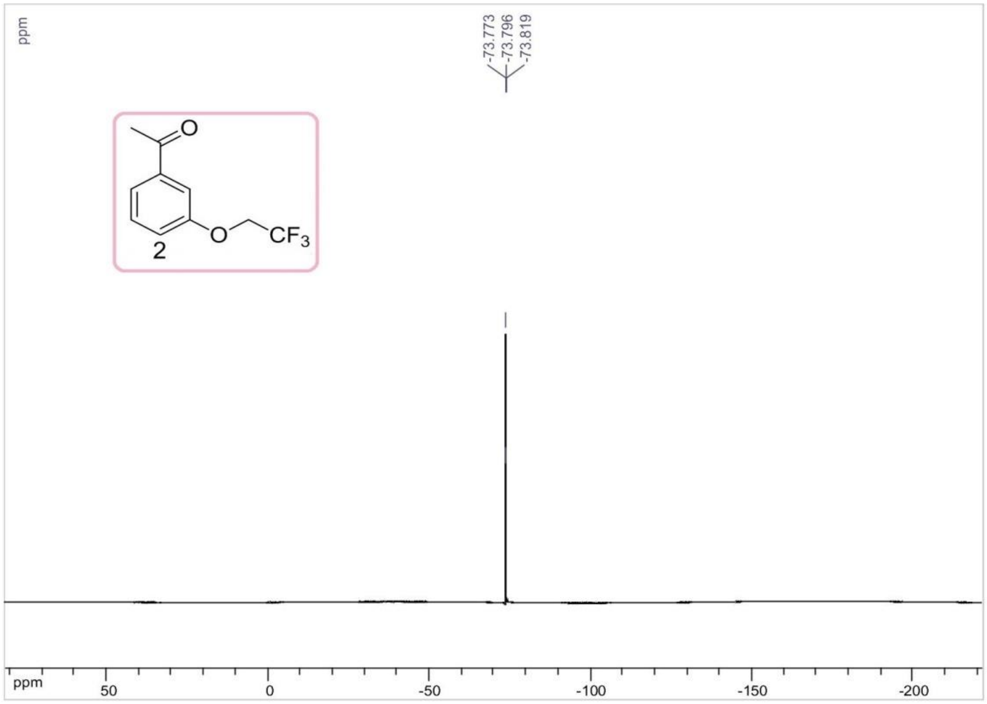
ppm
-73.773
-73.796
-73.819
O
O
CF3
2
ppm
50
0
-50
-100
-150
-200

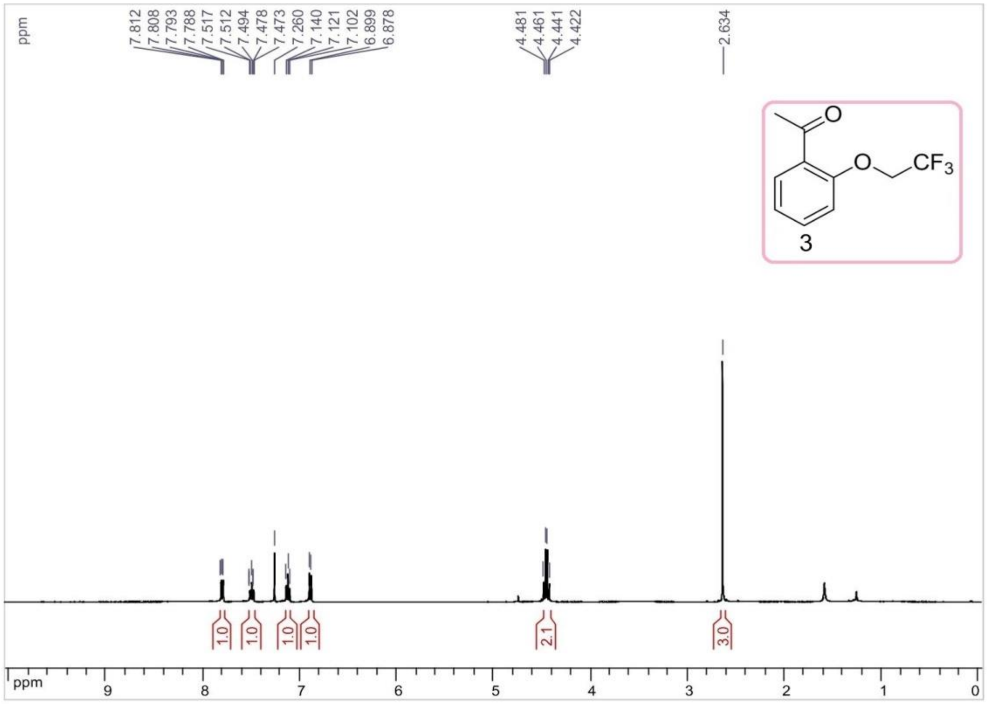
ppm
7.812
7.808
7.793
7.788
7.517
7.512
7.494
7.478
7.473
7.260
7.140
7.121
7.102
6.899
6.878
4.481
4.461
4.441
4.422
2.634
O
O
CF3
3
1.0
1.0
1.0
1.0
2.1
3.0
ppm
9
8
7
6
5
4
3
2
1
0

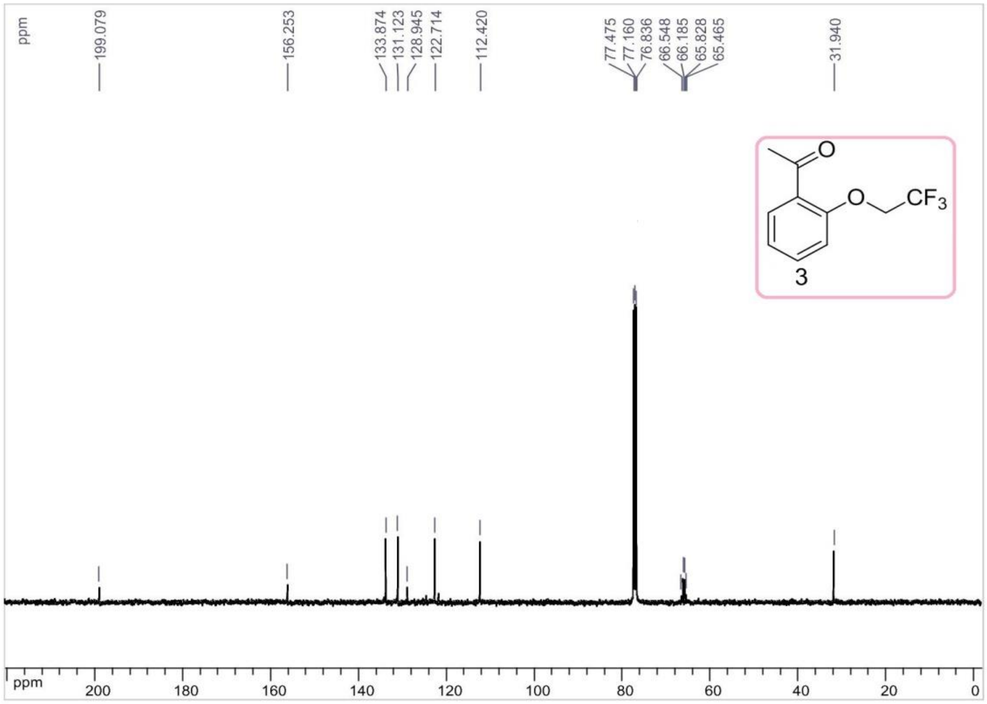
ppm
199.079
156.253
133.874
131.123
128.945
122.714
112.420
77.475
77.160
76.836
66.548
66.185
65.828
65.465
31.940
O
O
CF3
3
ppm
200
180
160
140
120
100
80
60
40
20
0

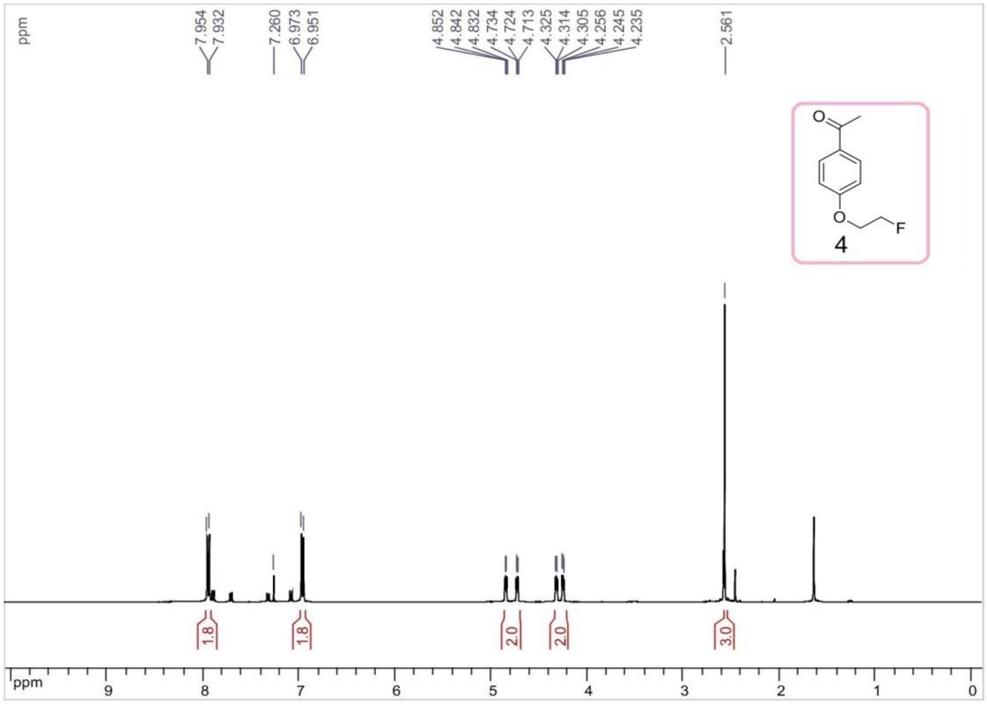
ppm
7.954
7.932
7.260
6.973
6.951
4.852
4.842
4.832
4.734
4.724
4.713
4.325
4.314
4.305
4.256
4.245
4.235
2.561
O
F
4
1.8
1.8
2.0
2.0
3.0
ppm 9 8 7 6 5 4 3 2 1 0

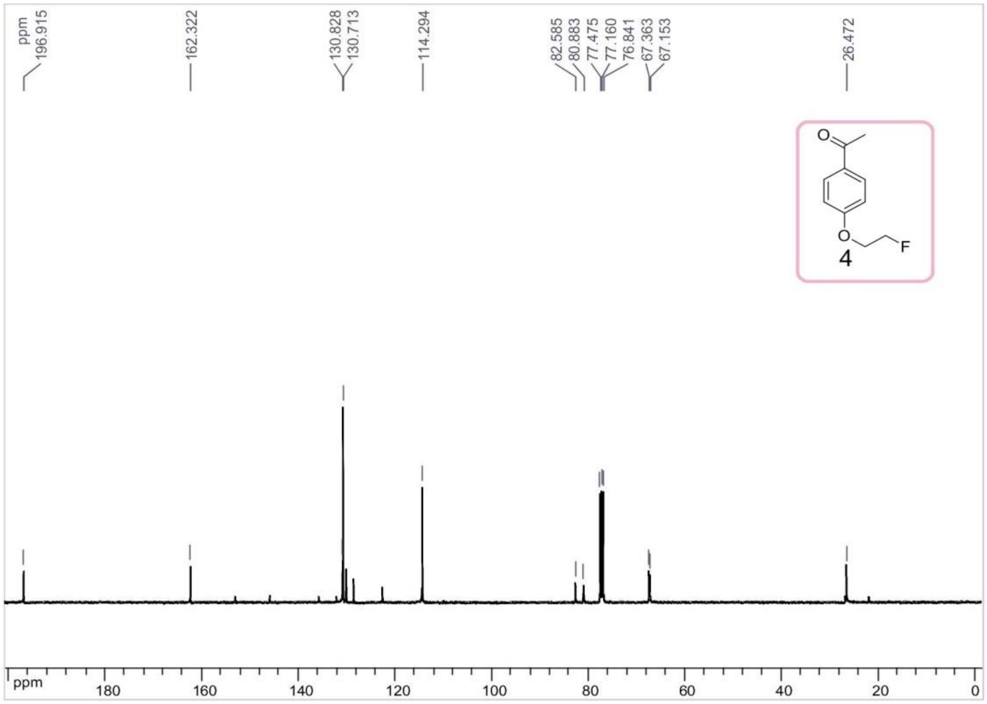
ppm
196.915
162.322
130.828
130.713
114.294
82.585
80.883
77.475
77.160
76.841
67.363
67.153
26.472
O
F
4
ppm 180 160 140 120 100 80 60 40 20 0

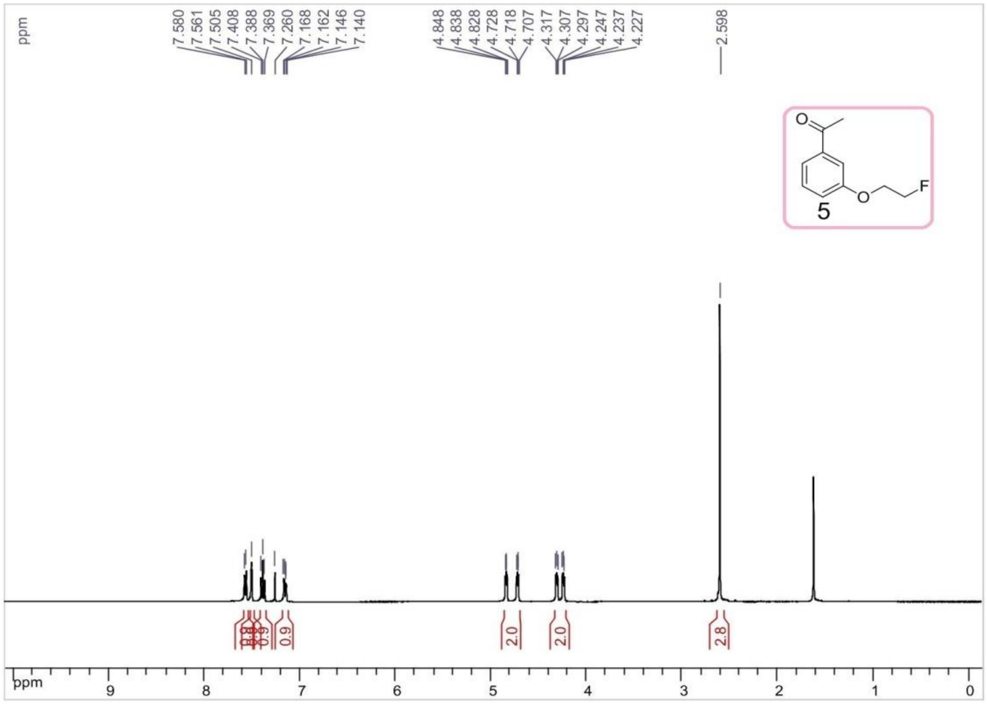
ppm
7.580
7.561
7.505
7.408
7.388
7.369
7.260
7.168
7.162
7.146
7.140
4.848
4.838
4.828
4.728
4.718
4.707
4.317
4.307
4.297
4.247
4.237
4.227
2.598
5
0.9
2.0
2.0
2.8
ppm
9
8
7
6
5
4
3
2
1
0

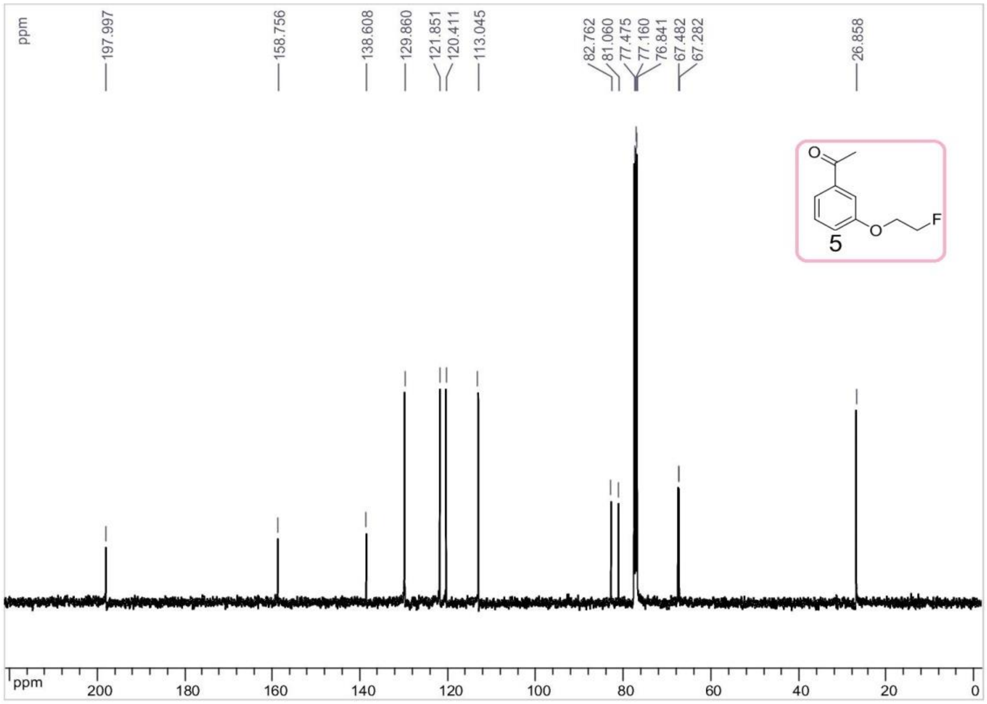
ppm
197.997
158.756
138.608
129.860
121.851
120.411
113.045
82.762
81.060
77.475
77.160
76.841
67.482
67.282
26.858
5
ppm
200
180
160
140
120
100
80
60
40
20
0

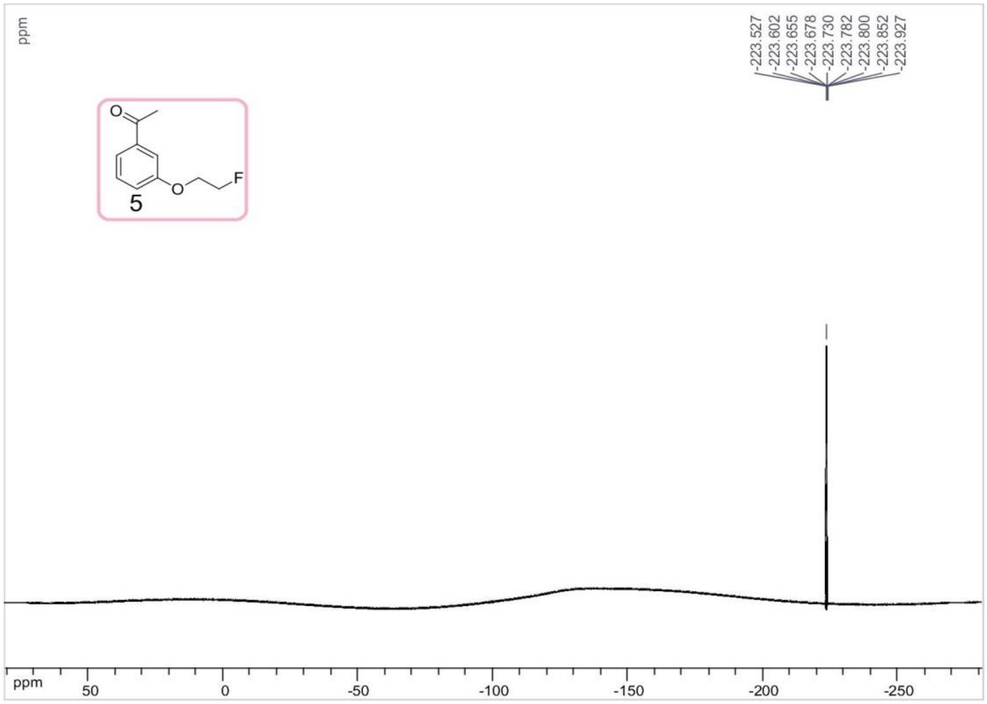
ppm
-223.527
-223.602
-223.655
-223.678
-223.730
-223.782
-223.800
-223.852
-223.927
O
F
5
ppm 50 0 -50 -100 -150 -200 -250

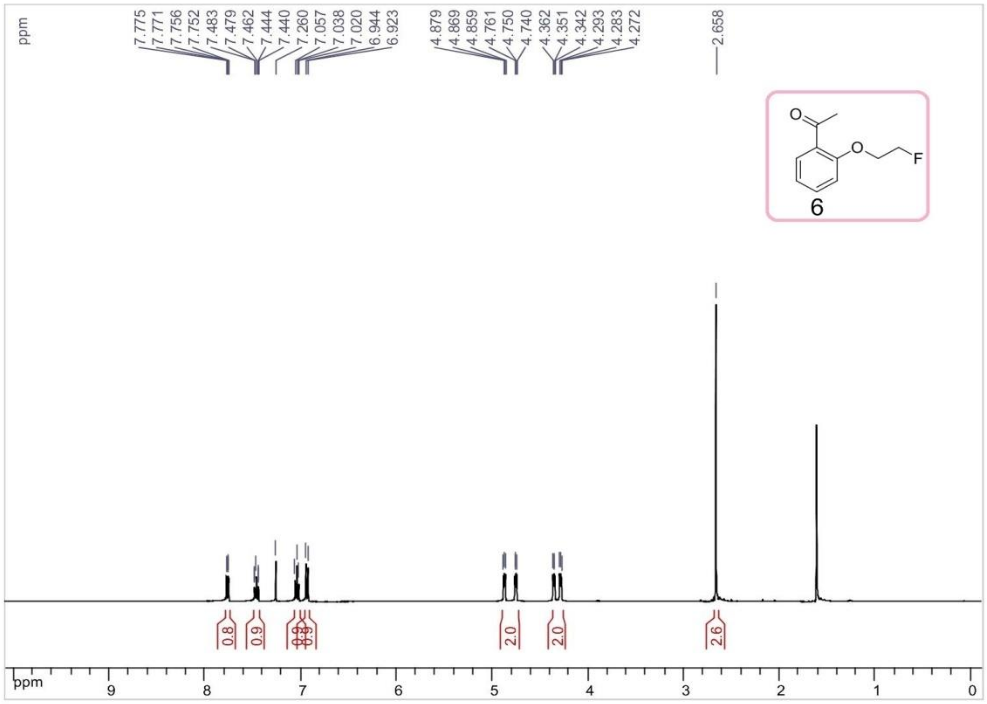
ppm
7.775
7.771
7.756
7.752
7.483
7.479
7.462
7.444
7.440
7.260
7.057
7.038
7.020
6.944
6.923
4.879
4.869
4.859
4.761
4.750
4.740
4.362
4.351
4.342
4.293
4.283
4.272
2.658
O
F
6
0.8
0.9
2.0
2.0
2.6
ppm 9 8 7 6 5 4 3 2 1 0

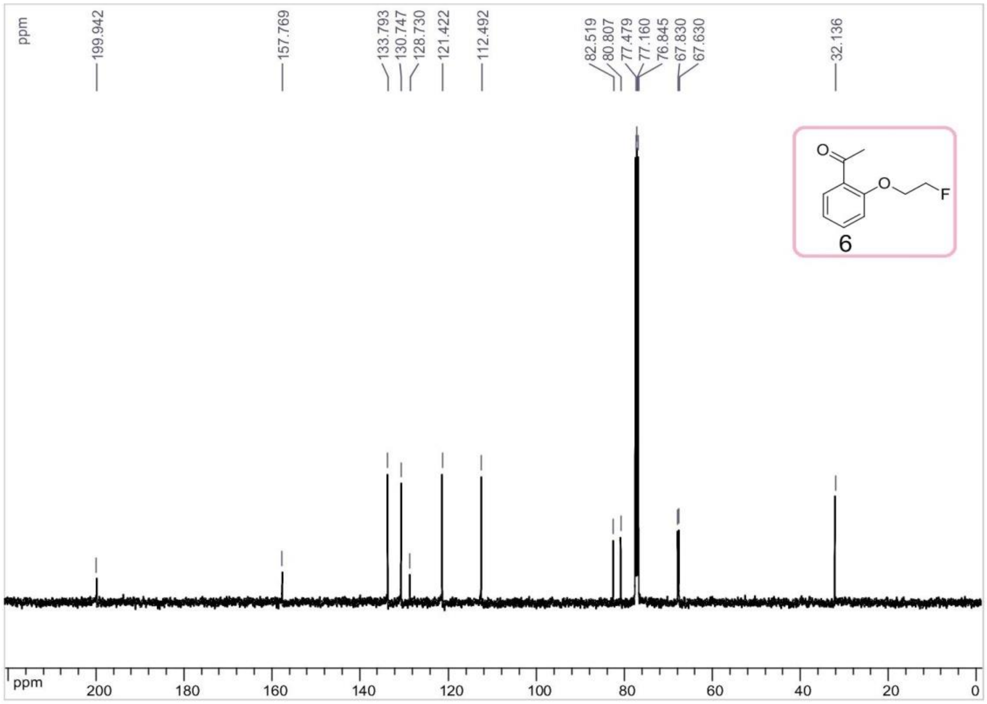
ppm
199.942
157.769
133.793
130.747
128.730
121.422
112.492
82.519
80.807
77.479
77.160
76.845
67.830
67.630
32.136
O
O
F
6
ppm
200
180
160
140
120
100
80
60
40
20
0

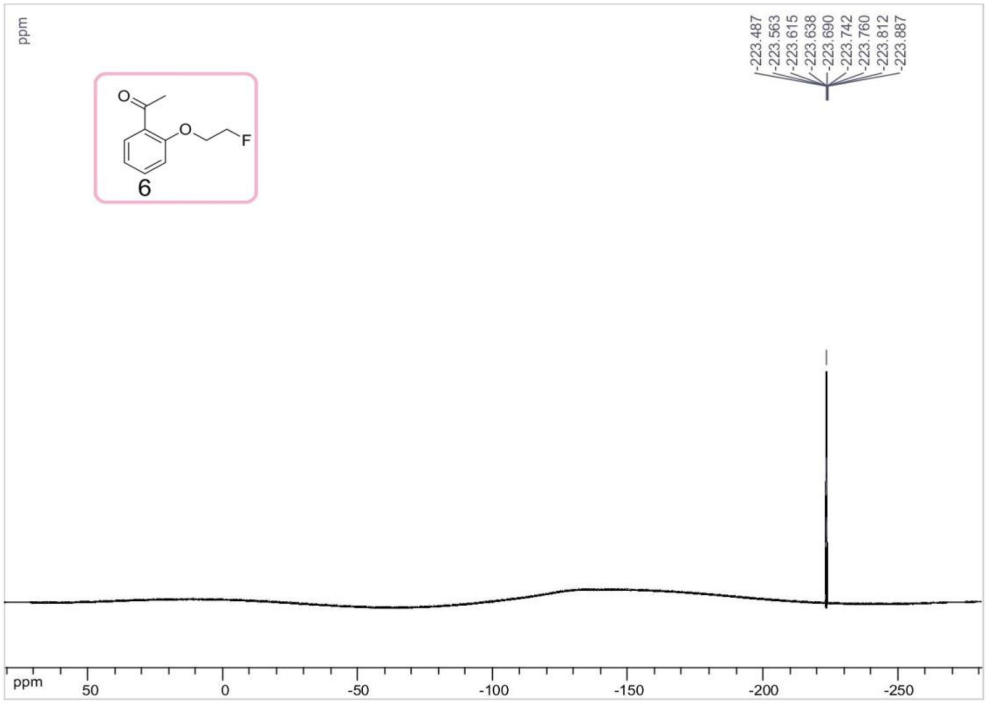
ppm
-223.487
-223.563
-223.615
-223.638
-223.690
-223.742
-223.760
-223.812
-223.887
O
O
F
6
ppm
50
0
-50
-100
-150
-200
-250

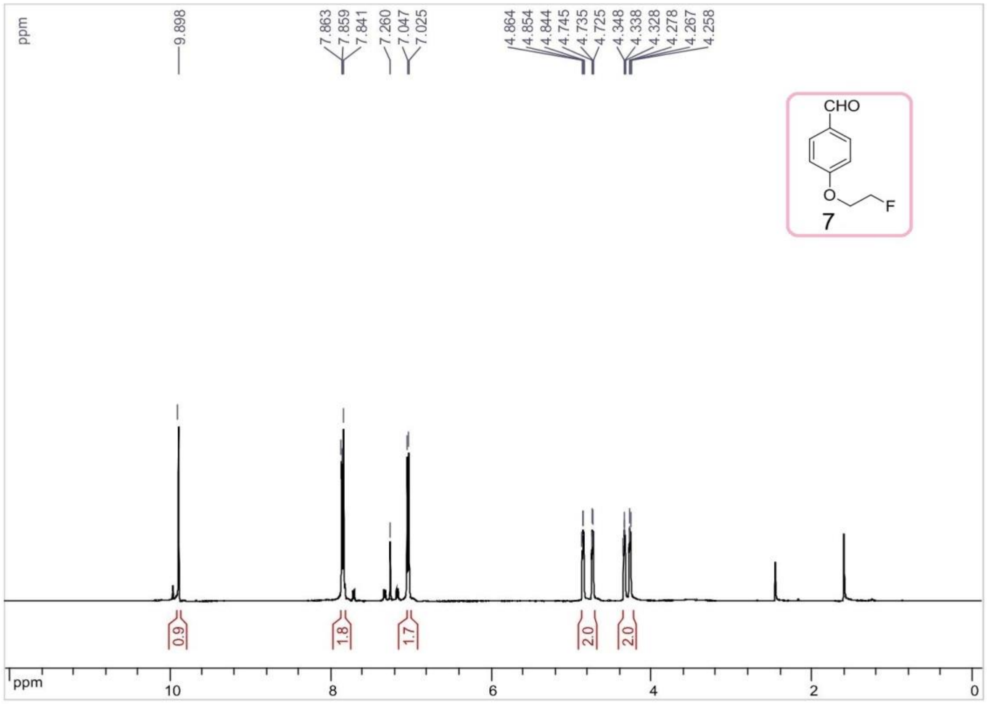
ppm
9.898
7.863
7.859
7.841
7.260
7.047
7.025
4.864
4.854
4.844
4.745
4.735
4.725
4.348
4.338
4.328
4.278
4.267
4.258
CHO
O
F
7
0.9
1.8
1.7
2.0
2.0
ppm
10
8
6
4
2
0

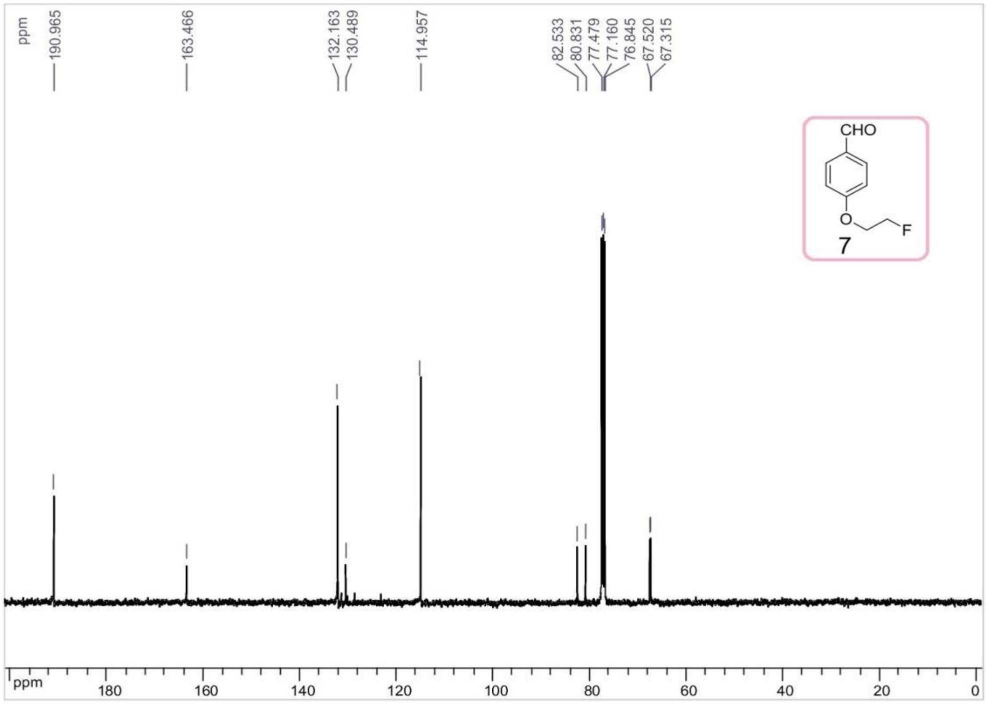
ppm
190.965
163.466
132.163
130.489
114.957
82.533
80.831
77.479
77.160
76.845
67.520
67.315
CHO
O
F
7
ppm
180
160
140
120
100
80
60
40
20
0

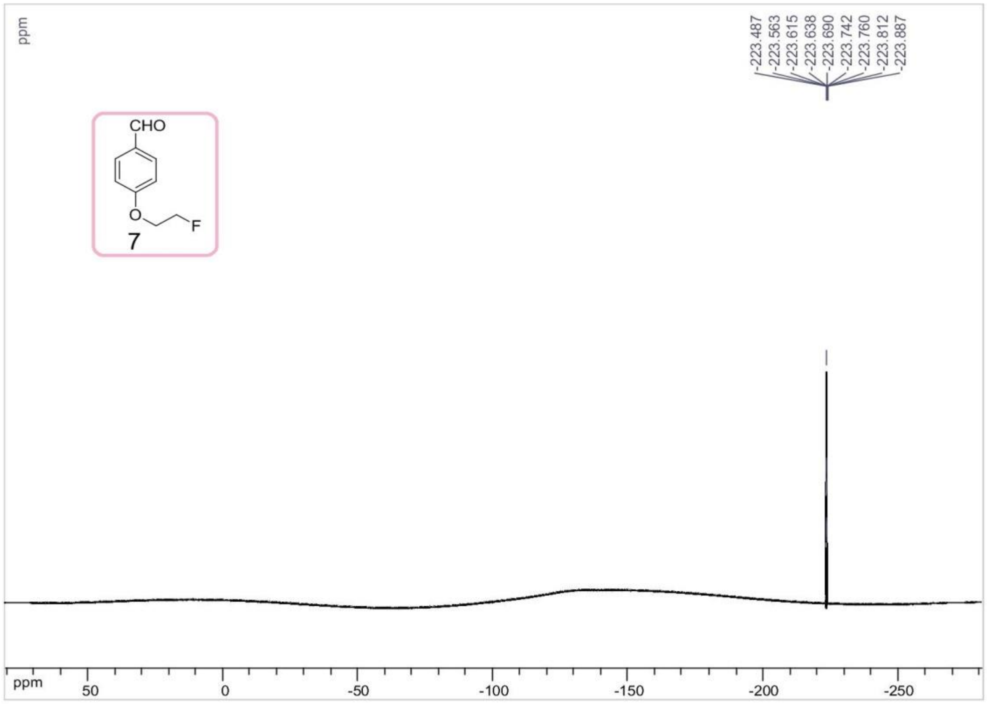
ppm
-223.487
-223.563
-223.615
-223.638
-223.690
-223.742
-223.760
-223.812
-223.887
CHO
O
F
7
ppm 50 0 -50 -100 -150 -200 -250

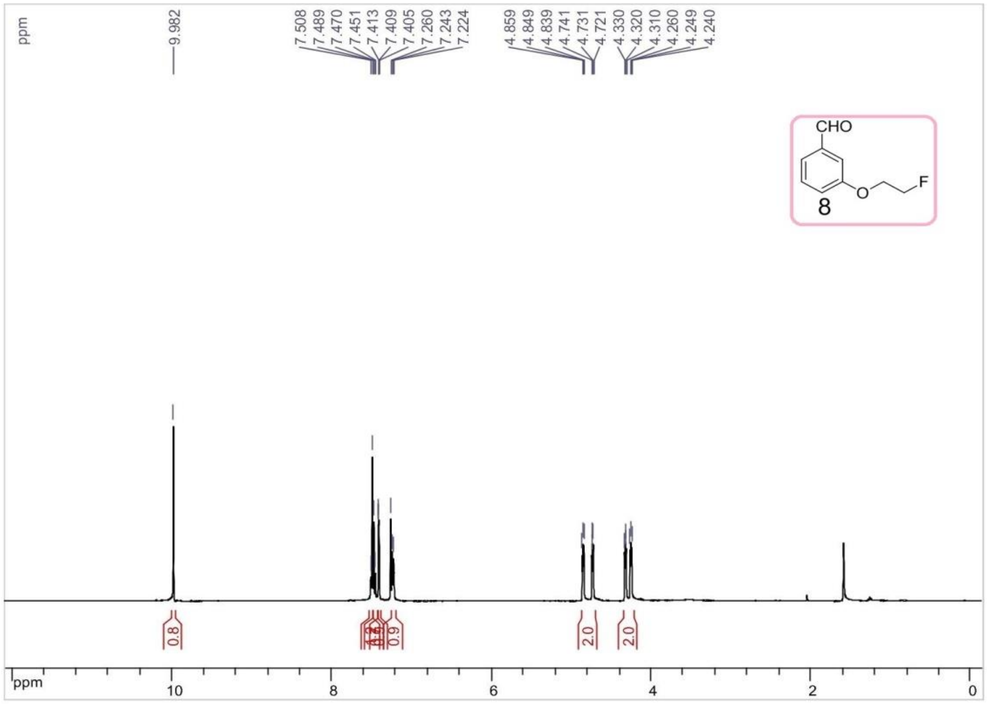
ppm
9.982
7.508
7.489
7.470
7.451
7.413
7.409
7.405
7.260
7.243
7.224
4.859
4.849
4.839
4.741
4.731
4.721
4.330
4.320
4.310
4.260
4.249
4.240
CHO
O
F
8
0.8
0.9
2.0
2.0
ppm 10 8 6 4 2 0

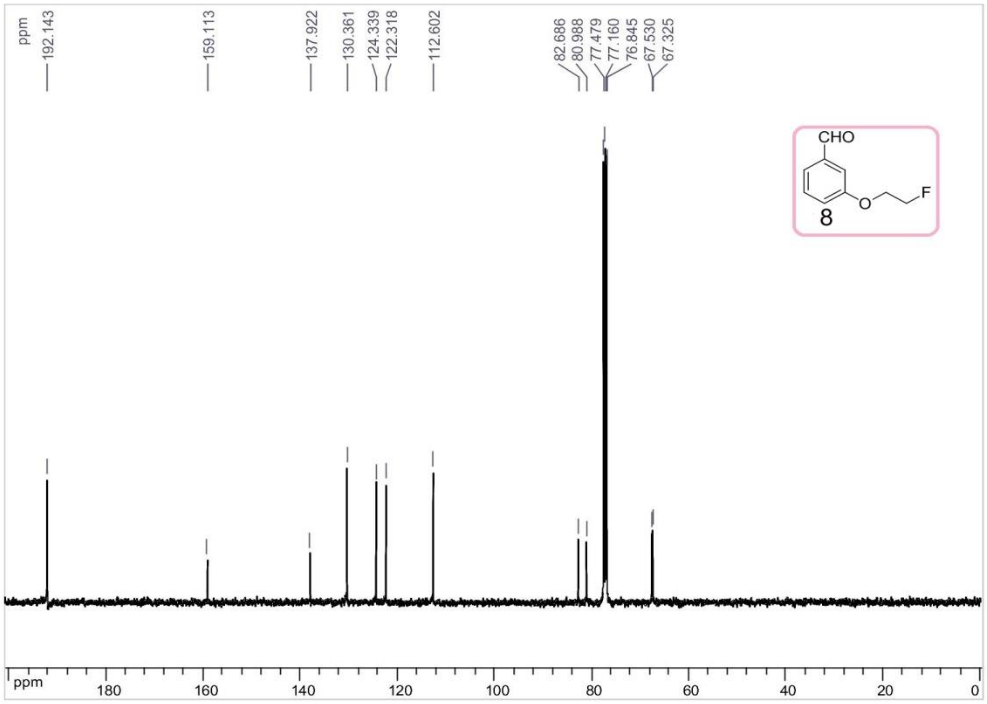
ppm
192.143
159.113
137.922
130.361
124.339
122.318
112.602
82.686
80.988
77.479
77.160
76.845
67.530
67.325
CHO
O
F
8
ppm
180
160
140
120
100
80
60
40
20
0

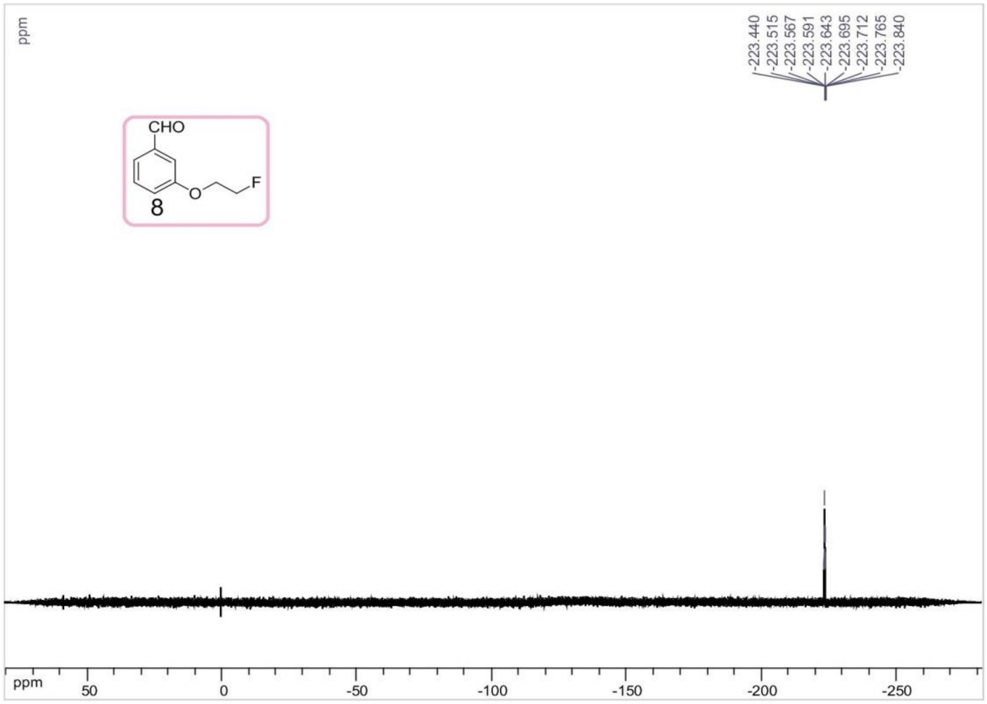
ppm
-223.440
-223.515
-223.567
-223.591
-223.643
-223.695
-223.712
-223.765
-223.840
CHO
O
F
8
ppm
50
0
-50
-100
-150
-200
-250

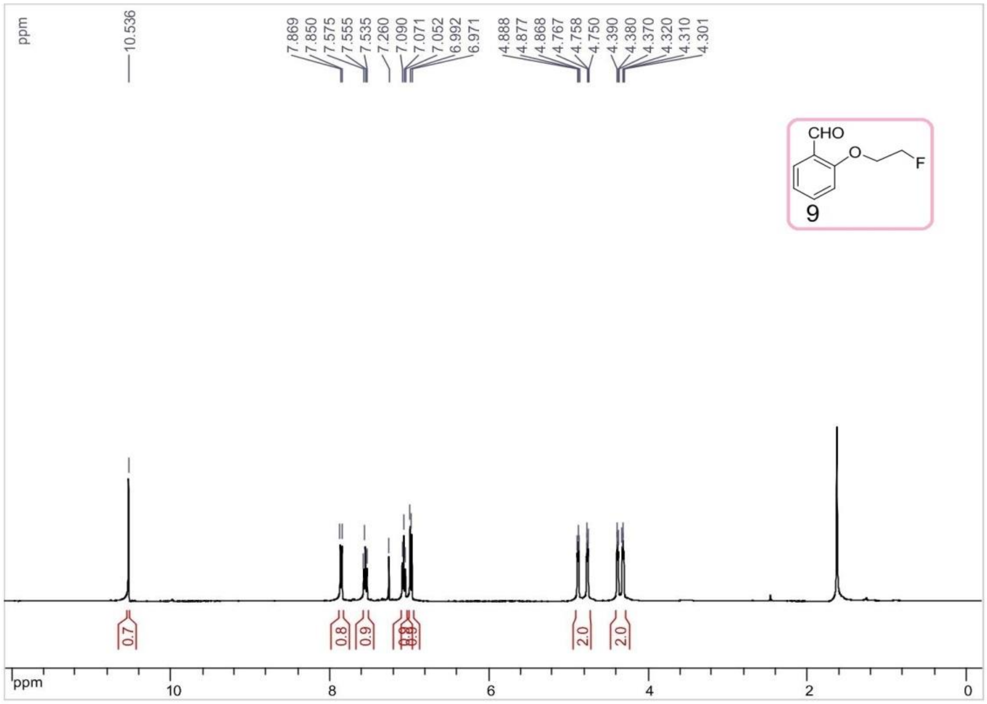
ppm
10.536
7.869
7.850
7.575
7.555
7.535
7.260
7.090
7.071
7.052
6.992
6.971
4.888
4.877
4.868
4.767
4.758
4.750
4.390
4.380
4.370
4.320
4.310
4.301
CHO
O
F
9
0.7
0.8
0.9
2.0
2.0

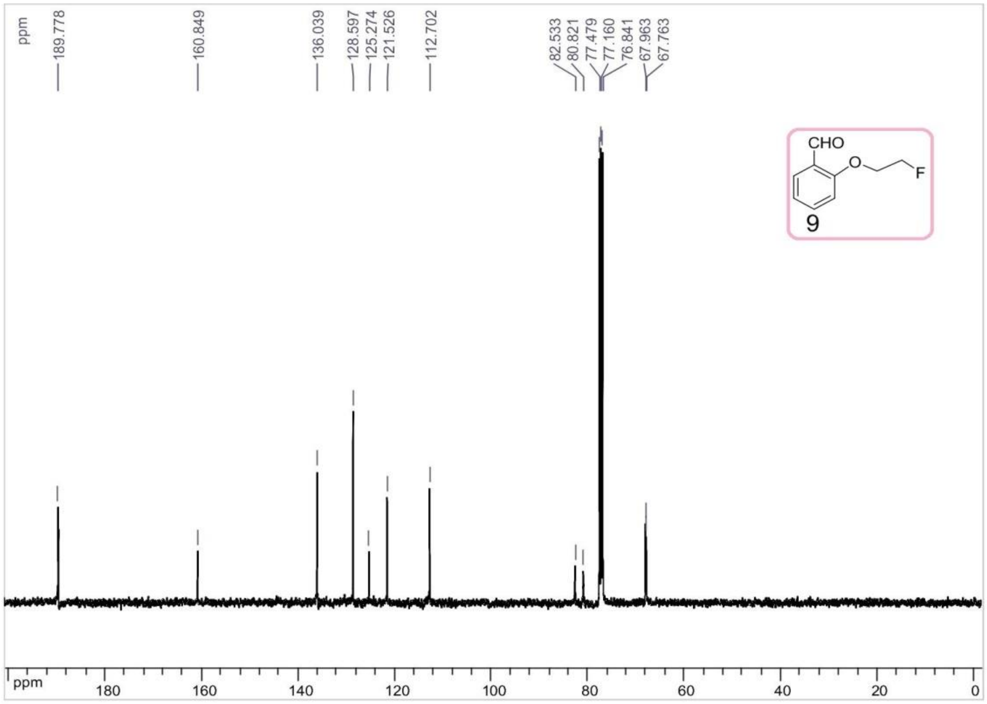
ppm
189.778
160.849
136.039
128.597
125.274
121.526
112.702
82.533
80.821
77.479
77.160
76.841
67.963
67.763
CHO
O
F
9

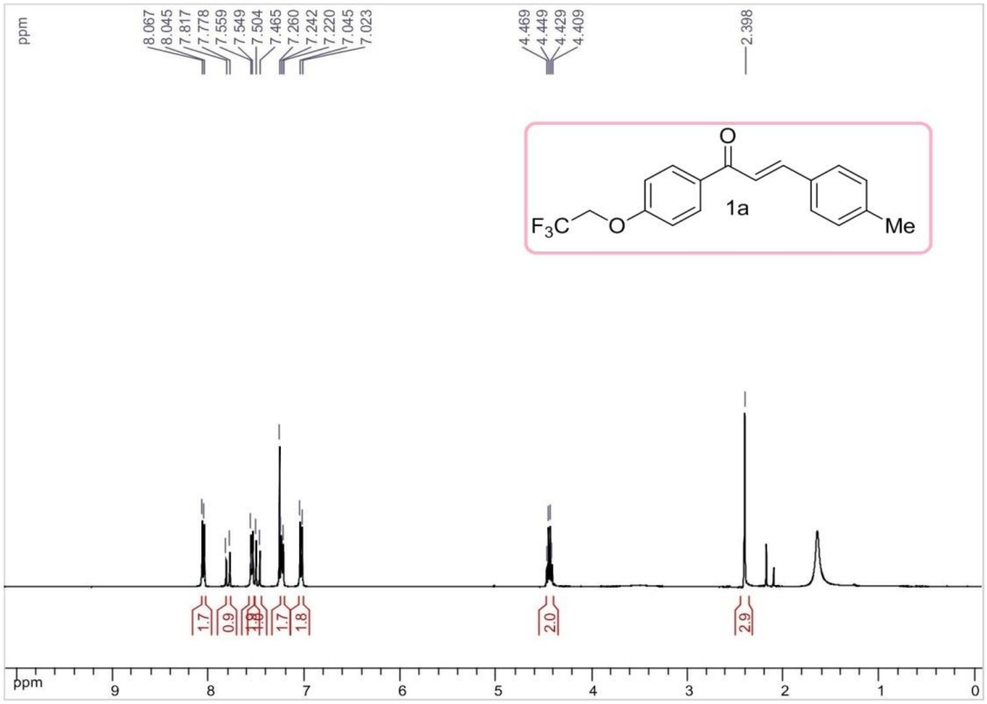
ppm
8.067
8.045
7.817
7.778
7.559
7.549
7.504
7.465
7.260
7.242
7.220
7.045
7.023
4.469
4.449
4.429
4.409
2.398
O
F3C
1a
Me
1.7
0.9
1.7
1.8
2.0
2.9
ppm
9
8
7
6
5
4
3
2
1
0

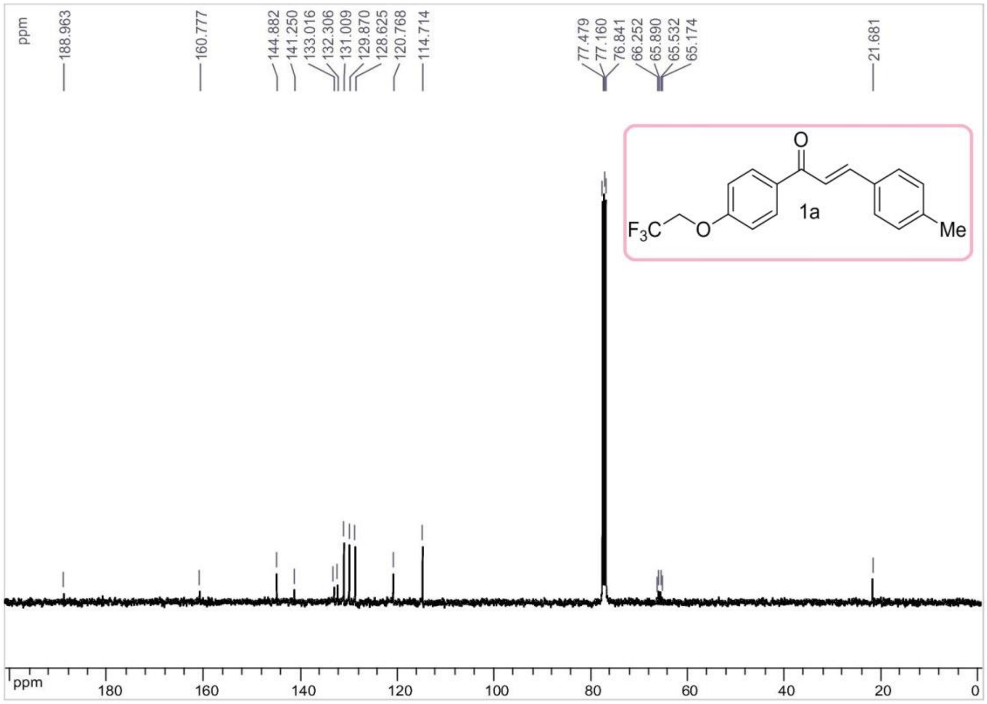
ppm
188.963
160.777
144.882
141.250
133.016
132.306
131.009
129.870
128.625
120.768
114.714
77.479
77.160
76.841
66.252
65.890
65.532
65.174
21.681
O
F3C
1a
Me
ppm
180
160
140
120
100
80
60
40
20
0

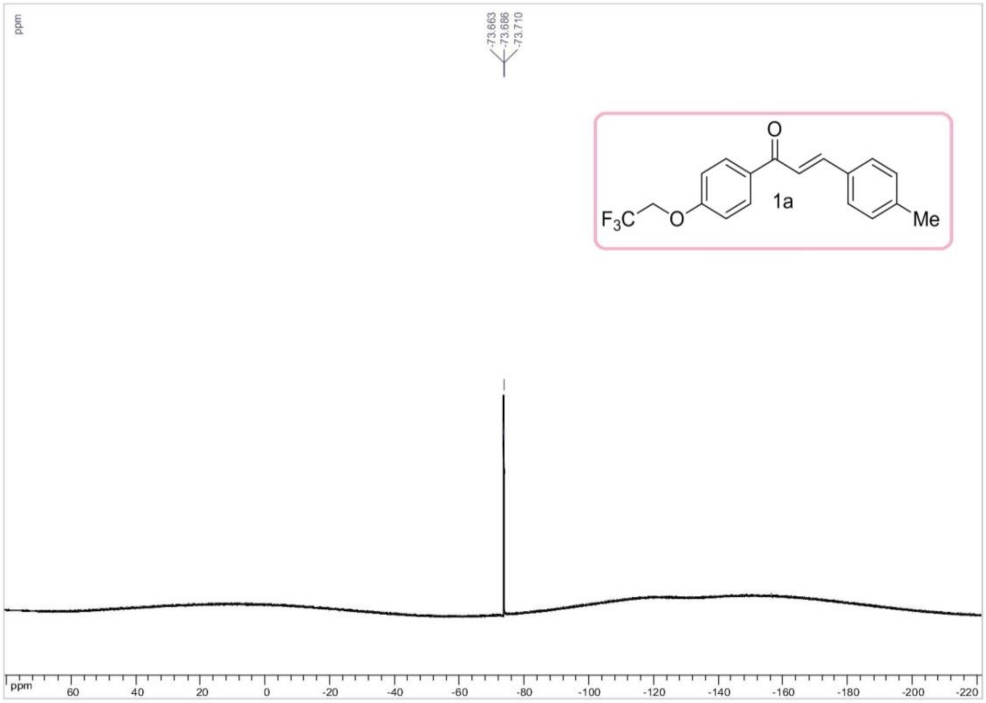
-73.663
-73.686
-73.710
1a
F3C
O
Me
ppm
60 40 20 0 -20 -40 -60 -80 -100 -120 -140 -160 -180 -200 -220

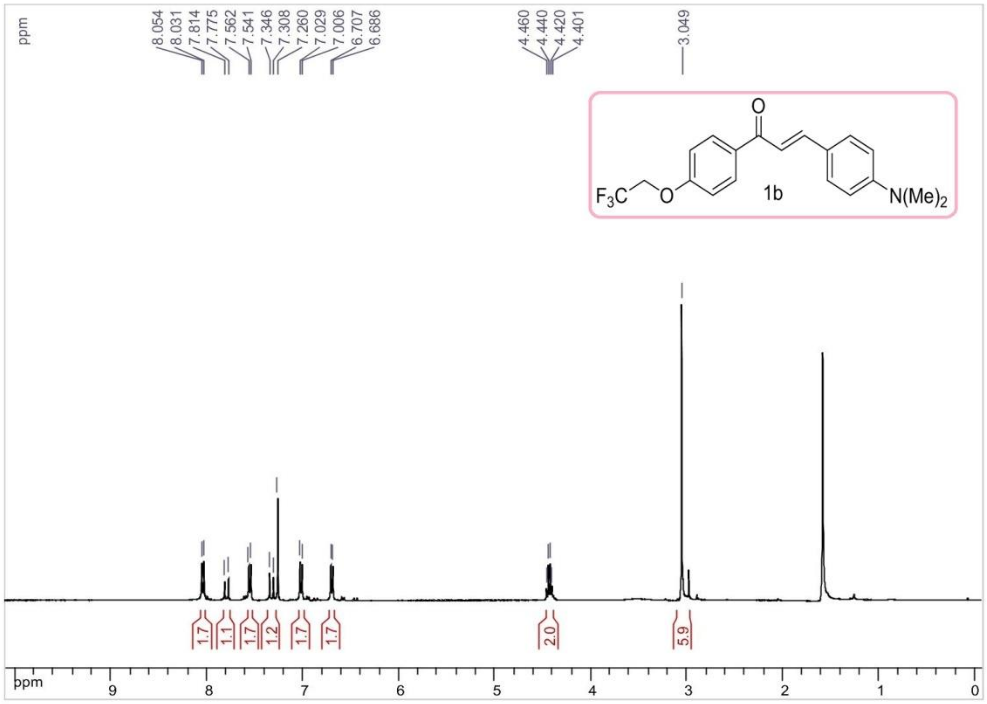
8.054
8.031
7.814
7.775
7.562
7.541
7.346
7.308
7.260
7.029
7.006
6.707
6.686
4.460
4.440
4.420
4.401
3.049
1b
F3C
O
N(Me)2
1.7
1.1
1.7
1.2
1.7
1.7
2.0
5.9
ppm
9 8 7 6 5 4 3 2 1 0

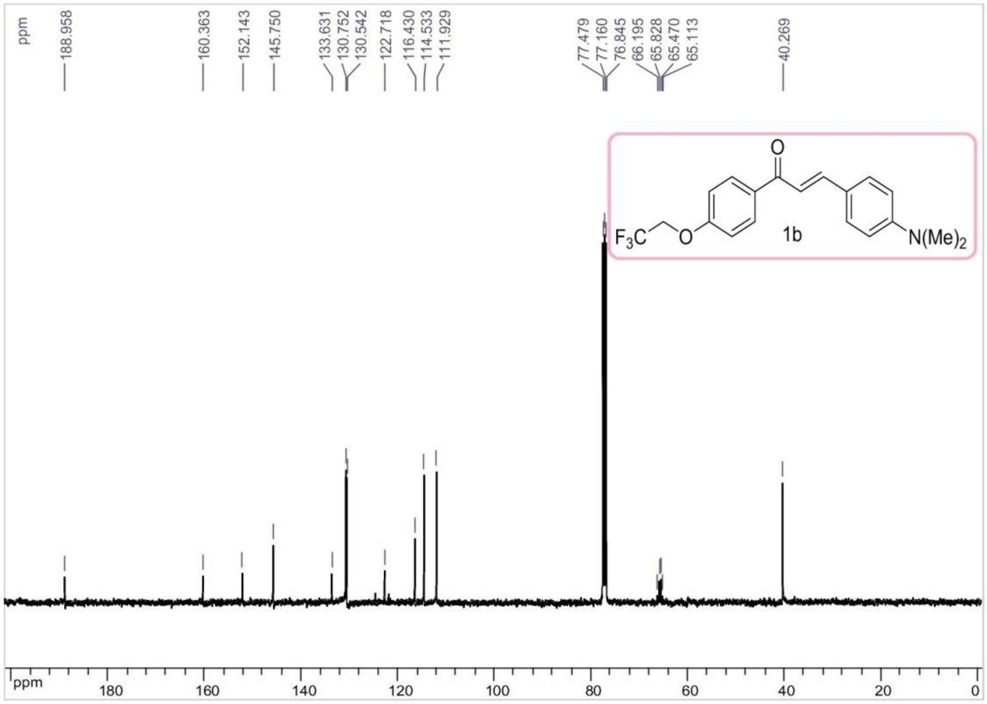
ppm
188.958
160.363
152.143
145.750
133.631
130.752
130.542
122.718
116.430
114.533
111.929
77.479
77.160
76.845
66.195
65.828
65.470
65.113
40.269
O
F_3C
O
1b
N(Me)$_2$
ppm
180
160
140
120
100
80
60
40
20
0

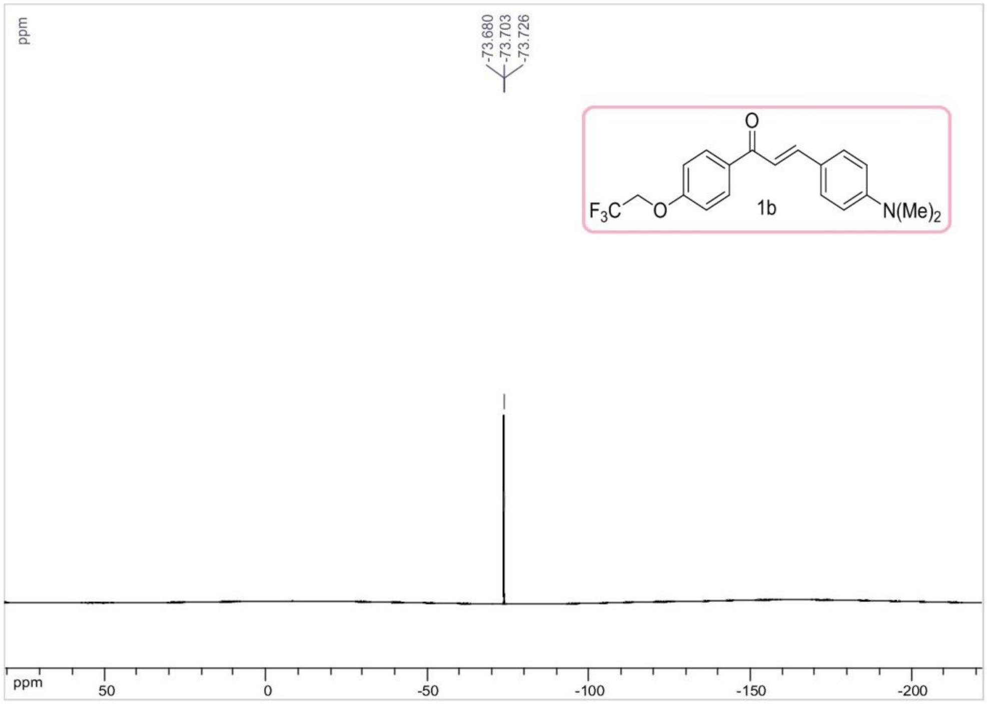
ppm
-73.680
-73.703
-73.726
O
F_3C
O
1b
N(Me)$_2$
ppm
50
0
-50
-100
-150
-200

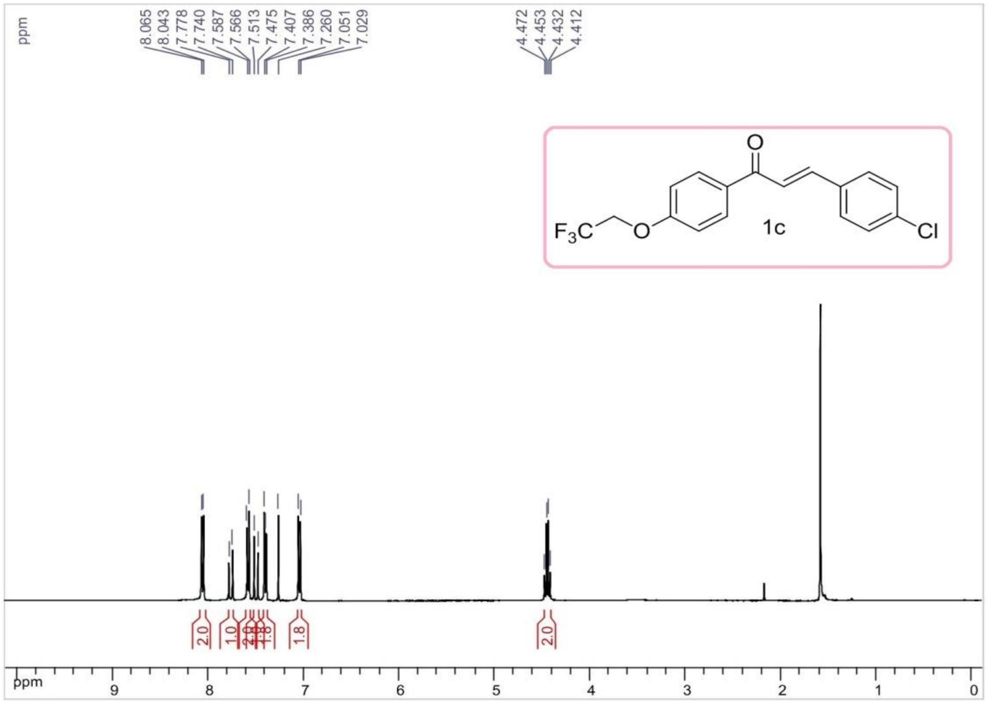
ppm
8.065
8.043
7.778
7.740
7.587
7.566
7.513
7.475
7.407
7.386
7.260
7.051
7.029
4.472
4.453
4.432
4.412
1c
2.0
1.0
1.8
2.0
ppm
9
8
7
6
5
4
3
2
1
0

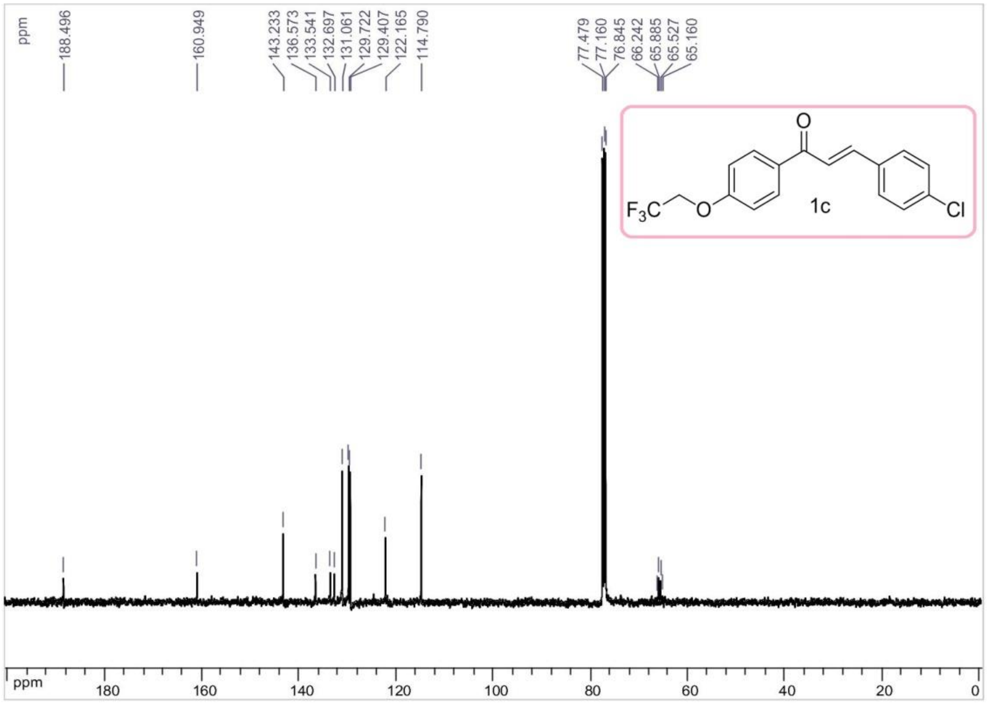
ppm
188.496
160.949
143.233
136.573
133.541
132.697
131.061
129.722
129.407
122.165
114.790
77.479
77.160
76.845
66.242
65.885
65.527
65.160
1c
ppm
180
160
140
120
100
80
60
40
20
0

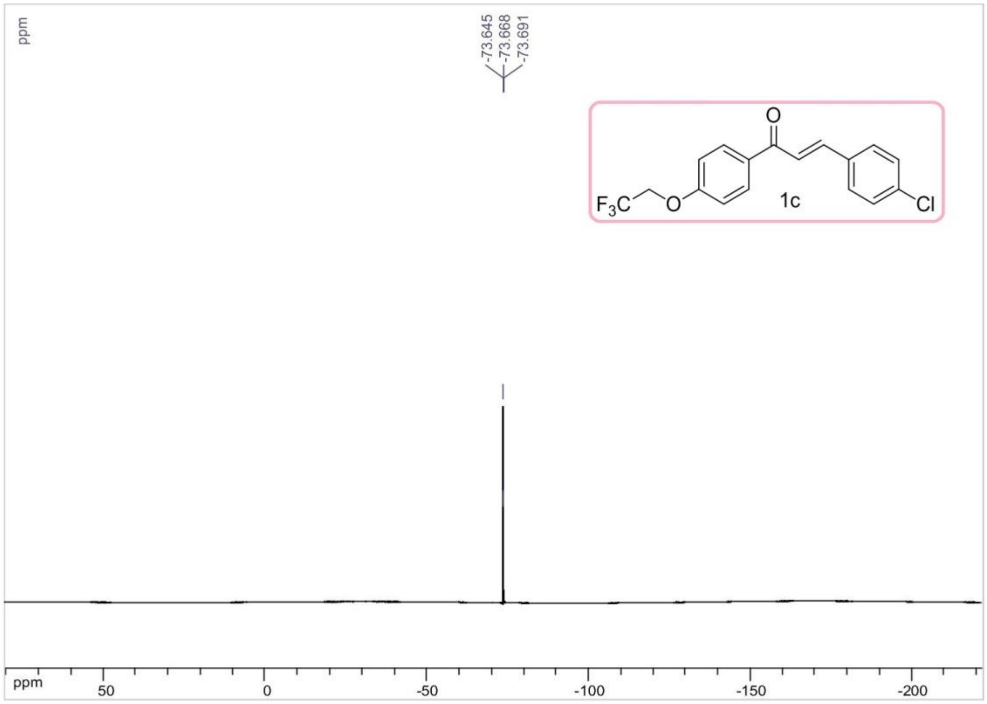
ppm
-73.645
-73.668
-73.691
O
F3C
O
1c
Cl
ppm
50
0
-50
-100
-150
-200

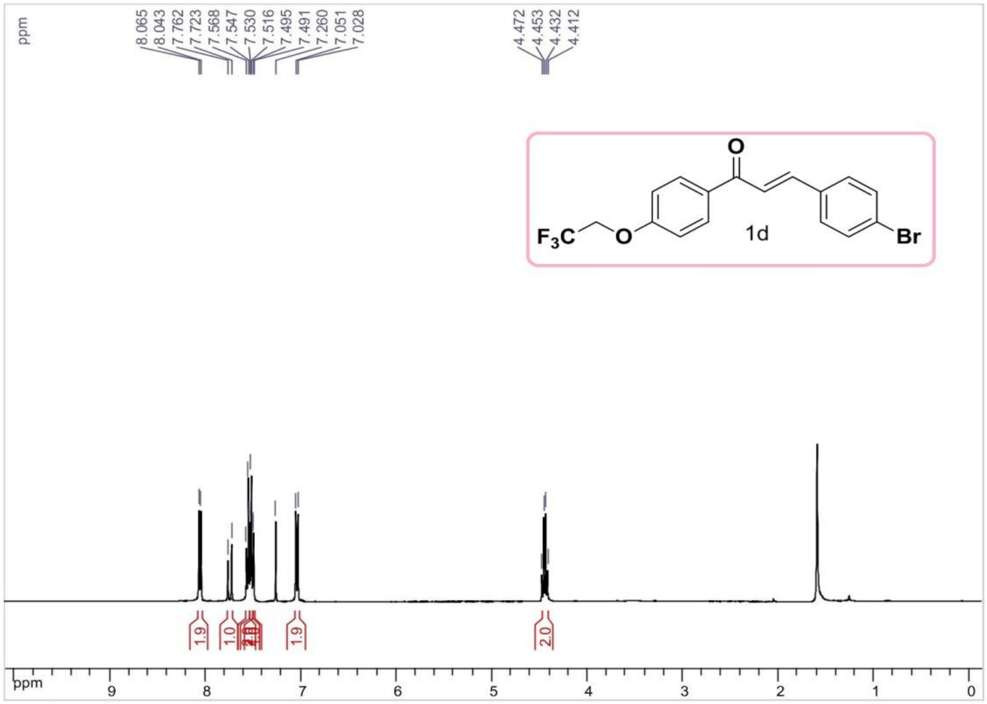
ppm
8.065
8.043
7.762
7.723
7.568
7.547
7.530
7.516
7.495
7.491
7.260
7.051
7.028
4.472
4.453
4.432
4.412
O
F3C
O
1d
Br
1.9
1.0
1.9
2.0
ppm
9
8
7
6
5
4
3
2
1
0

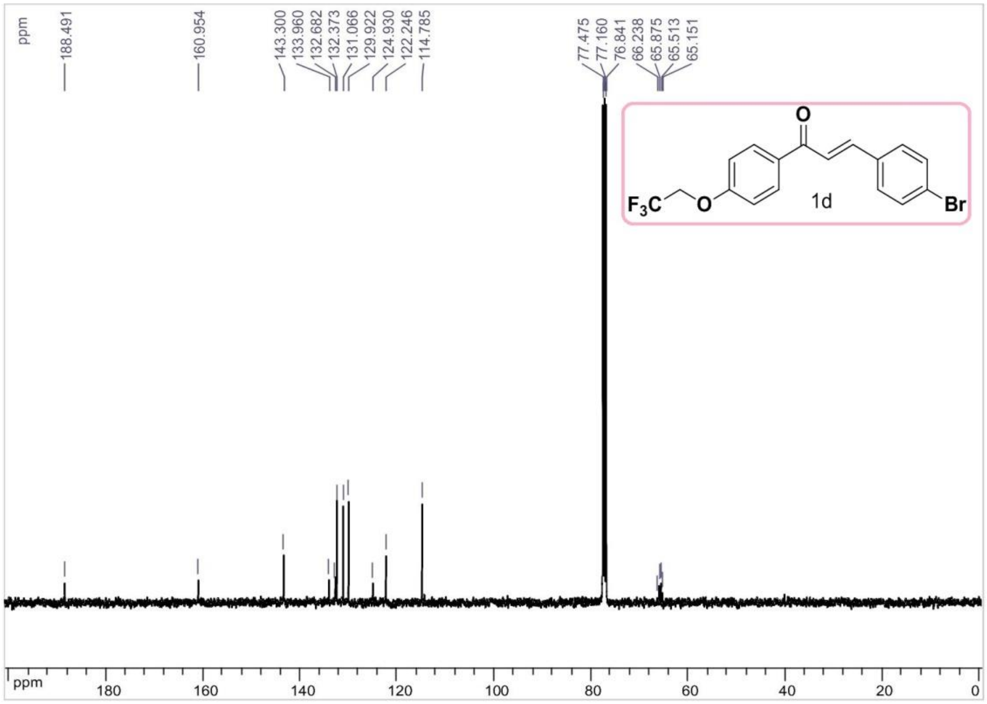
ppm
188.491
160.954
143.300
133.960
132.682
132.373
131.066
129.922
124.930
122.246
114.785
77.475
77.160
76.841
66.238
65.875
65.513
65.151
O
F3C
O
1d
Br
ppm
180
160
140
120
100
80
60
40
20
0

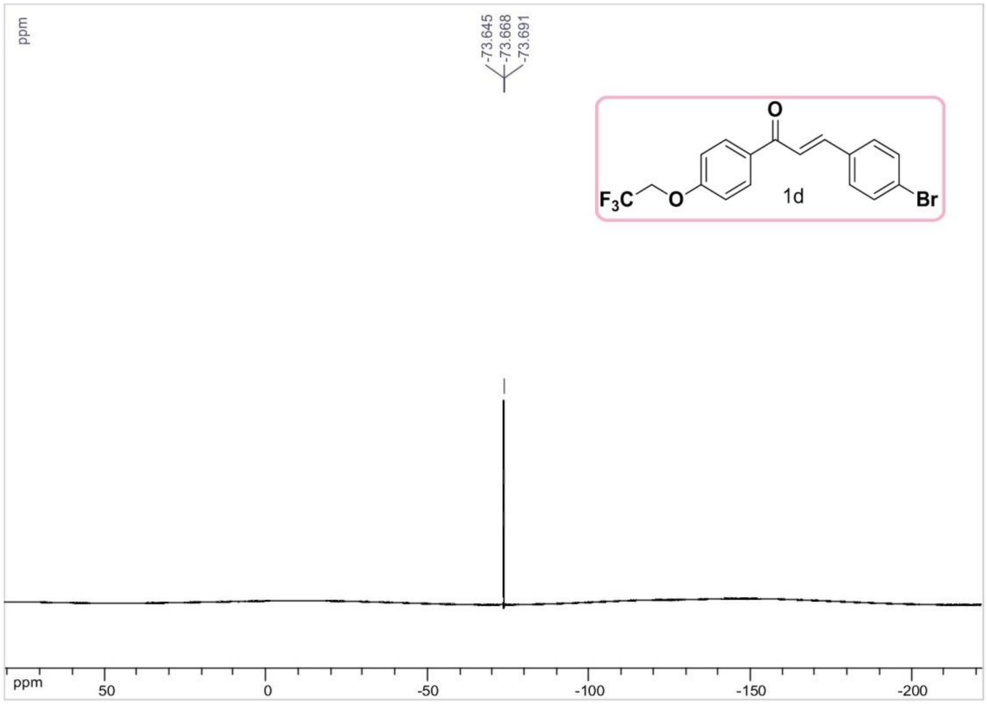
ppm
-73.645
-73.668
-73.691
O
F3C
O
1d
Br
ppm
50
0
-50
-100
-150
-200

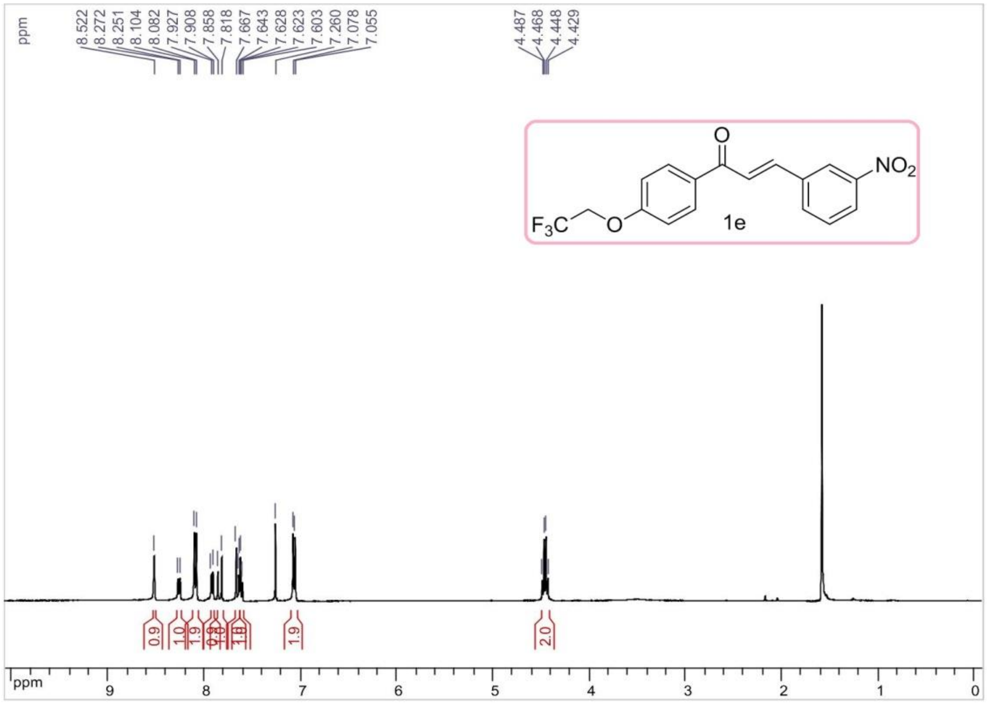
ppm
8.522
8.272
8.251
8.104
8.082
7.927
7.908
7.858
7.818
7.667
7.643
7.628
7.623
7.603
7.260
7.078
7.055
4.487
4.468
4.448
4.429
O
NO_2
F_3C
O
1e
0.9
1.0
1.9
0.9
1.8
1.9
2.0
ppm
9
8
7
6
5
4
3
2
1
0

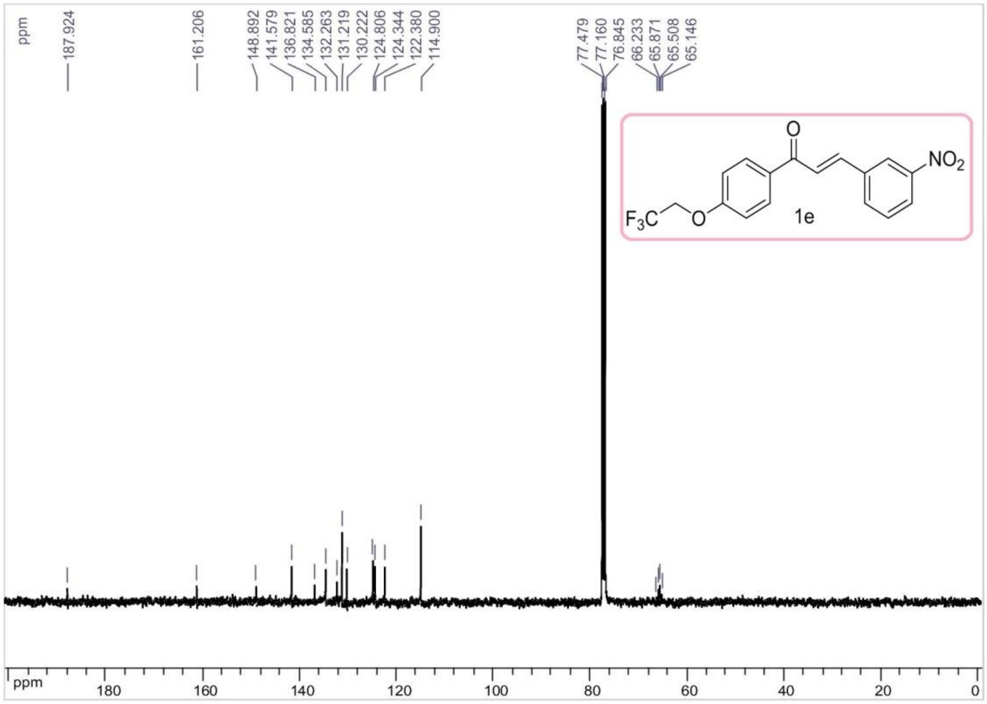
ppm
187.924
161.206
148.892
141.579
136.821
134.585
132.263
131.219
130.222
124.806
124.344
122.380
114.900
77.479
77.160
76.845
66.233
65.871
65.508
65.146
O
NO_2
F_3C
O
1e
ppm
180
160
140
120
100
80
60
40
20
0

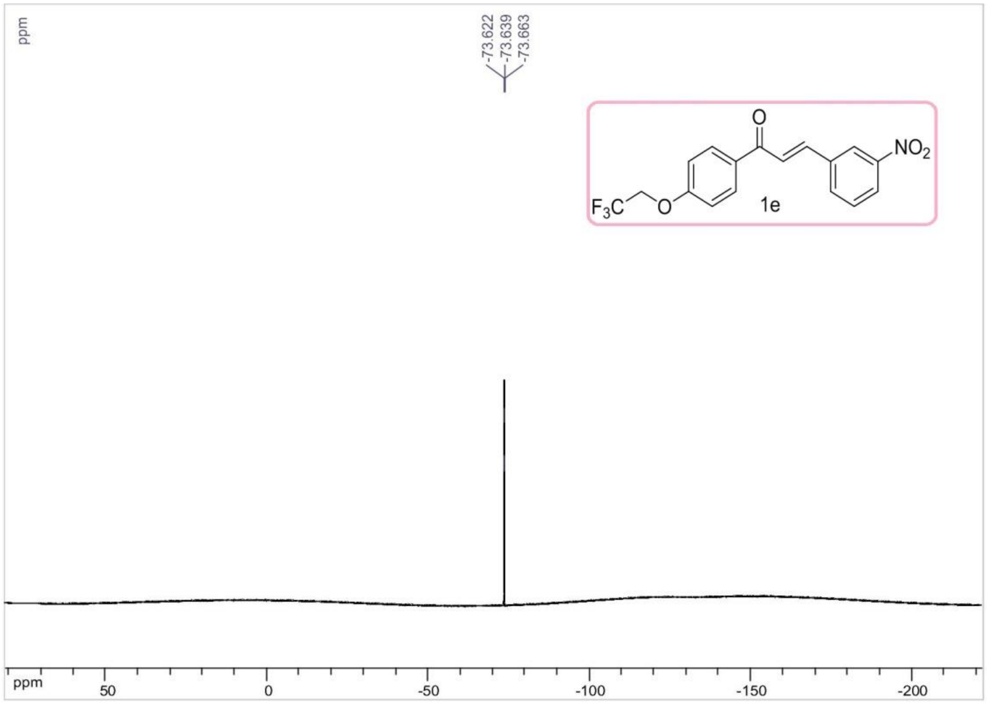

ppm
-73.622
-73.639
-73.663
O
NO2
F3C
O
1e
ppm
50
0
-50
-100
-150
-200

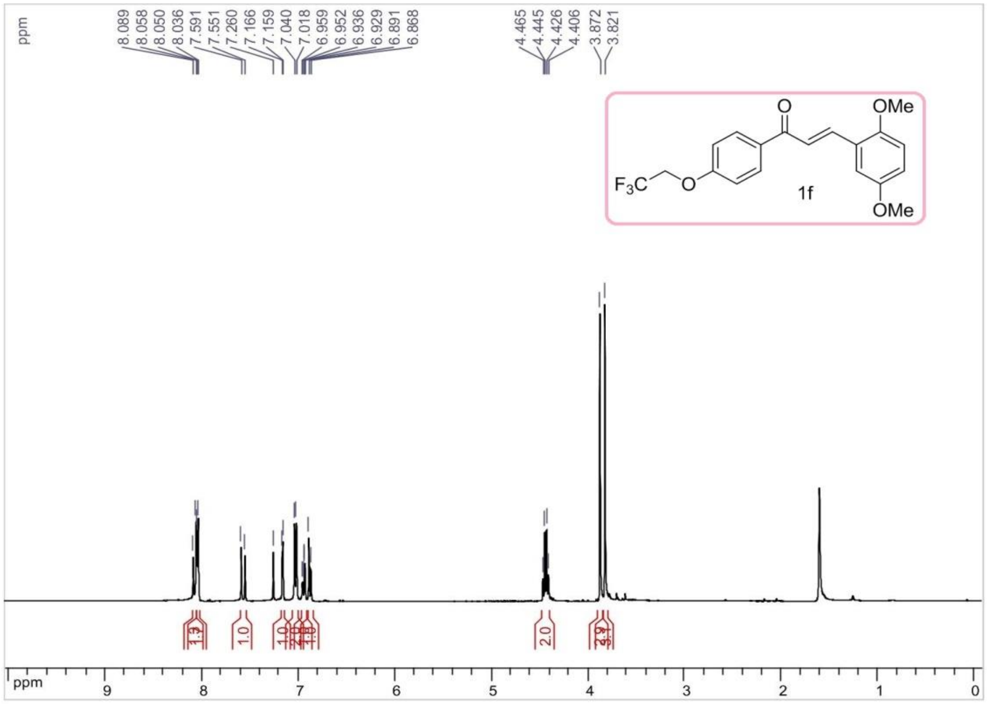

ppm
8.089
8.058
8.050
8.036
7.591
7.551
7.260
7.166
7.159
7.040
7.018
6.959
6.952
6.936
6.929
6.891
6.868
4.465
4.445
4.426
4.406
3.872
3.821
O
OMe
F3C
O
1f
OMe
2.0
1.0
ppm
9
8
7
6
5
4
3
2
1
0

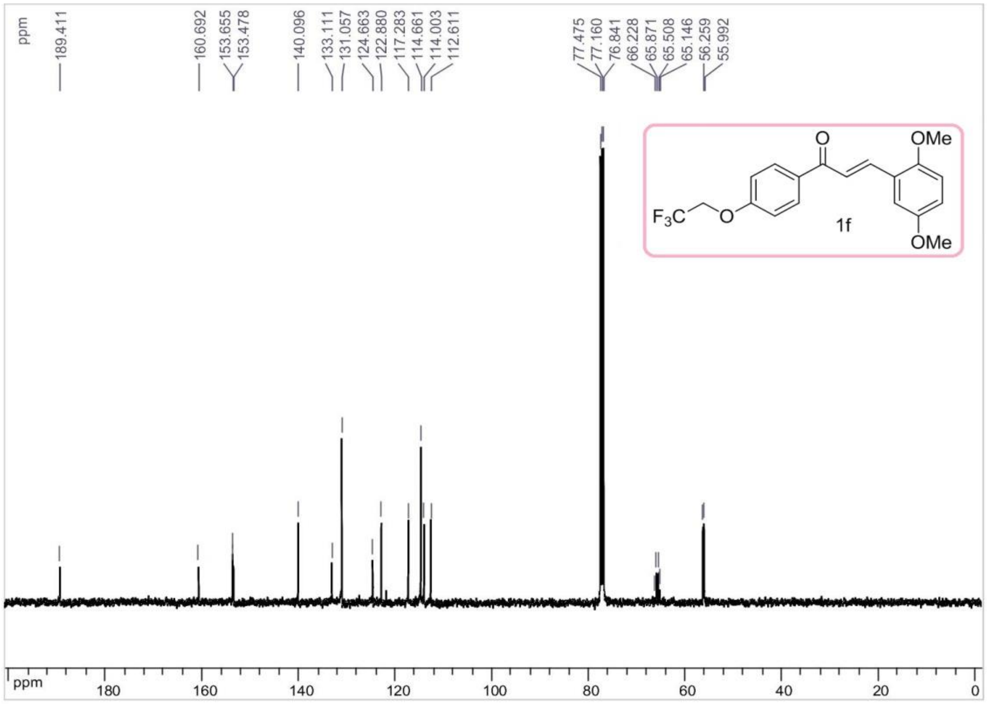
ppm
189.411
160.692
153.655
153.478
140.096
133.111
131.057
124.663
122.880
117.283
114.661
114.003
112.611
77.475
77.160
76.841
66.228
65.871
65.508
65.146
56.259
55.992
O
OMe
F_3C
O
1f
OMe
ppm
180
160
140
120
100
80
60
40
20
0

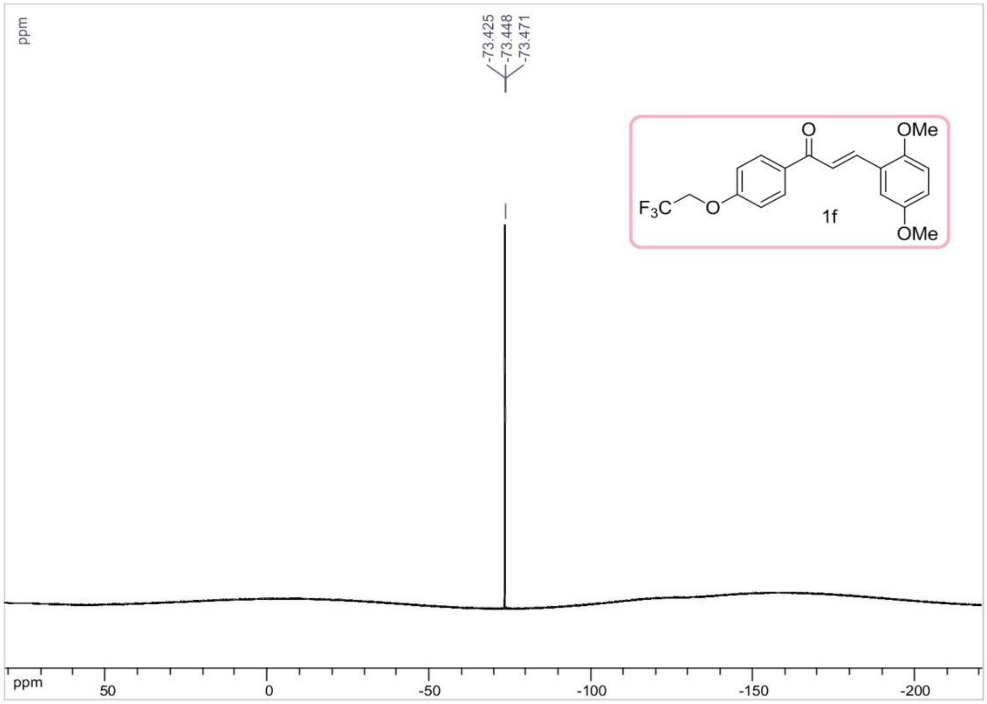
ppm
-73.425
-73.448
-73.471
O
OMe
F_3C
O
1f
OMe
ppm
50
0
-50
-100
-150
-200

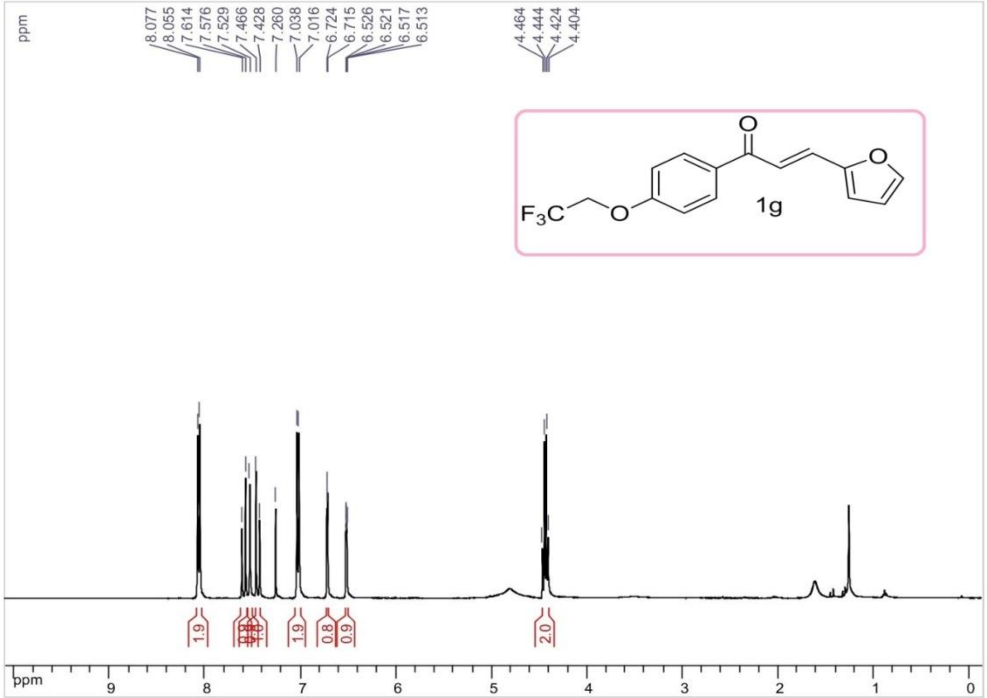
ppm
8.077
8.055
7.614
7.576
7.529
7.466
7.428
7.260
7.038
7.016
6.724
6.715
6.526
6.521
6.517
6.513
4.464
4.444
4.424
4.404
O
O
F3C
O
1g
1.9
1.9
0.8
0.9
2.0
ppm
9
8
7
6
5
4
3
2
1
0

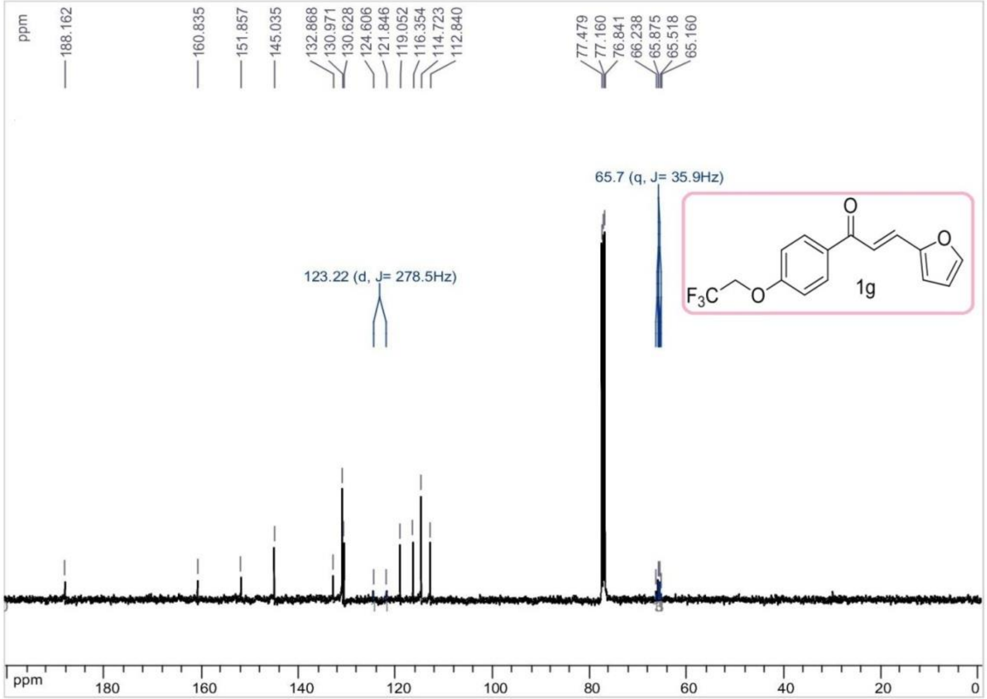
ppm
188.162
160.835
151.857
145.035
132.868
130.971
130.628
124.606
121.846
119.052
116.354
114.723
112.840
77.479
77.160
76.841
66.238
65.875
65.518
65.160
65.7 (q, J= 35.9Hz)
123.22 (d, J= 278.5Hz)
O
O
F3C
O
1g
ppm
180
160
140
120
100
80
60
40
20
0

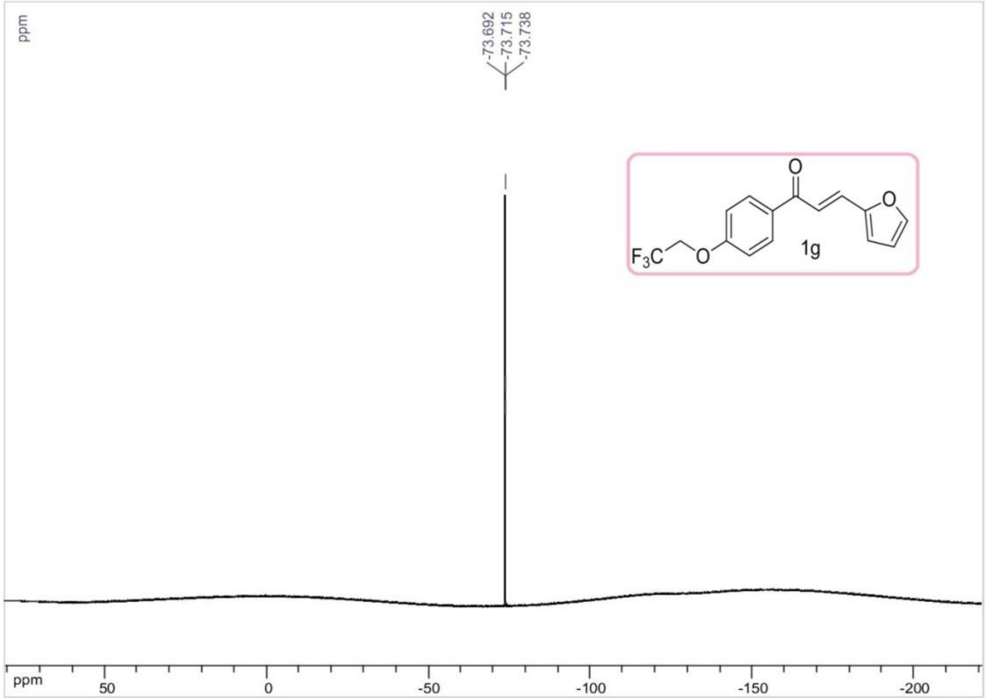
ppm
-73.692
-73.715
-73.738
O
O
F3C
O
1g
ppm 50 0 -50 -100 -150 -200

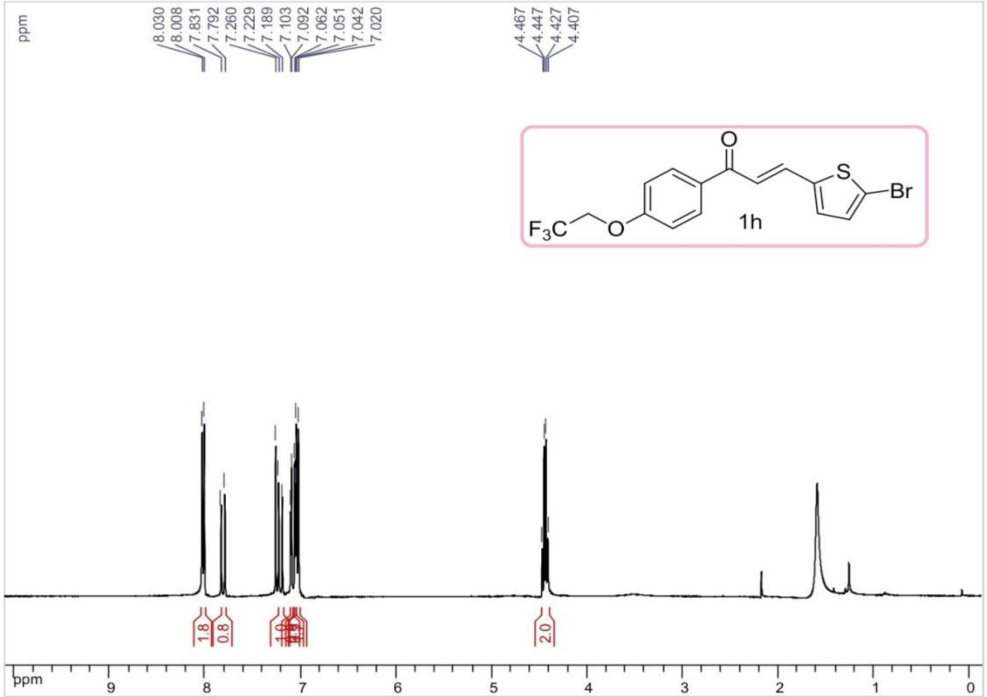
ppm
8.030
8.008
7.831
7.792
7.260
7.229
7.189
7.103
7.092
7.062
7.051
7.042
7.020
4.467
4.447
4.427
4.407
O
S
Br
F3C
O
1h
1.8
0.8
1.0
2.0
ppm 9 8 7 6 5 4 3 2 1 0

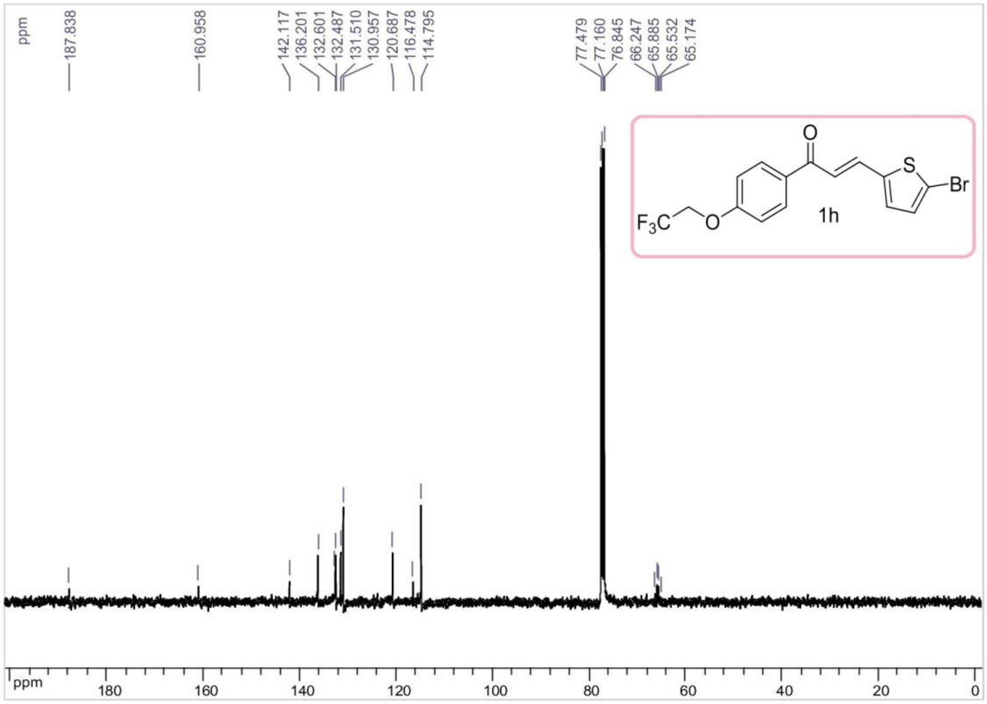
ppm
187.838
160.958
142.117
136.201
132.601
132.487
131.510
130.957
120.687
116.478
114.795
77.479
77.160
76.845
66.247
65.885
65.532
65.174
O
S
Br
F_3C
O
1h
ppm
180
160
140
120
100
80
60
40
20
0

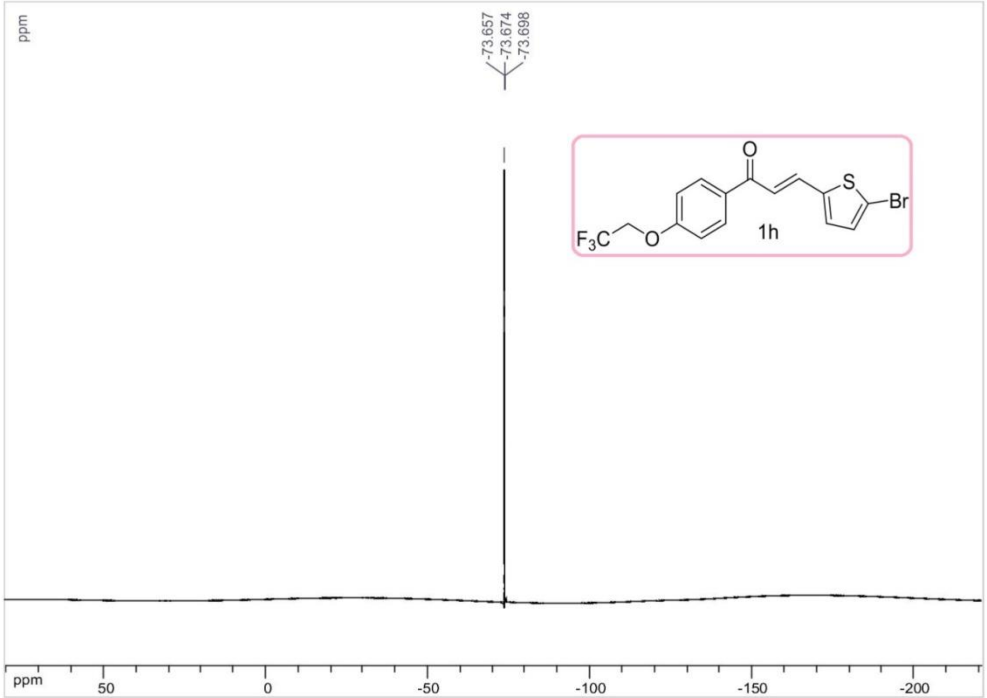
ppm
-73.657
-73.674
-73.698
O
S
Br
F_3C
O
1h
ppm
50
0
-50
-100
-150
-200

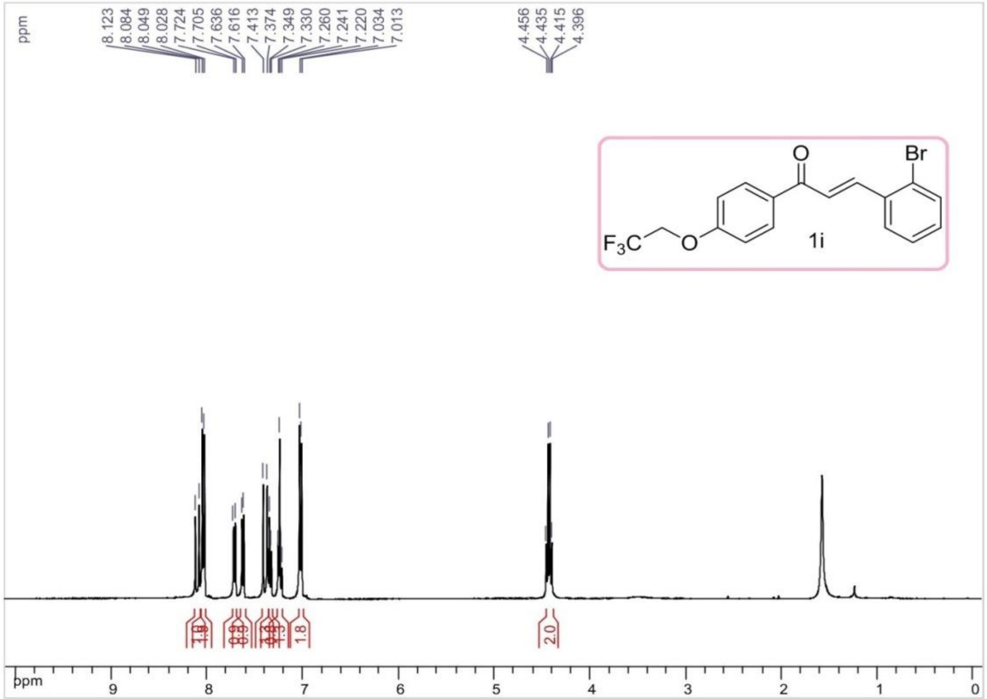
ppm
8.123
8.084
8.049
8.028
7.724
7.705
7.636
7.616
7.413
7.374
7.349
7.330
7.260
7.241
7.220
7.034
7.013
4.456
4.435
4.415
4.396
O
Br
F3C
O
1i
1.8
2.0
ppm
9
8
7
6
5
4
3
2
1
0

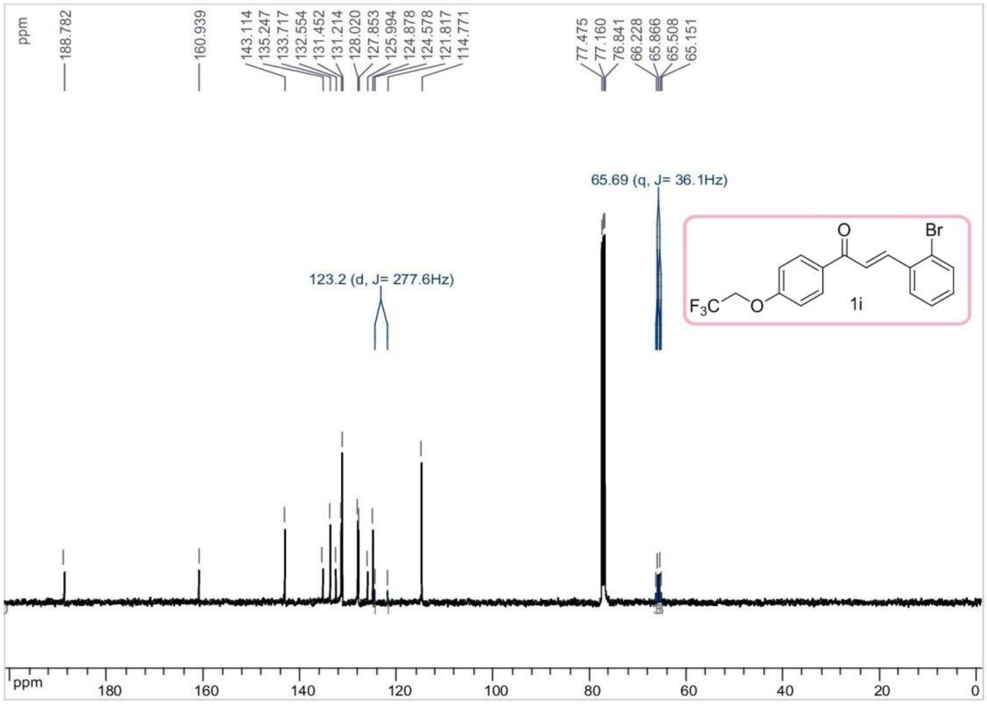
ppm
188.782
160.939
143.114
135.247
133.717
132.554
131.452
131.214
128.020
127.853
125.994
124.878
124.578
121.817
114.771
77.475
77.160
76.841
66.228
65.866
65.508
65.151
65.69 (q, J= 36.1Hz)
123.2 (d, J= 277.6Hz)
O
Br
F3C
O
1i
ppm
180
160
140
120
100
80
60
40
20
0

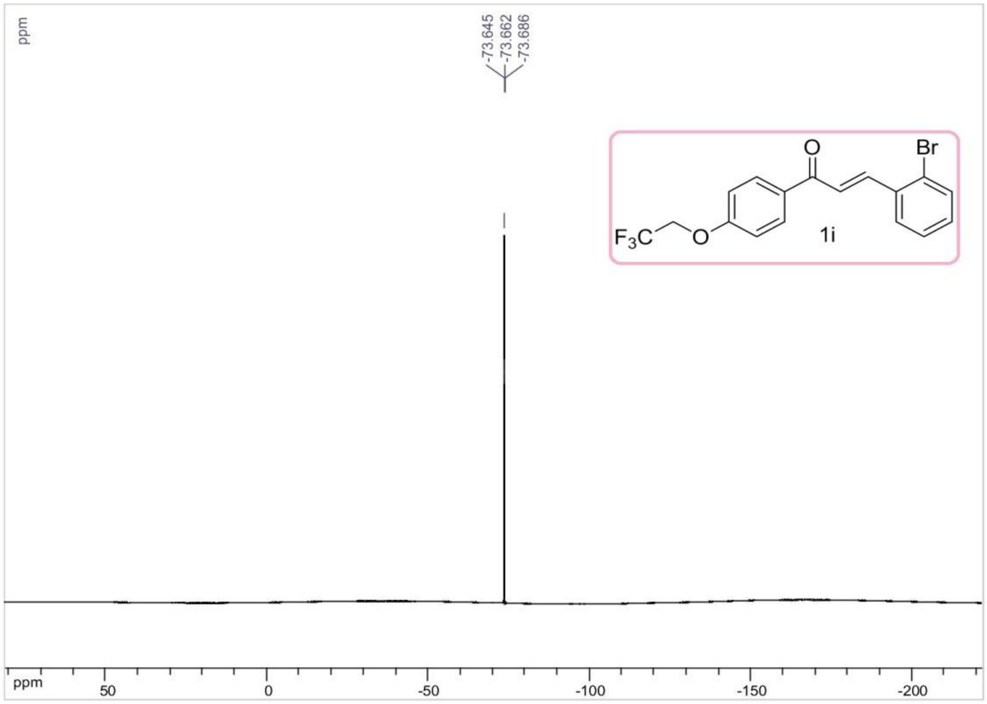

-73.645
-73.662
-73.686
1i
ppm
50
0
-50
-100
-150
-200

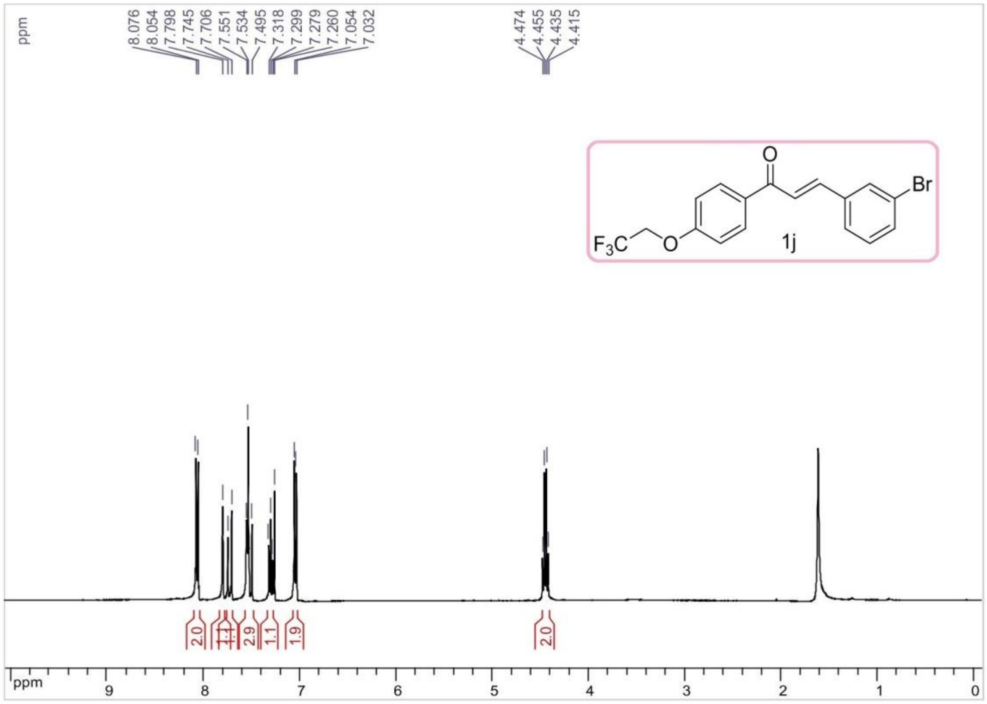

8.076
8.054
7.798
7.745
7.706
7.551
7.534
7.495
7.318
7.299
7.279
7.260
7.054
7.032
4.474
4.455
4.435
4.415
1j
2.0
2.9
1.1
1.9
2.0
ppm
9
8
7
6
5
4
3
2
1
0

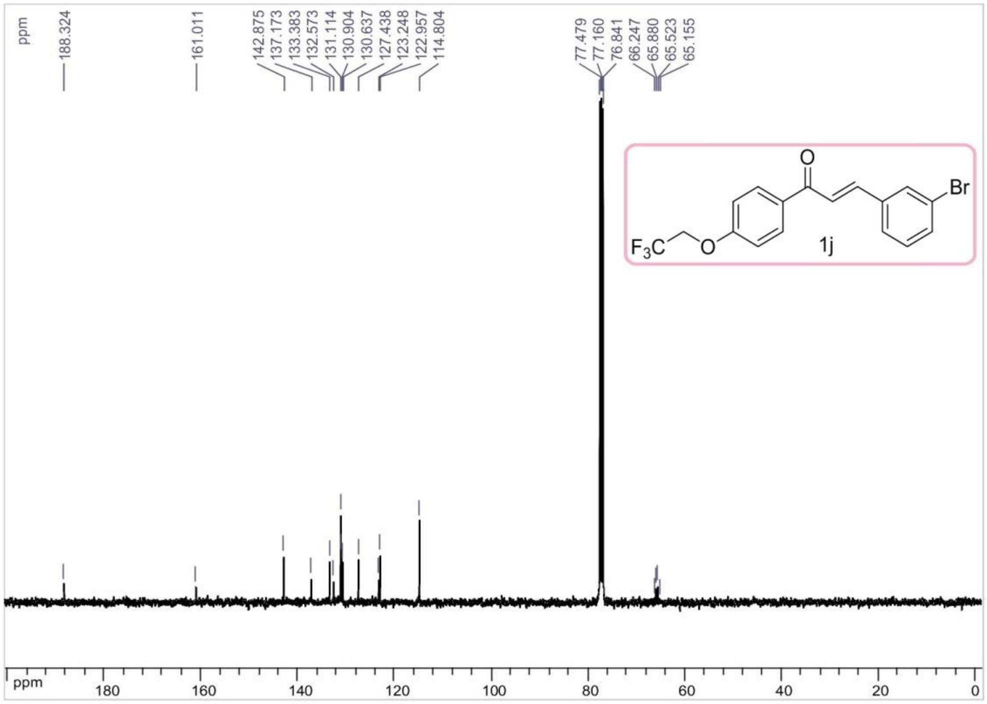
ppm
188.324
161.011
142.875
137.173
133.383
132.573
131.114
130.904
130.637
127.438
123.248
122.957
114.804
77.479
77.160
76.841
66.247
65.880
65.523
65.155
O
Br
F3C
O
1j
ppm
180
160
140
120
100
80
60
40
20
0

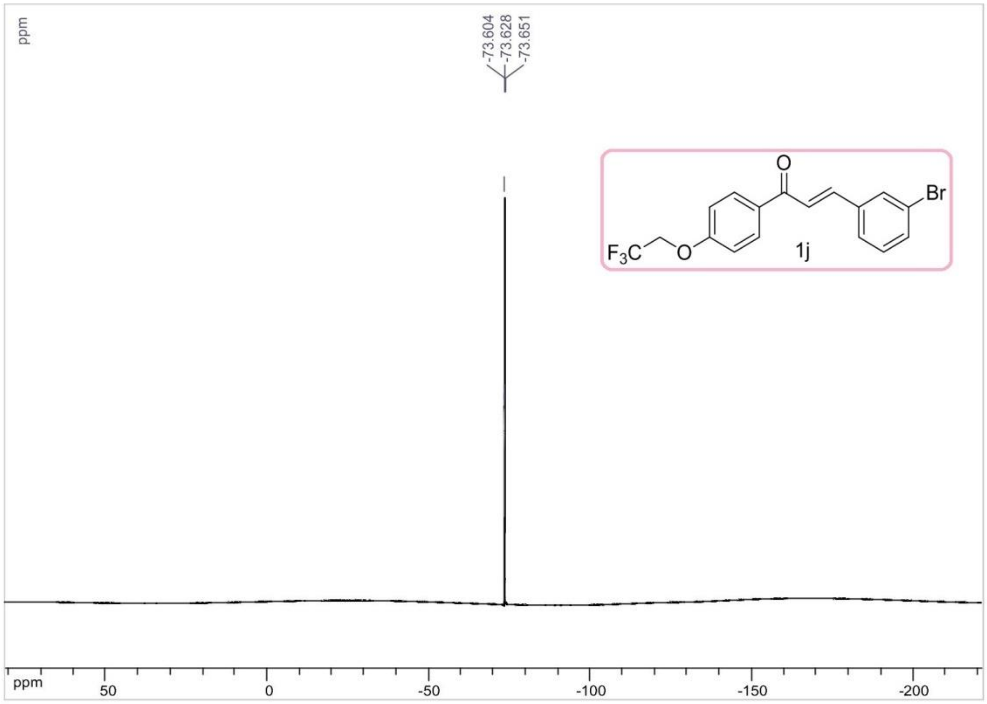
ppm
-73.604
-73.628
-73.651
O
Br
F3C
O
1j
ppm
50
0
-50
-100
-150
-200

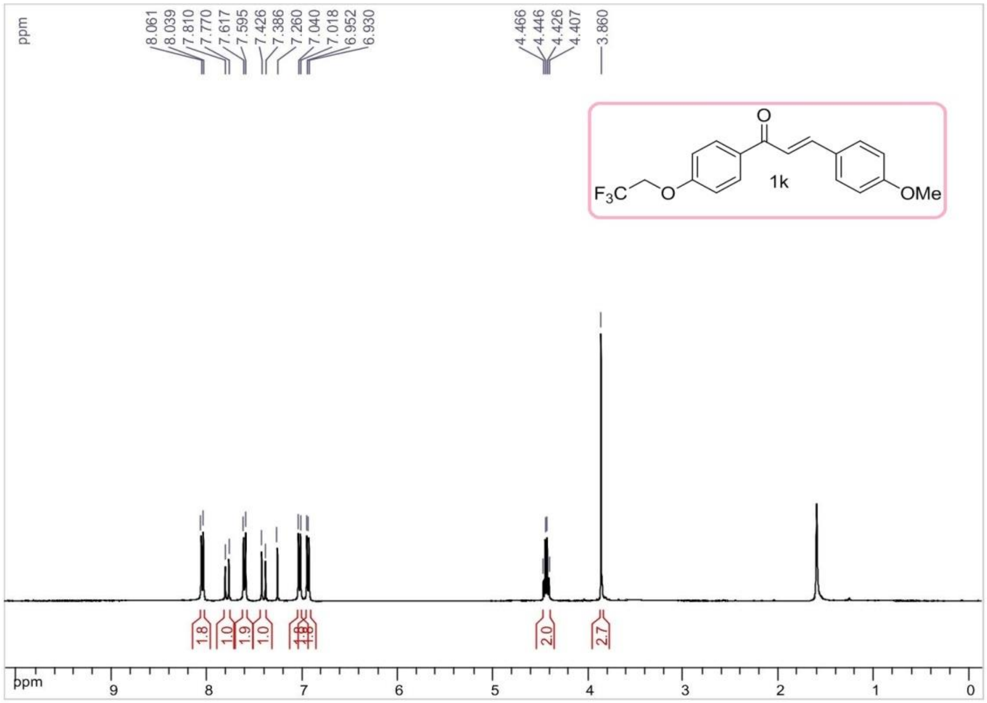

ppm
8.061
8.039
7.810
7.770
7.617
7.595
7.426
7.386
7.260
7.040
7.018
6.952
6.930
4.466
4.446
4.426
4.407
3.860
O
1k
F3C
O
OMe
1.8
1.0
1.9
1.0
2.0
2.7
ppm
9
8
7
6
5
4
3
2
1
0

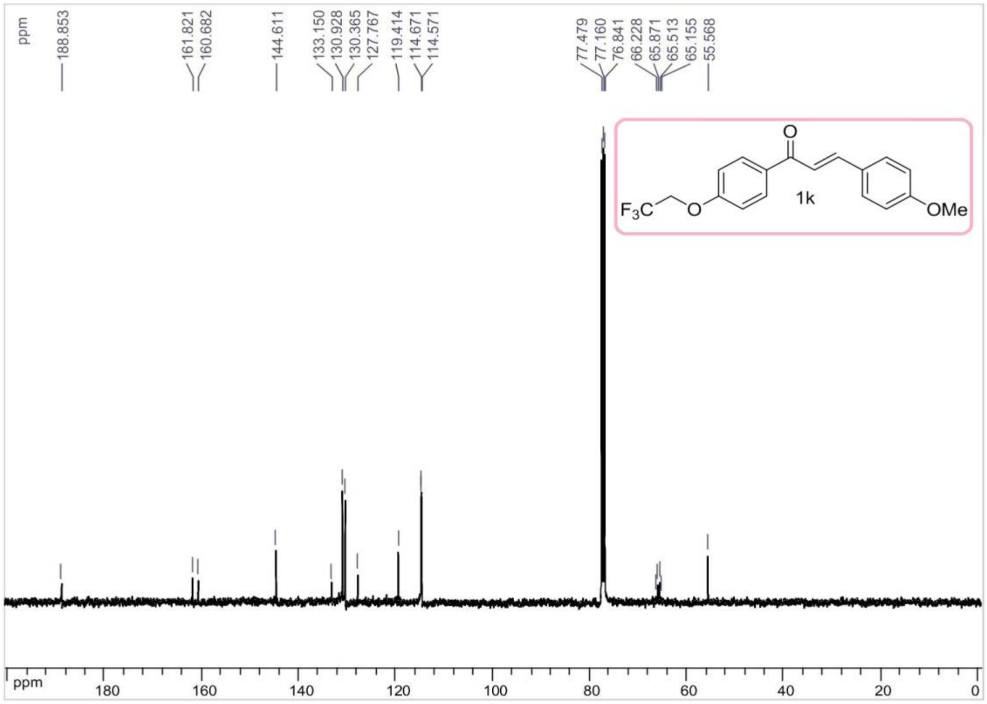

ppm
188.853
161.821
160.682
144.611
133.150
130.928
130.365
127.767
119.414
114.671
114.571
77.479
77.160
76.841
66.228
65.871
65.513
65.155
55.568
O
1k
F3C
O
OMe
ppm
180
160
140
120
100
80
60
40
20
0

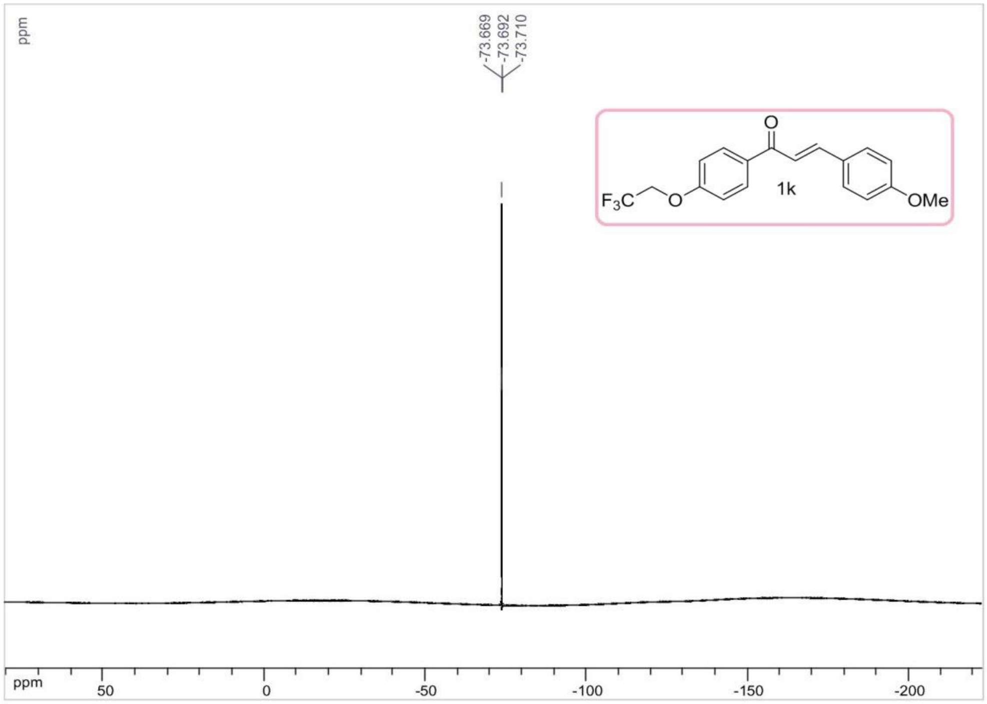
ppm
-73.669
-73.692
-73.710
O
F3C
O
1k
OMe
ppm
50
0
-50
-100
-150
-200

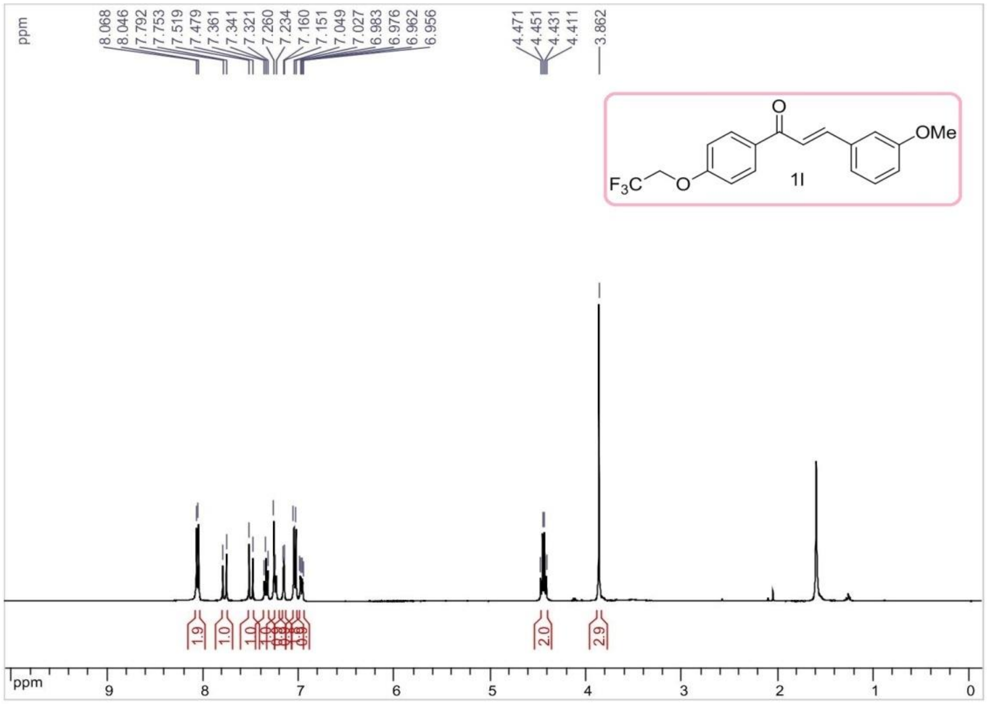
ppm
8.068
8.046
7.792
7.753
7.519
7.479
7.361
7.341
7.321
7.260
7.234
7.160
7.151
7.049
7.027
6.983
6.976
6.962
6.956
4.471
4.451
4.431
4.411
3.862
O
OMe
F3C
O
1l
1.9
1.0
1.0
2.0
2.9
ppm
9
8
7
6
5
4
3
2
1
0

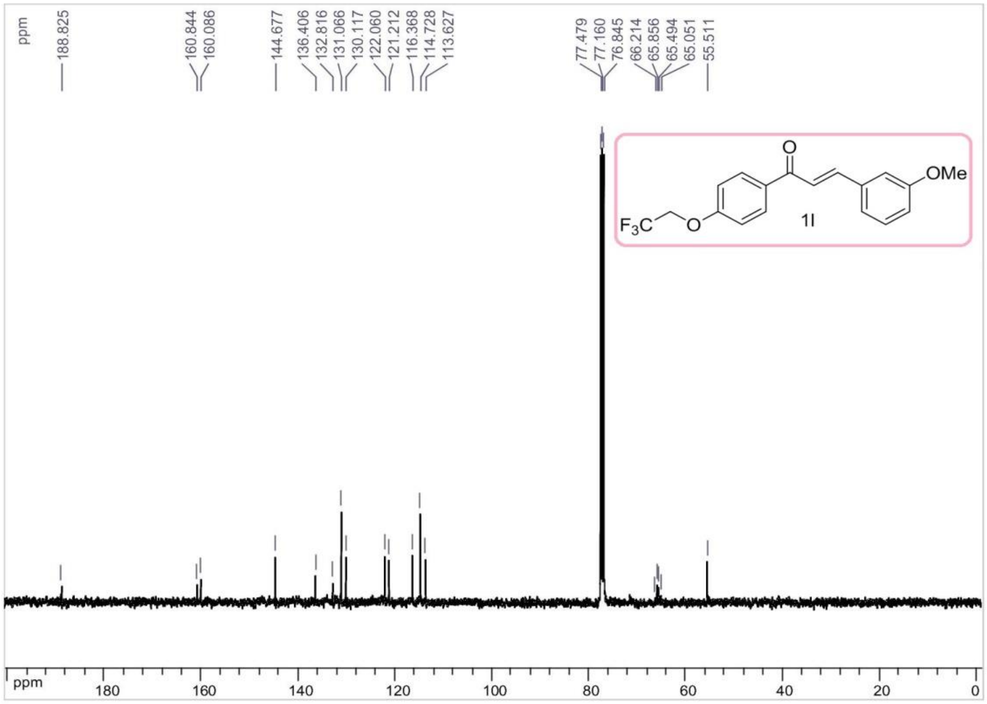
ppm
188.825
160.844
160.086
144.677
136.406
132.816
131.066
130.117
122.060
121.212
116.368
114.728
113.627
77.479
77.160
76.845
66.214
65.856
65.494
65.051
55.511
O
OMe
F_3C
O
1l
ppm
180
160
140
120
100
80
60
40
20
0

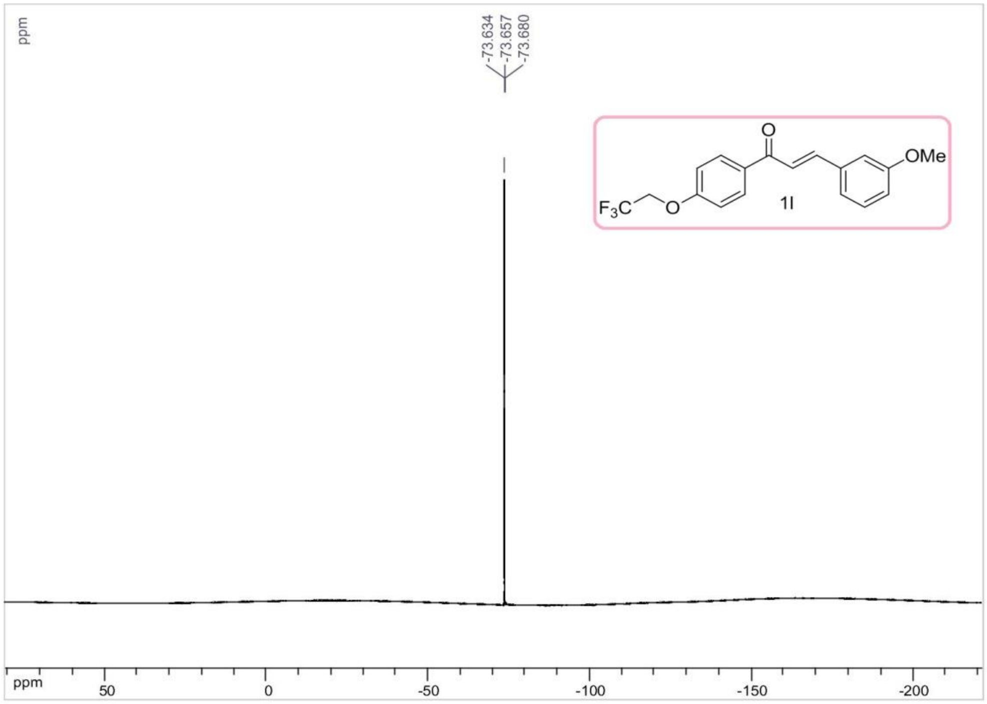
ppm
-73.634
-73.657
-73.680
O
OMe
F_3C
O
1l
ppm
50
0
-50
-100
-150
-200

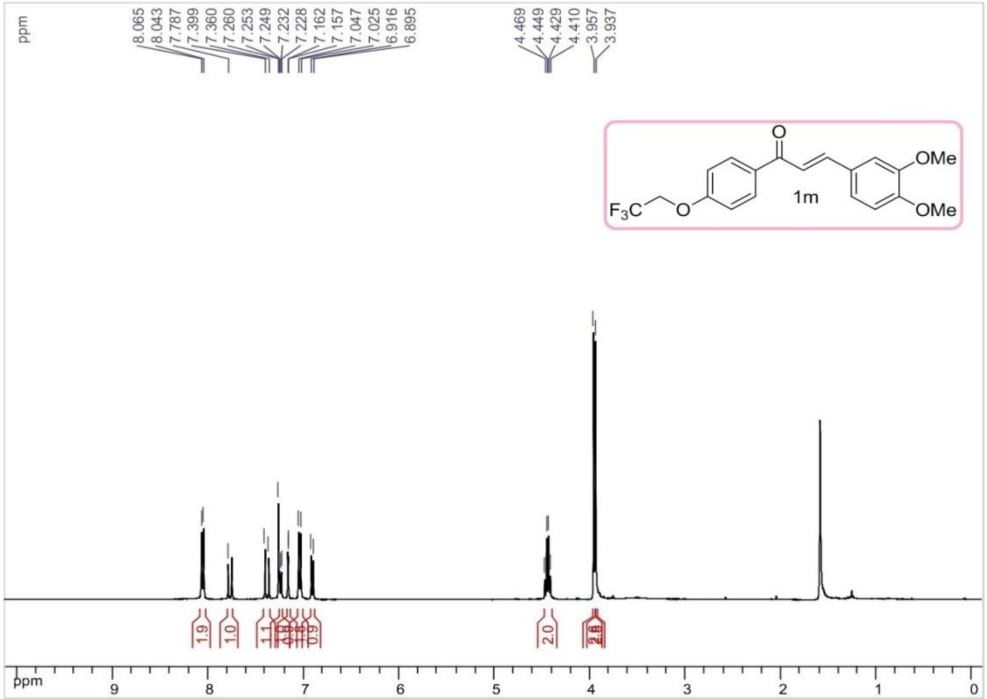
ppm
8.065
8.043
7.787
7.399
7.360
7.260
7.253
7.249
7.232
7.228
7.162
7.157
7.047
7.025
6.916
6.895
4.469
4.449
4.429
4.410
3.957
3.937
OMe
OMe
F3C
O
1m
1.9
1.0
2.0
ppm
9
8
7
6
5
4
3
2
1
0

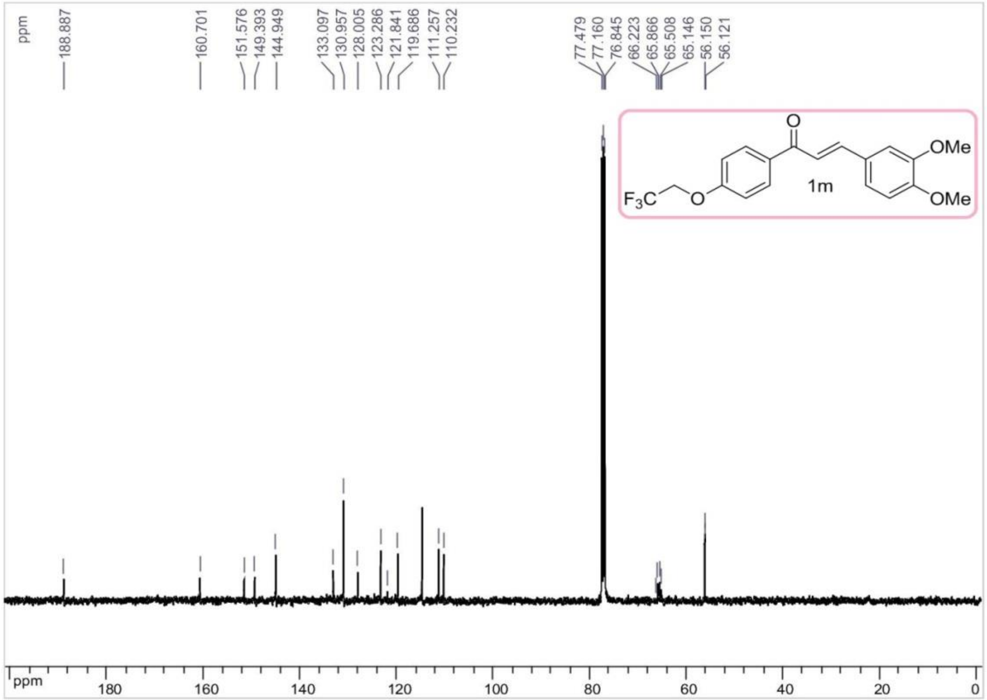
ppm
188.887
160.701
151.576
149.393
144.949
133.097
130.957
128.005
123.286
121.841
119.686
111.257
110.232
77.479
77.160
76.845
66.223
65.866
65.508
65.146
56.150
56.121
OMe
OMe
F3C
O
1m
ppm
180
160
140
120
100
80
60
40
20
0

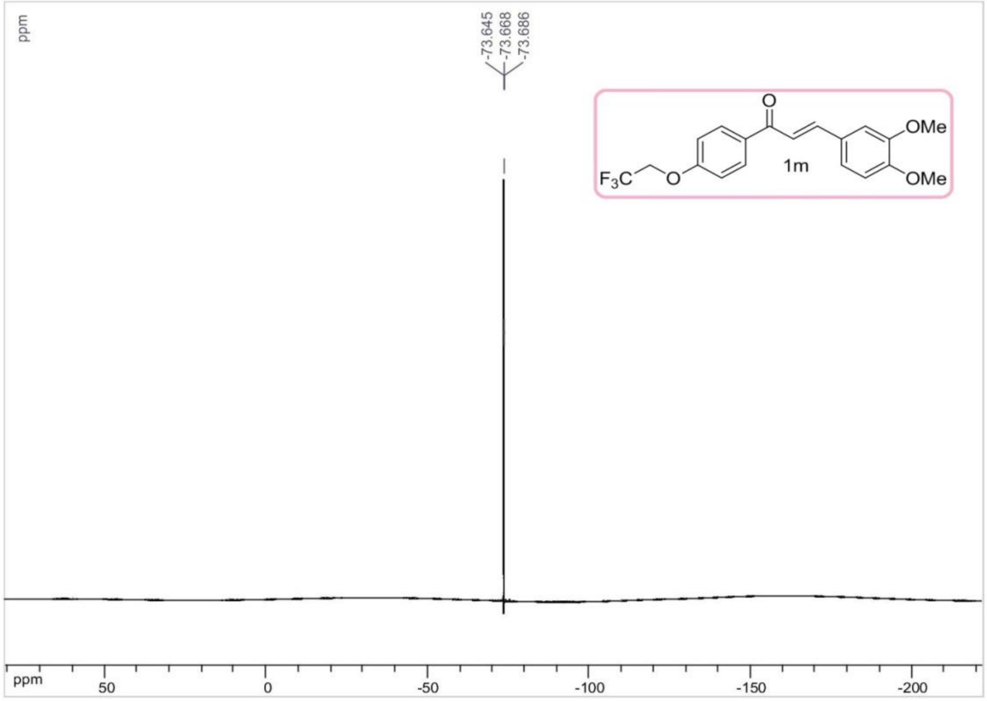
ppm
-73.645
-73.668
-73.686
F3C
O
OMe
OMe
1m
ppm
50
0
-50
-100
-150
-200

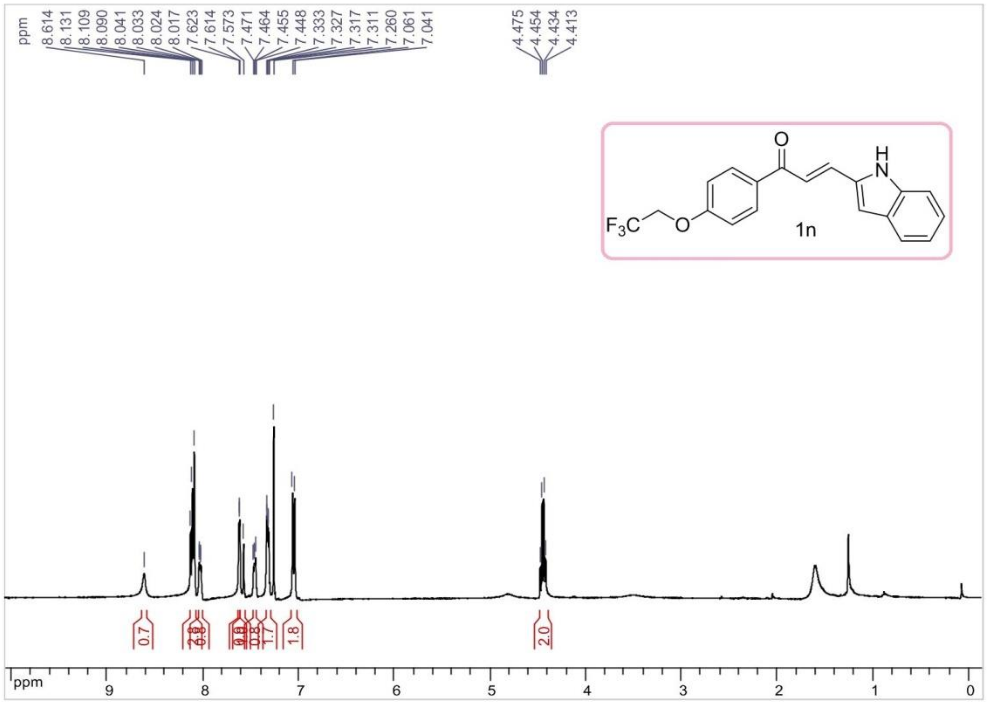
ppm
8.614
8.131
8.109
8.090
8.041
8.033
8.024
8.017
7.623
7.614
7.573
7.471
7.464
7.455
7.448
7.333
7.327
7.317
7.311
7.260
7.061
7.041
4.475
4.454
4.434
4.413
O
H
N
F3C
O
1n
0.7
1.8
2.0
ppm
9
8
7
6
5
4
3
2
1
0

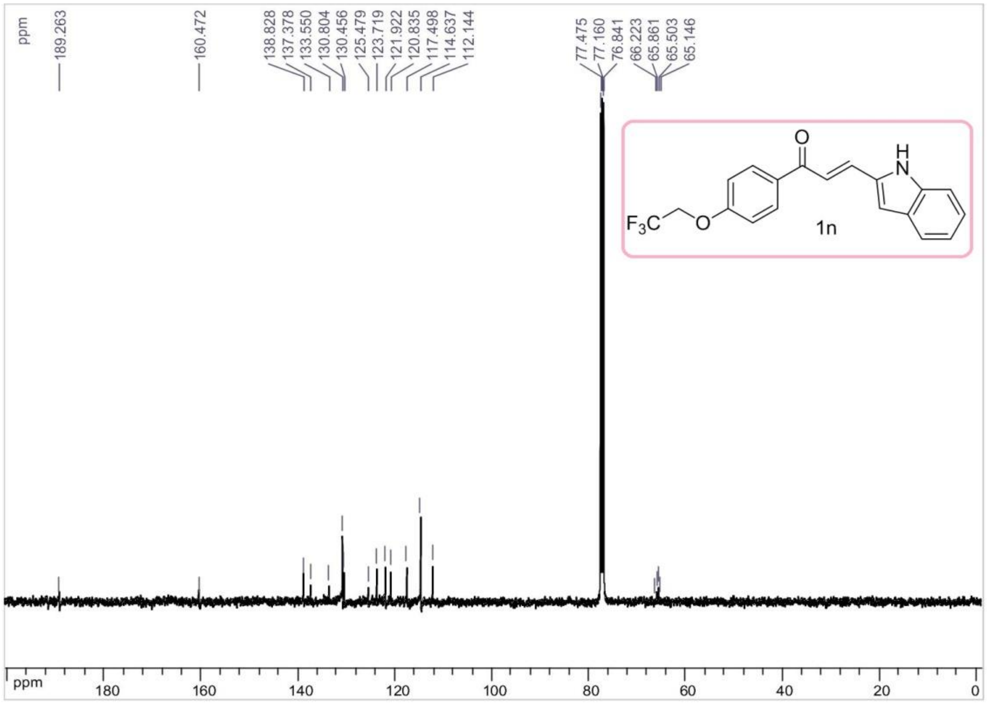
ppm
189.263
160.472
138.828
137.378
133.550
130.804
130.456
125.479
123.719
121.922
120.835
117.498
114.637
112.144
77.475
77.160
76.841
66.223
65.861
65.503
65.146
O
H
N
F_3C
O
1n
ppm
180
160
140
120
100
80
60
40
20
0

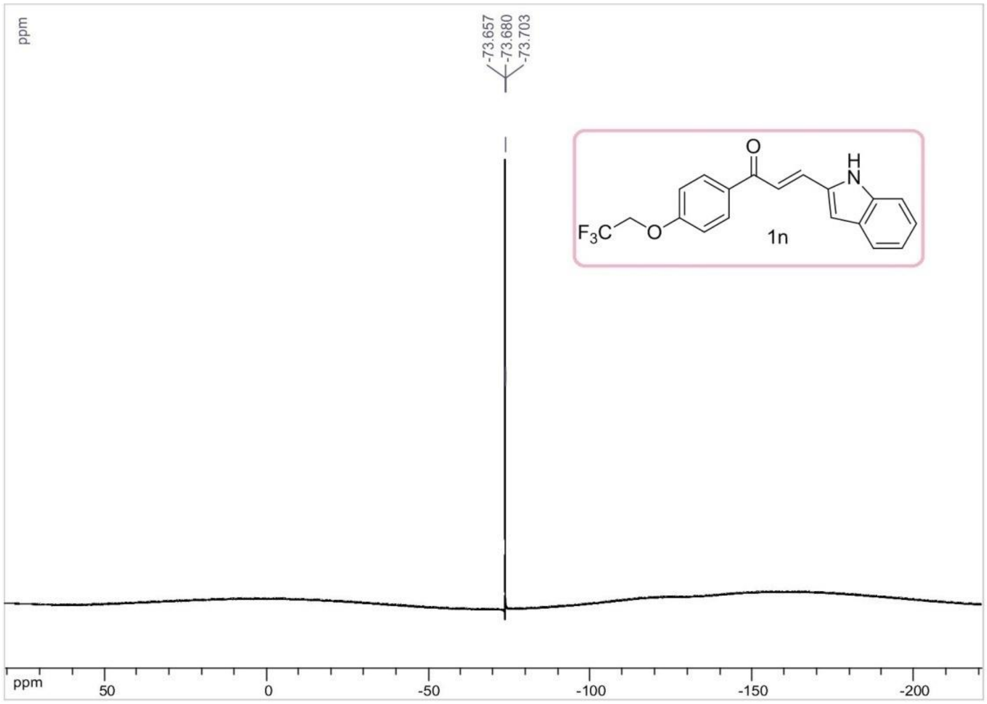
ppm
-73.657
-73.680
-73.703
O
H
N
F_3C
O
1n
ppm
50
0
-50
-100
-150
-200

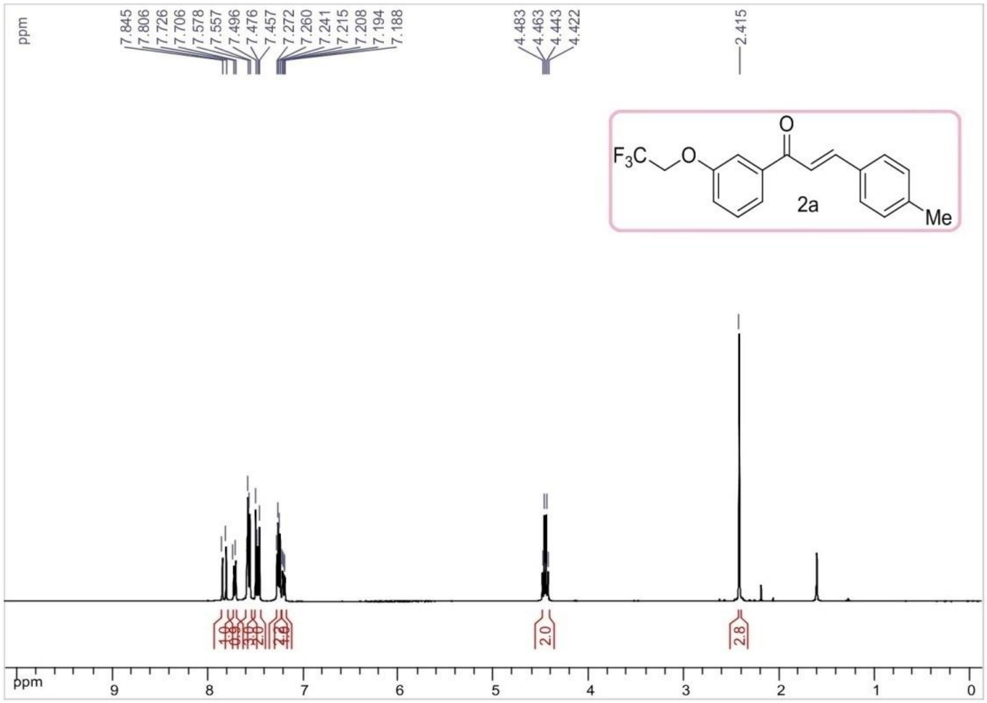
ppm
7.845
7.806
7.726
7.706
7.578
7.557
7.496
7.476
7.457
7.272
7.260
7.241
7.215
7.208
7.194
7.188
4.483
4.463
4.443
4.422
2.415
O
F3C
O
2a
Me
2.0
2.8
ppm
9
8
7
6
5
4
3
2
1
0

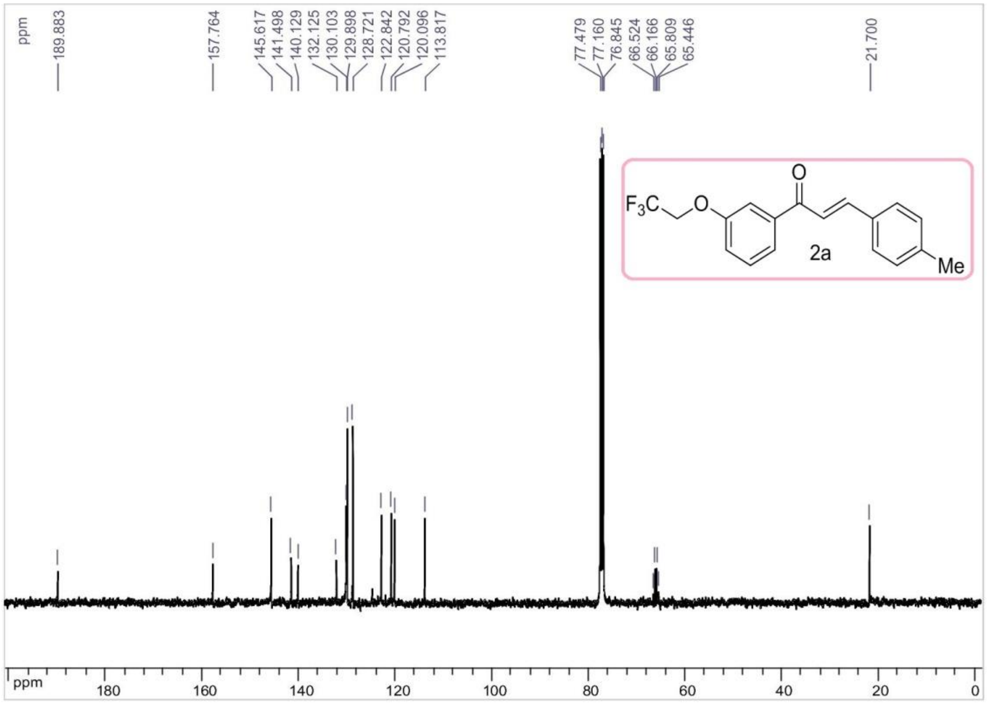
ppm
189.883
157.764
145.617
141.498
140.129
132.125
130.103
129.898
128.721
122.842
120.792
120.096
113.817
77.479
77.160
76.845
66.524
66.166
65.809
65.446
21.700
O
F3C
O
2a
Me
ppm
180
160
140
120
100
80
60
40
20
0

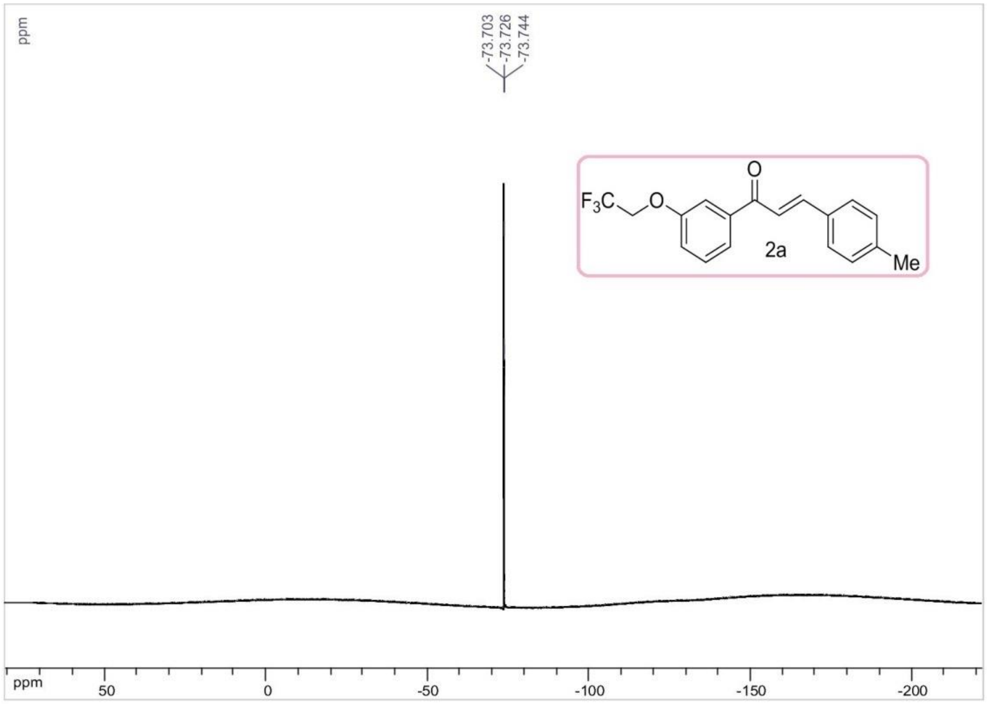

ppm
-73.703
-73.726
-73.744
F3C
O
O
2a
Me
ppm
50
0
-50
-100
-150
-200

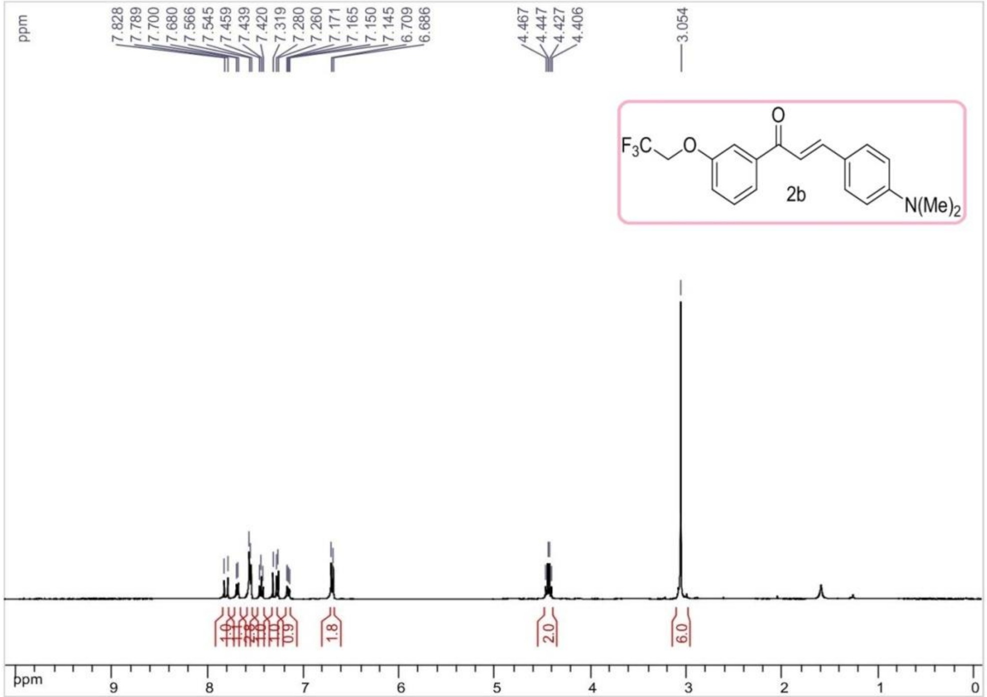

ppm
7.828
7.789
7.700
7.680
7.566
7.545
7.459
7.439
7.420
7.319
7.280
7.260
7.171
7.165
7.150
7.145
6.709
6.686
4.467
4.447
4.427
4.406
3.054
F3C
O
O
2b
N(Me)2
1.8
2.0
6.0
ppm
9
8
7
6
5
4
3
2
1
0

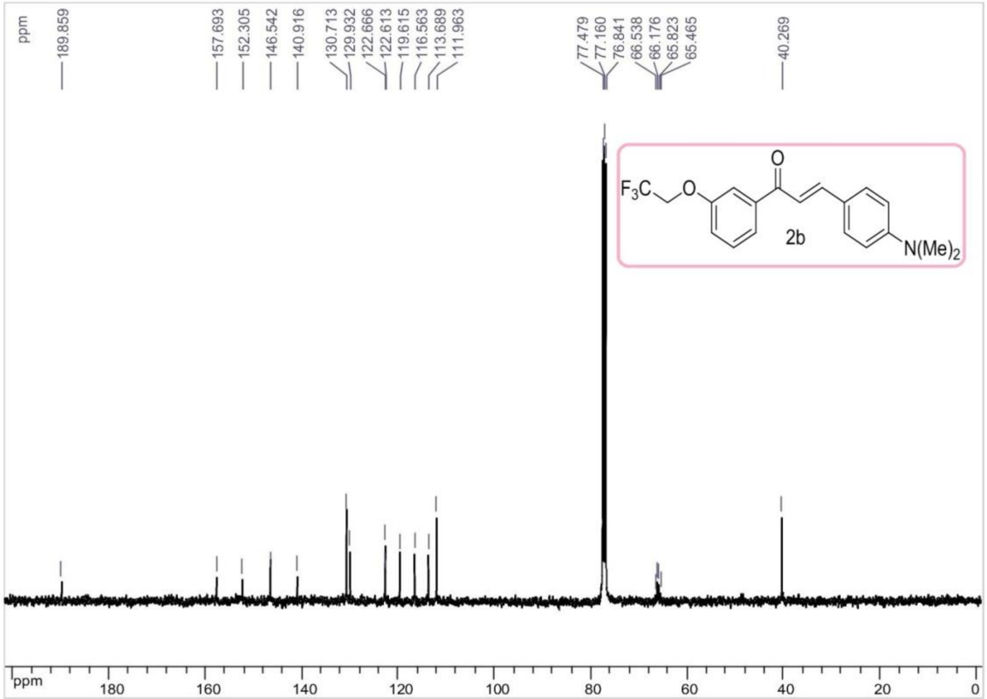
ppm
189.859
157.693
152.305
146.542
140.916
130.713
129.932
122.666
122.613
119.615
116.563
113.689
111.963
77.479
77.160
76.841
66.538
66.176
65.823
65.465
40.269
O
F3C
O
2b
N(Me)2
ppm
180
160
140
120
100
80
60
40
20
0

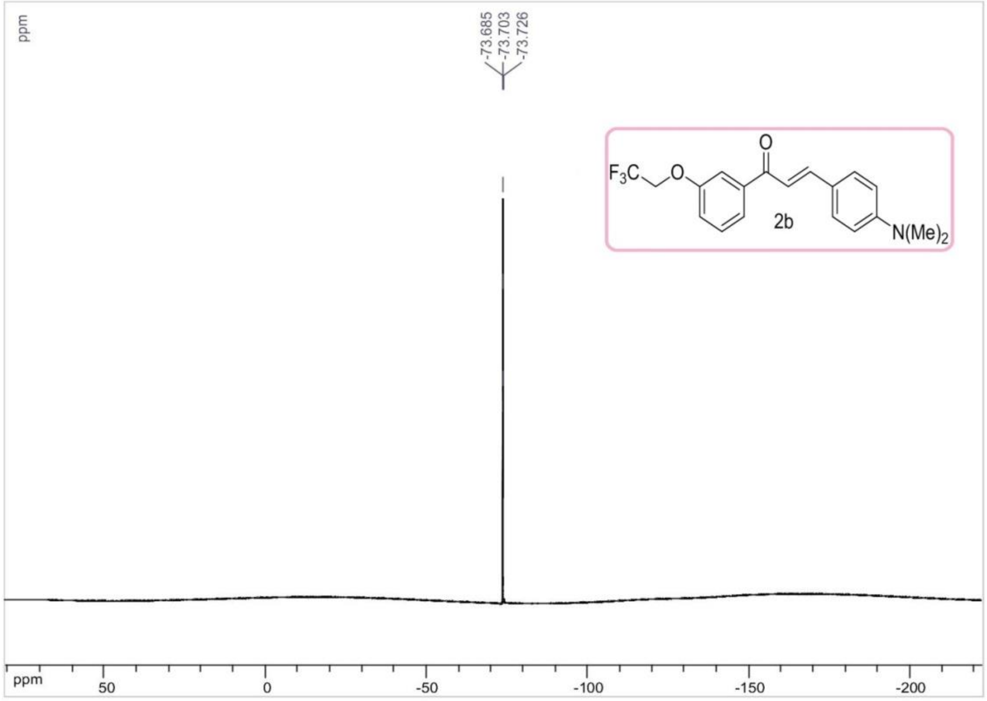
ppm
-73.685
-73.703
-73.726
O
F3C
O
2b
N(Me)2
ppm
50
0
-50
-100
-150
-200

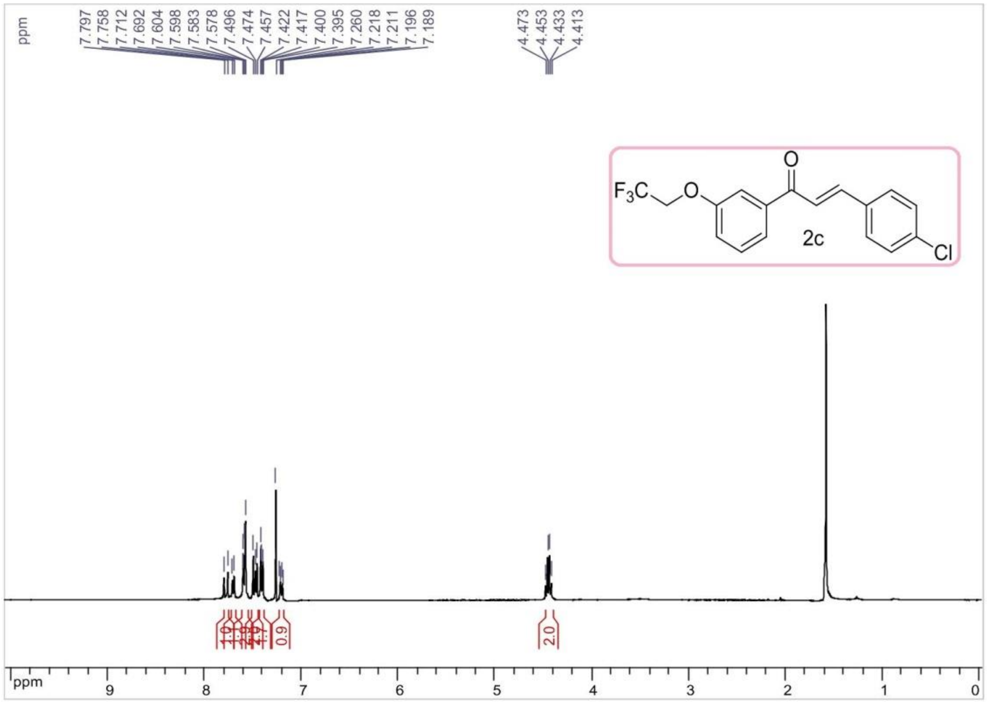
ppm
7.797
7.758
7.712
7.692
7.604
7.598
7.583
7.578
7.496
7.474
7.457
7.422
7.417
7.400
7.395
7.260
7.218
7.211
7.196
7.189
4.473
4.453
4.433
4.413
O
F3C
O
2c
Cl
2.0
0.9
ppm
9
8
7
6
5
4
3
2
1
0

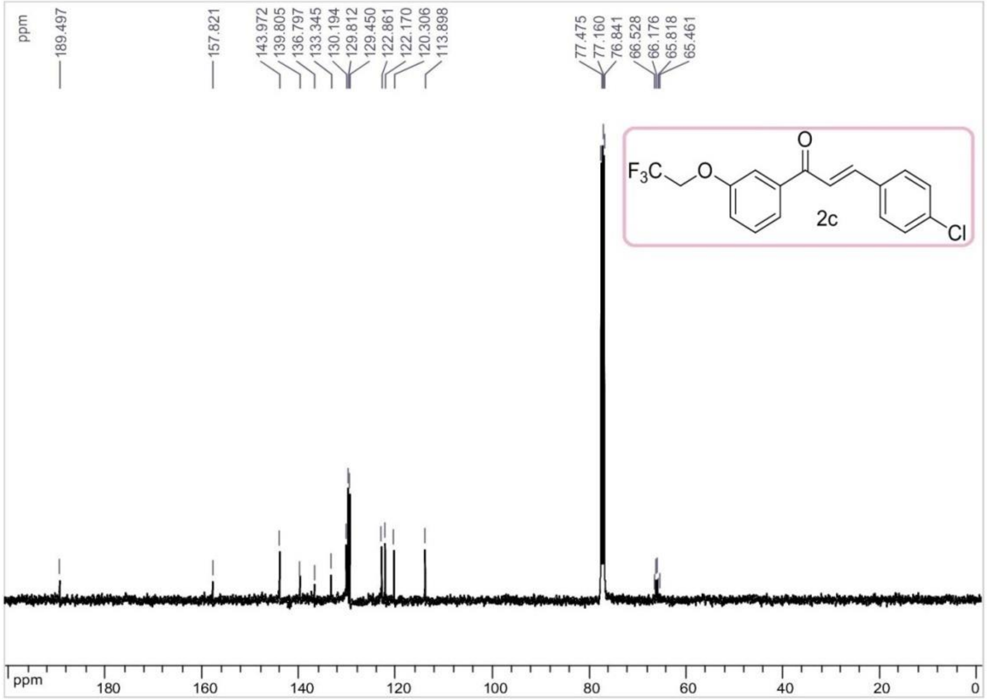
ppm
189.497
157.821
143.972
139.805
136.797
133.345
130.194
129.812
129.450
122.861
122.170
120.306
113.898
77.475
77.160
76.841
66.528
66.176
65.818
65.461
O
F3C
O
2c
Cl
ppm
180
160
140
120
100
80
60
40
20
0

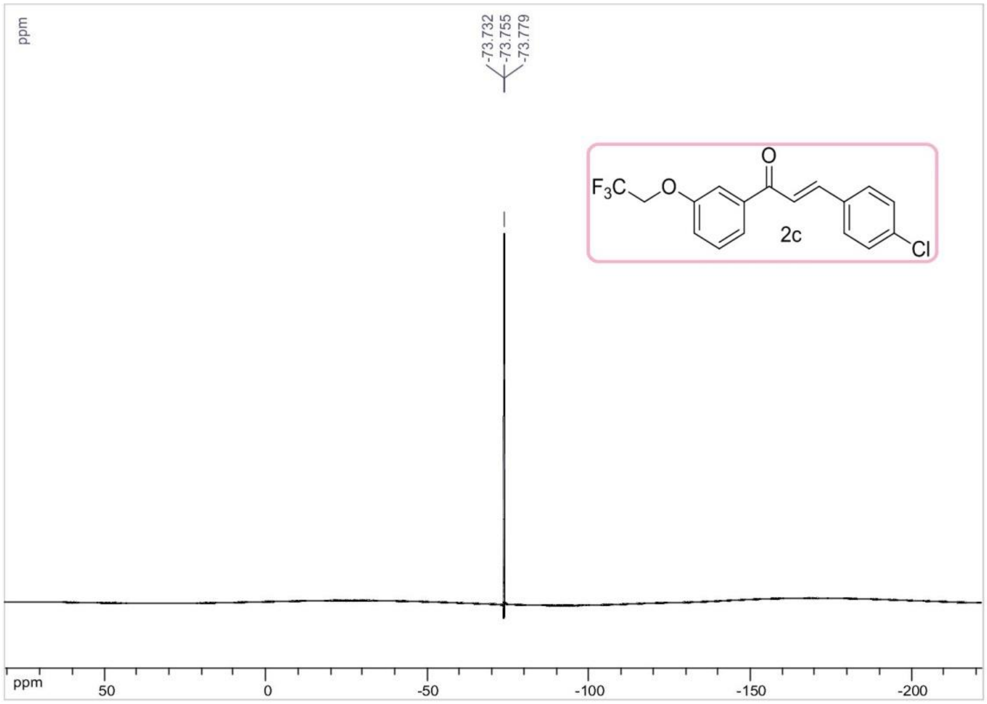
ppm
-73.732
-73.755
-73.779
F3C O O
2c
Cl
ppm
50
0
-50
-100
-150
-200

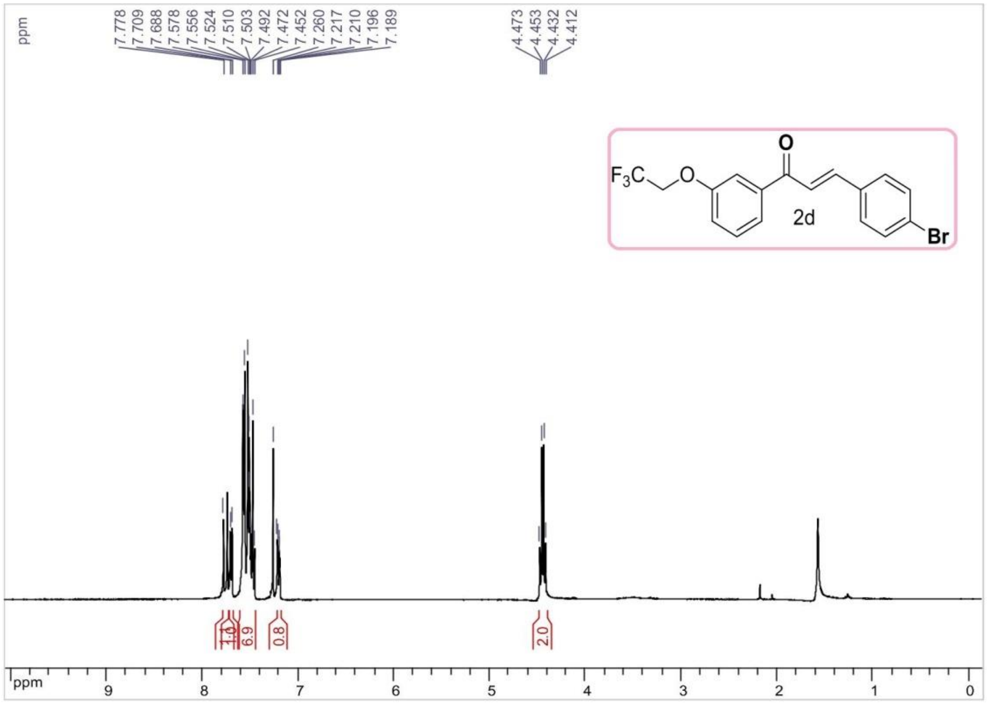
ppm
7.778
7.709
7.688
7.578
7.556
7.524
7.510
7.503
7.492
7.472
7.452
7.260
7.217
7.210
7.196
7.189
4.473
4.453
4.432
4.412
F3C O O
2d
Br
1.0
6.9
0.8
2.0
ppm
9
8
7
6
5
4
3
2
1
0

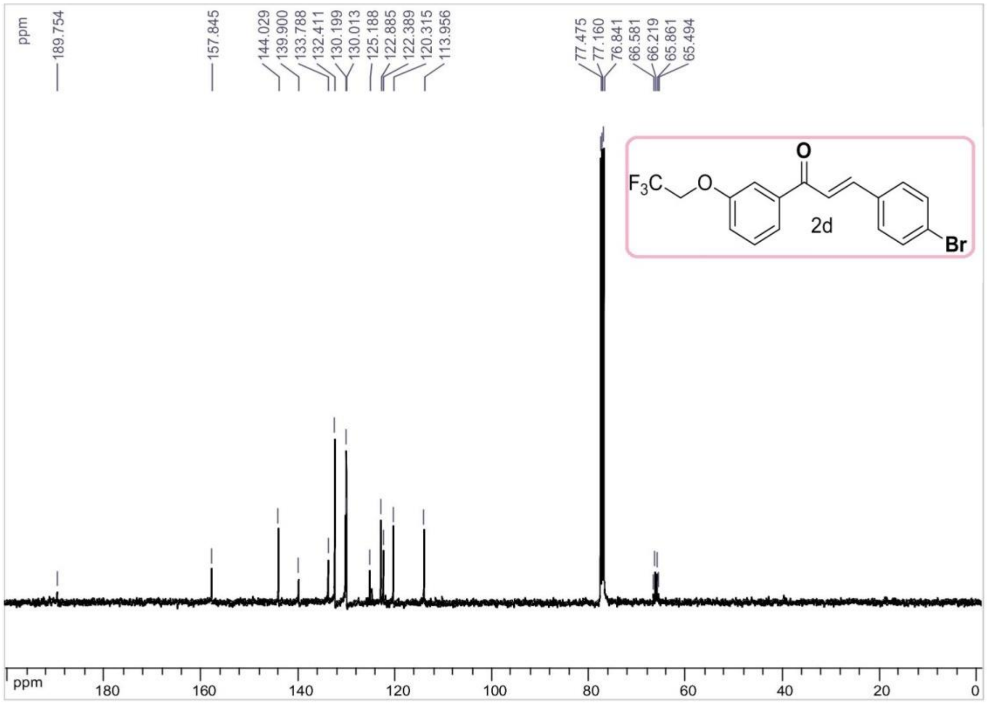

ppm
189.754
157.845
144.029
139.900
133.788
132.411
130.199
130.013
125.188
122.885
122.389
120.315
113.956
77.475
77.160
76.841
66.581
66.219
65.861
65.494
O
F3C
2d
Br
ppm
180
160
140
120
100
80
60
40
20
0

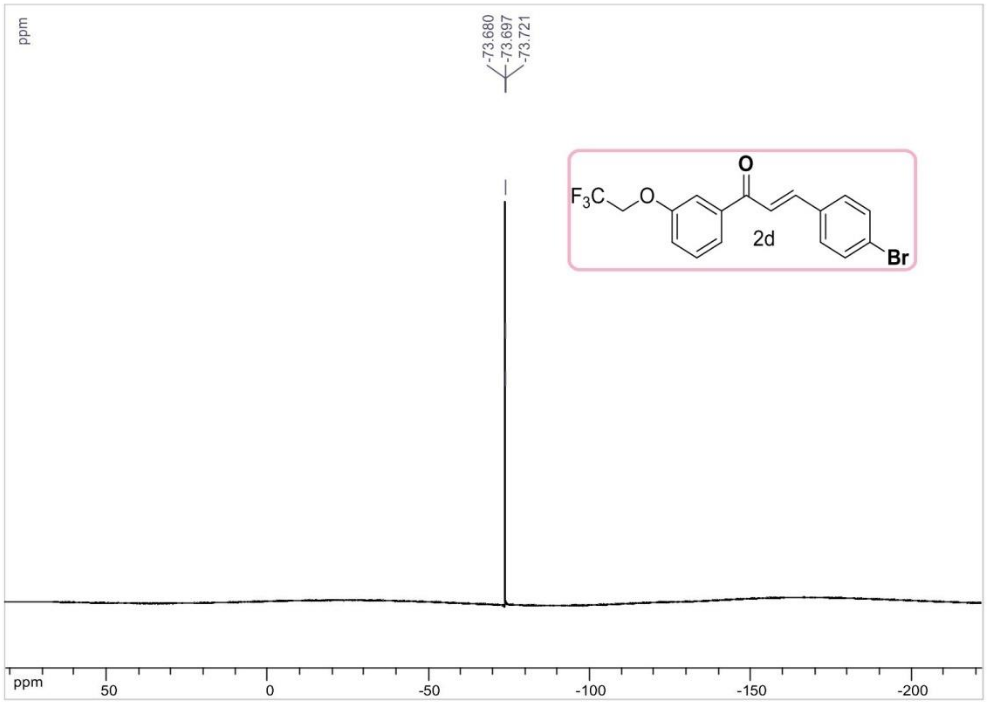

ppm
-73.680
-73.697
-73.721
O
F3C
2d
Br
ppm
50
0
-50
-100
-150
-200

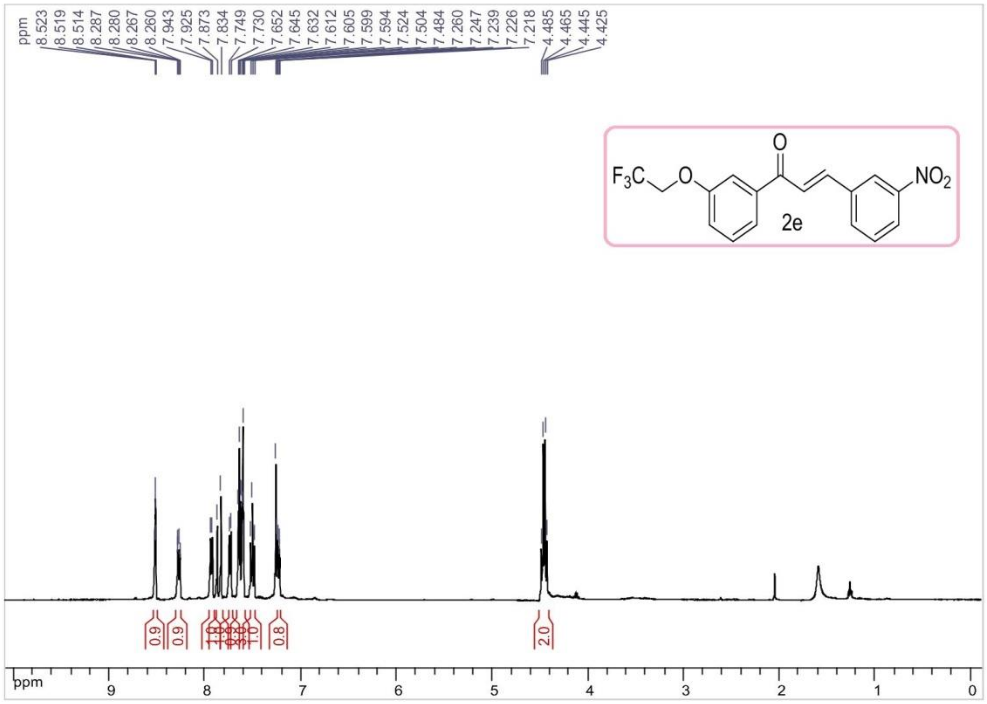
ppm
8.523
8.519
8.514
8.287
8.280
8.267
8.260
7.943
7.925
7.873
7.834
7.749
7.730
7.652
7.645
7.632
7.612
7.605
7.599
7.594
7.524
7.504
7.484
7.260
7.247
7.239
7.226
7.218
4.485
4.465
4.445
4.425
F_3C
O
O
NO_2
2e
0.9
0.9
0.8
2.0
ppm
9
8
7
6
5
4
3
2
1
0

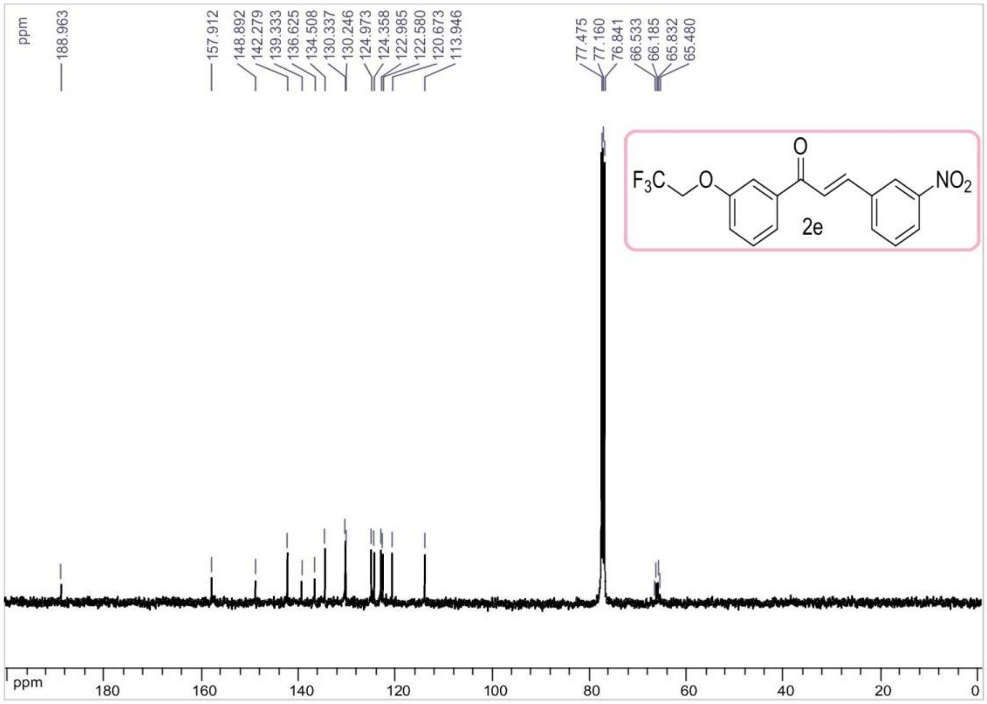
ppm
188.963
157.912
148.892
142.279
139.333
136.625
134.508
130.337
130.246
124.973
124.358
122.985
122.580
120.673
113.946
77.475
77.160
76.841
66.533
66.185
65.832
65.480
F_3C
O
O
NO_2
2e
ppm
180
160
140
120
100
80
60
40
20
0

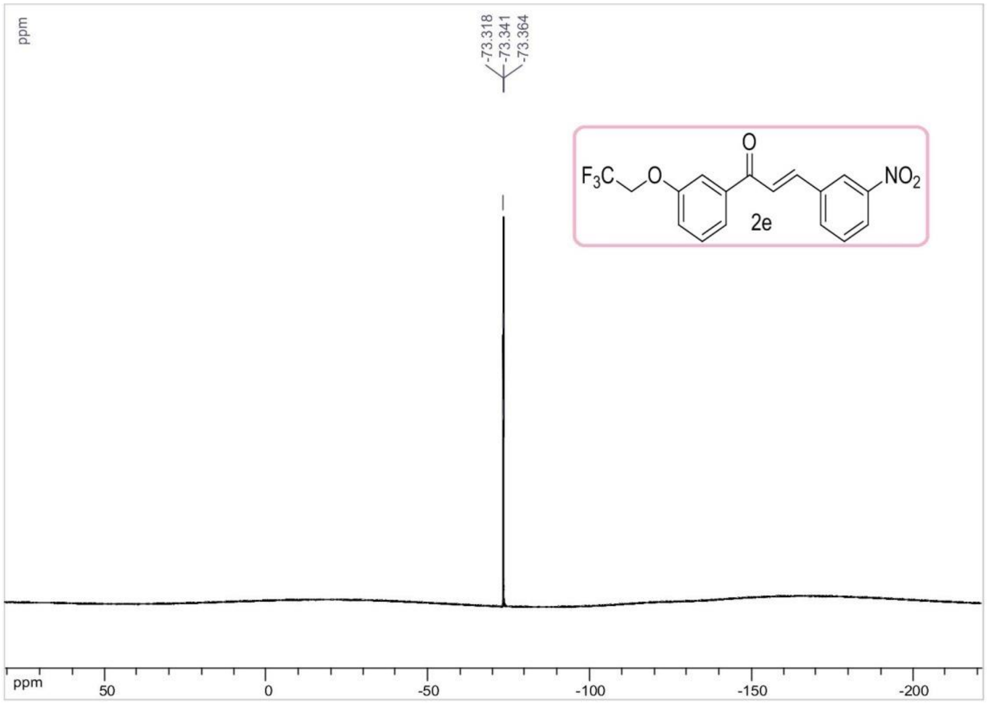
ppm
-73.318
-73.341
-73.364
O
F3C
O
NO2
2e
ppm
50
0
-50
-100
-150
-200

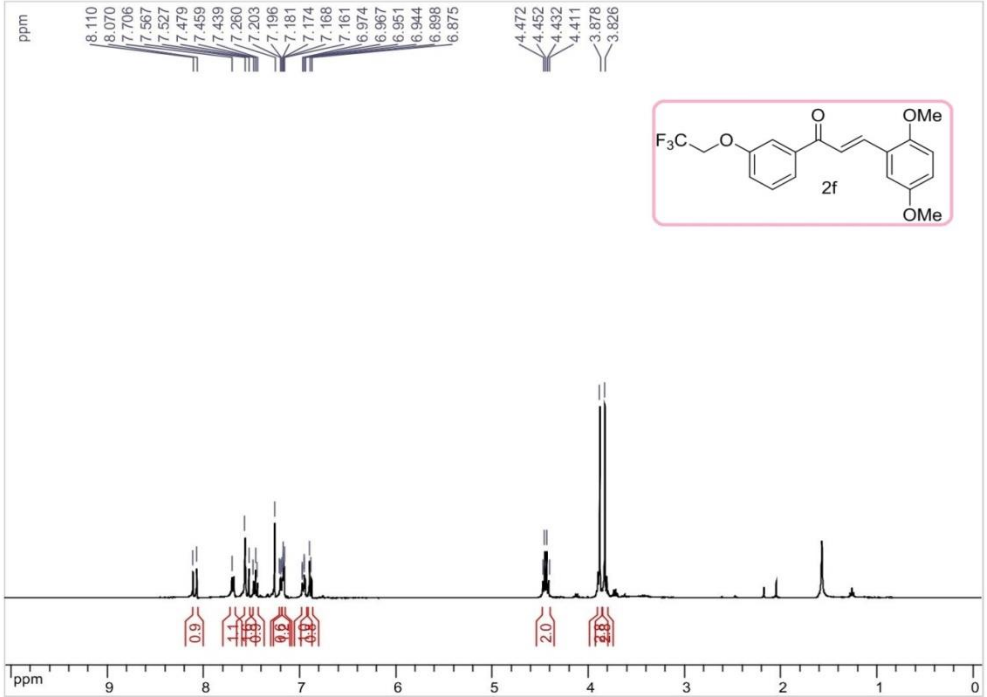
ppm
8.110
8.070
7.706
7.567
7.527
7.479
7.459
7.439
7.260
7.203
7.196
7.181
7.174
7.168
7.161
6.974
6.967
6.951
6.944
6.898
6.875
4.472
4.452
4.432
4.411
3.878
3.826
O
OMe
F3C
O
2f
OMe
0.9
1.1
2.0
ppm
9
8
7
6
5
4
3
2
1
0

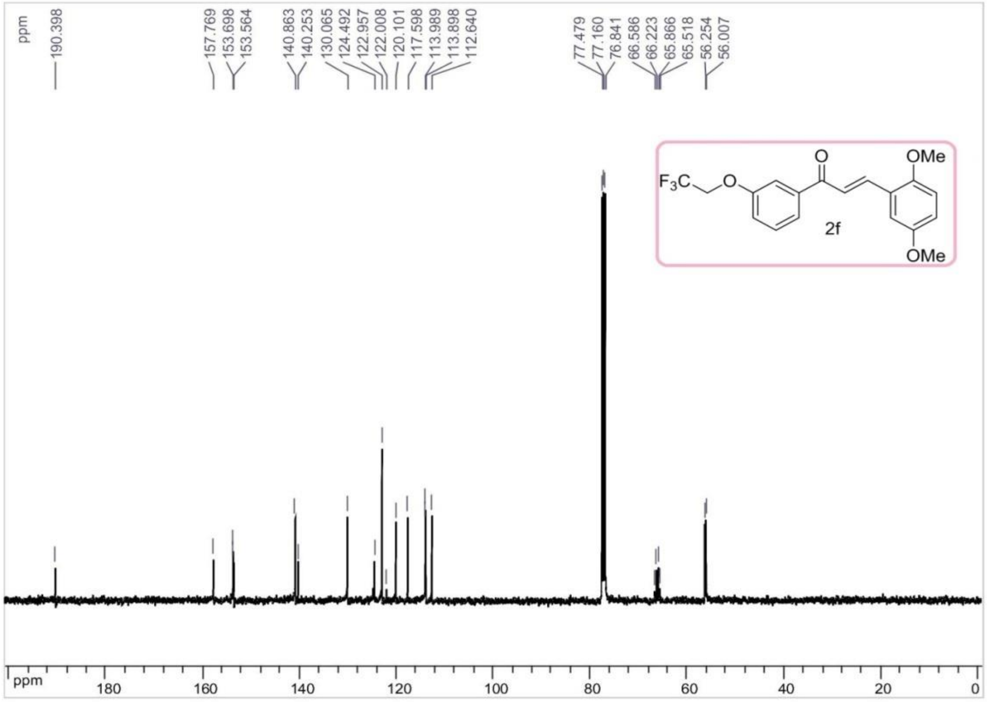
ppm
190.398
157.769
153.698
153.564
140.863
140.253
130.065
124.492
122.957
122.008
120.101
117.598
113.989
113.898
112.640
77.479
77.160
76.841
66.586
66.223
65.866
65.518
56.254
56.007
O
OMe
F3C
O
2f
OMe
ppm
180
160
140
120
100
80
60
40
20
0

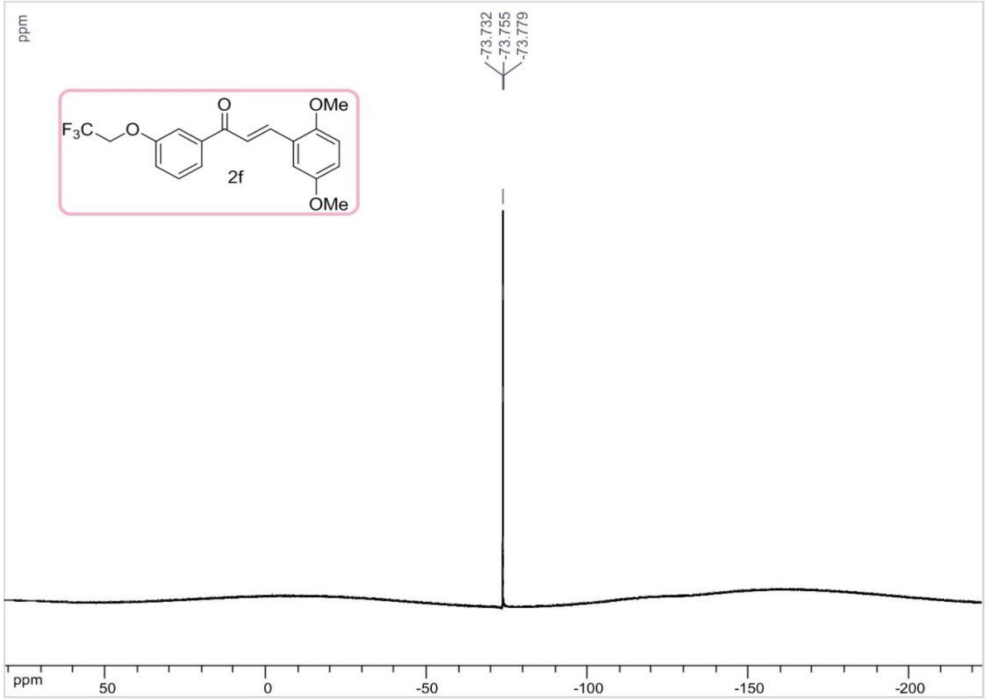
ppm
-73.732
-73.755
-73.779
O
OMe
F3C
O
2f
OMe
ppm
50
0
-50
-100
-150
-200

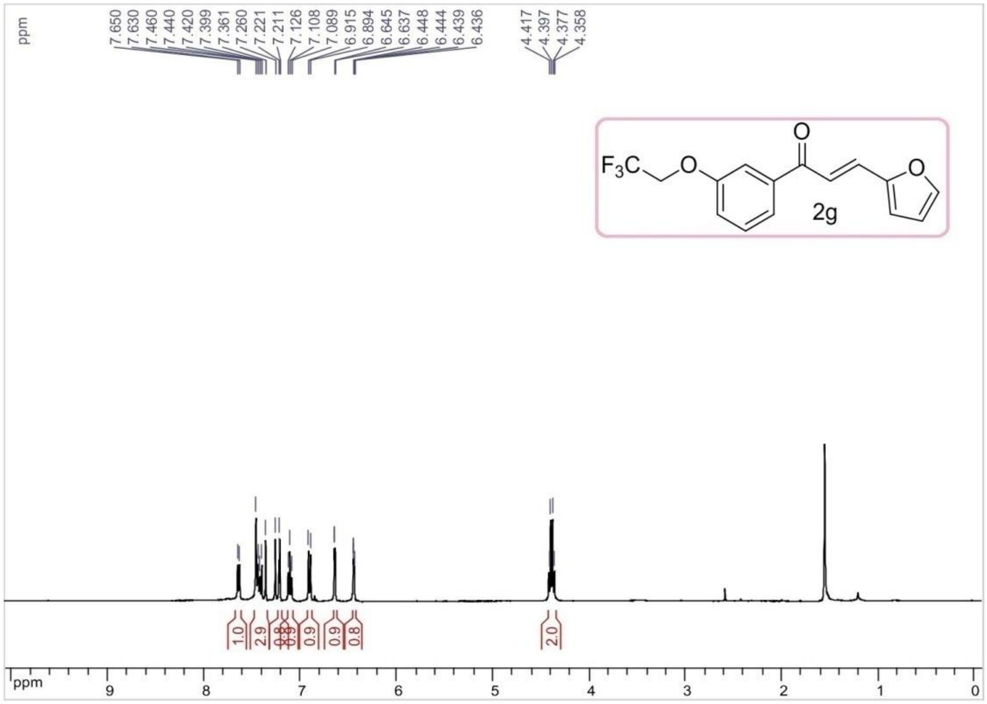
ppm
7.650
7.630
7.460
7.440
7.420
7.399
7.361
7.260
7.221
7.211
7.126
7.108
7.089
6.915
6.894
6.645
6.637
6.448
6.444
6.439
6.436
4.417
4.397
4.377
4.358
F3C
O
O
2g
1.0
2.9
0.9
0.9
0.8
2.0
ppm
9
8
7
6
5
4
3
2
1
0

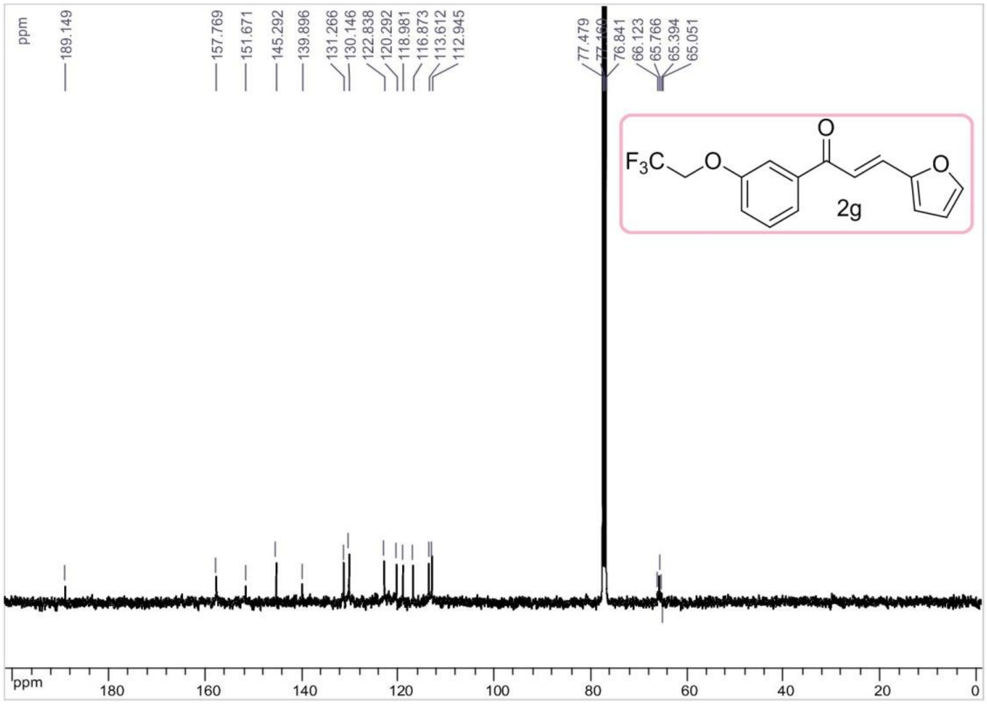
ppm
189.149
157.769
151.671
145.292
139.896
131.266
130.146
122.838
120.292
118.981
116.873
113.612
112.945
77.479
76.841
66.123
65.766
65.394
65.051
F3C
O
O
2g
ppm
180
160
140
120
100
80
60
40
20
0

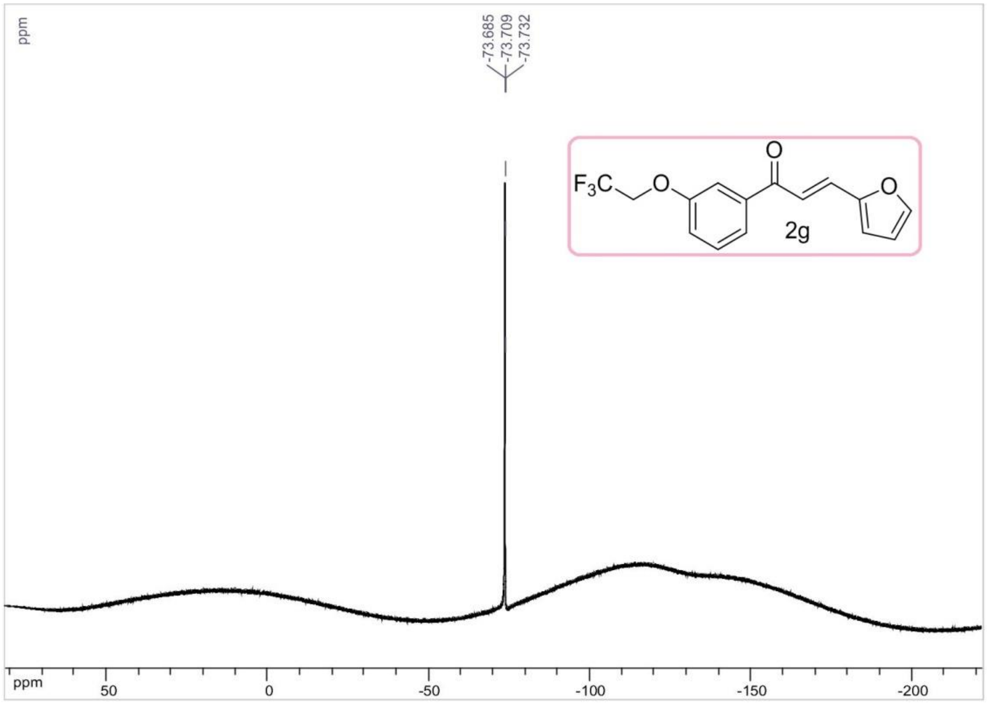
-73.685
-73.709
-73.732
F3C
O
O
O
2g
ppm
50
0
-50
-100
-150
-200

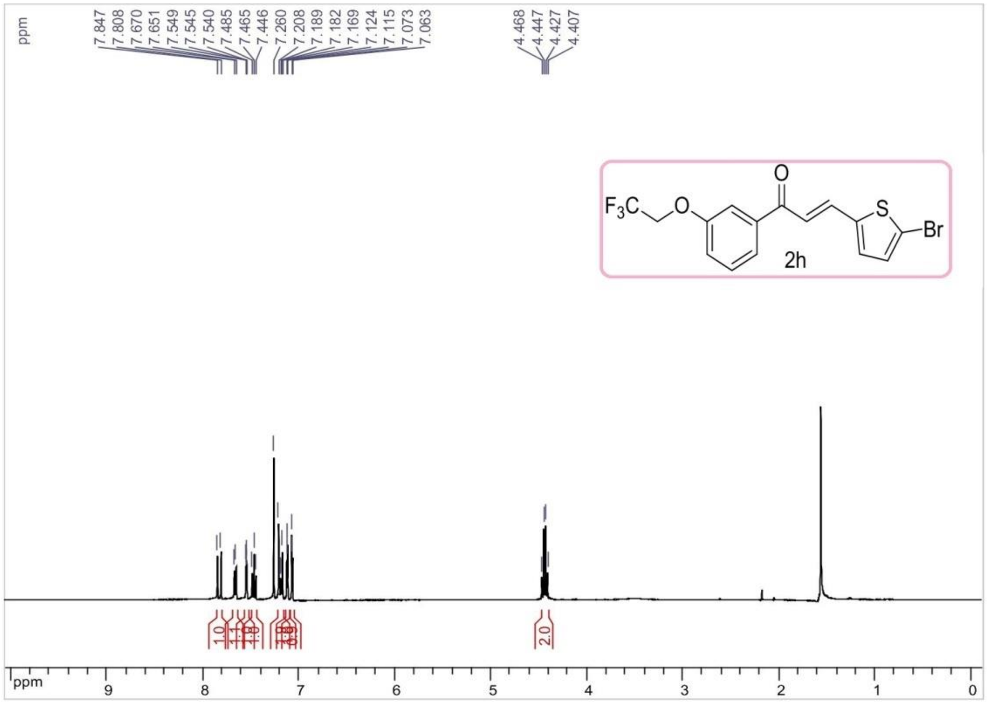
7.847
7.808
7.670
7.651
7.549
7.545
7.540
7.485
7.465
7.446
7.260
7.208
7.189
7.182
7.169
7.124
7.115
7.073
7.063
4.468
4.447
4.427
4.407
F3C
O
O
S
Br
2h
2.0
ppm
9
8
7
6
5
4
3
2
1
0

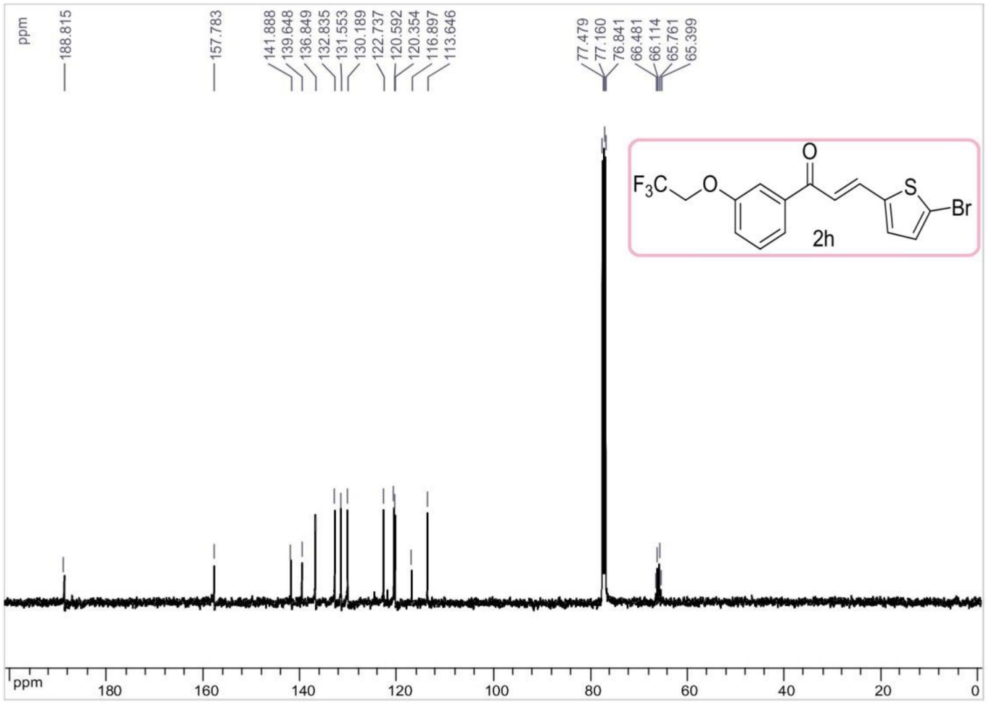
ppm
188.815
157.783
141.888
139.648
136.849
132.835
131.553
130.189
122.737
120.592
120.354
116.897
113.646
77.479
77.160
76.841
66.481
66.114
65.761
65.399
F_3C
O
S
Br
2h
ppm
180
160
140
120
100
80
60
40
20
0

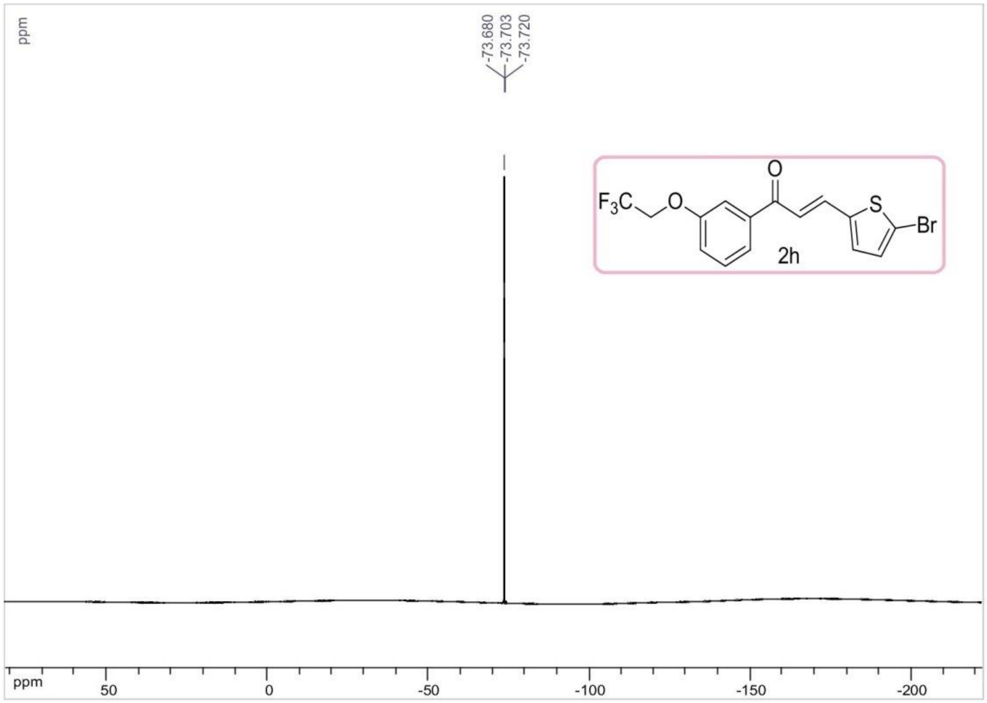
ppm
-73.680
-73.703
-73.720
F_3C
O
S
Br
2h
ppm
50
0
-50
-100
-150
-200

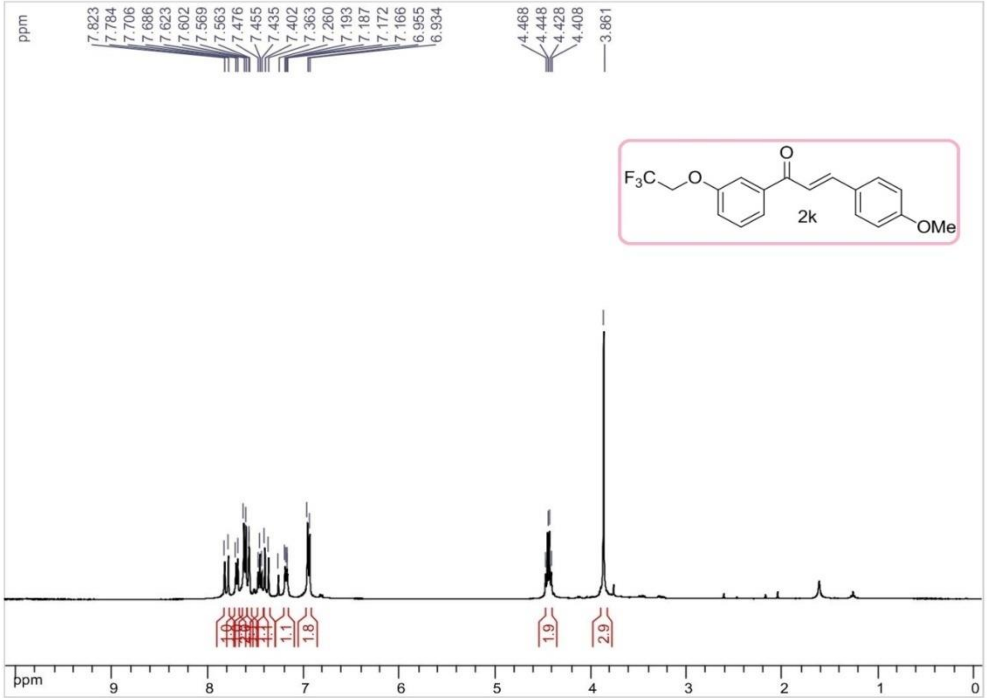
7.823
7.784
7.706
7.686
7.623
7.602
7.569
7.563
7.476
7.455
7.435
7.402
7.363
7.260
7.193
7.187
7.172
7.166
6.955
6.934
4.468
4.448
4.428
4.408
3.861
2k
OMe
1.9
2.9
1.1
1.8
ppm
9
8
7
6
5
4
3
2
1
0

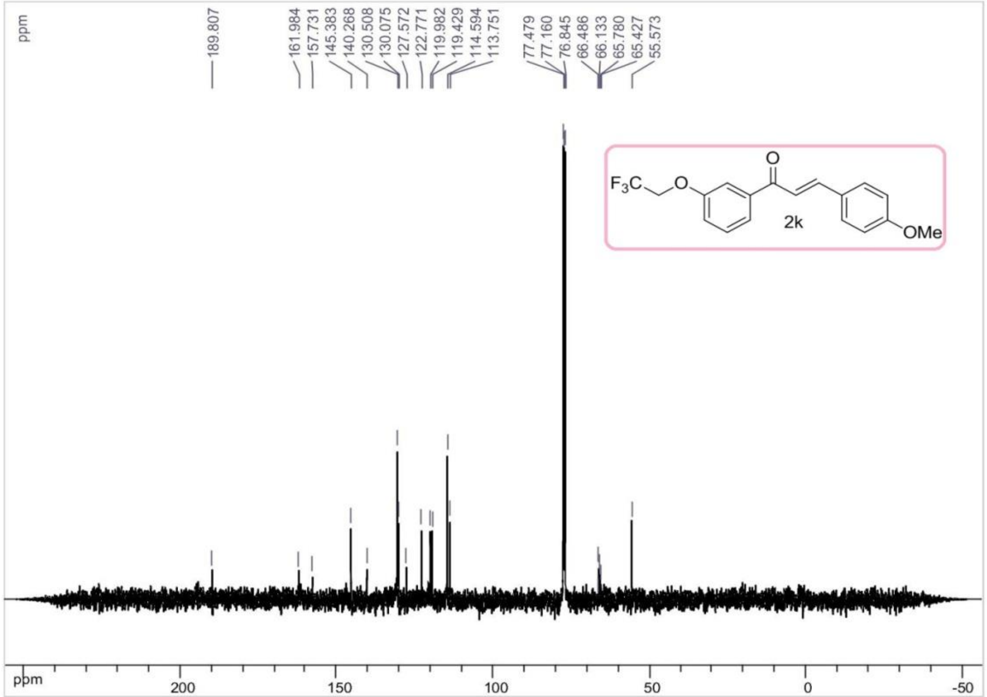
189.807
161.984
157.731
145.383
140.268
130.508
130.075
127.572
122.771
119.982
119.429
114.594
113.751
77.479
77.160
76.845
66.486
66.133
65.780
65.427
55.573
2k
OMe
ppm
200
150
100
50
0
-50

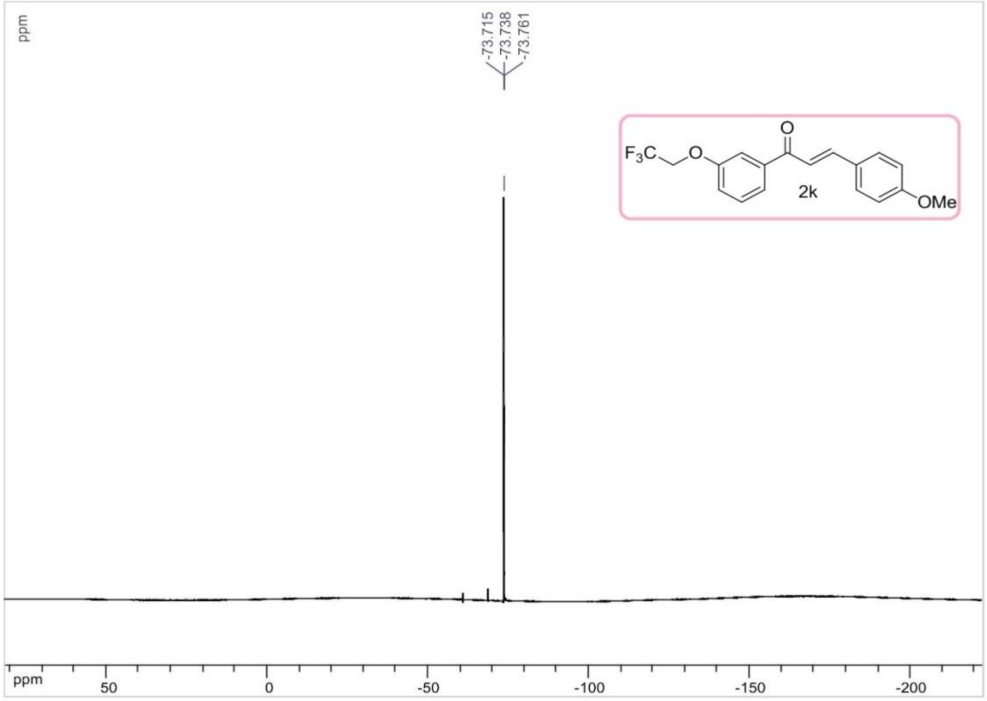
ppm
-73.715
-73.738
-73.761
F3C
O
O
2k
OMe
ppm
50
0
-50
-100
-150
-200

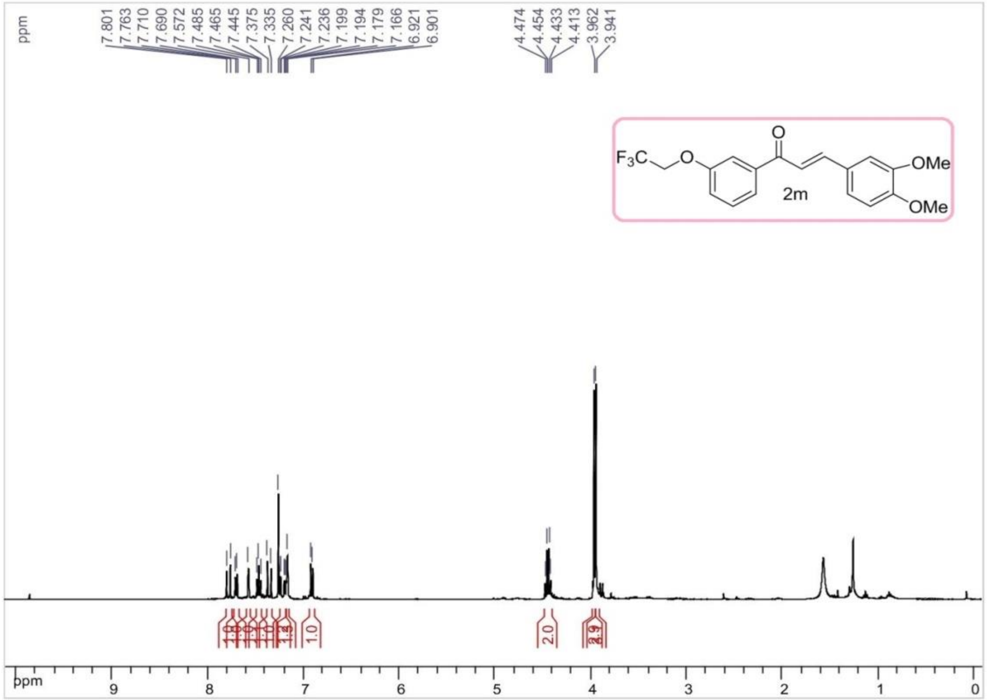
ppm
7.801
7.763
7.710
7.690
7.572
7.485
7.465
7.445
7.375
7.335
7.260
7.241
7.236
7.199
7.194
7.179
7.166
6.921
6.901
4.474
4.454
4.433
4.413
3.962
3.941
F3C
O
O
OMe
2m
OMe
1.0
2.0
ppm
9
8
7
6
5
4
3
2
1
0

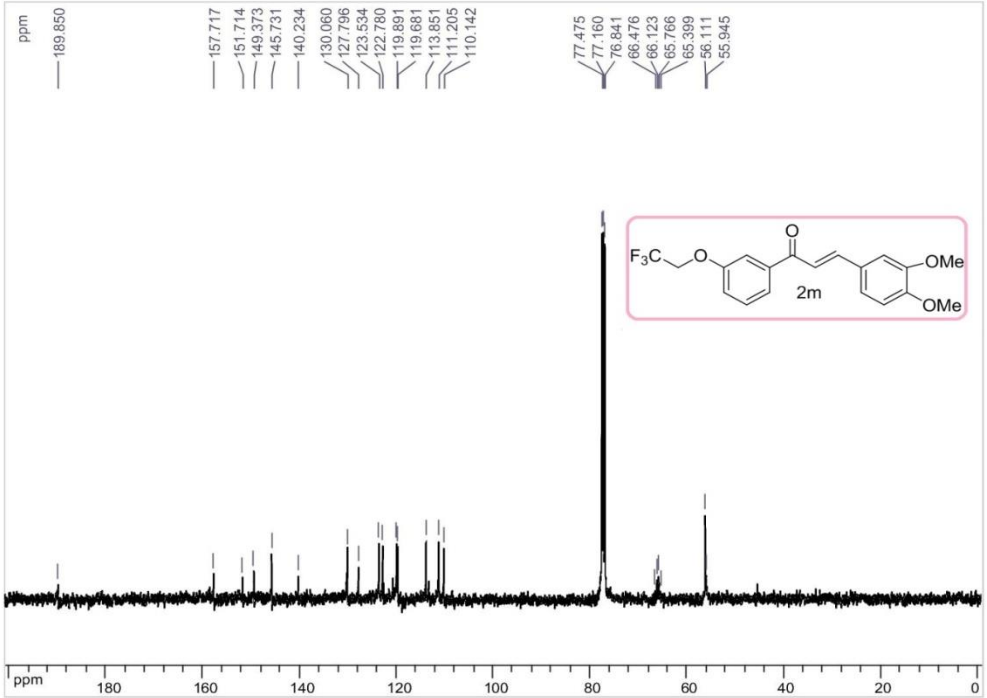
ppm
189.850
157.717
151.714
149.373
145.731
140.234
130.060
127.796
123.534
122.780
119.891
119.681
113.851
111.205
110.142
77.475
77.160
76.841
66.476
66.123
65.766
65.399
56.111
55.945
F3C
O
OMe
OMe
2m
ppm
180
160
140
120
100
80
60
40
20
0

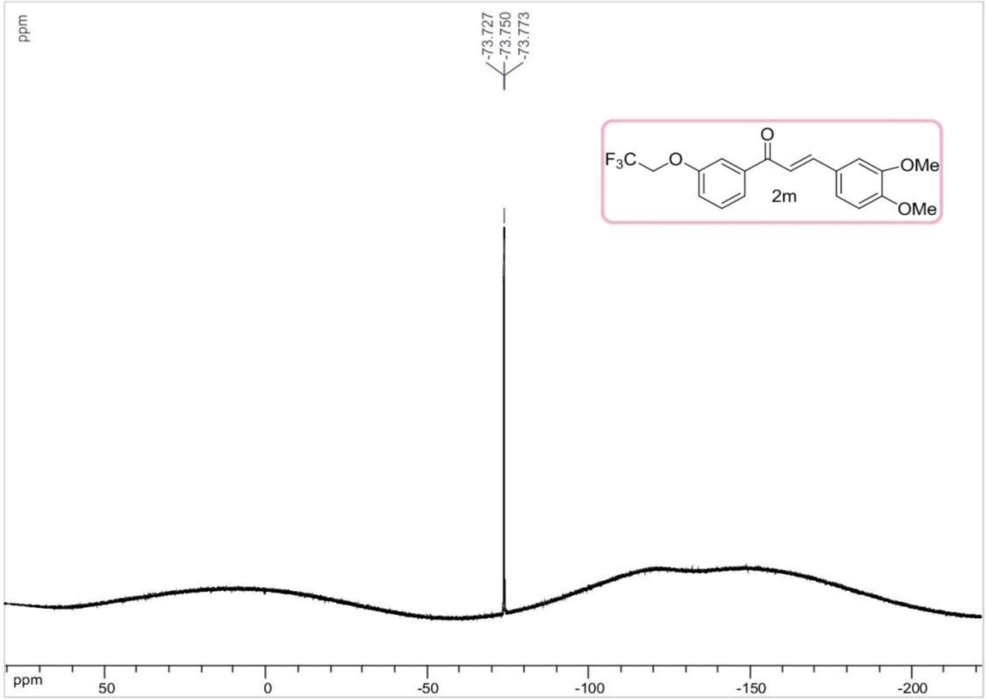
ppm
-73.727
-73.750
-73.773
F3C
O
OMe
OMe
2m
ppm
50
0
-50
-100
-150
-200

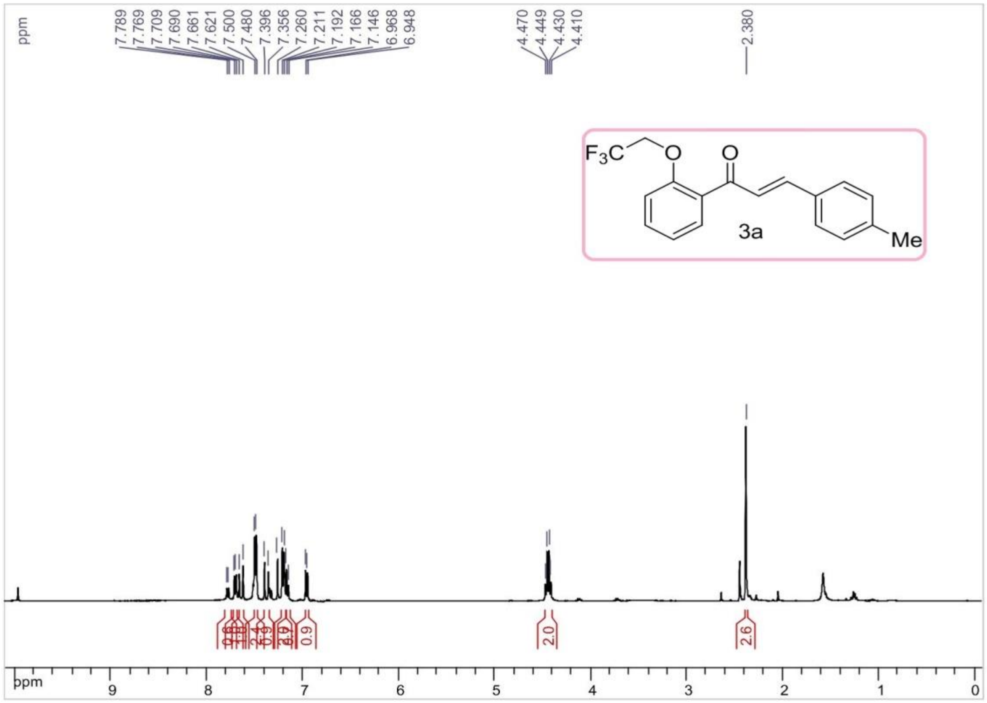
ppm
7.789
7.769
7.709
7.690
7.661
7.621
7.500
7.480
7.396
7.356
7.260
7.211
7.192
7.166
7.146
6.968
6.948
4.470
4.449
4.430
4.410
2.380
F3C
O
O
3a
Me
0.9
2.0
2.6
ppm
9
8
7
6
5
4
3
2
1
0

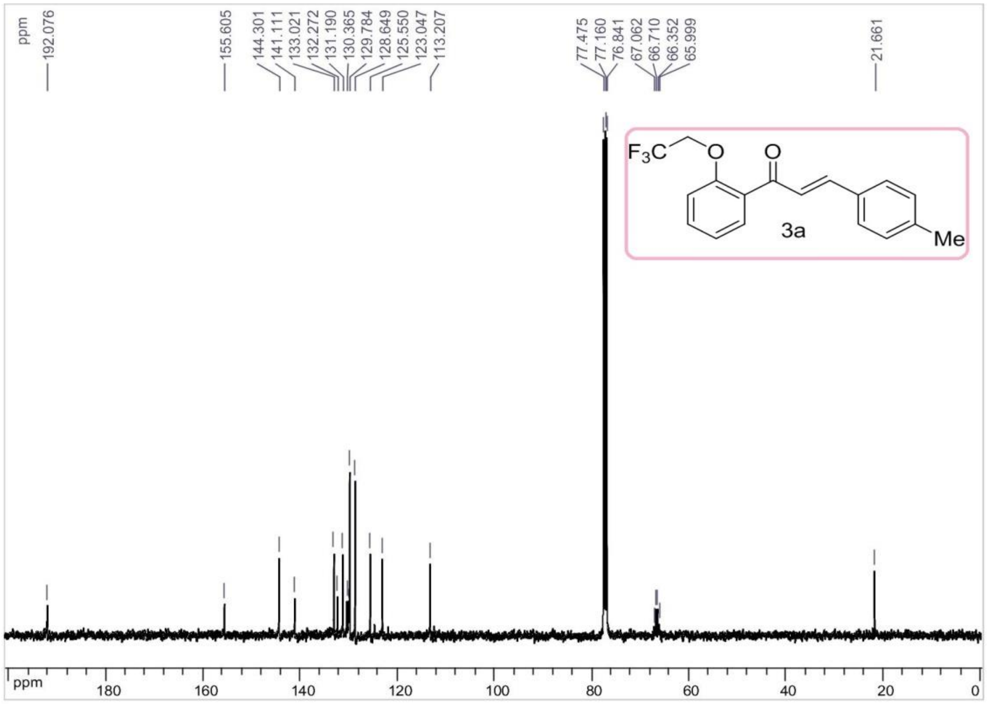
ppm
192.076
155.605
144.301
141.111
133.021
132.272
131.190
130.365
129.784
128.649
125.550
123.047
113.207
77.475
77.160
76.841
67.062
66.710
66.352
65.999
21.661
F3C
O
O
3a
Me
ppm
180
160
140
120
100
80
60
40
20
0

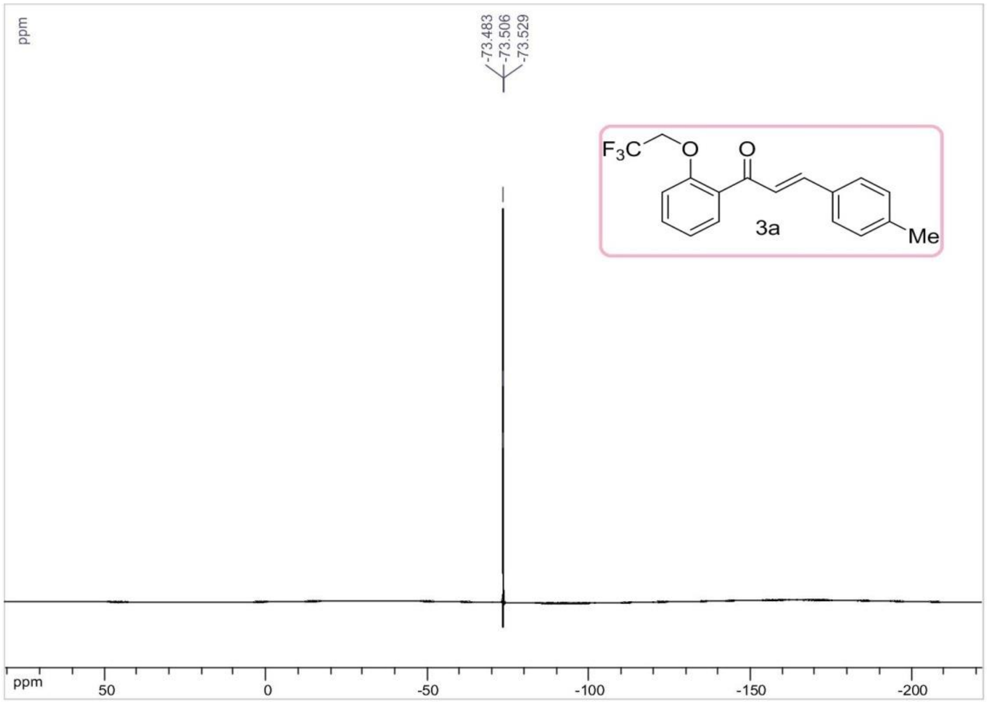
ppm
-73.483
-73.506
-73.529
F3C
O
O
3a
Me
ppm 50 0 -50 -100 -150 -200

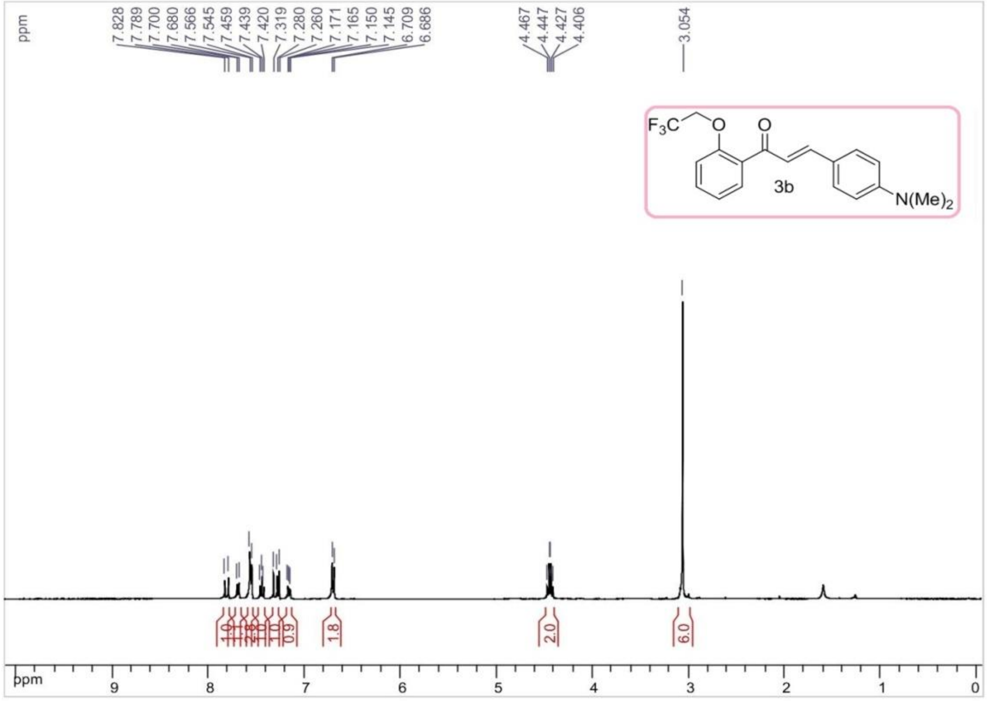
ppm
7.828
7.789
7.700
7.680
7.566
7.545
7.459
7.439
7.420
7.319
7.280
7.260
7.171
7.165
7.150
7.145
6.709
6.686
4.467
4.447
4.427
4.406
3.054
F3C
O
O
3b
N(Me)2
0.9
1.8
2.0
6.0
ppm 9 8 7 6 5 4 3 2 1 0

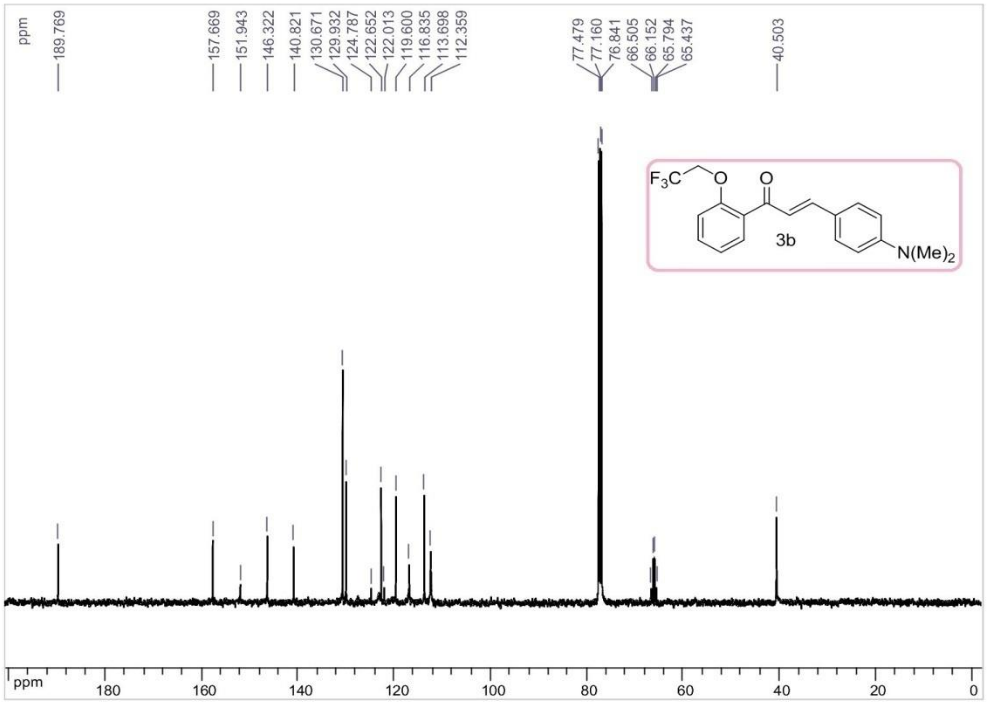
ppm
189.769
157.669
151.943
146.322
140.821
130.671
129.932
124.787
122.652
122.013
119.600
116.835
113.698
112.359
77.479
77.160
76.841
66.505
66.152
65.794
65.437
40.503
F3C
O
O
3b
N(Me)2
ppm
180
160
140
120
100
80
60
40
20
0

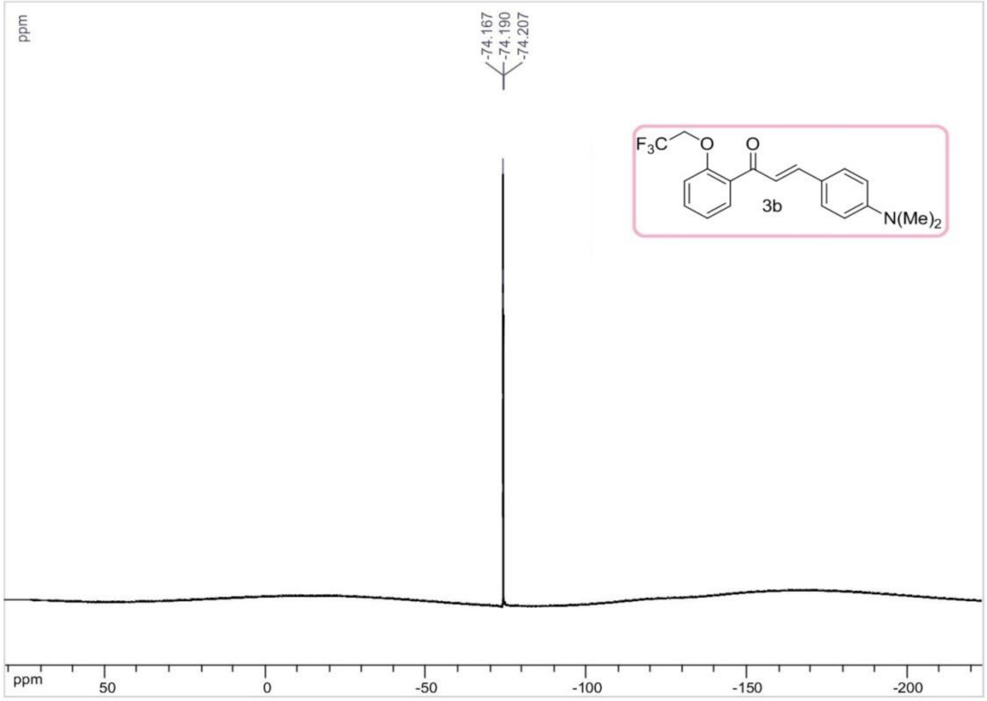
ppm
-74.167
-74.190
-74.207
F3C
O
O
3b
N(Me)2
ppm
50
0
-50
-100
-150
-200

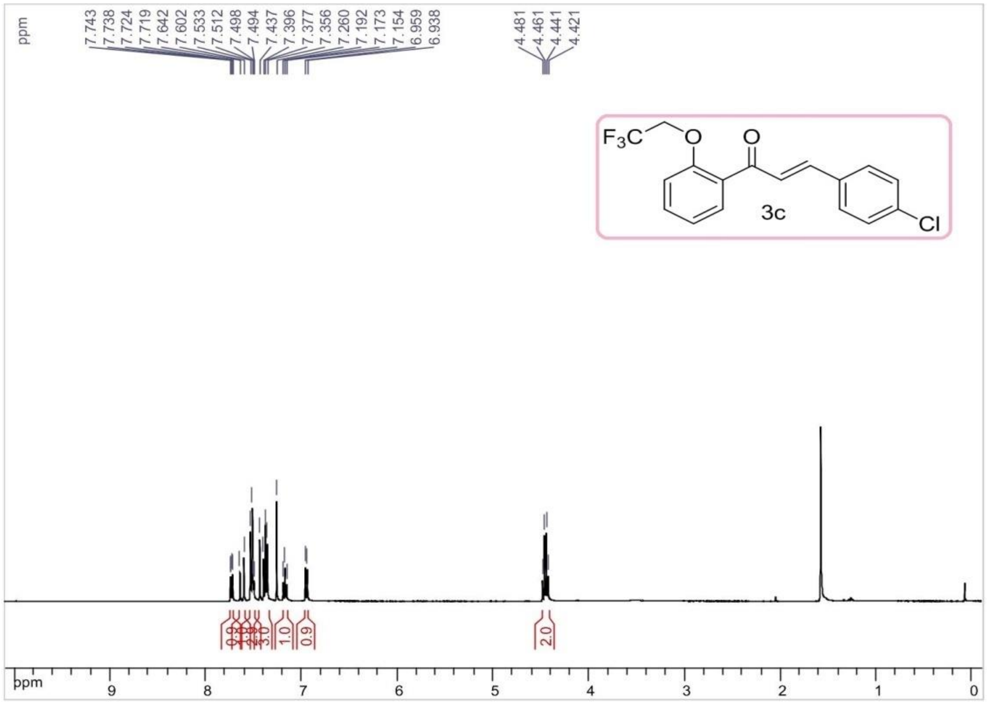
ppm
7.743
7.738
7.724
7.719
7.642
7.602
7.533
7.512
7.498
7.494
7.437
7.396
7.377
7.356
7.260
7.192
7.173
7.154
6.959
6.938
4.481
4.461
4.441
4.421
F_3C
O
O
3c
Cl
1.0
0.9
2.0
ppm
9
8
7
6
5
4
3
2
1
0

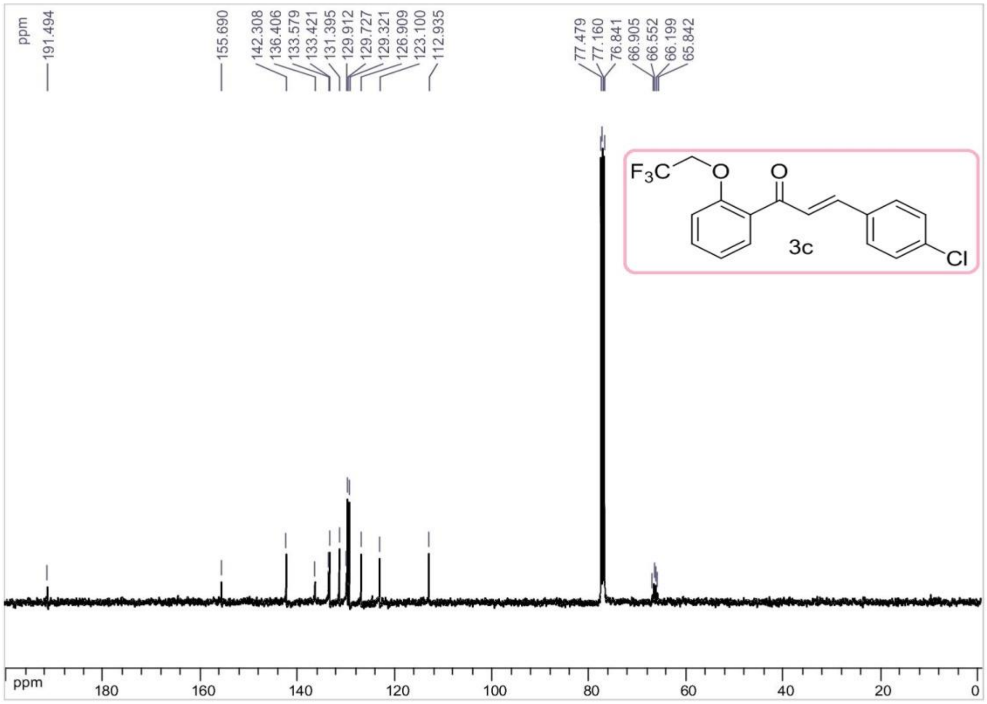
ppm
191.494
155.690
142.308
136.406
133.579
133.421
131.395
129.912
129.727
129.321
126.909
123.100
112.935
77.479
77.160
76.841
66.905
66.552
66.199
65.842
F_3C
O
O
3c
Cl
ppm
180
160
140
120
100
80
60
40
20
0

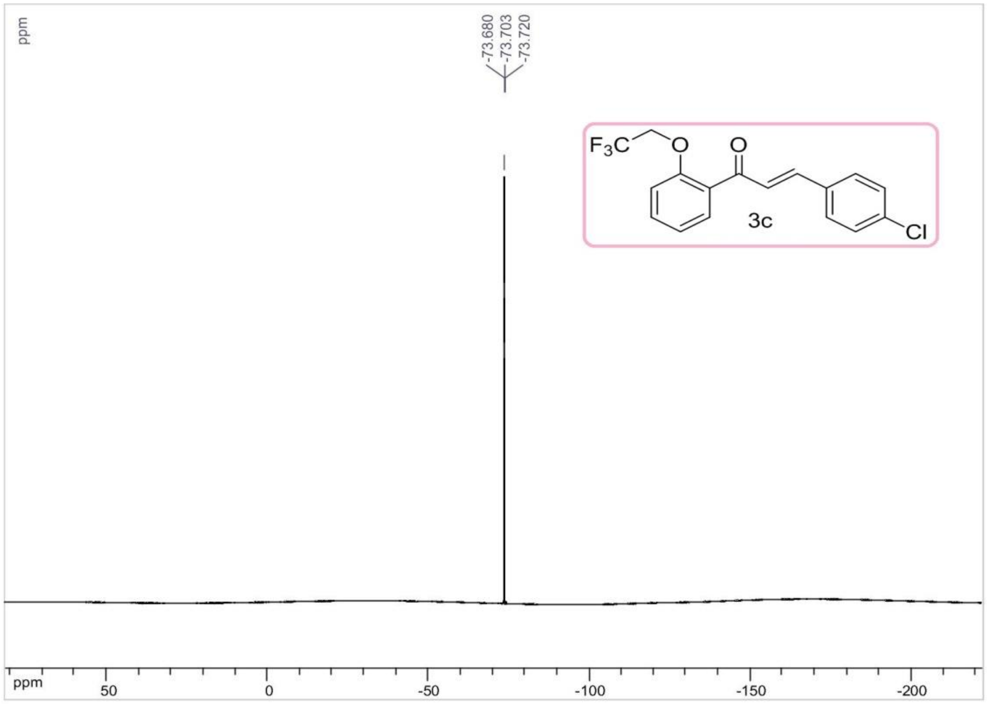
ppm
-73.680
-73.703
-73.720
F3C
O
O
3c
Cl
ppm
50
0
-50
-100
-150
-200

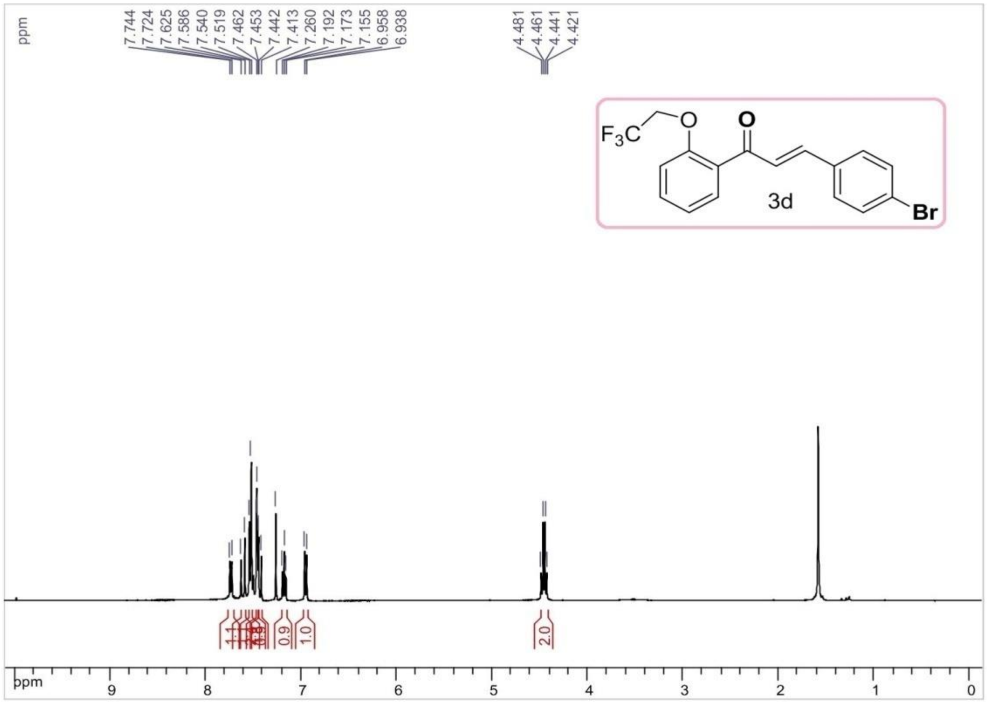
ppm
7.744
7.724
7.625
7.586
7.540
7.519
7.462
7.453
7.442
7.413
7.260
7.192
7.173
7.155
6.958
6.938
4.481
4.461
4.441
4.421
F3C
O
O
3d
Br
0.9
1.0
2.0
ppm
9
8
7
6
5
4
3
2
1
0

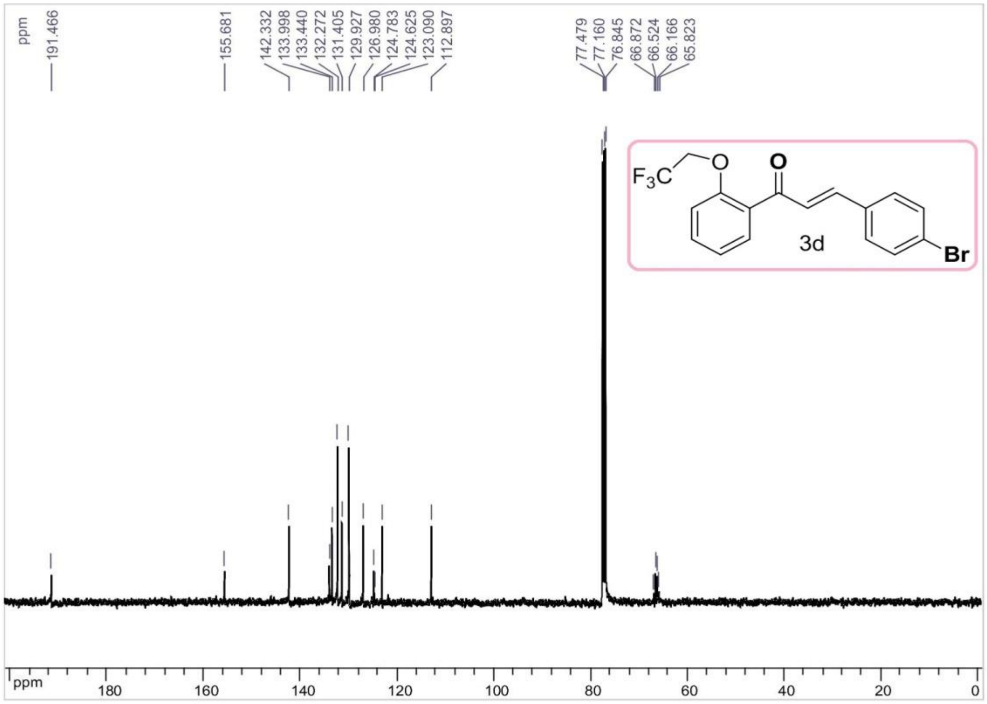

ppm
191.466
155.681
142.332
133.998
133.440
132.272
131.405
129.927
126.980
124.783
124.625
123.090
112.897
77.479
77.160
76.845
66.872
66.524
66.166
65.823
3d
ppm 180 160 140 120 100 80 60 40 20 0

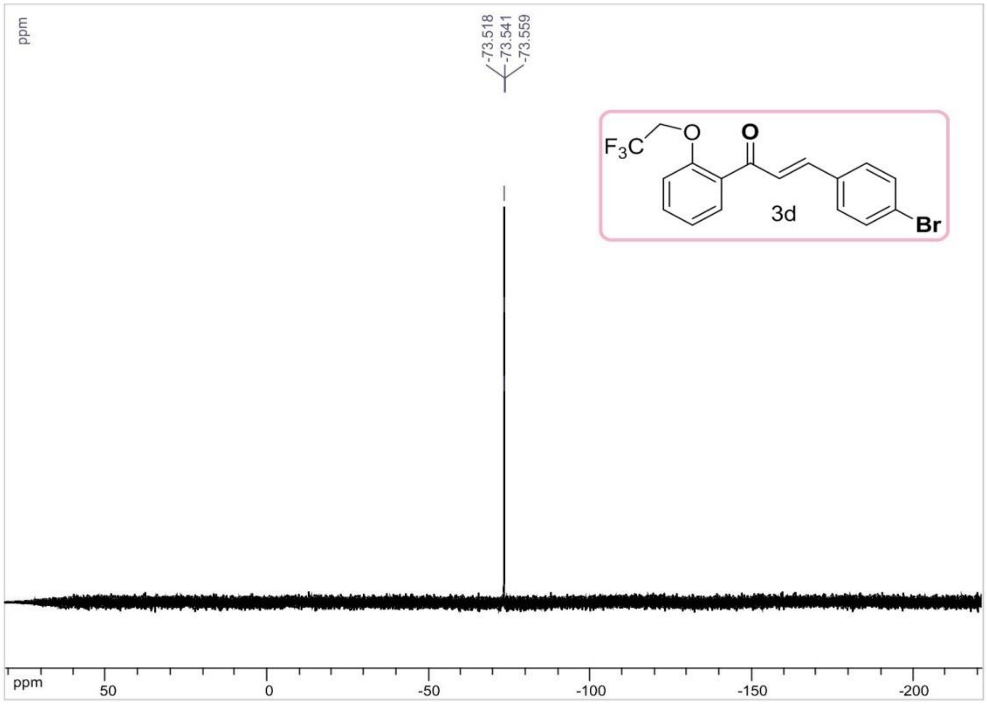

ppm
-73.518
-73.541
-73.559
3d
ppm 50 0 -50 -100 -150 -200

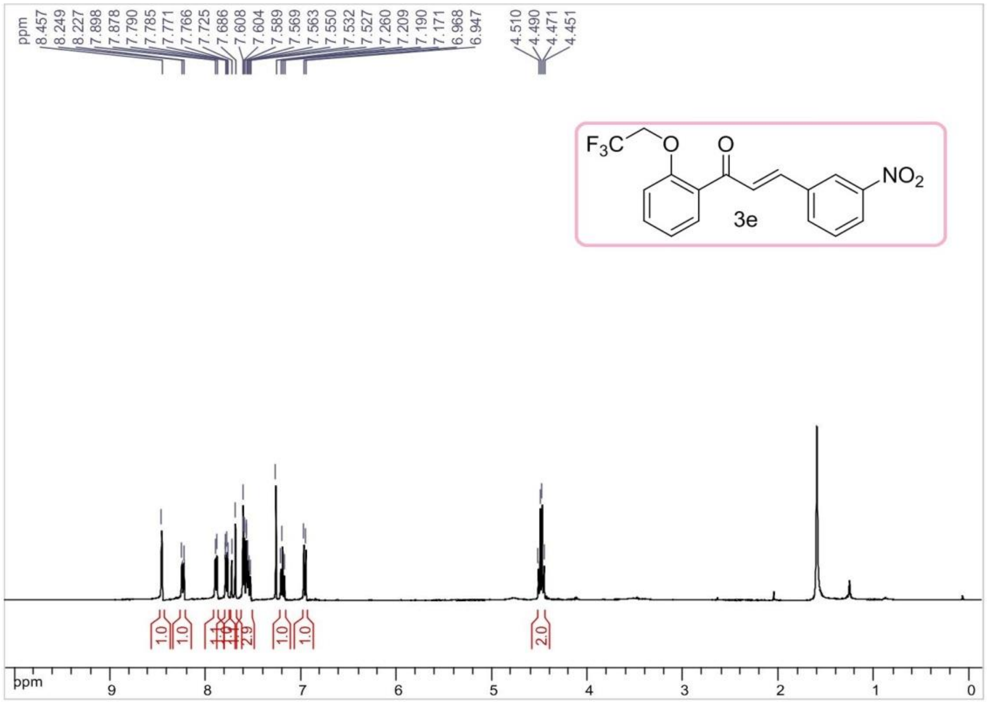

ppm
8.457
8.249
8.227
7.898
7.878
7.790
7.785
7.771
7.766
7.725
7.686
7.608
7.604
7.589
7.569
7.563
7.550
7.532
7.527
7.260
7.209
7.190
7.171
6.968
6.947
4.510
4.490
4.471
4.451
3e
1.0
1.0
2.0
ppm
9
8
7
6
5
4
3
2
1
0

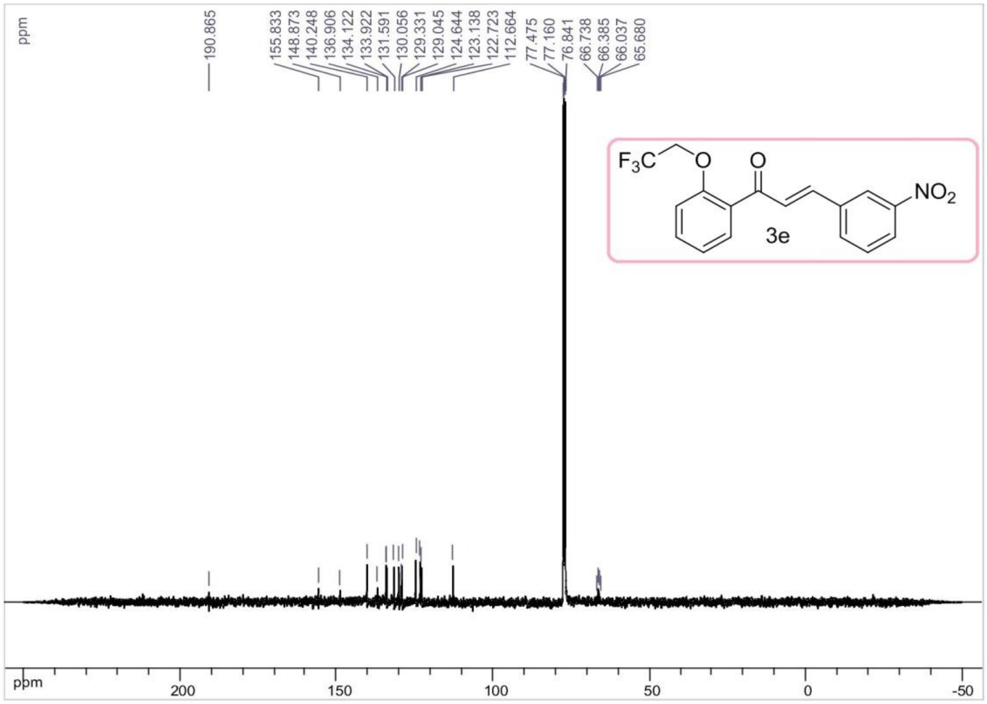

ppm
190.865
155.833
148.873
140.248
136.906
134.122
133.922
131.591
130.056
129.331
129.045
124.644
123.138
122.723
112.664
77.475
77.160
76.841
66.738
66.385
66.037
65.680
3e
ppm
200
150
100
50
0
-50

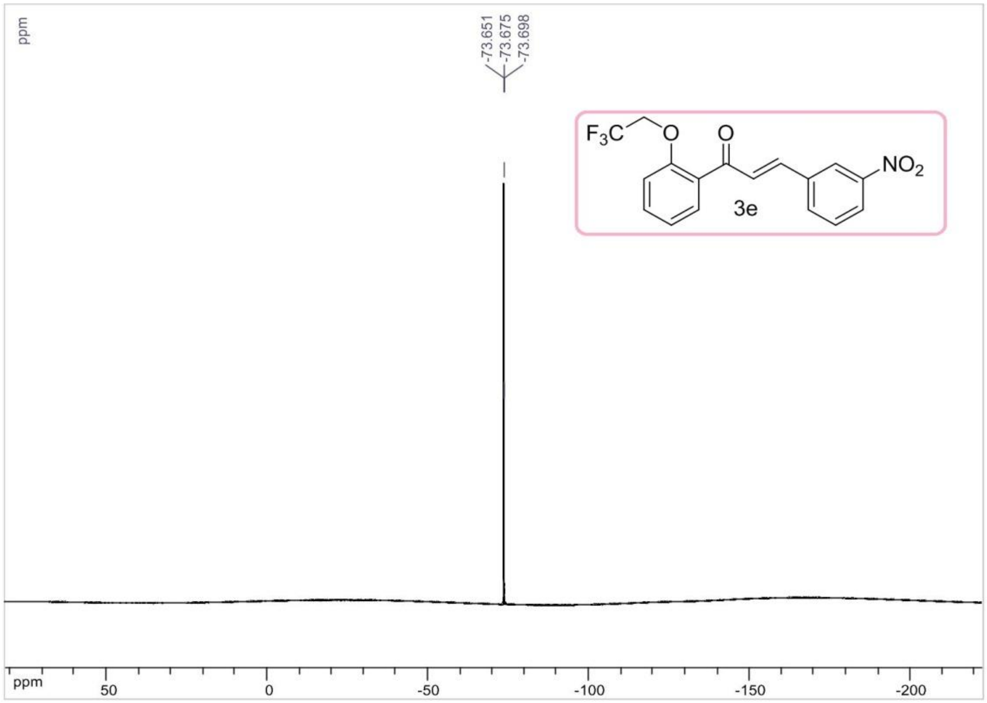
ppm
-73.651
-73.675
-73.698
F3C
O
O
NO2
3e
ppm
50
0
-50
-100
-150
-200

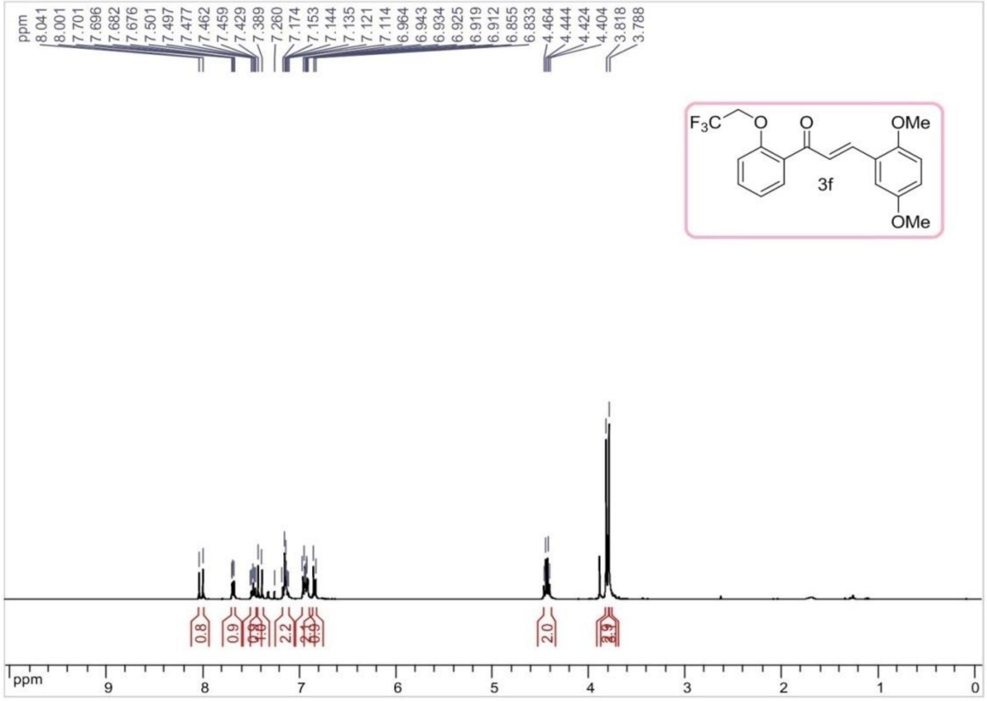
ppm
8.041
8.001
7.701
7.696
7.682
7.676
7.501
7.497
7.477
7.462
7.459
7.429
7.389
7.260
7.174
7.153
7.144
7.135
7.121
7.114
6.964
6.943
6.934
6.925
6.919
6.912
6.855
6.833
4.464
4.444
4.424
4.404
3.818
3.788
F3C
O
O
OMe
3f
OMe
0.8
0.9
2.2
2.0
ppm
9
8
7
6
5
4
3
2
1
0

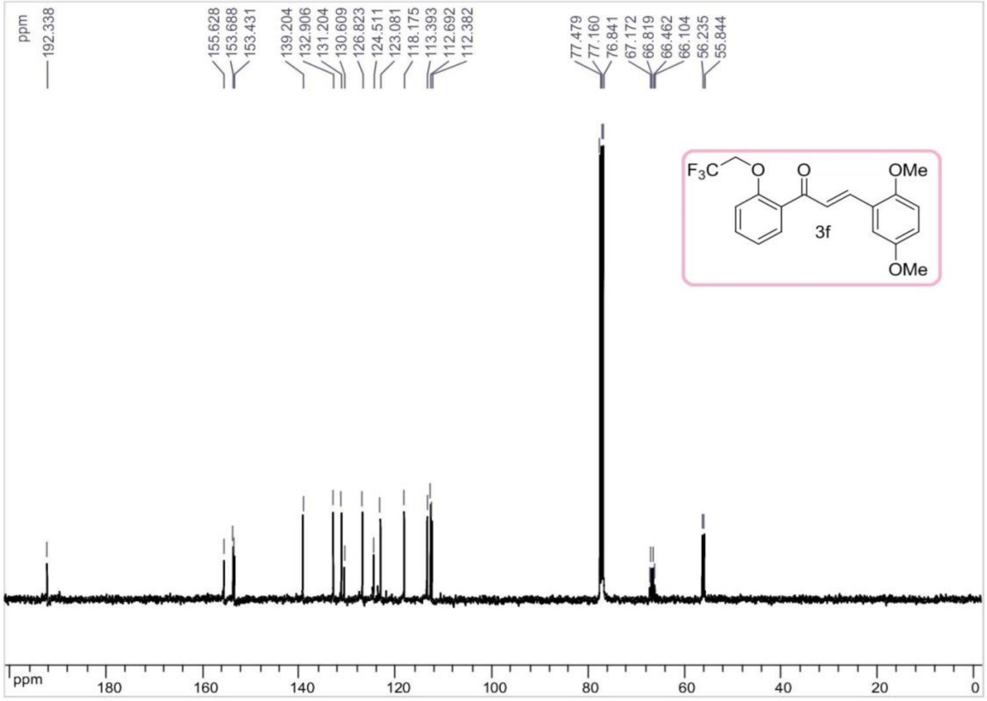
ppm
192.338
155.628
153.688
153.431
139.204
132.906
131.204
130.609
126.823
124.511
123.081
118.175
113.393
112.692
112.382
77.479
77.160
76.841
67.172
66.819
66.462
66.104
56.235
55.844
F3C
O
O
OMe
3f
OMe
ppm
180
160
140
120
100
80
60
40
20
0

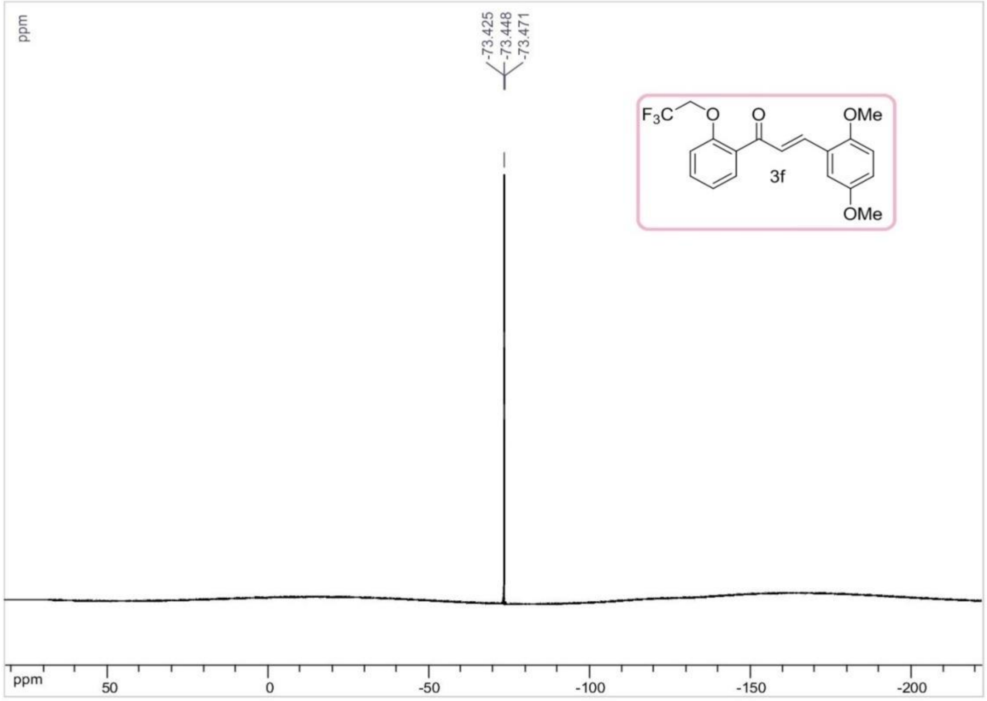
ppm
-73.425
-73.448
-73.471
F3C
O
O
OMe
3f
OMe
ppm
50
0
-50
-100
-150
-200

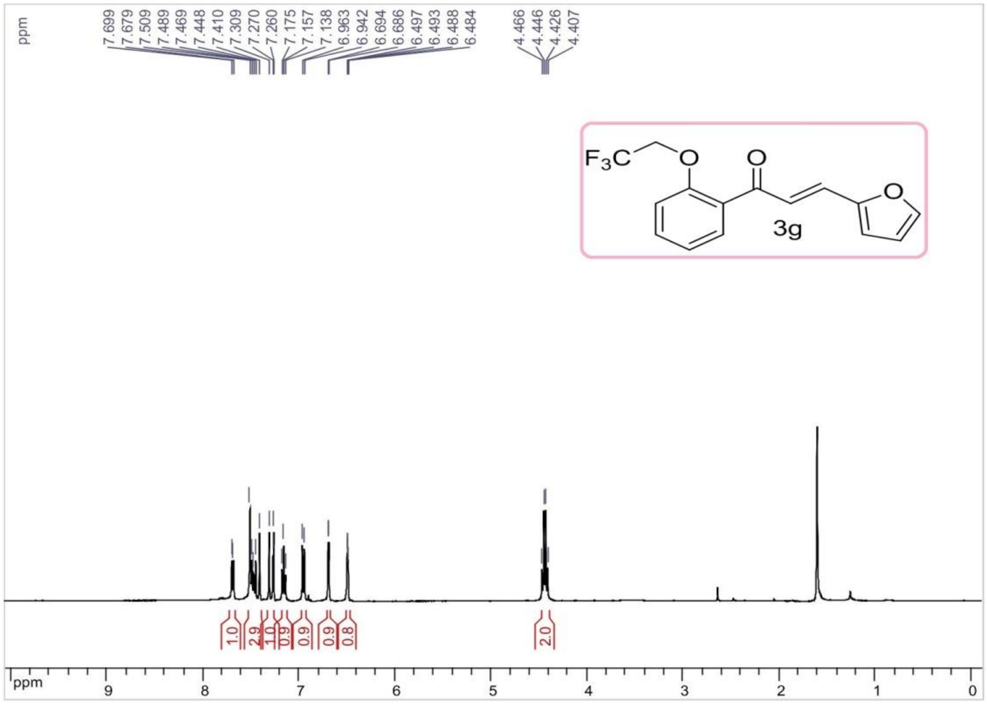
ppm
7.699
7.679
7.509
7.489
7.469
7.448
7.410
7.309
7.270
7.260
7.175
7.157
7.138
6.963
6.942
6.694
6.686
6.497
6.493
6.488
6.484
4.466
4.446
4.426
4.407
F_3C
O
O
O
3g
1.0
2.9
1.0
0.9
0.9
0.9
0.8
2.0
ppm
9
8
7
6
5
4
3
2
1
0

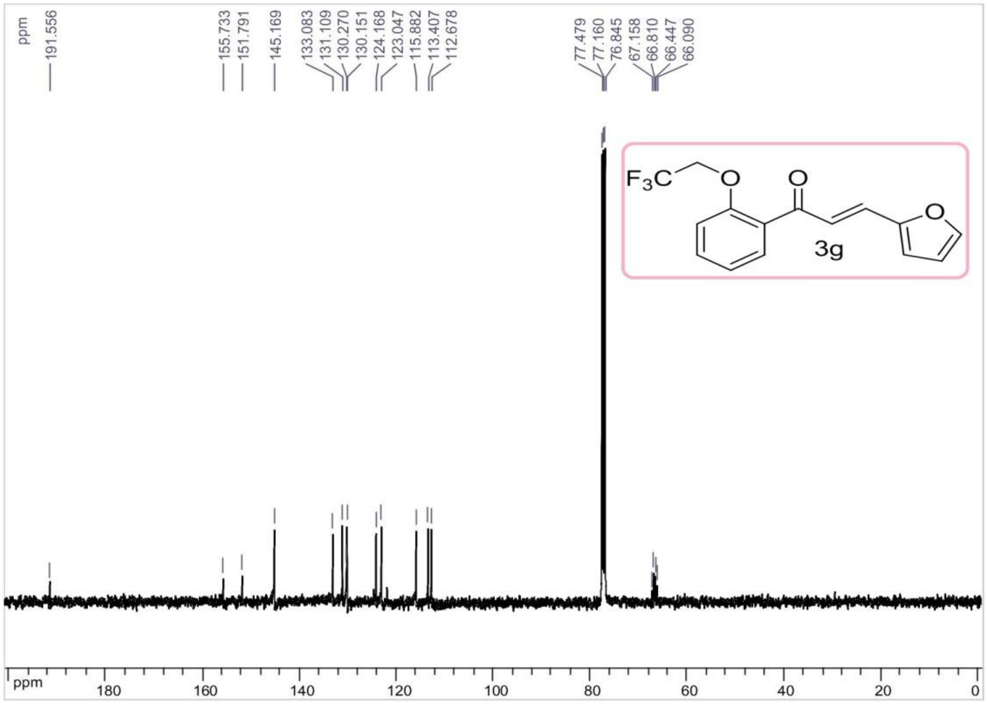
ppm
191.556
155.733
151.791
145.169
133.083
131.109
130.270
130.151
124.168
123.047
115.882
113.407
112.678
77.479
77.160
76.845
67.158
66.810
66.447
66.090
F_3C
O
O
O
3g
ppm
180
160
140
120
100
80
60
40
20
0

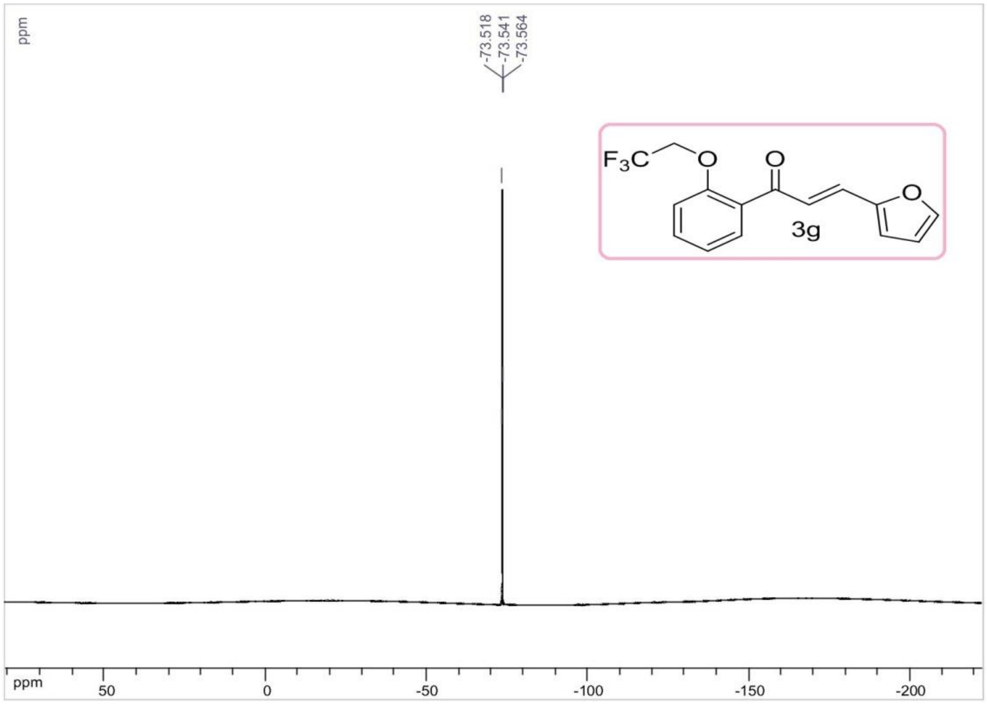
ppm
-73.518
-73.541
-73.564
F3C
O
O
O
3g
ppm
50
0
-50
-100
-150
-200

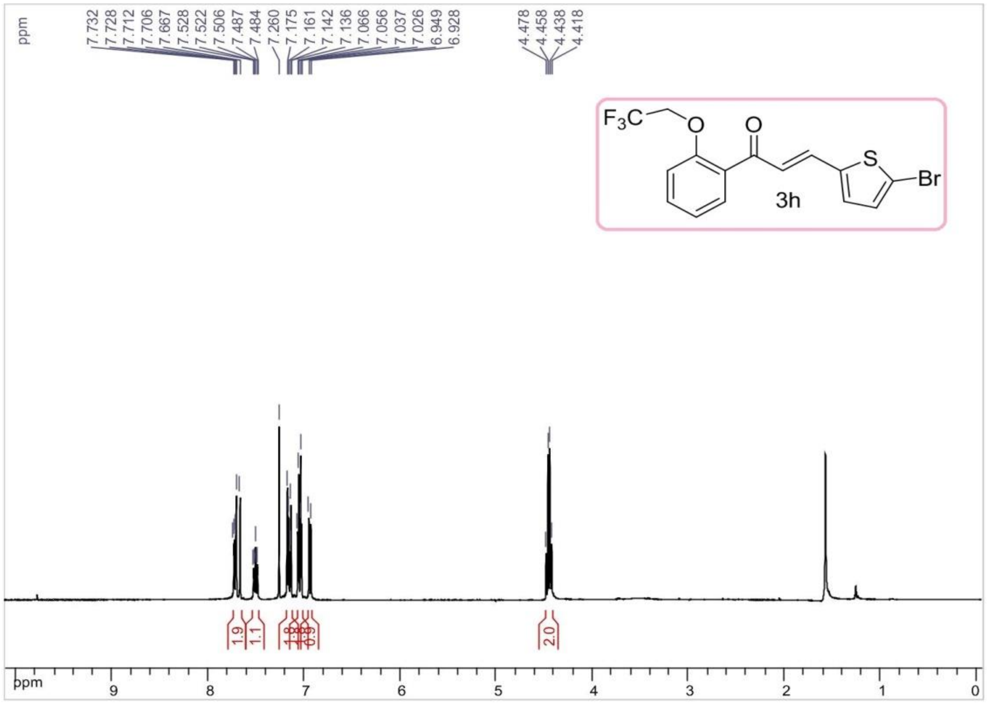
ppm
7.732
7.728
7.712
7.706
7.667
7.528
7.522
7.506
7.487
7.484
7.260
7.175
7.161
7.142
7.136
7.066
7.056
7.037
7.026
6.949
6.928
4.478
4.458
4.438
4.418
F3C
O
O
S
Br
3h
1.9
1.1
2.0
ppm
9
8
7
6
5
4
3
2
1
0

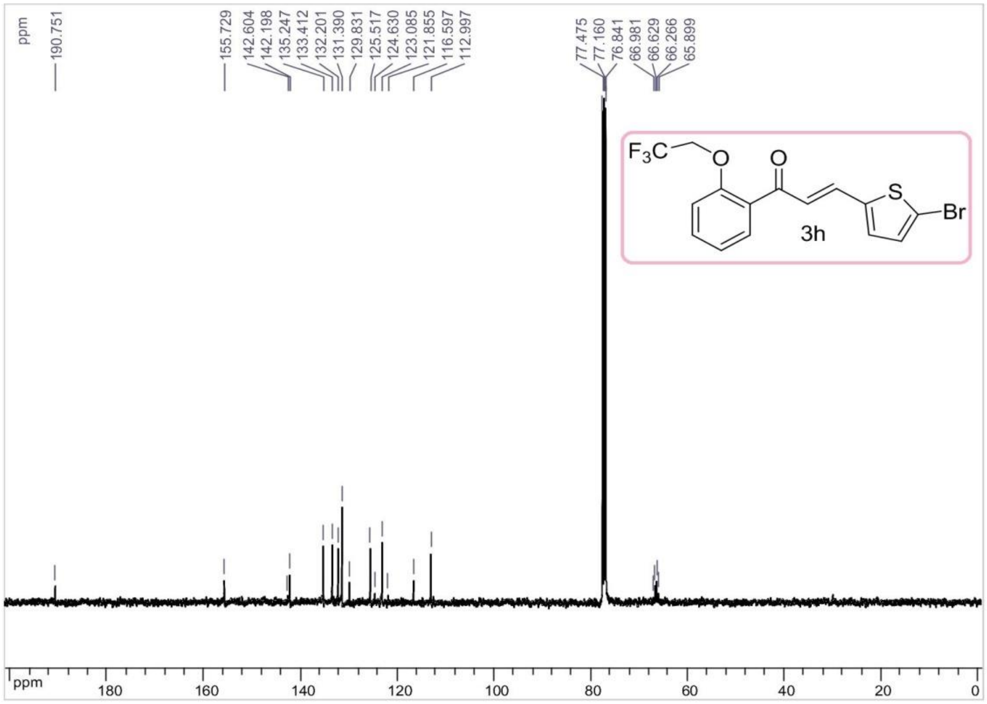
ppm
190.751
155.729
142.604
142.198
135.247
133.412
132.201
131.390
129.831
125.517
124.630
123.085
121.855
116.597
112.997
77.475
77.160
76.841
66.981
66.629
66.266
65.899
F3C
O
O
S
Br
3h
ppm
180
160
140
120
100
80
60
40
20
0

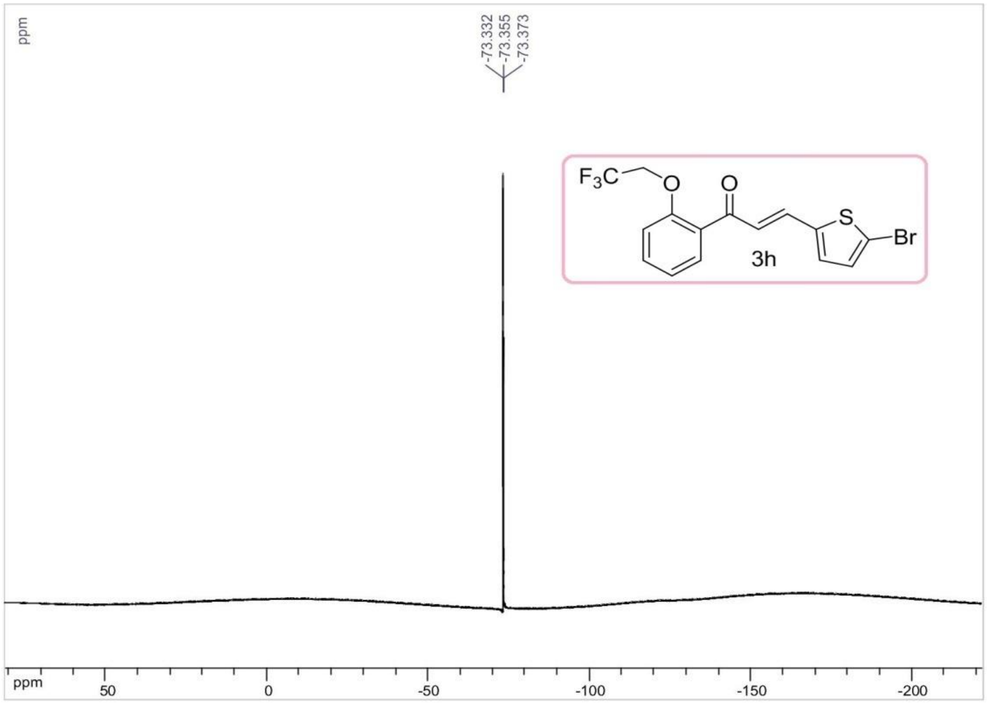
ppm
-73.332
-73.355
-73.373
F3C
O
O
S
Br
3h
ppm
50
0
-50
-100
-150
-200

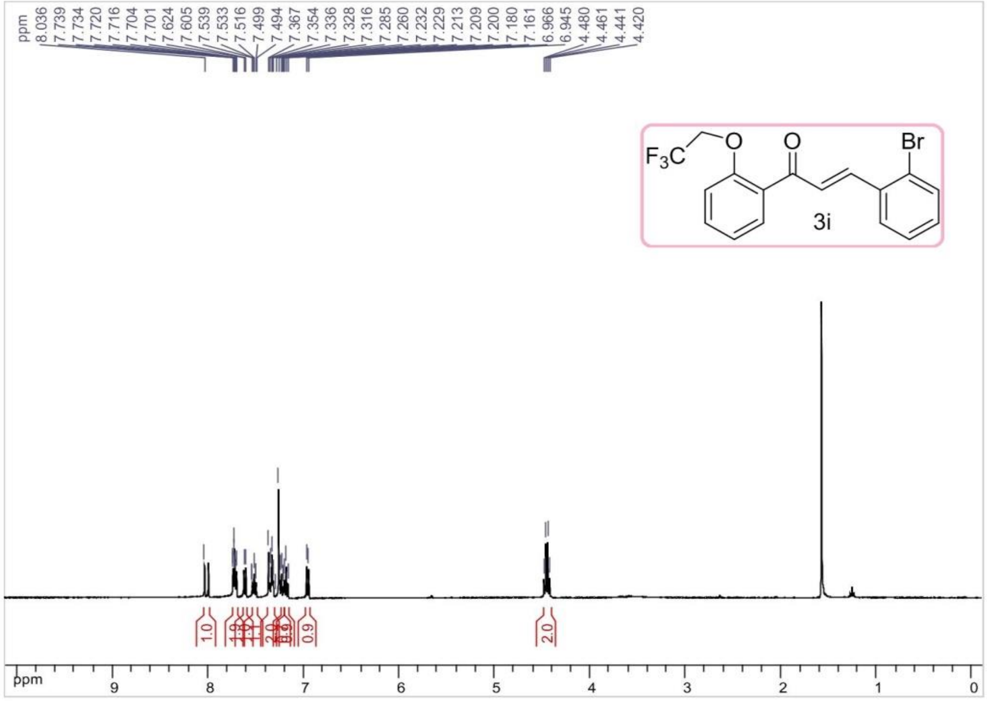
ppm
8.036
7.739
7.734
7.720
7.716
7.704
7.701
7.624
7.605
7.539
7.533
7.516
7.499
7.494
7.367
7.354
7.336
7.328
7.316
7.285
7.260
7.232
7.229
7.213
7.209
7.200
7.180
7.161
6.966
6.945
4.480
4.461
4.441
4.420
Br
3i
1.0
2.0
0.9
ppm
9
8
7
6
5
4
3
2
1
0

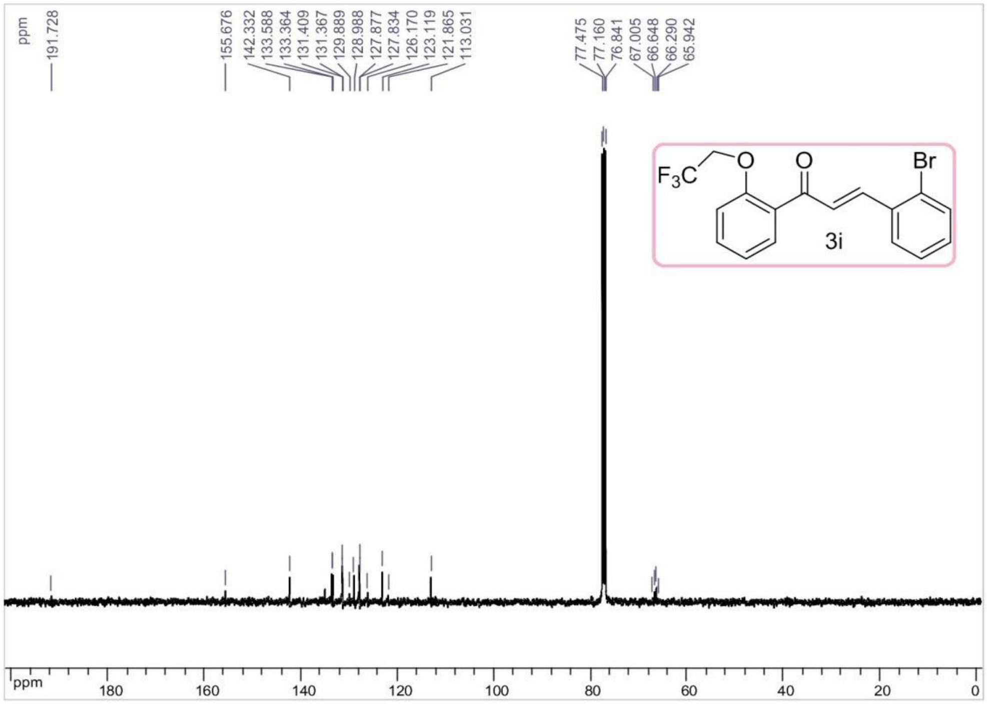
ppm
191.728
155.676
142.332
133.588
133.364
131.409
131.367
129.889
128.988
127.877
127.834
126.170
123.119
121.865
113.031
77.475
77.160
76.841
67.005
66.648
66.290
65.942
Br
3i
ppm
180
160
140
120
100
80
60
40
20
0

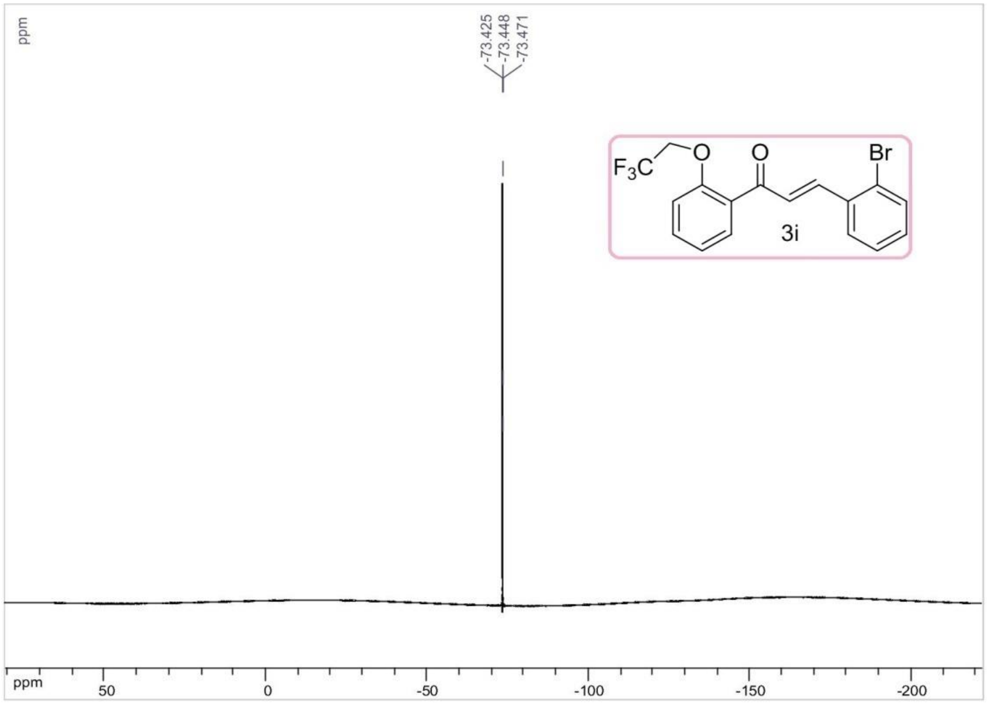
ppm
-73.425
-73.448
-73.471
F3C
O
O
Br
3i
ppm
50
0
-50
-100
-150
-200

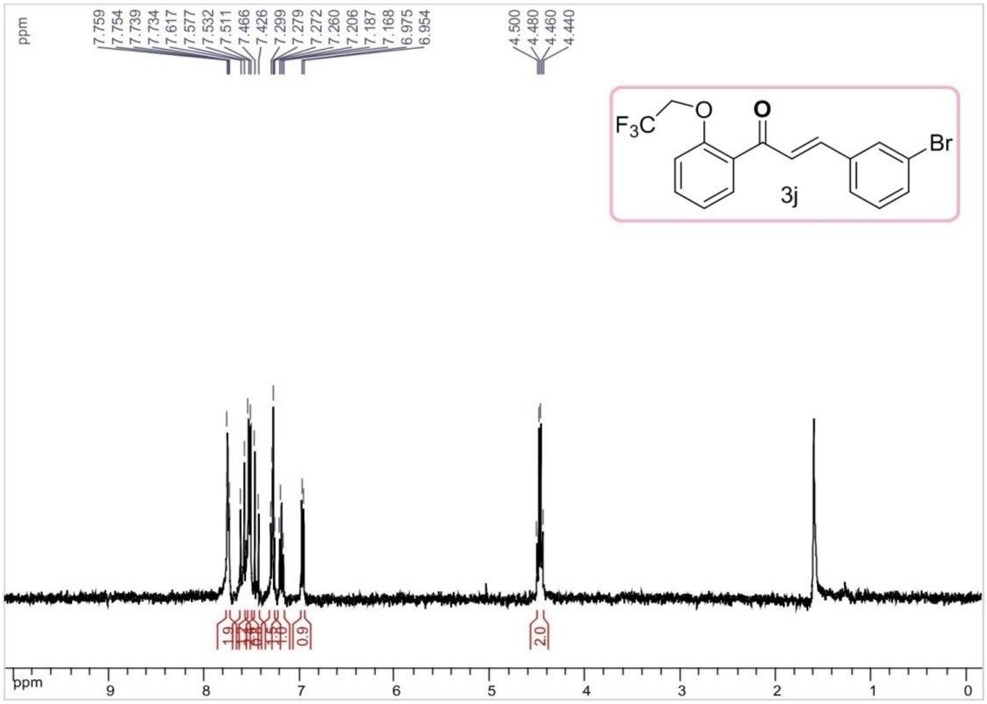
ppm
7.759
7.754
7.739
7.734
7.617
7.577
7.532
7.511
7.466
7.426
7.299
7.279
7.272
7.260
7.206
7.187
7.168
6.975
6.954
4.500
4.480
4.460
4.440
F3C
O
O
Br
3j
1.9
0.9
2.0
ppm
9
8
7
6
5
4
3
2
1
0

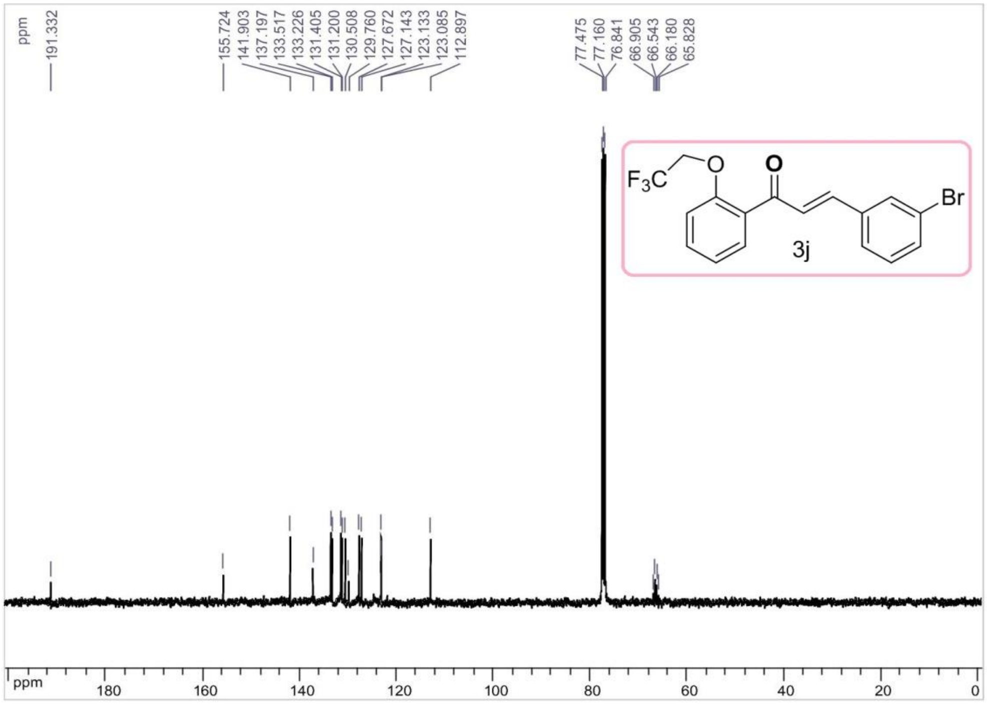
ppm
191.332
155.724
141.903
137.197
133.517
133.226
131.405
131.200
130.508
129.760
127.672
127.143
123.133
123.085
112.897
77.475
77.160
76.841
66.905
66.543
66.180
65.828
3j
ppm
180
160
140
120
100
80
60
40
20
0

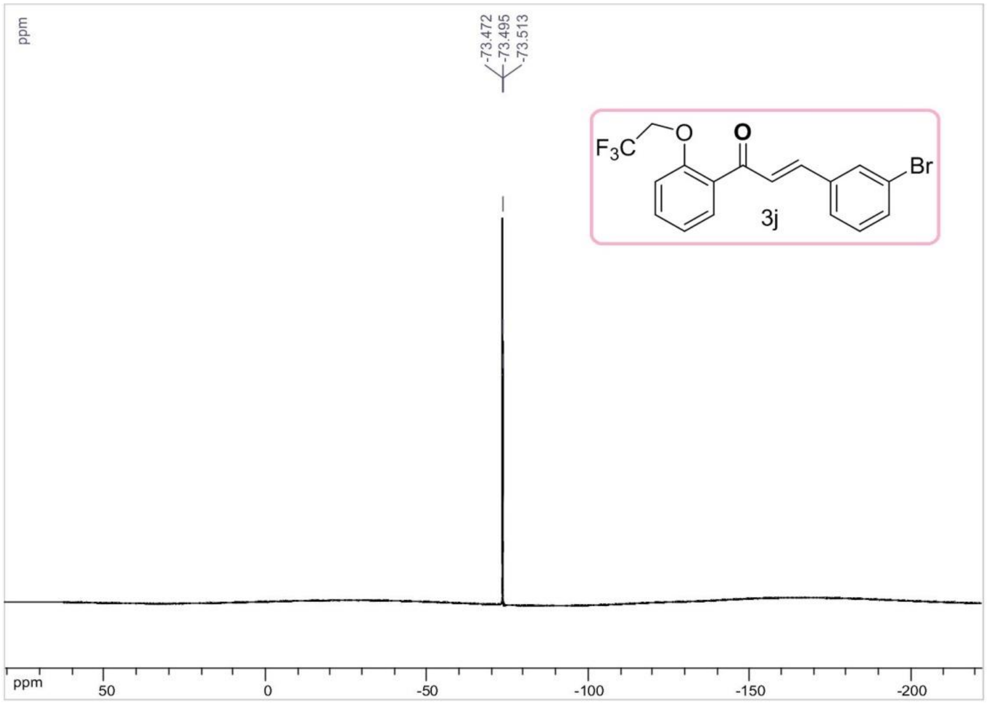
ppm
-73.472
-73.495
-73.513
3j
ppm
50
0
-50
-100
-150
-200

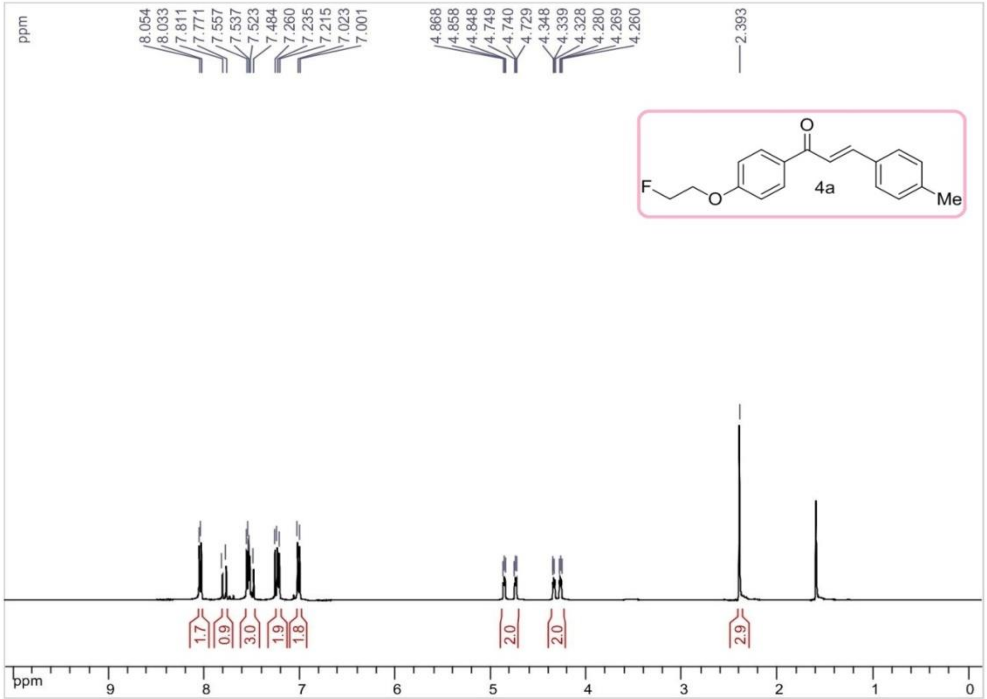
ppm
8.054
8.033
7.811
7.771
7.557
7.537
7.523
7.484
7.260
7.235
7.215
7.023
7.001
4.868
4.858
4.848
4.749
4.740
4.729
4.348
4.339
4.328
4.280
4.269
4.260
2.393
O
F
O
4a
Me
1.7
0.9
3.0
1.9
1.8
2.0
2.0
2.9
ppm
9
8
7
6
5
4
3
2
1
0

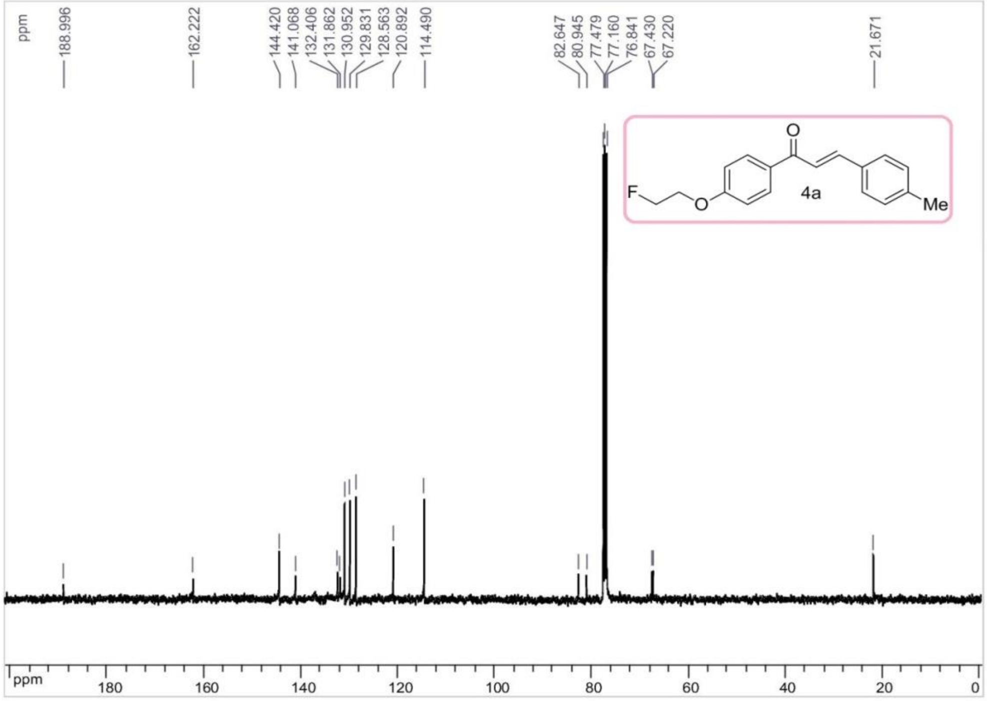
ppm
188.996
162.222
144.420
141.068
132.406
131.862
130.952
129.831
128.563
120.892
114.490
82.647
80.945
77.479
77.160
76.841
67.430
67.220
21.671
O
F
O
4a
Me
ppm
180
160
140
120
100
80
60
40
20
0

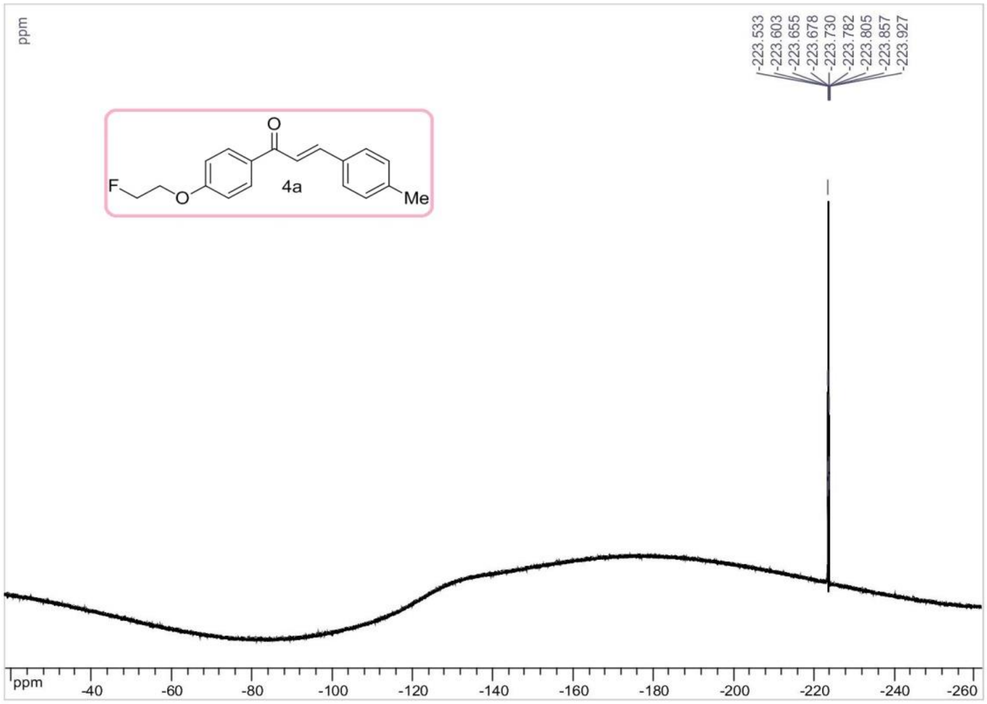

ppm
-223.533
-223.603
-223.655
-223.678
-223.730
-223.782
-223.805
-223.857
-223.927
F
O
O
4a
Me
ppm
-40
-60
-80
-100
-120
-140
-160
-180
-200
-220
-240
-260

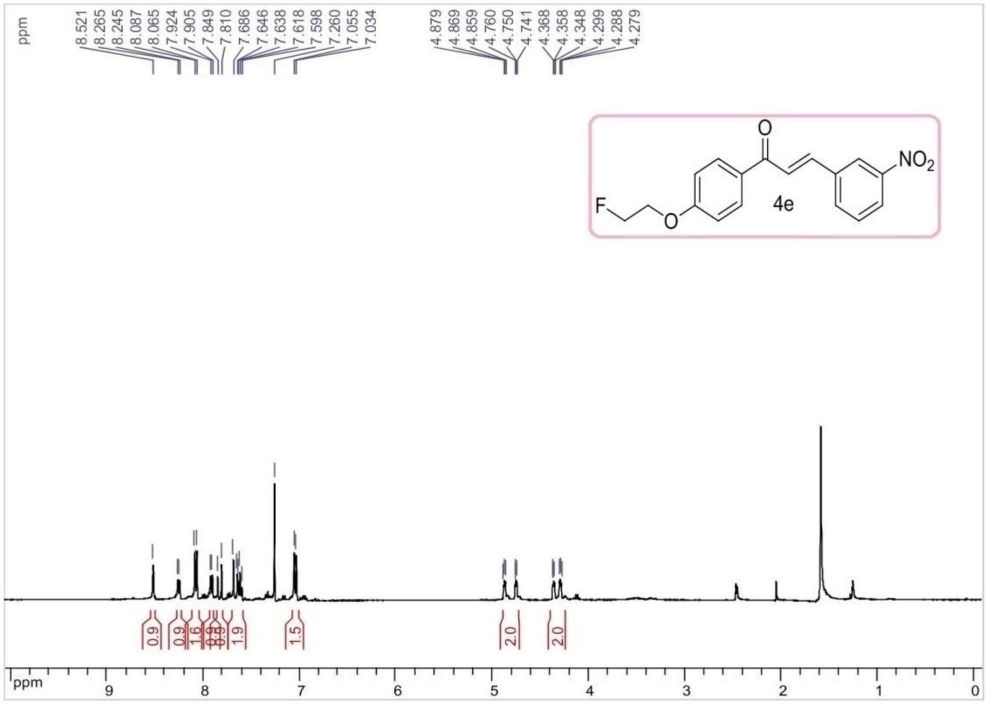

ppm
8.521
8.265
8.245
8.087
8.065
7.924
7.905
7.849
7.810
7.686
7.646
7.638
7.618
7.598
7.260
7.055
7.034
4.879
4.869
4.859
4.760
4.750
4.741
4.368
4.358
4.348
4.299
4.288
4.279
O
NO_2
4e
F
O
0.9
0.9
1.6
1.9
1.5
2.0
2.0
ppm
9
8
7
6
5
4
3
2
1
0

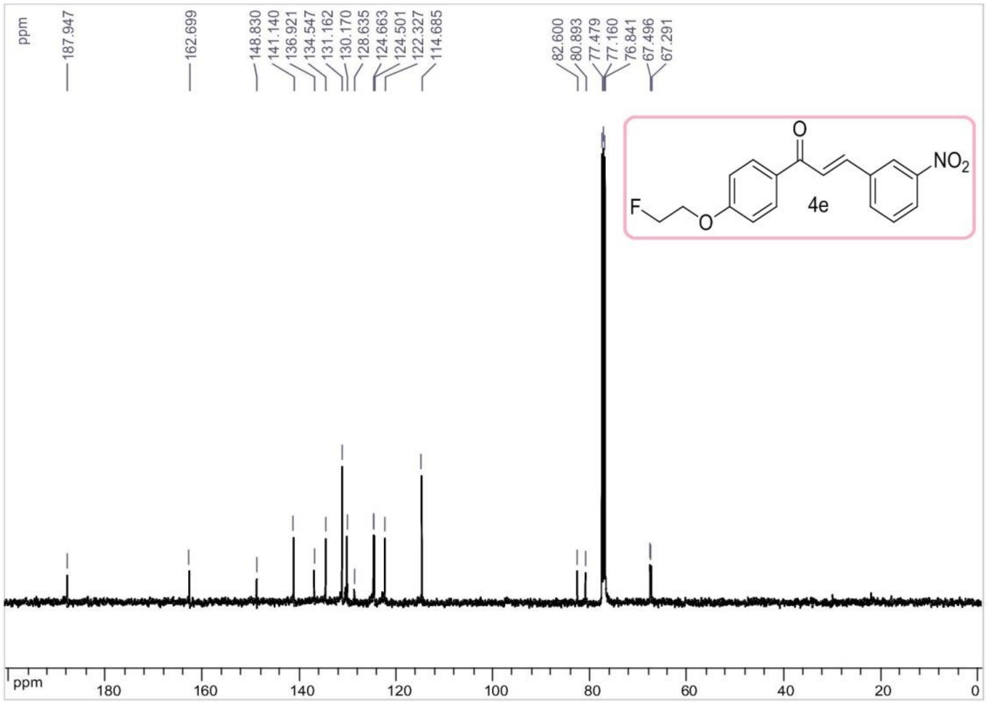
ppm
187.947
162.699
148.830
141.140
136.921
134.547
131.162
130.170
128.635
124.663
124.501
122.327
114.685
82.600
80.893
77.479
77.160
76.841
67.496
67.291
O
NO2
F
O
4e
ppm
180
160
140
120
100
80
60
40
20
0

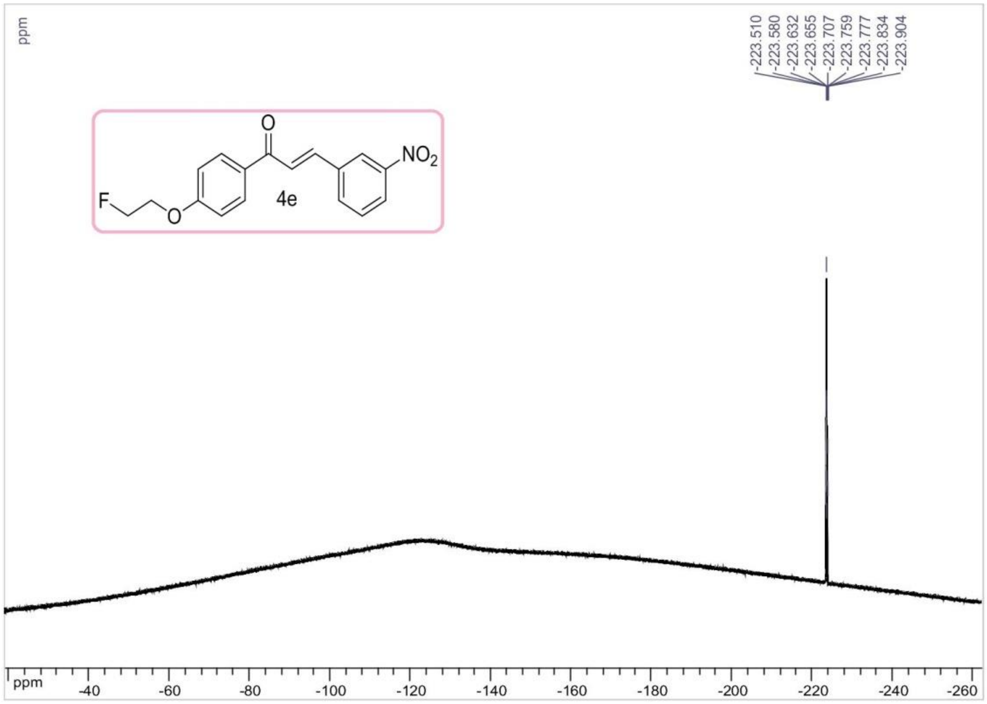
ppm
-223.510
-223.580
-223.632
-223.655
-223.707
-223.759
-223.777
-223.834
-223.904
O
NO2
F
O
4e
ppm
-40
-60
-80
-100
-120
-140
-160
-180
-200
-220
-240
-260

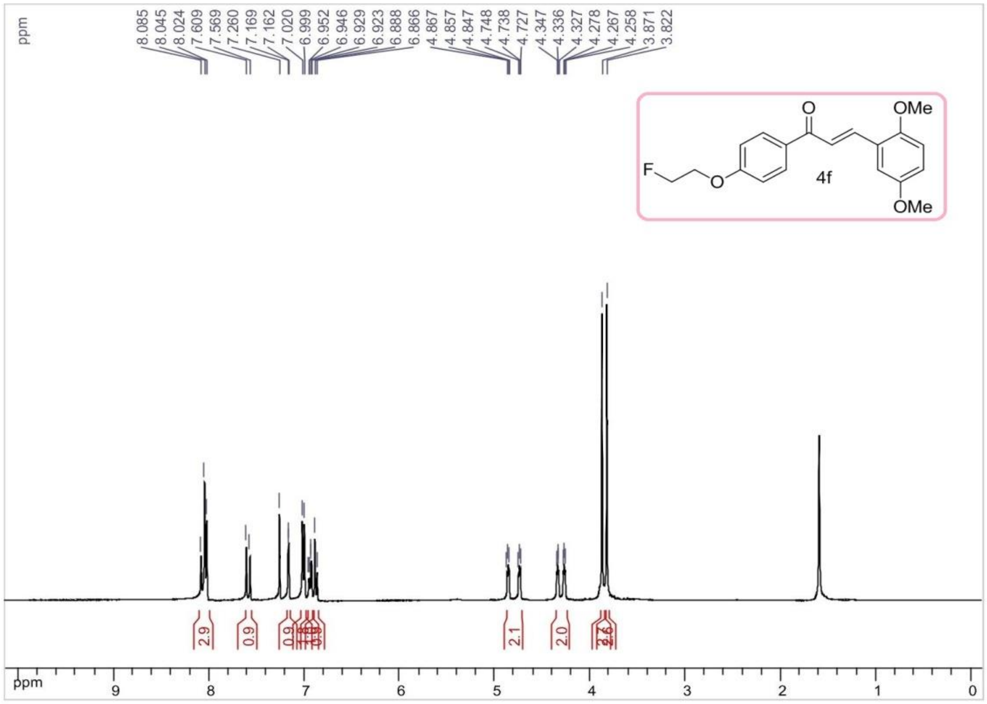
ppm
8.085
8.045
8.024
7.609
7.569
7.260
7.169
7.162
7.020
6.999
6.952
6.946
6.929
6.923
6.888
6.866
4.867
4.857
4.847
4.748
4.738
4.727
4.347
4.336
4.327
4.278
4.267
4.258
3.871
3.822
OMe
F
O
4f
OMe
2.9
0.9
0.9
2.1
2.0
ppm
9
8
7
6
5
4
3
2
1
0

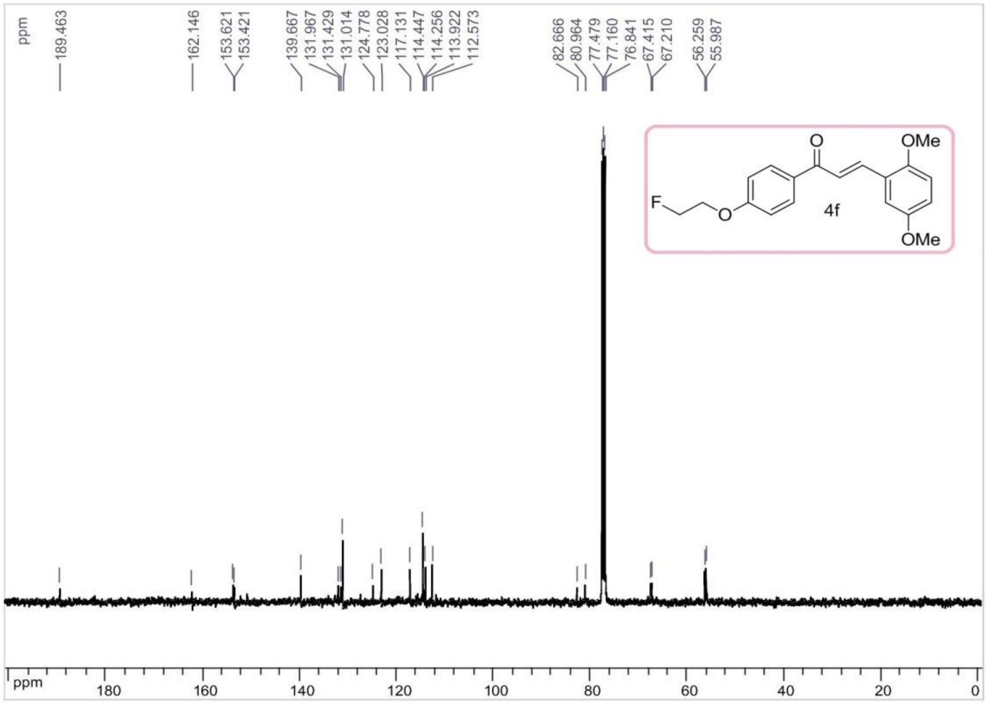
ppm
189.463
162.146
153.621
153.421
139.667
131.967
131.429
131.014
124.778
123.028
117.131
114.447
114.256
113.922
112.573
82.666
80.964
77.479
77.160
76.841
67.415
67.210
56.259
55.987
O
OMe
F
O
4f
OMe
ppm
180
160
140
120
100
80
60
40
20
0

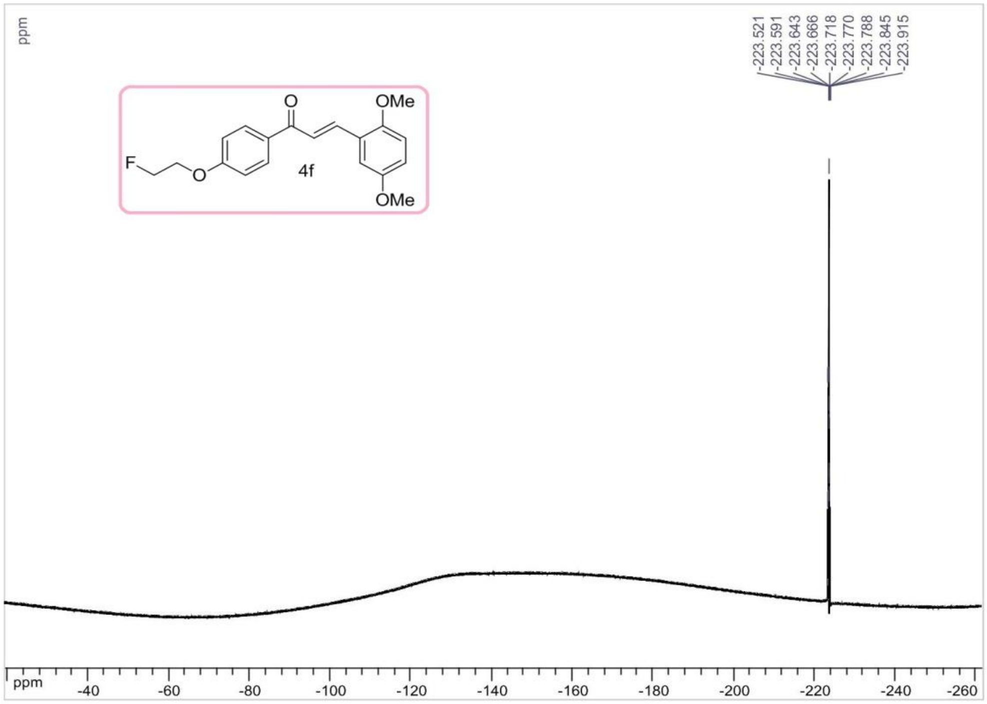
ppm
-223.521
-223.591
-223.643
-223.666
-223.718
-223.770
-223.788
-223.845
-223.915
OMe
F
O
4f
OMe
ppm -40 -60 -80 -100 -120 -140 -160 -180 -200 -220 -240 -260

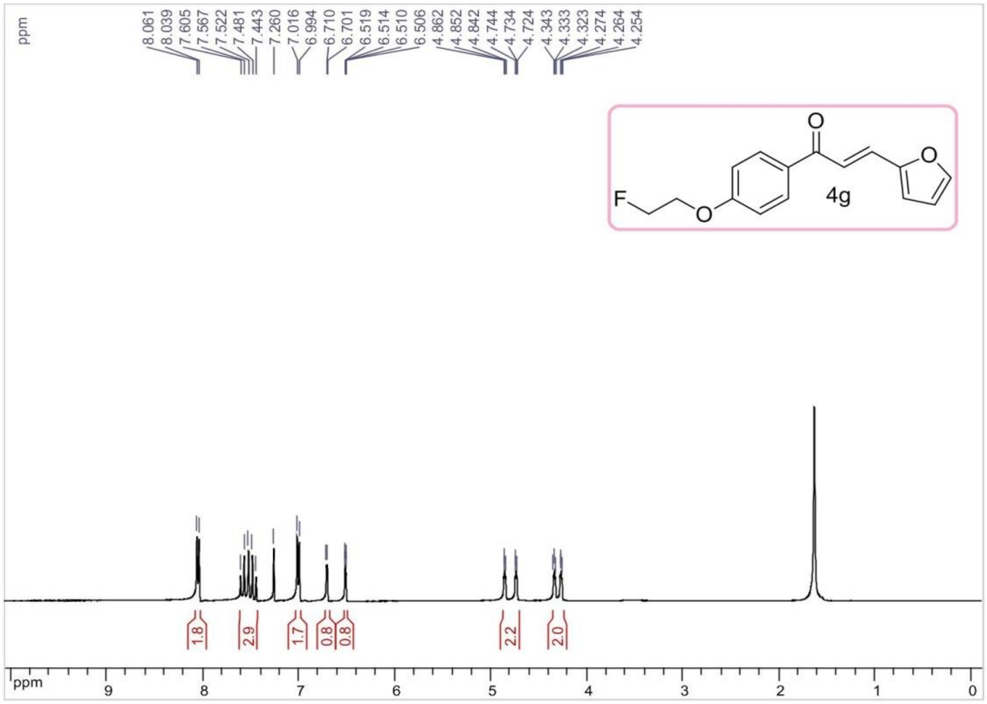
ppm
8.061
8.039
7.605
7.567
7.522
7.481
7.443
7.260
7.016
6.994
6.710
6.701
6.519
6.514
6.510
6.506
4.862
4.852
4.842
4.744
4.734
4.724
4.343
4.333
4.323
4.274
4.264
4.254
O
F
O
4g
1.8
2.9
1.7
0.8
0.8
2.2
2.0
ppm 9 8 7 6 5 4 3 2 1 0

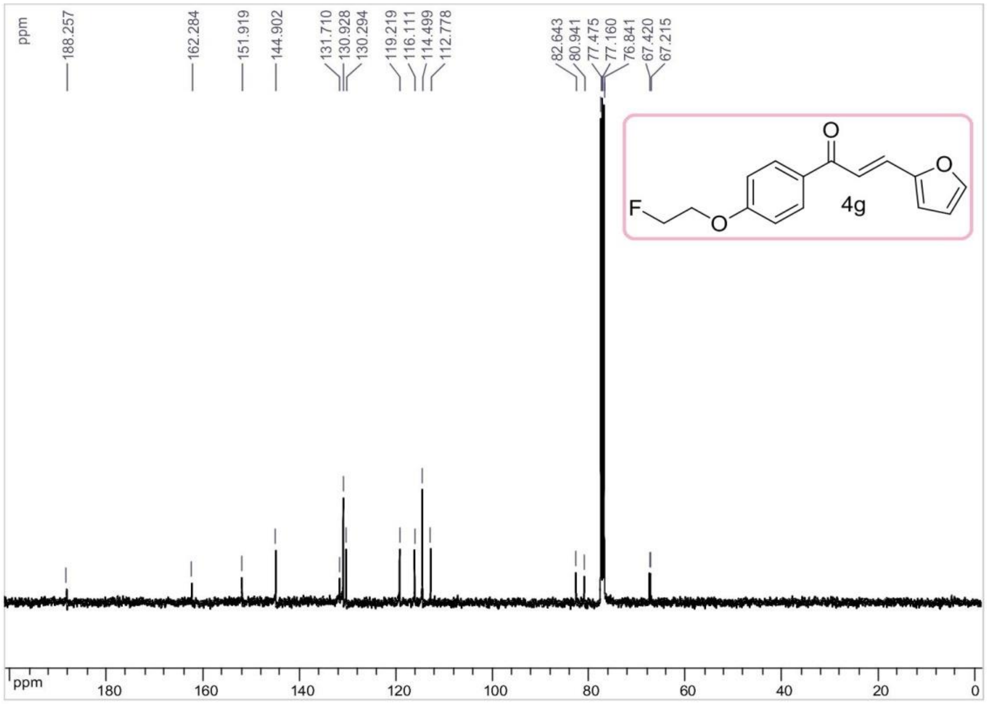
ppm
188.257
162.284
151.919
144.902
131.710
130.928
130.294
119.219
116.111
114.499
112.778
82.643
80.941
77.475
77.160
76.841
67.420
67.215
O
F
O
4g
O
ppm
180
160
140
120
100
80
60
40
20
0

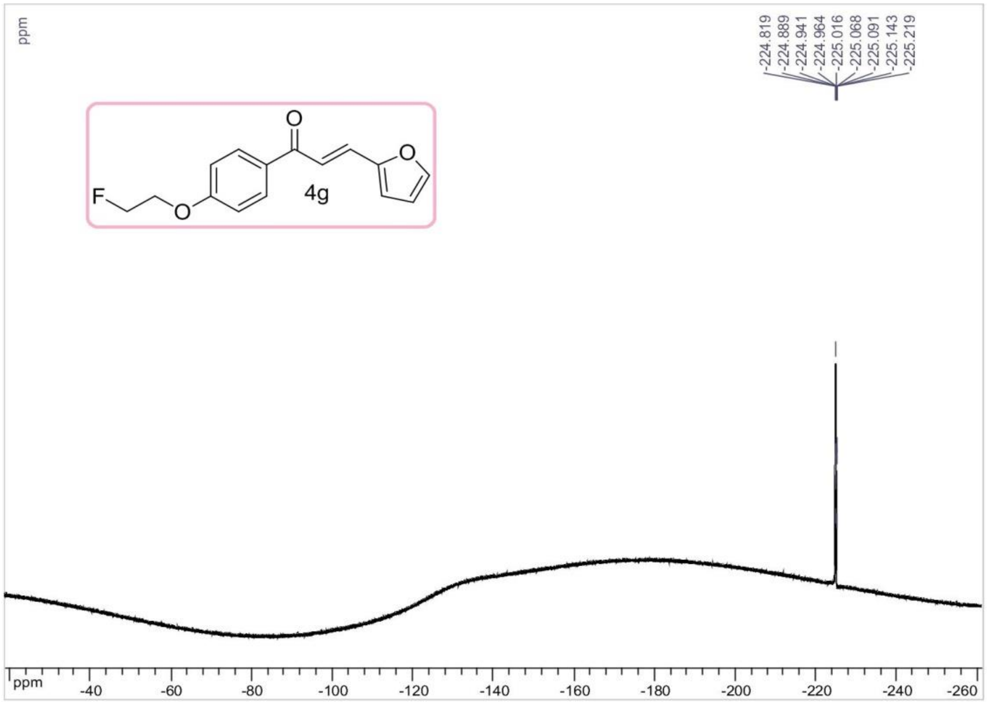
ppm
-224.819
-224.889
-224.941
-224.964
-225.016
-225.068
-225.091
-225.143
-225.219
O
F
O
4g
O
ppm
-40
-60
-80
-100
-120
-140
-160
-180
-200
-220
-240
-260

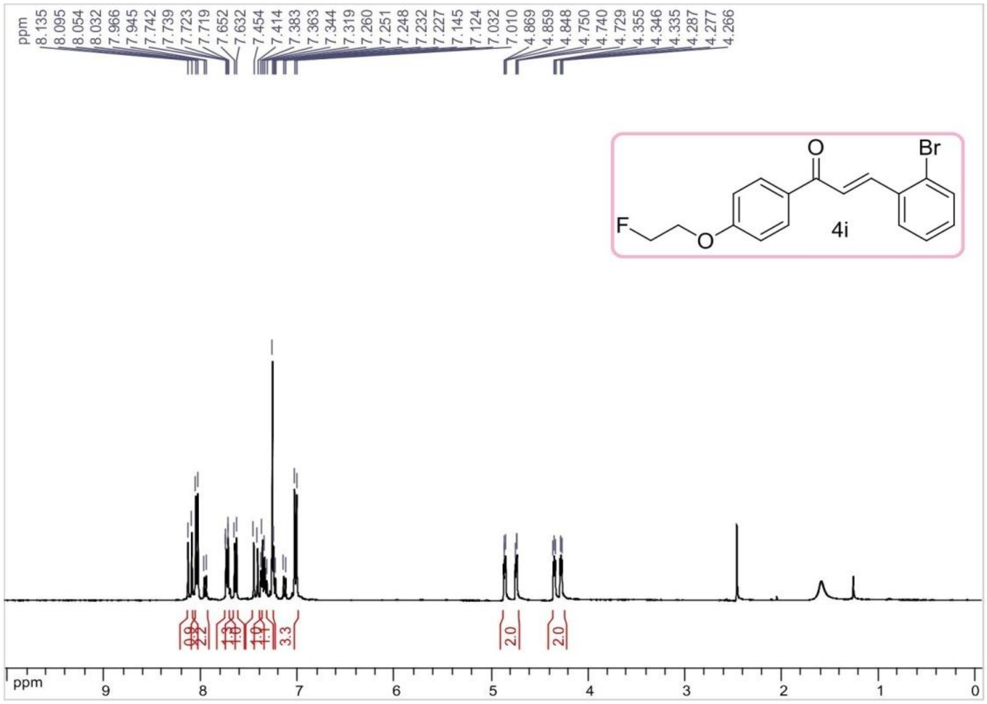
ppm
8.135
8.095
8.054
8.032
7.966
7.945
7.742
7.739
7.723
7.719
7.652
7.632
7.454
7.414
7.383
7.363
7.344
7.319
7.260
7.251
7.248
7.232
7.227
7.145
7.124
7.032
7.010
4.869
4.859
4.848
4.750
4.740
4.729
4.355
4.346
4.335
4.287
4.277
4.266
F
O
O
Br
4i
2.0
2.0
3.3
ppm
9
8
7
6
5
4
3
2
1
0

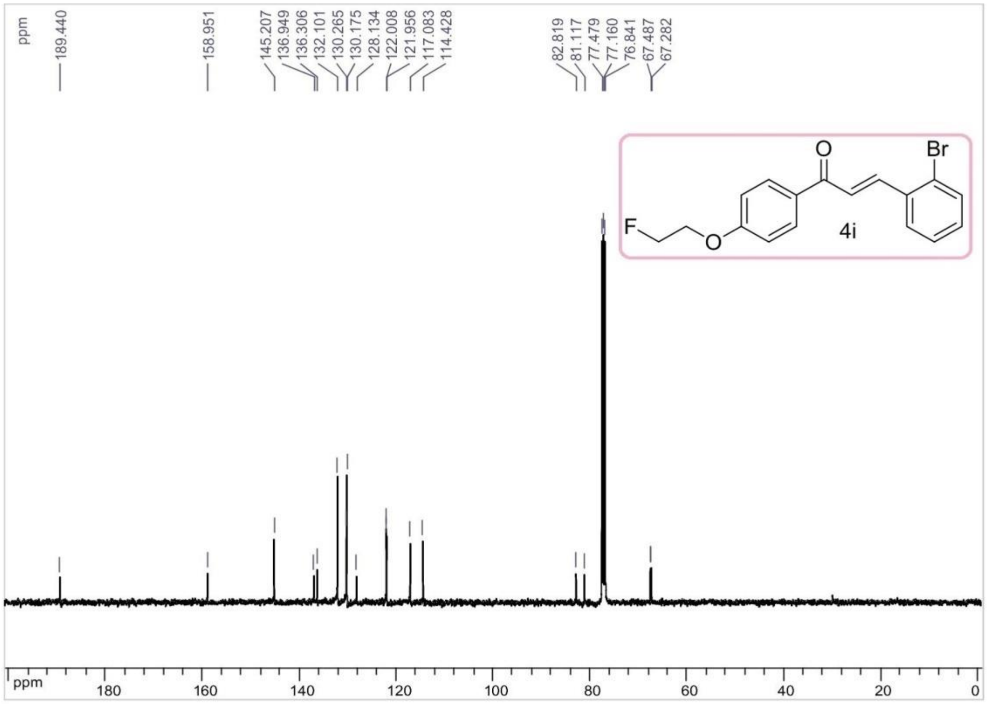
ppm
189.440
158.951
145.207
136.949
136.306
132.101
130.265
130.175
128.134
122.008
121.956
117.083
114.428
82.819
81.117
77.479
77.160
76.841
67.487
67.282
F
O
O
Br
4i
ppm
180
160
140
120
100
80
60
40
20
0

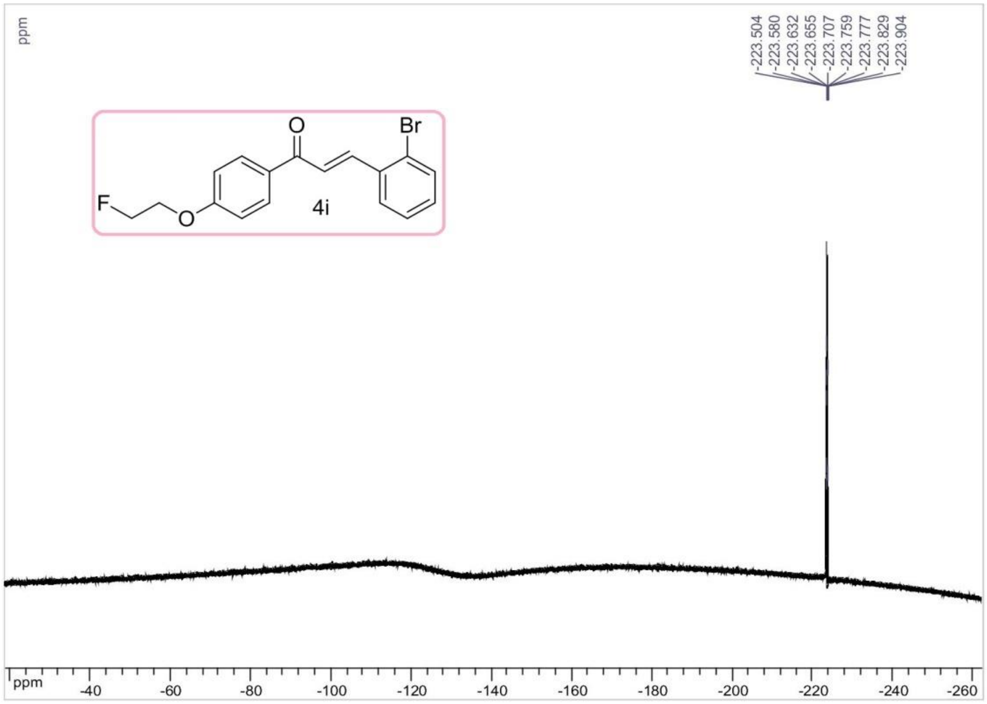

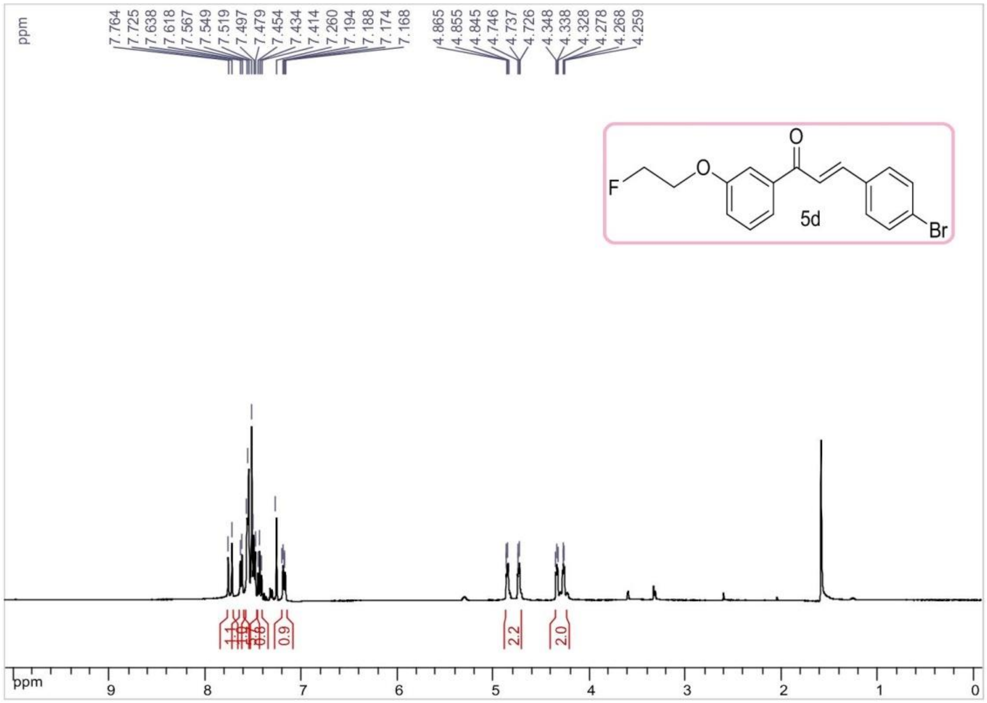

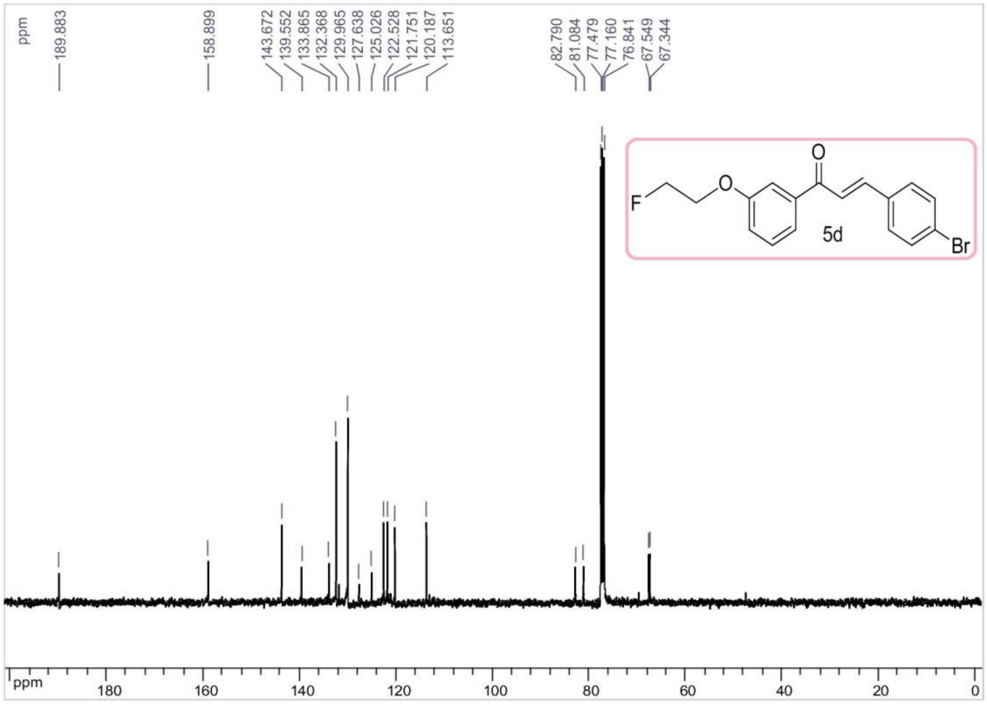
5d
ppm
189.883
158.899
143.672
139.552
133.865
132.368
129.965
127.638
125.026
122.528
121.751
120.187
113.651
82.790
81.084
77.479
77.160
76.841
67.549
67.344
180
160
140
120
100
80
60
40
20
0

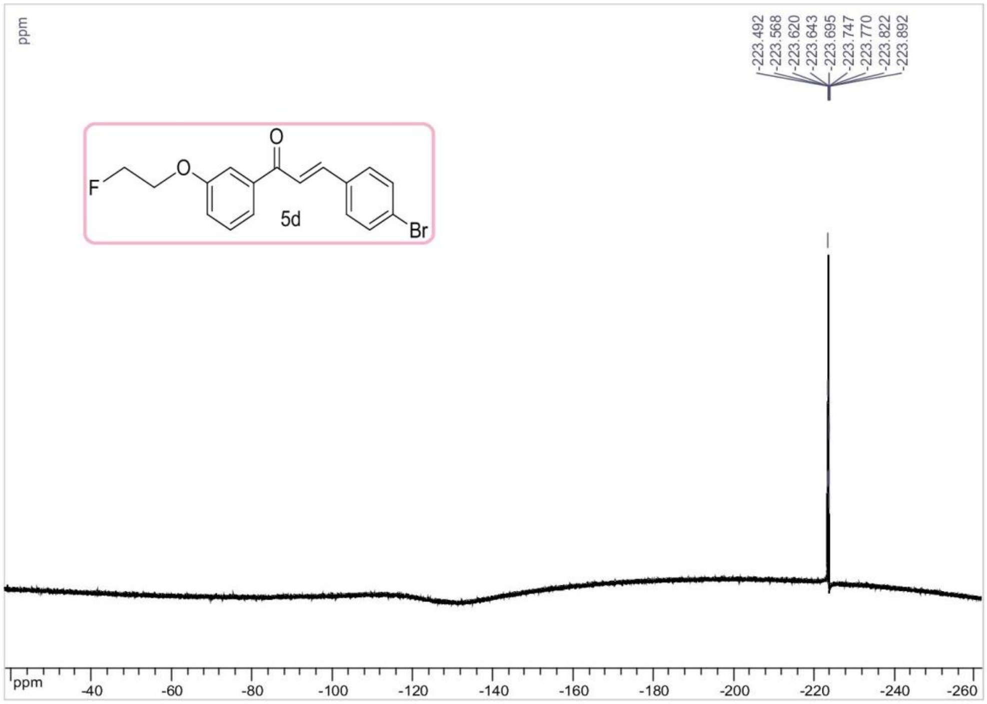
5d
ppm
-223.492
-223.568
-223.620
-223.643
-223.695
-223.747
-223.770
-223.822
-223.892
-40
-60
-80
-100
-120
-140
-160
-180
-200
-220
-240
-260

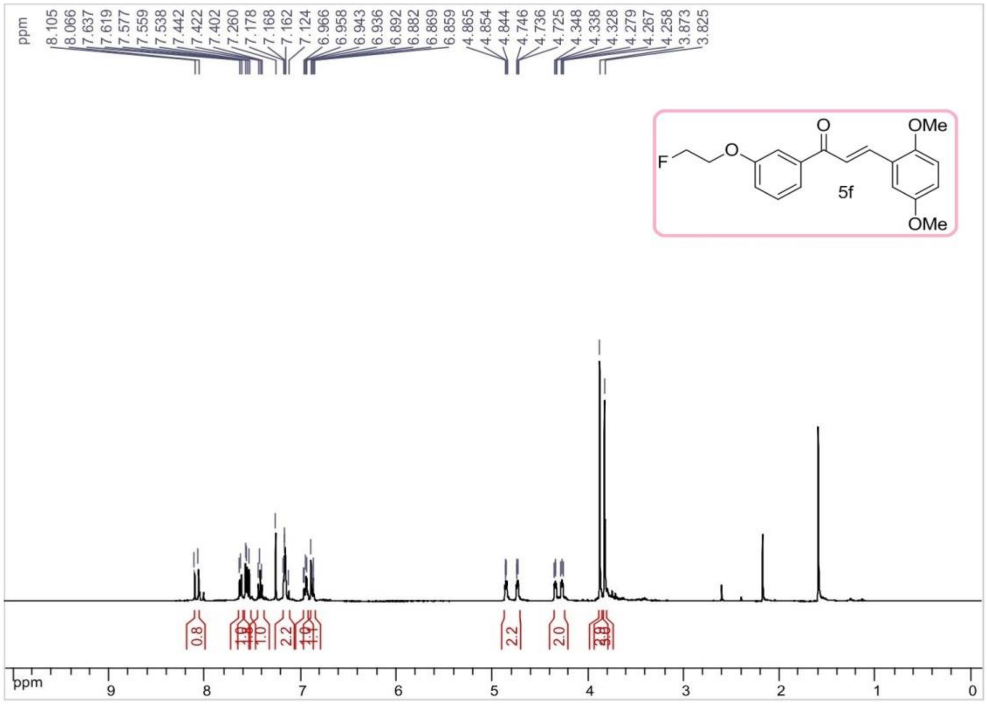
ppm
8.105
8.066
7.637
7.619
7.577
7.559
7.538
7.442
7.422
7.402
7.260
7.178
7.168
7.162
7.124
6.966
6.958
6.943
6.936
6.892
6.882
6.869
6.859
4.865
4.854
4.844
4.746
4.736
4.725
4.348
4.338
4.328
4.279
4.267
4.258
3.873
3.825
O
OMe
F
O
5f
OMe
0.8
2.2
2.0
2.2
ppm
9
8
7
6
5
4
3
2
1
0

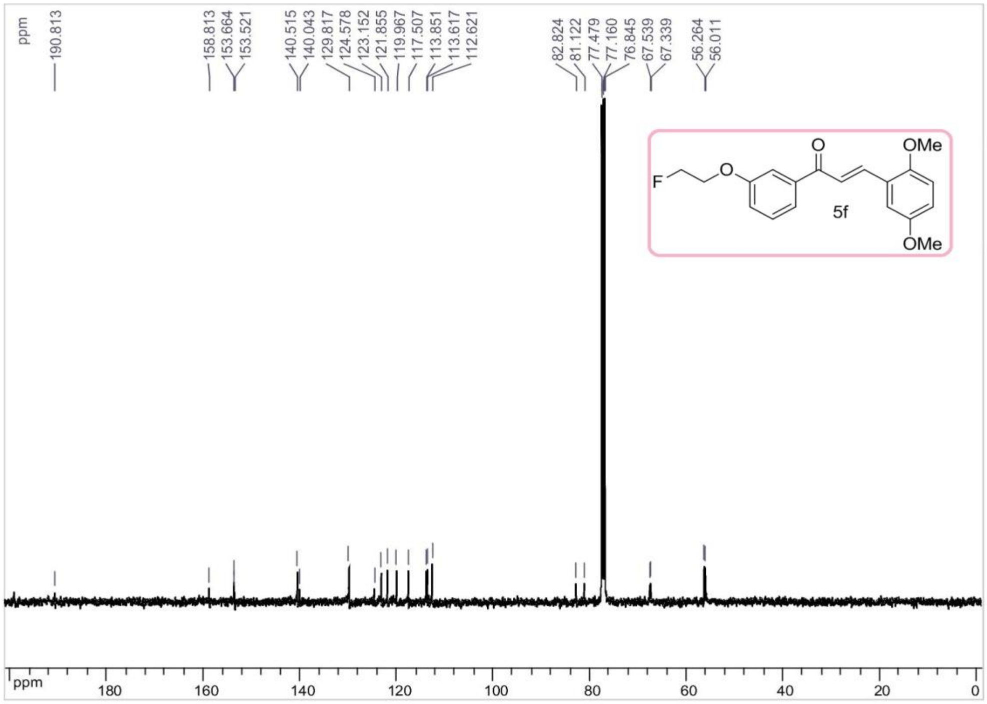
ppm
190.813
158.813
153.664
153.521
140.515
140.043
129.817
124.578
123.152
121.855
119.967
117.507
113.851
113.617
112.621
82.824
81.122
77.479
77.160
76.845
67.539
67.339
56.264
56.011
O
OMe
F
O
5f
OMe
ppm
180
160
140
120
100
80
60
40
20
0

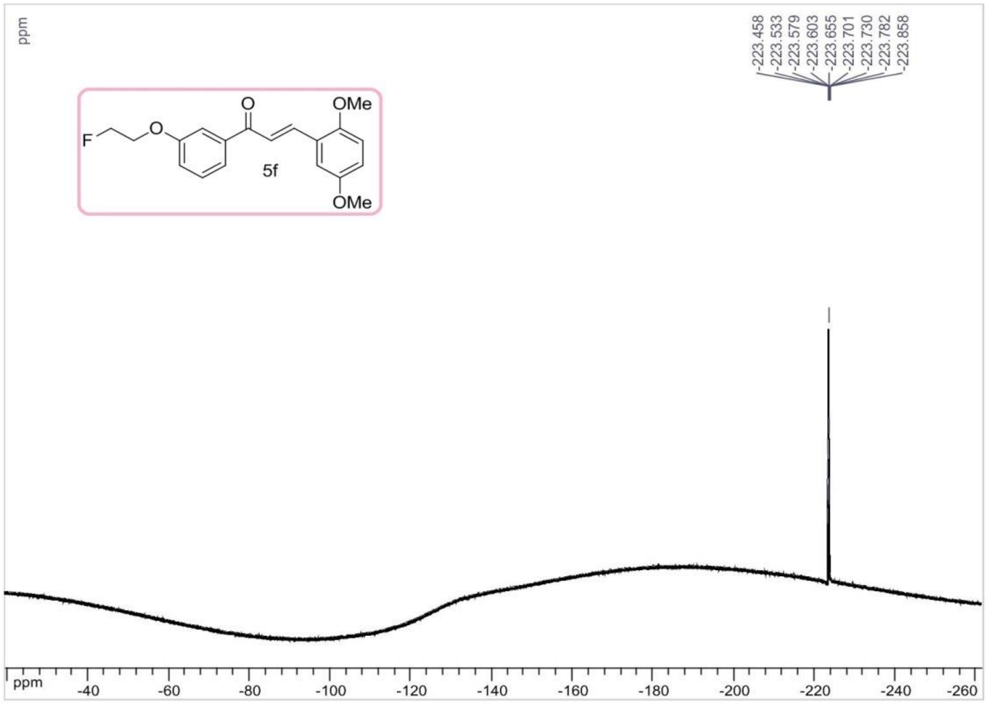

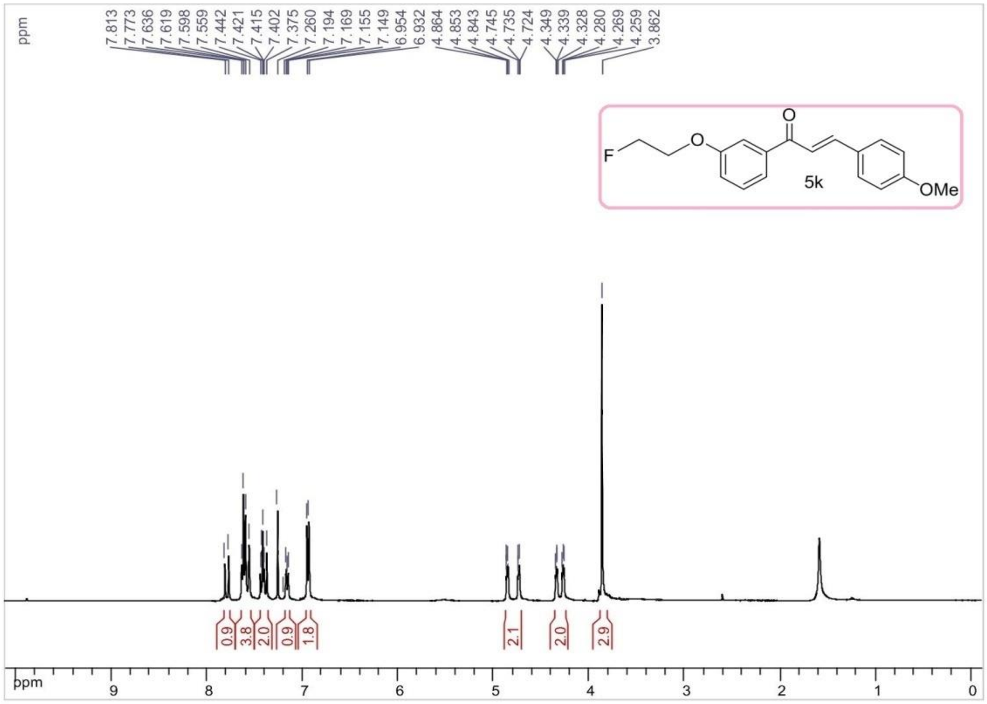

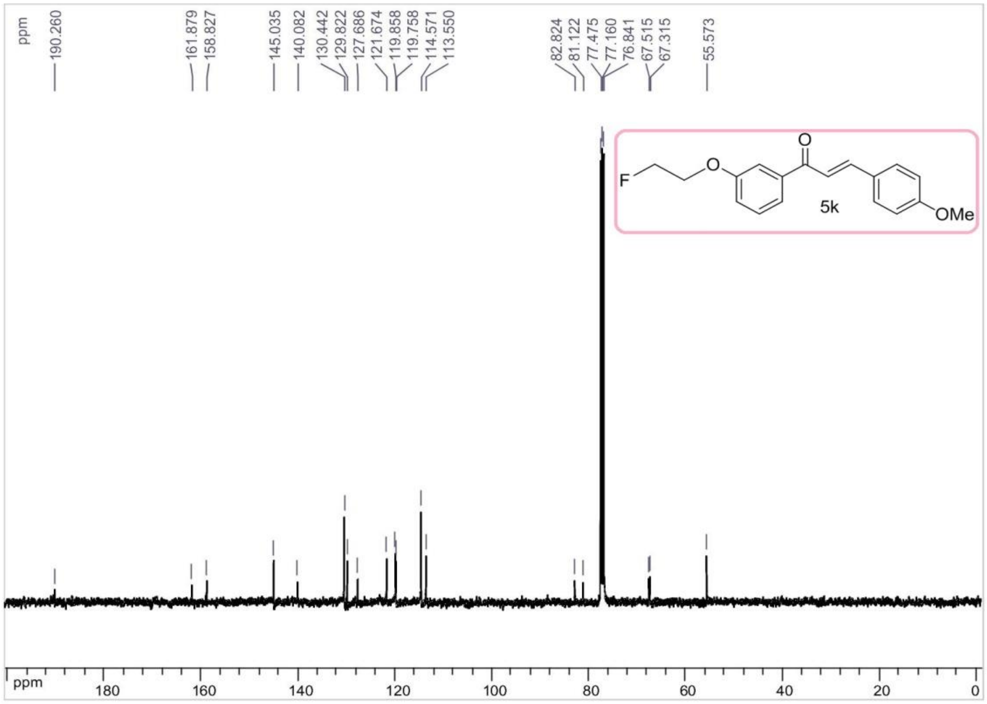
ppm
190.260
161.879
158.827
145.035
140.082
130.442
129.822
127.686
121.674
119.858
119.758
114.571
113.550
82.824
81.122
77.475
77.160
76.841
67.515
67.315
55.573
F
O
O
5k
OMe
ppm
180
160
140
120
100
80
60
40
20
0

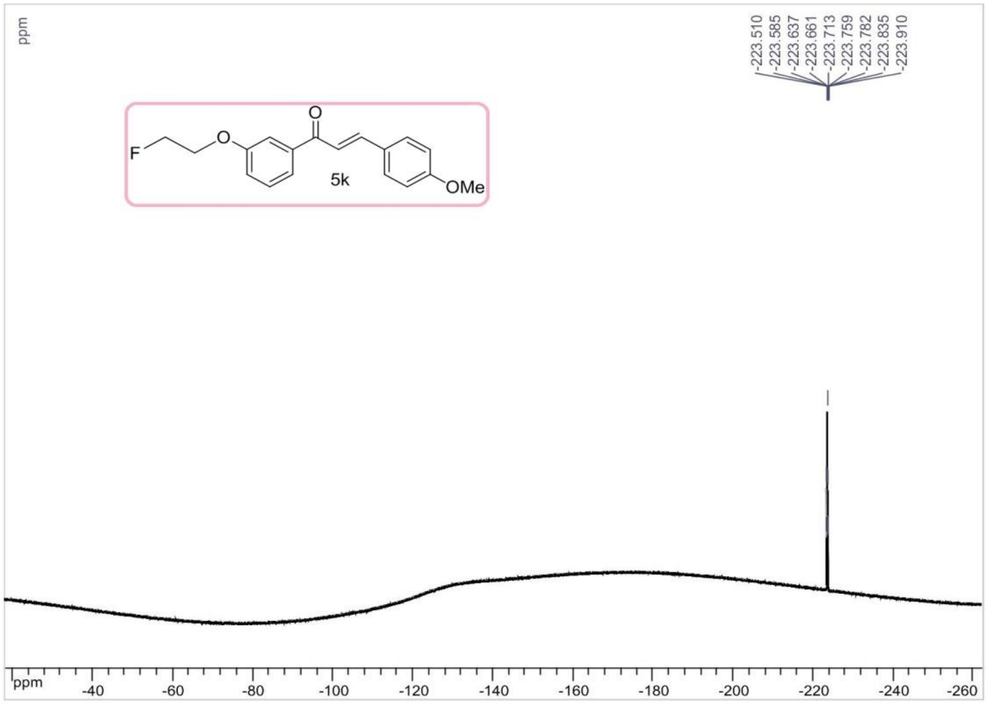
ppm
-223.510
-223.585
-223.637
-223.661
-223.713
-223.759
-223.782
-223.835
-223.910
F
O
O
5k
OMe
ppm
-40
-60
-80
-100
-120
-140
-160
-180
-200
-220
-240
-260

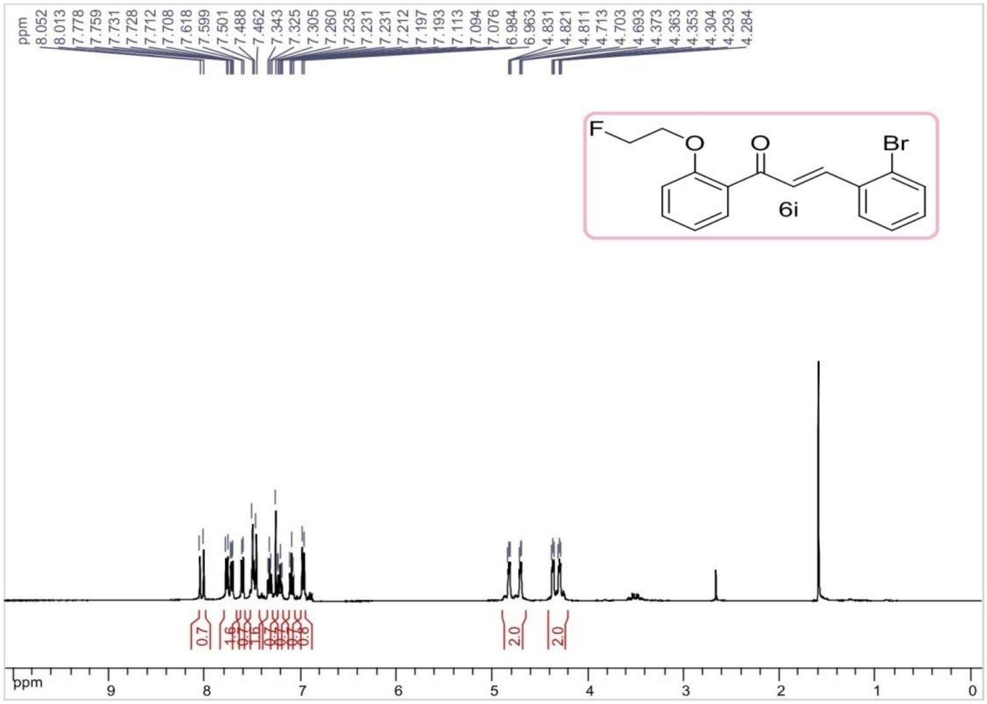
ppm
8.052
8.013
7.778
7.759
7.731
7.728
7.712
7.708
7.618
7.599
7.501
7.488
7.462
7.343
7.325
7.305
7.260
7.235
7.235
7.231
7.231
7.212
7.197
7.193
7.113
7.094
7.076
6.984
6.963
4.831
4.821
4.811
4.713
4.703
4.693
4.373
4.363
4.353
4.304
4.293
4.284
F
O
O
Br
6i
0.7
2.0
2.0
ppm
9
8
7
6
5
4
3
2
1
0

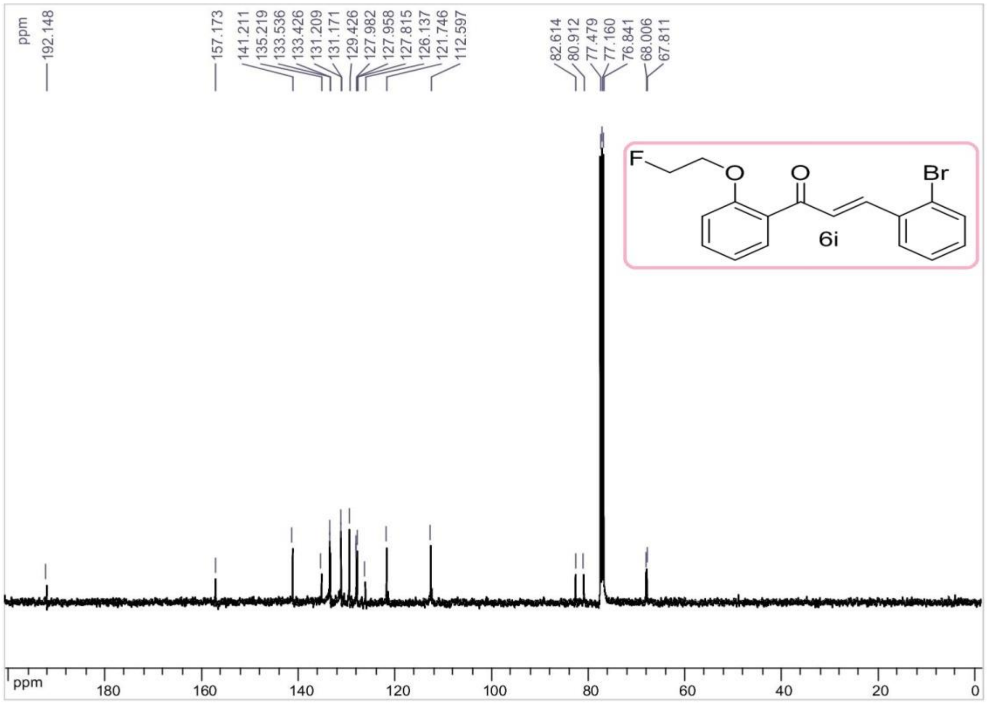
ppm
192.148
157.173
141.211
135.219
133.536
133.426
131.209
131.171
129.426
127.982
127.958
127.815
126.137
121.746
112.597
82.614
80.912
77.479
77.160
76.841
68.006
67.811
F
O
O
Br
6i
ppm
180
160
140
120
100
80
60
40
20
0

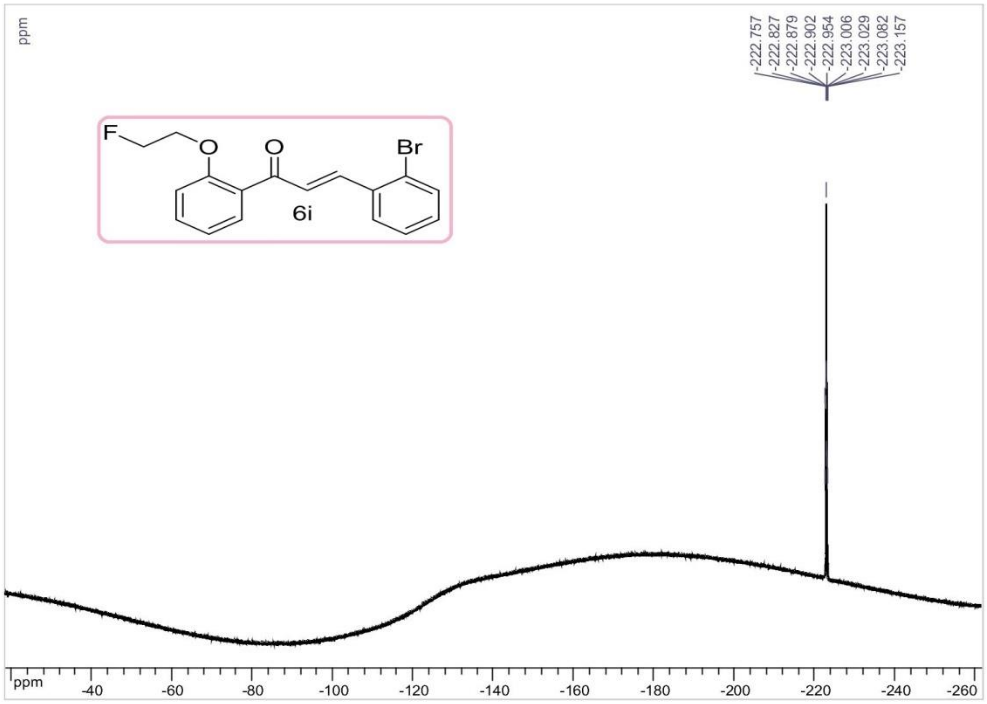

-222.757
-222.827
-222.879
-222.902
-222.954
-223.006
-223.029
-223.082
-223.157
F
O
O
Br
6i
ppm
-40
-60
-80
-100
-120
-140
-160
-180
-200
-220
-240
-260

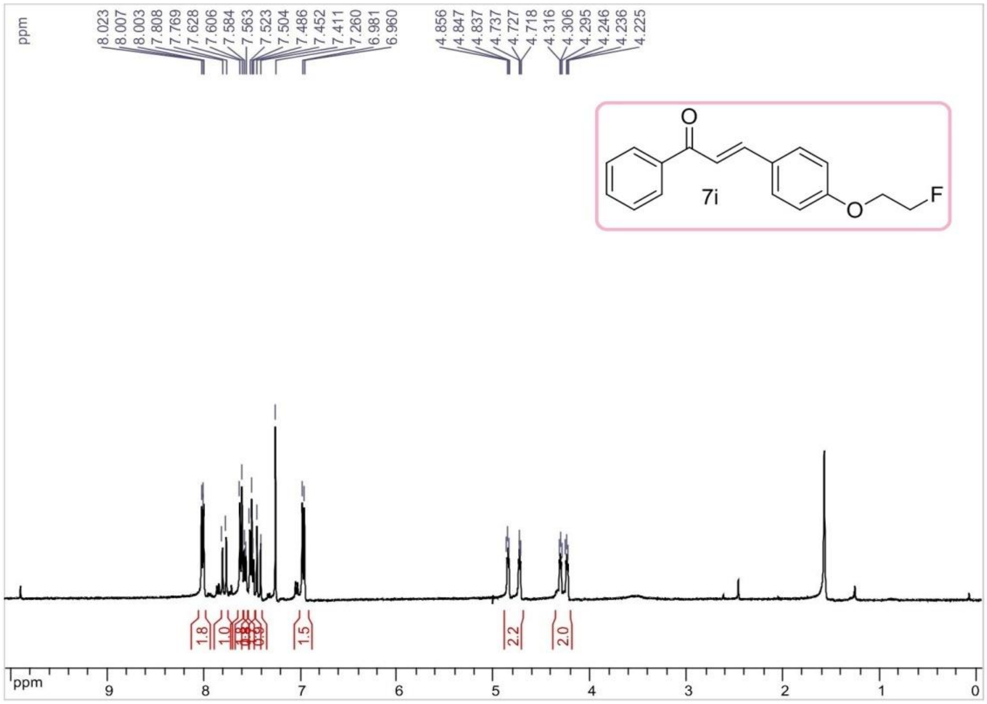

8.023
8.007
8.003
7.808
7.769
7.628
7.606
7.584
7.563
7.523
7.504
7.486
7.452
7.411
7.260
6.981
6.960
4.856
4.847
4.837
4.737
4.727
4.718
4.316
4.306
4.295
4.246
4.236
4.225
O
7i
O
F
1.8
1.0
1.5
2.2
2.0
ppm
9
8
7
6
5
4
3
2
1
0

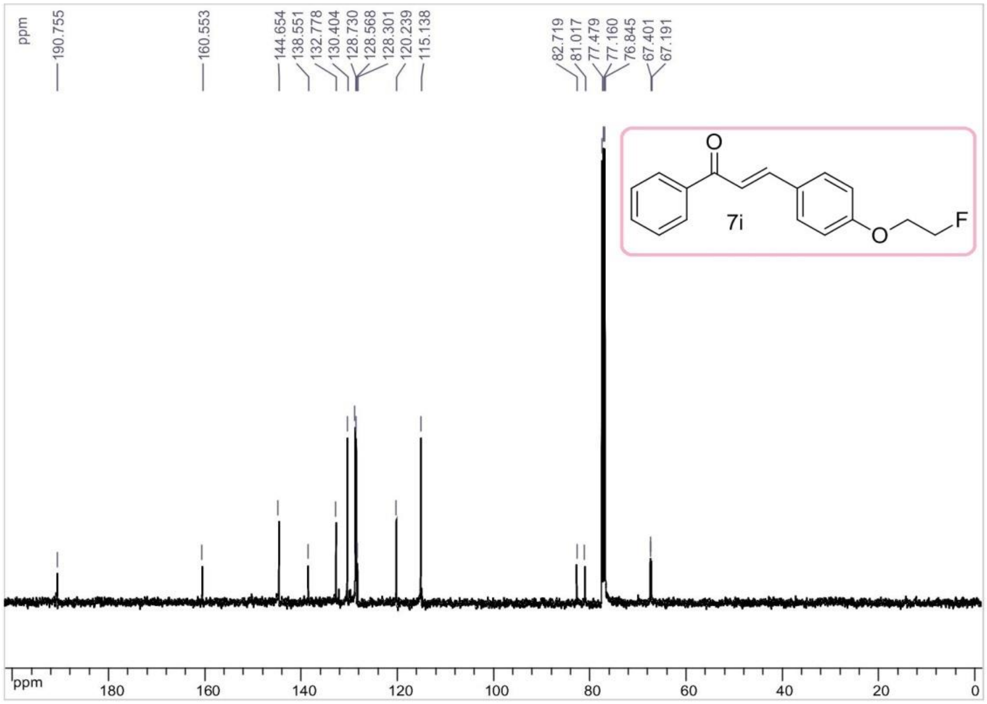
ppm
190.755
160.553
144.654
138.551
132.778
130.404
128.730
128.568
128.301
120.239
115.138
82.719
81.017
77.479
77.160
76.845
67.401
67.191
O
F
7i
ppm
180
160
140
120
100
80
60
40
20
0

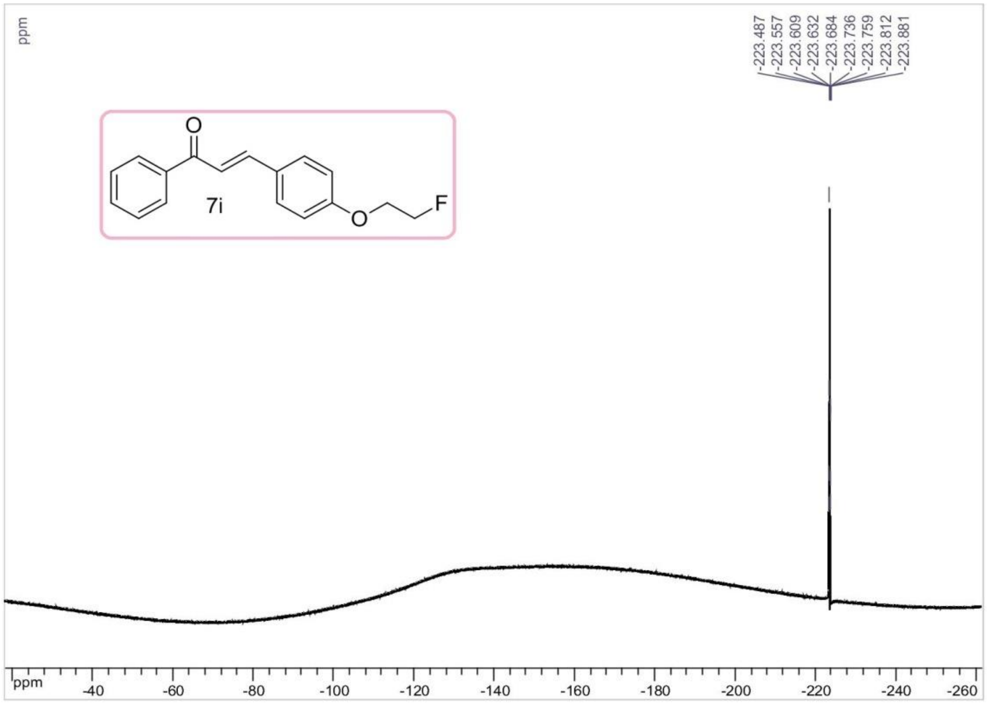
ppm
-223.487
-223.557
-223.609
-223.632
-223.684
-223.736
-223.759
-223.812
-223.881
O
O
F
7i
ppm
-40
-60
-80
-100
-120
-140
-160
-180
-200
-220
-240
-260

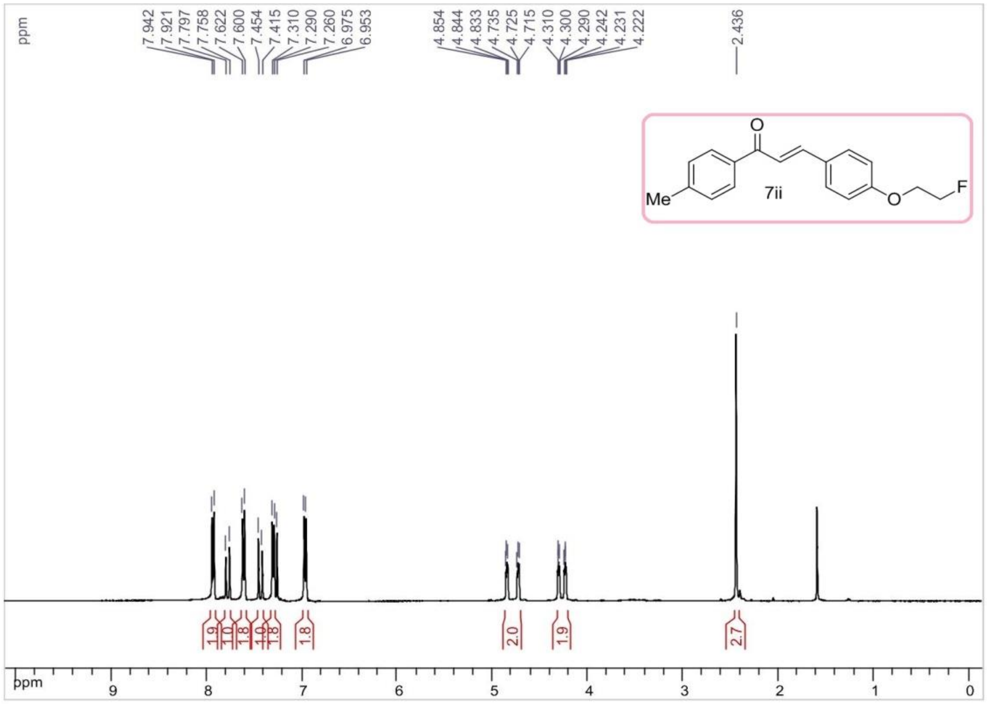
7ii
Me
O
F

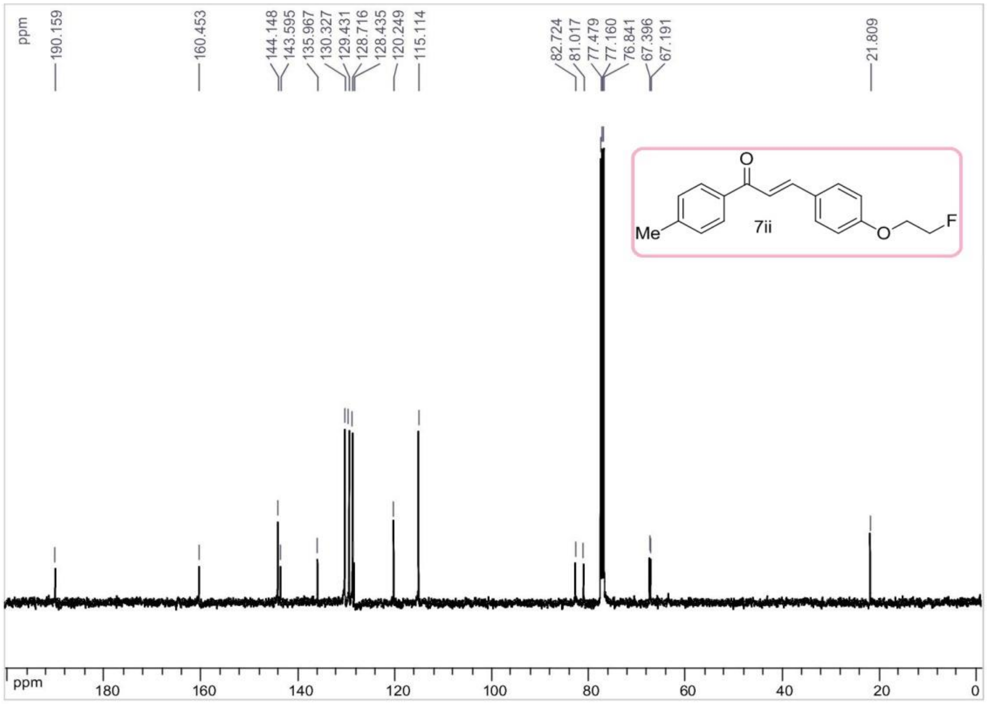
7ii
Me
O
F

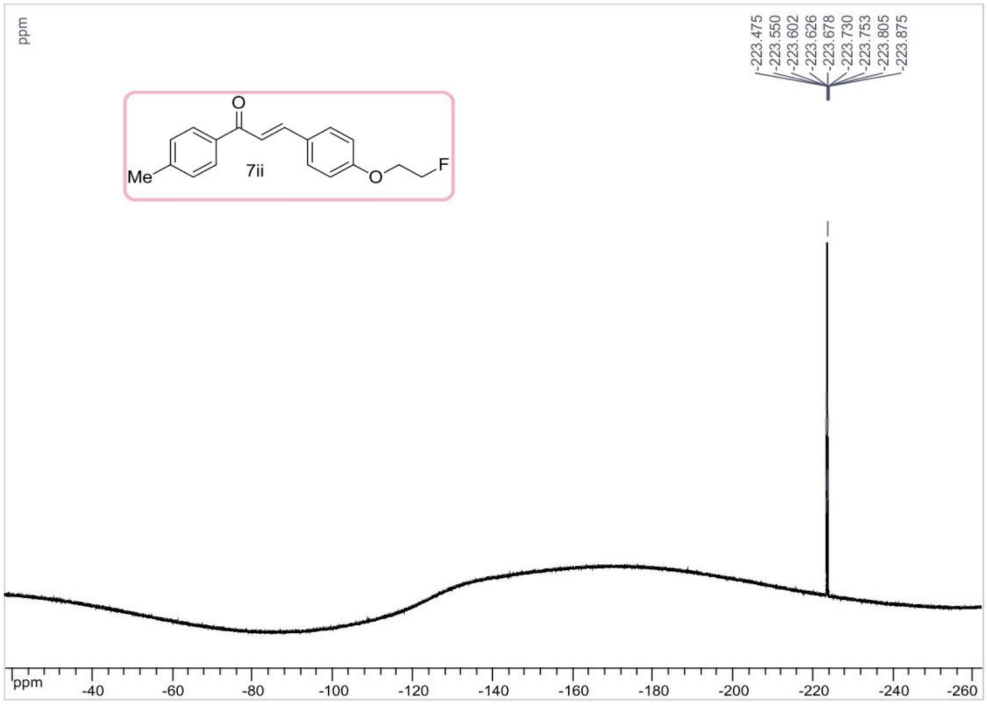
ppm
-223.475
-223.550
-223.602
-223.626
-223.678
-223.730
-223.753
-223.805
-223.875
Me
7ii
O
F
ppm
-40
-60
-80
-100
-120
-140
-160
-180
-200
-220
-240
-260

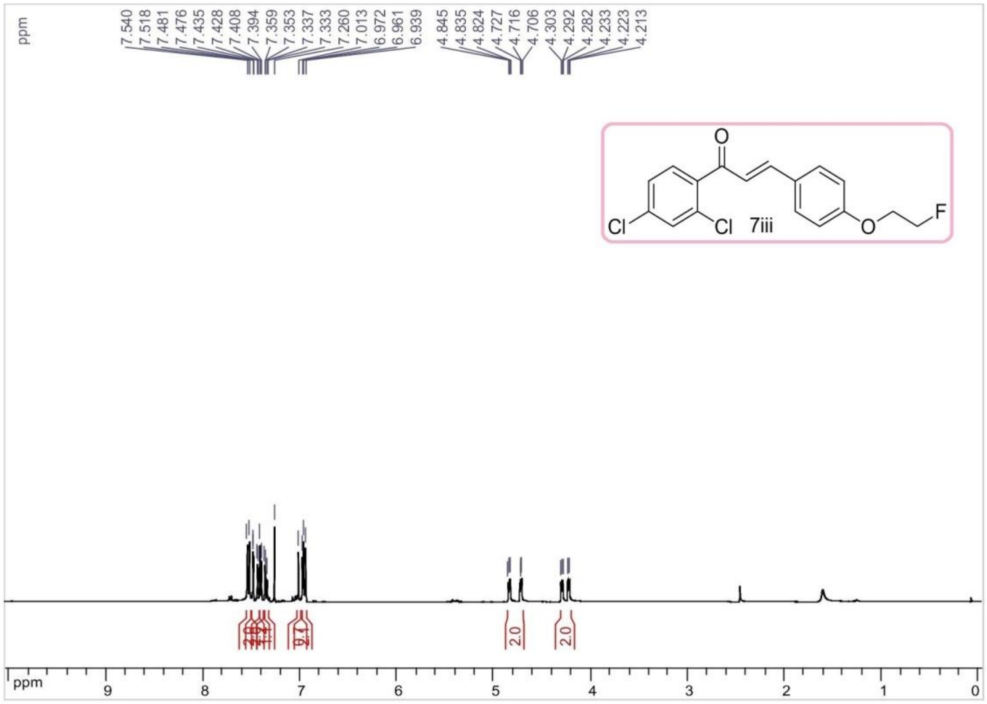
ppm
7.540
7.518
7.481
7.476
7.435
7.428
7.408
7.394
7.359
7.353
7.337
7.333
7.260
7.013
6.972
6.961
6.939
4.845
4.835
4.824
4.727
4.716
4.706
4.303
4.292
4.282
4.233
4.223
4.213
Cl
Cl
7iii
O
F
2.0
2.0
ppm
9
8
7
6
5
4
3
2
1
0

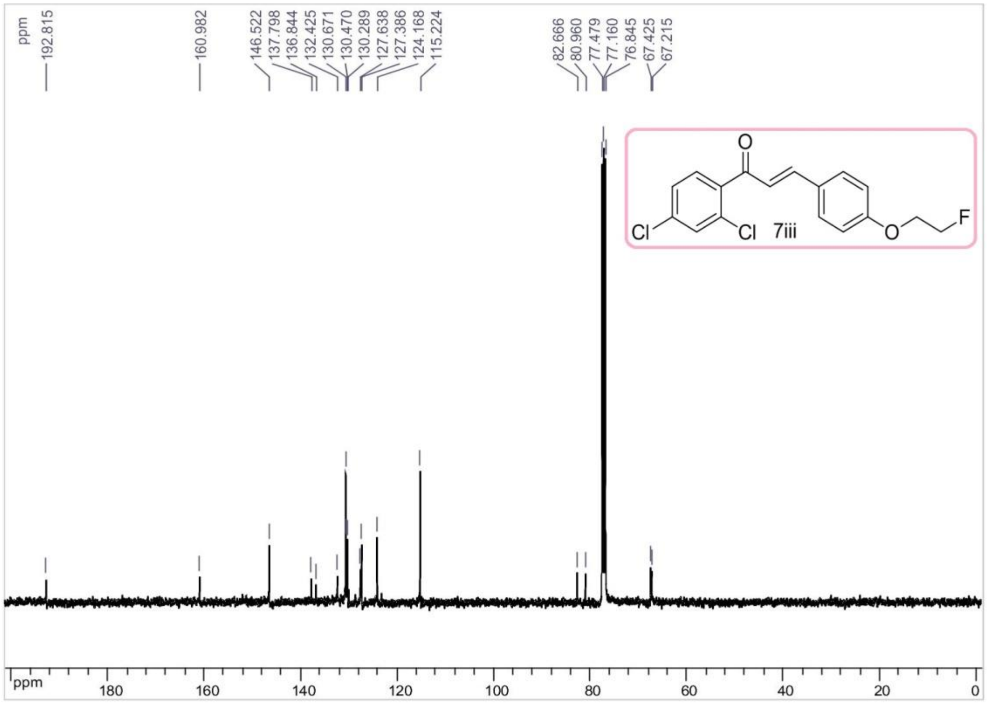
ppm
192.815
160.982
146.522
137.798
136.844
132.425
130.671
130.470
130.289
127.638
127.386
124.168
115.224
82.666
80.960
77.479
77.160
76.845
67.425
67.215
O
Cl
Cl
7iii
O
F
ppm
180
160
140
120
100
80
60
40
20
0

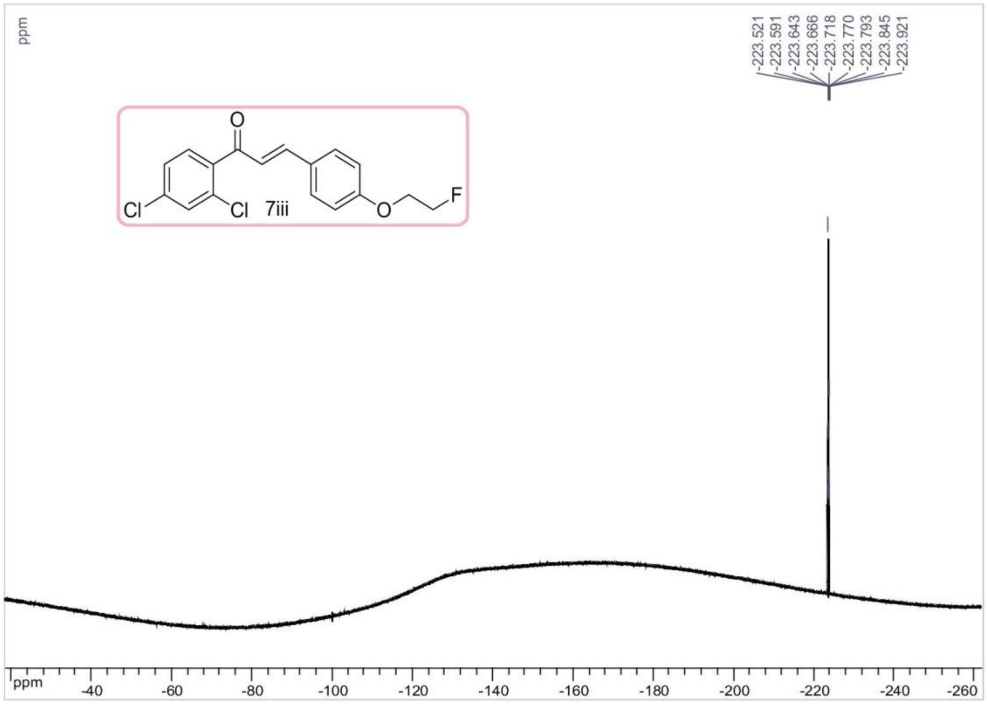
ppm
-223.521
-223.591
-223.643
-223.666
-223.718
-223.770
-223.793
-223.845
-223.921
O
Cl
Cl
7iii
O
F
ppm
-40
-60
-80
-100
-120
-140
-160
-180
-200
-220
-240
-260

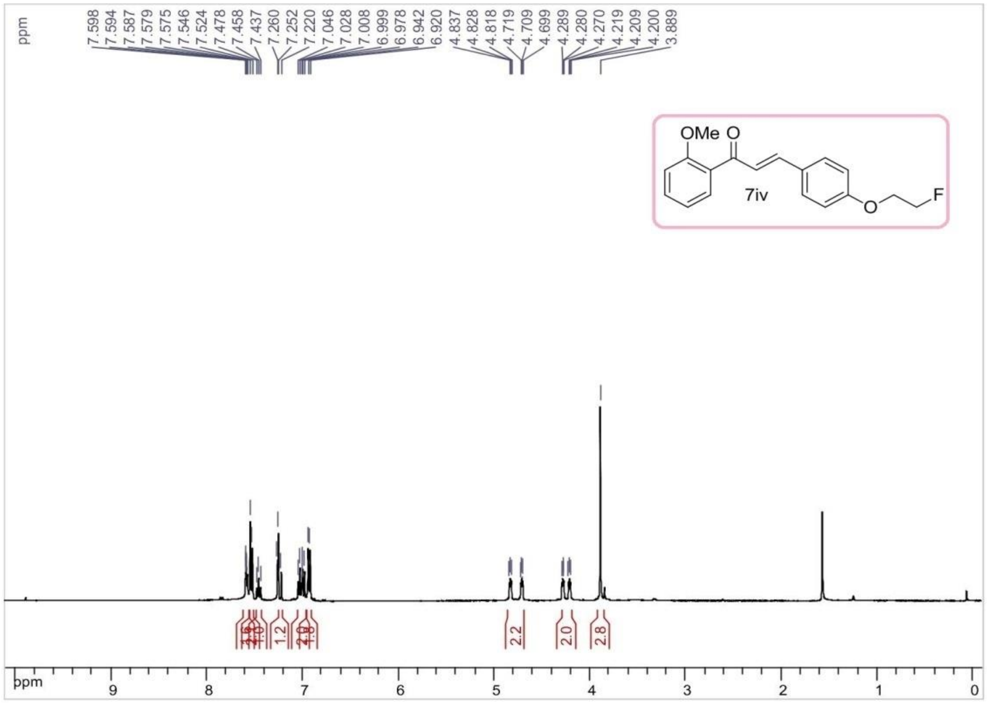
OMe
O
7iv
O
F
ppm
9
8
7
6
5
4
3
2
1
0

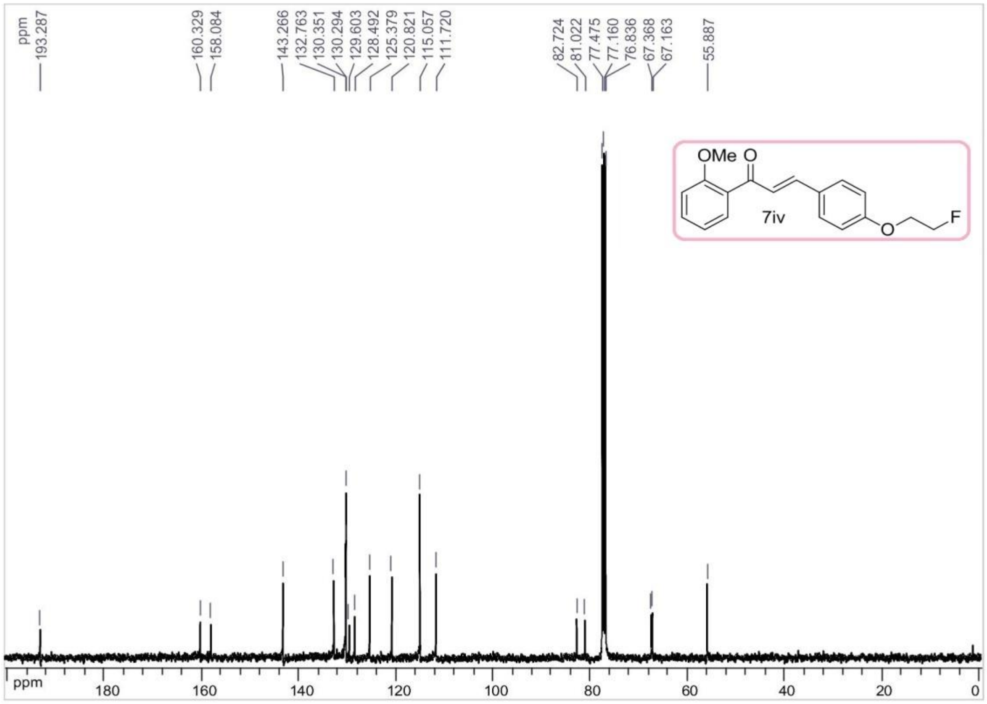
193.287
160.329
158.084
143.266
132.763
130.351
130.294
129.603
128.492
125.379
120.821
115.057
111.720
82.724
81.022
77.475
77.160
76.836
67.368
67.163
55.887
OMe
O
7iv
O
F
ppm
180
160
140
120
100
80
60
40
20
0

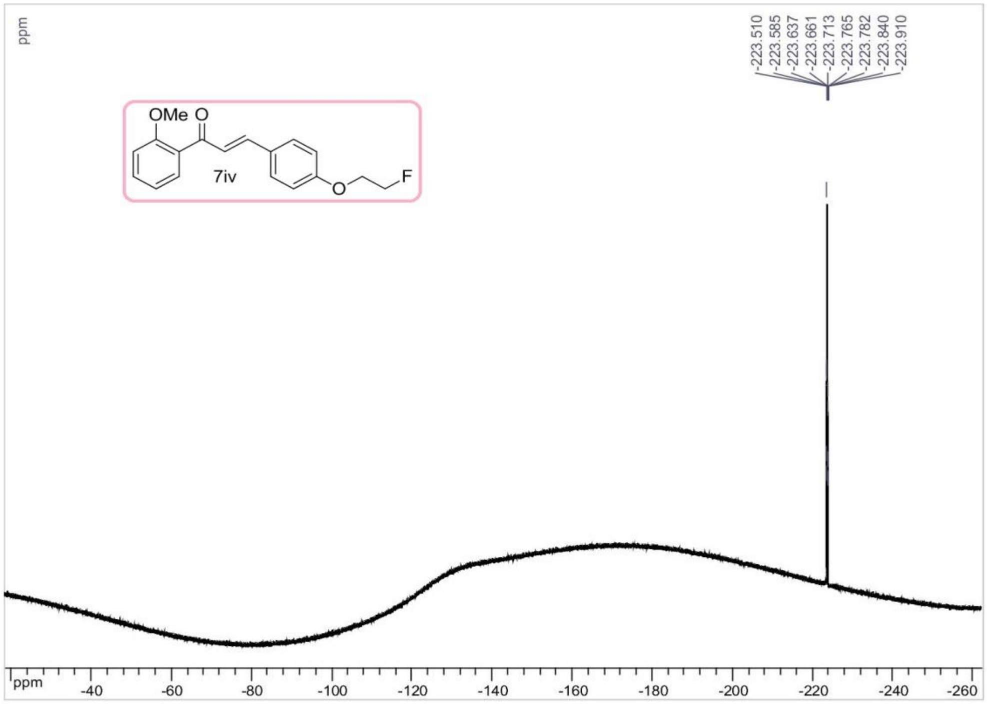
ppm
-223.510
-223.585
-223.637
-223.661
-223.713
-223.765
-223.782
-223.840
-223.910
OMe O
7iv
O
F
ppm -40 -60 -80 -100 -120 -140 -160 -180 -200 -220 -240 -260

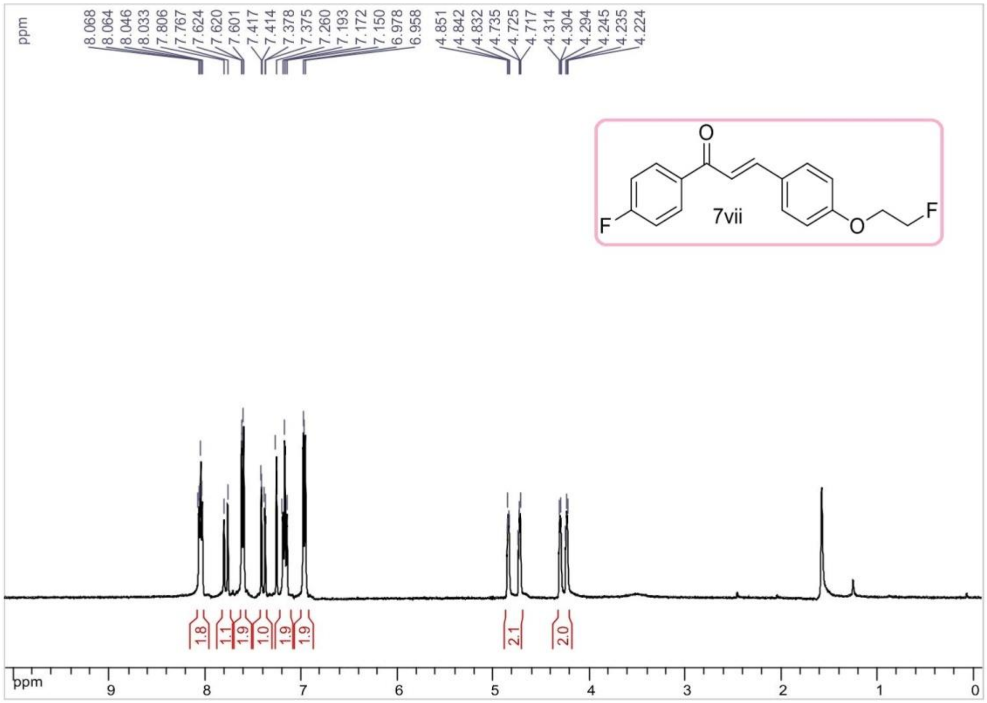
ppm
8.068
8.064
8.046
8.033
7.806
7.767
7.624
7.620
7.601
7.417
7.414
7.378
7.375
7.260
7.193
7.172
7.150
6.978
6.958
4.851
4.842
4.832
4.735
4.725
4.717
4.314
4.304
4.294
4.245
4.235
4.224
O
F
7vii
O
F
1.8 1.1 1.9 1.0 1.9 1.9 2.1 2.0
ppm 9 8 7 6 5 4 3 2 1 0

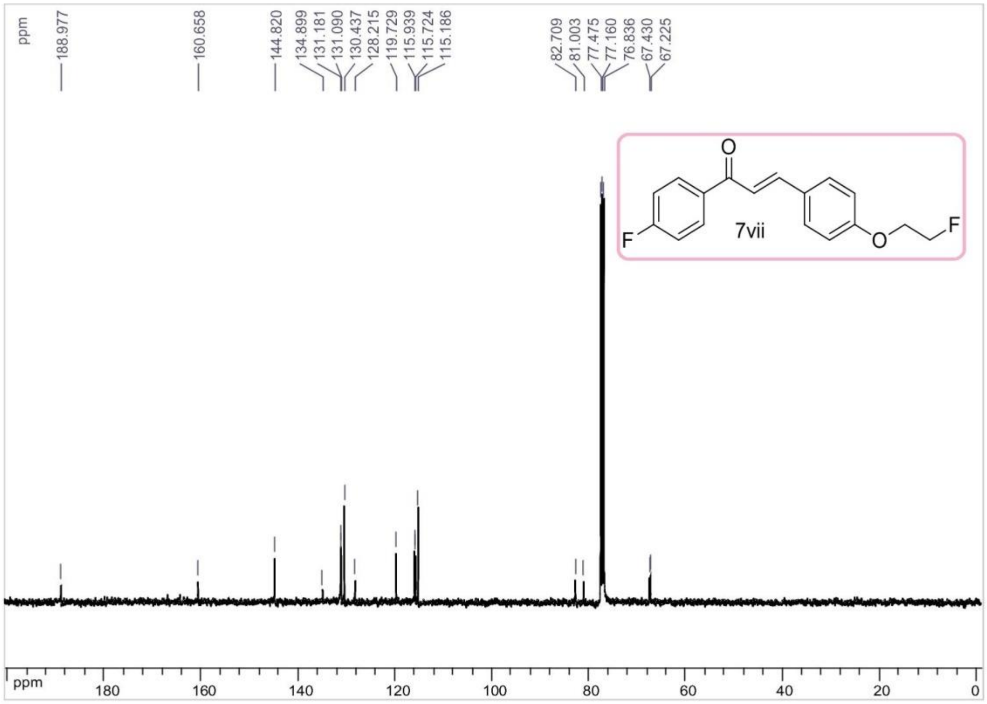
ppm
188.977
160.658
144.820
134.899
131.181
131.090
130.437
128.215
119.729
115.939
115.724
115.186
82.709
81.003
77.475
77.160
76.836
67.430
67.225
O
F
7vii
O
F
ppm
180
160
140
120
100
80
60
40
20
0

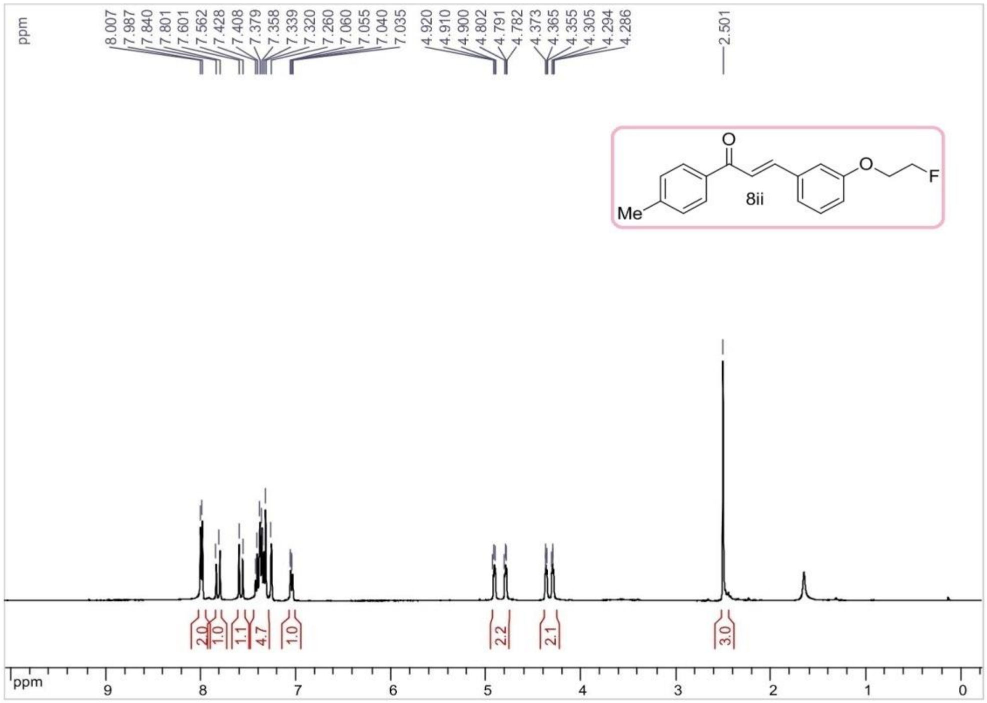
ppm
8.007
7.987
7.840
7.801
7.601
7.562
7.428
7.408
7.379
7.358
7.339
7.320
7.260
7.060
7.055
7.040
7.035
4.920
4.910
4.900
4.802
4.791
4.782
4.373
4.365
4.355
4.305
4.294
4.286
2.501
O
O
F
Me
8ii
2.0
1.0
1.1
4.7
1.0
2.2
2.1
3.0
ppm
9
8
7
6
5
4
3
2
1
0

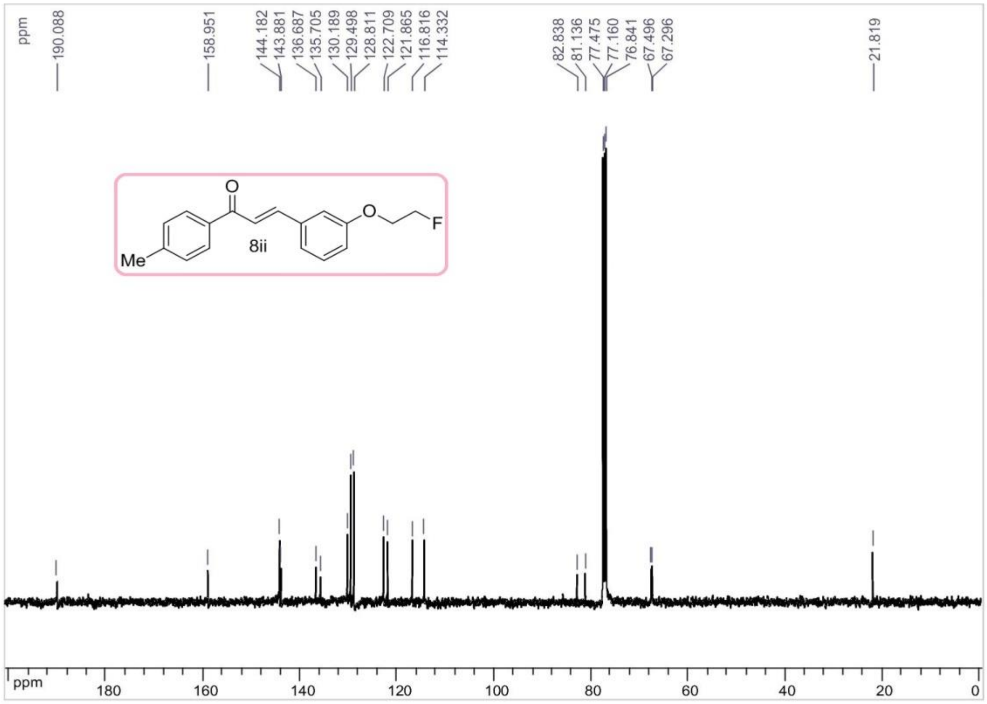
ppm
190.088
158.951
144.182
143.881
136.687
135.705
130.189
129.498
128.811
122.709
121.865
116.816
114.332
82.838
81.136
77.475
77.160
76.841
67.496
67.296
21.819
8ii
Me
O
F
ppm
180
160
140
120
100
80
60
40
20
0

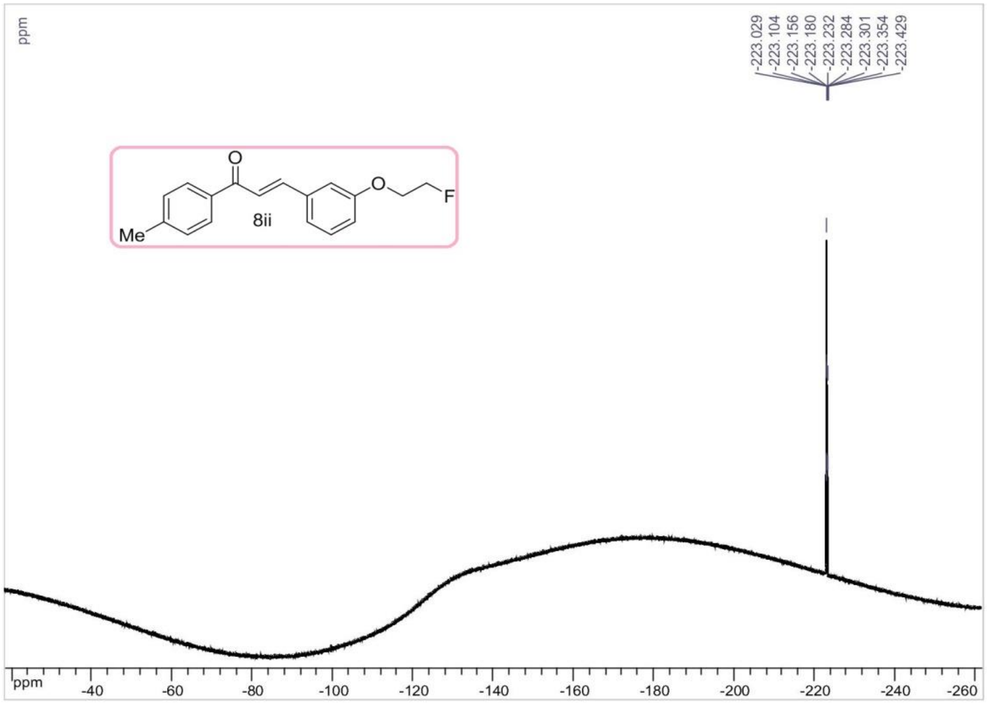
ppm
-223.029
-223.104
-223.156
-223.180
-223.232
-223.284
-223.301
-223.354
-223.429
8ii
Me
O
F
ppm
-40
-60
-80
-100
-120
-140
-160
-180
-200
-220
-240
-260

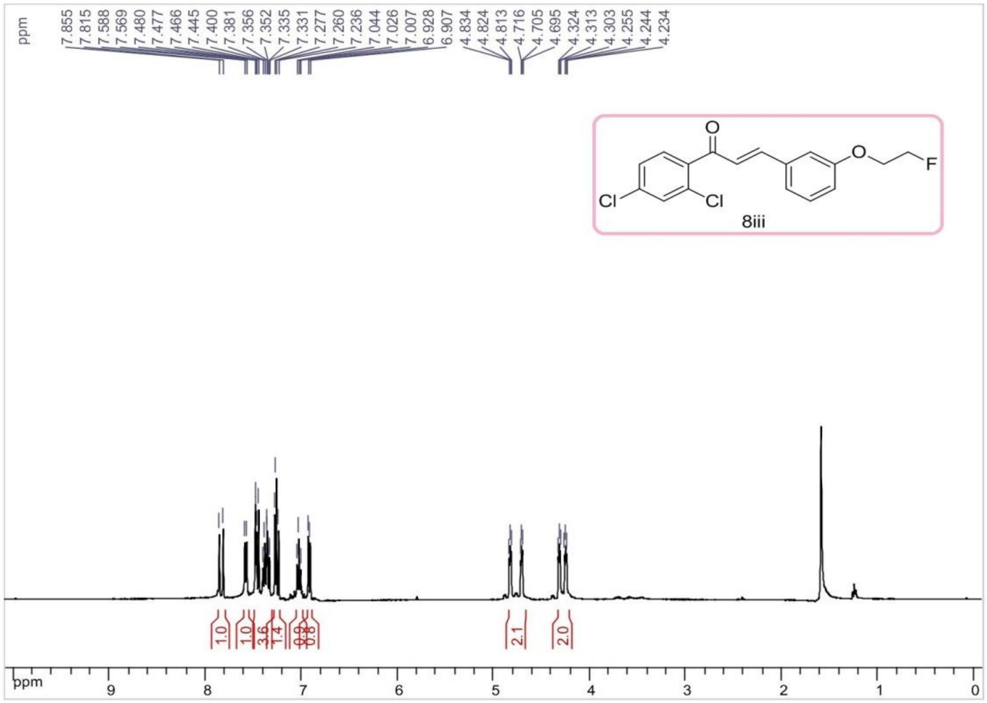
ppm
7.855
7.815
7.588
7.569
7.480
7.477
7.466
7.445
7.400
7.381
7.356
7.352
7.335
7.331
7.277
7.260
7.236
7.044
7.026
7.007
6.928
6.907
4.834
4.824
4.813
4.716
4.705
4.695
4.324
4.313
4.303
4.255
4.244
4.234
O
O
F
Cl
Cl
8iii
1.0
1.0
3.6
1.4
2.1
2.0
ppm
9
8
7
6
5
4
3
2
1
0

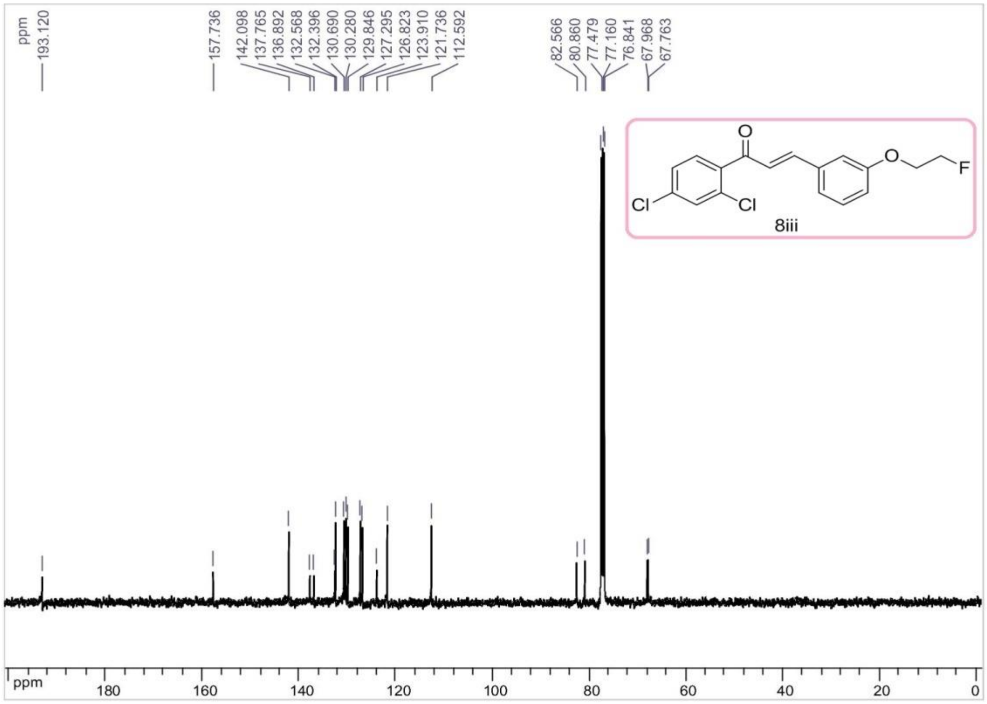
ppm
193.120
157.736
142.098
137.765
136.892
132.568
132.396
130.690
130.280
129.846
127.295
126.823
123.910
121.736
112.592
82.566
80.860
77.479
77.160
76.841
67.968
67.763
O
O
F
Cl
Cl
8iii
ppm
180
160
140
120
100
80
60
40
20
0

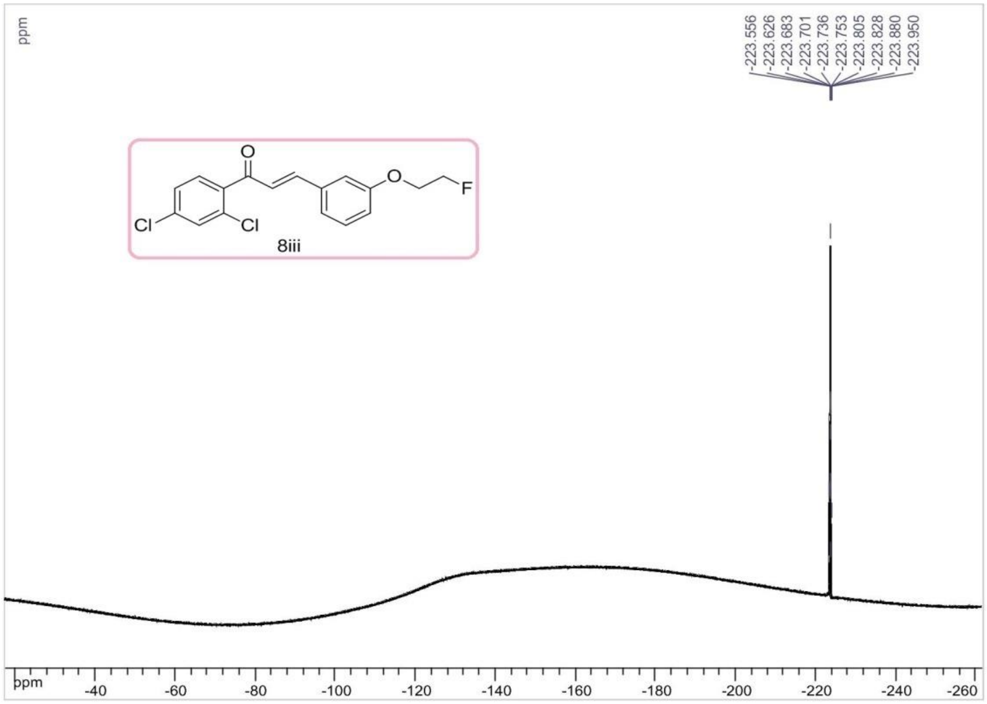

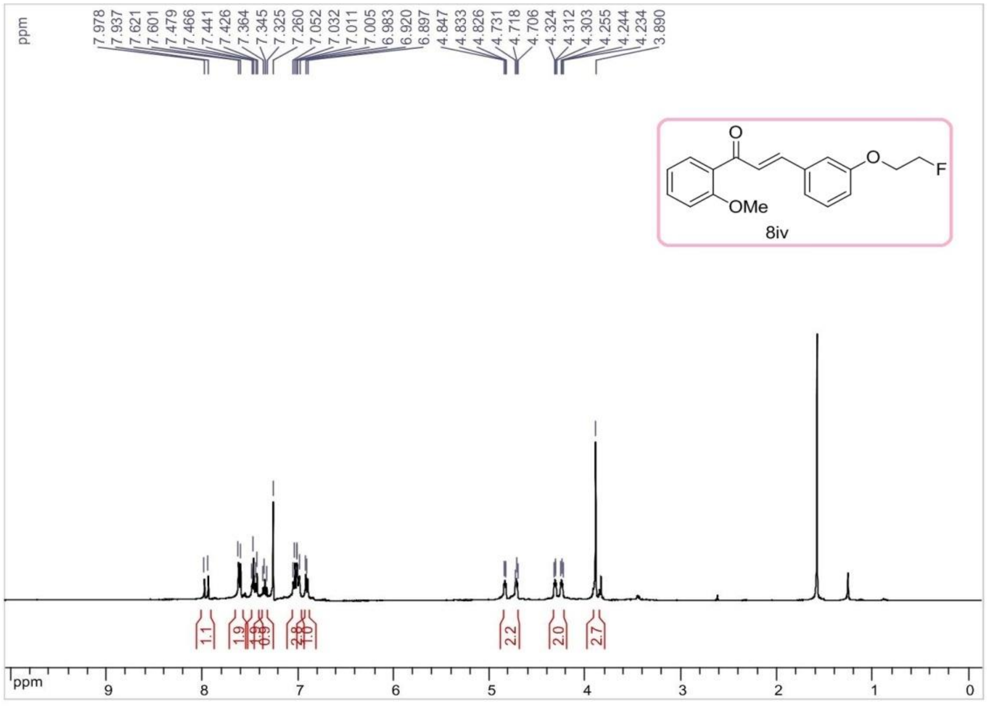

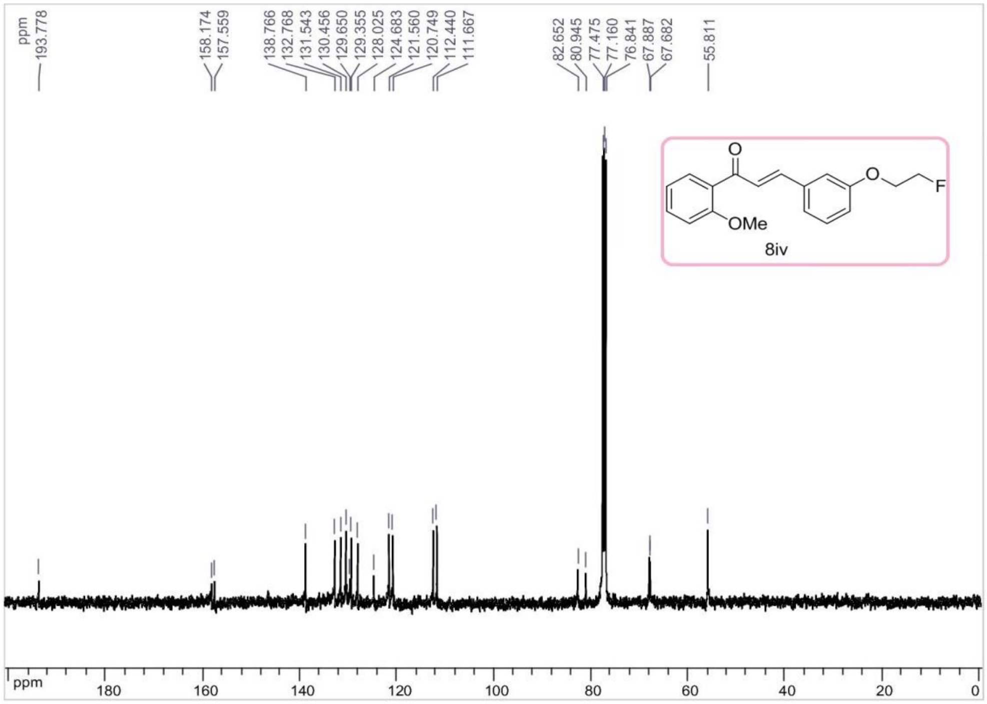
ppm
193.778
158.174
157.559
138.766
132.768
131.543
130.456
129.650
129.355
128.025
124.683
121.560
120.749
112.440
111.667
82.652
80.945
77.475
77.160
76.841
67.887
67.682
55.811
O
O
F
OMe
8iv
ppm
180
160
140
120
100
80
60
40
20
0

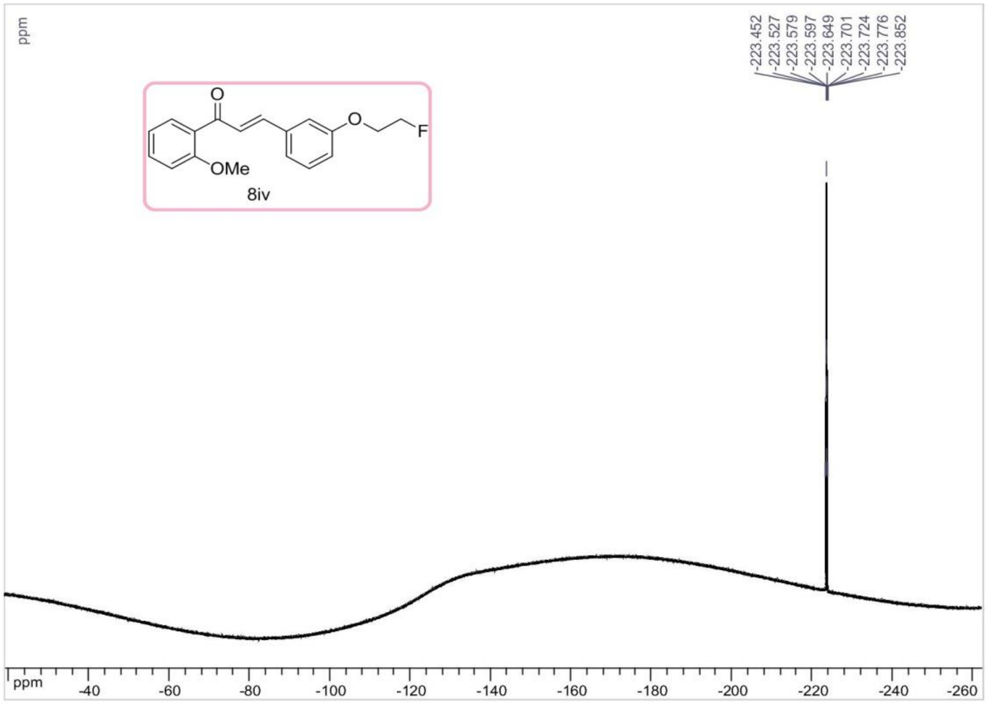
ppm
-223.452
-223.527
-223.579
-223.597
-223.649
-223.701
-223.724
-223.776
-223.852
O
O
F
OMe
8iv
ppm
-40
-60
-80
-100
-120
-140
-160
-180
-200
-220
-240
-260

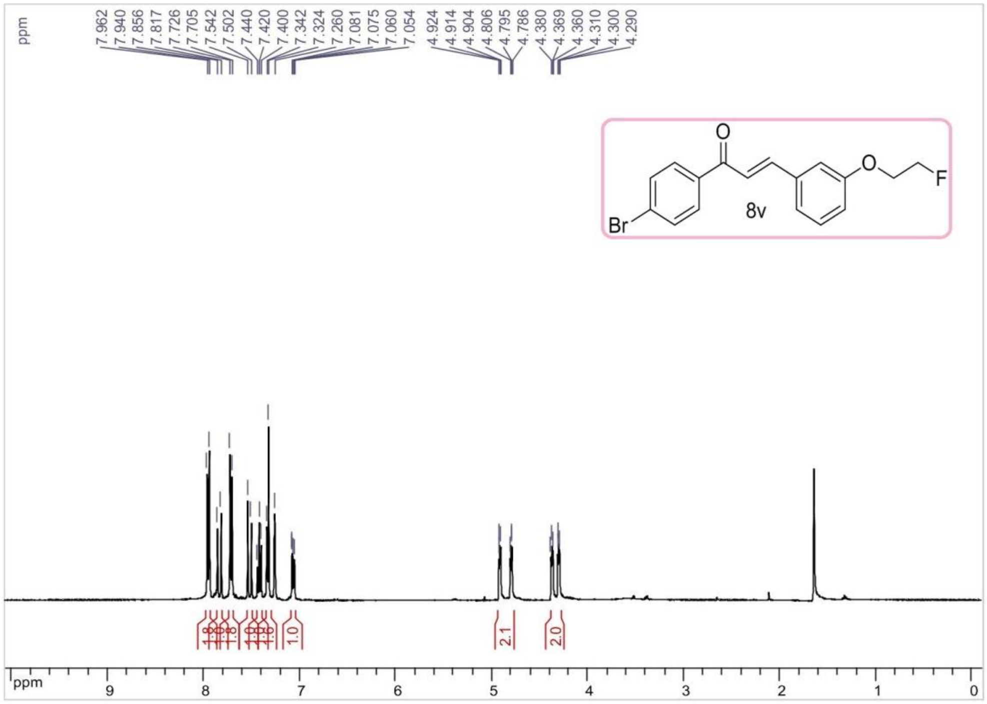

ppm
7.962
7.940
7.856
7.817
7.726
7.705
7.542
7.502
7.440
7.420
7.400
7.342
7.324
7.260
7.081
7.075
7.060
7.054
4.924
4.914
4.904
4.806
4.795
4.786
4.380
4.369
4.360
4.310
4.300
4.290
O
F
Br
8v
1.0
2.1
2.0
ppm
9
8
7
6
5
4
3
2
1
0

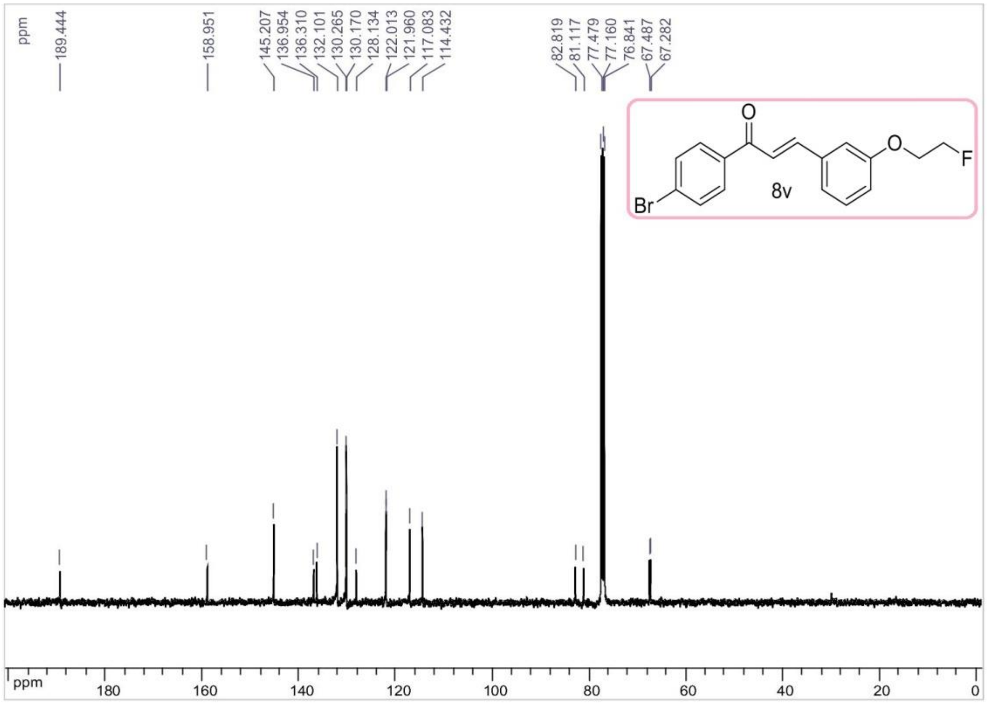

ppm
189.444
158.951
145.207
136.954
136.310
132.101
130.265
130.170
128.134
122.013
121.960
117.083
114.432
82.819
81.117
77.479
77.160
76.841
67.487
67.282
O
F
Br
8v
ppm
180
160
140
120
100
80
60
40
20
0

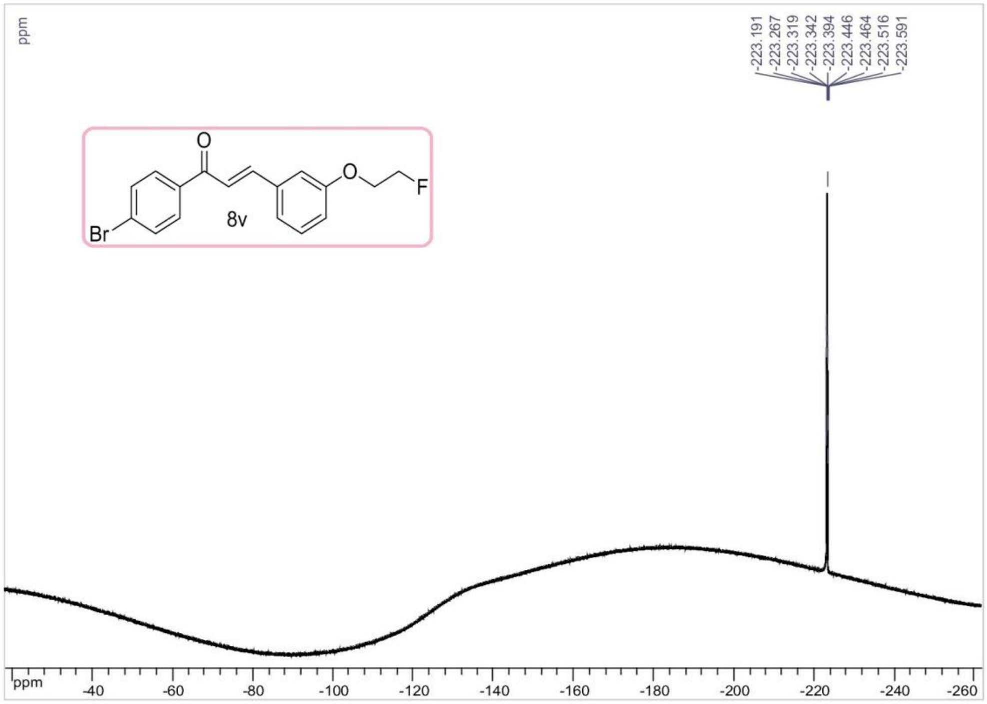
ppm
-223.191
-223.267
-223.319
-223.342
-223.394
-223.446
-223.464
-223.516
-223.591
8v
Br
O
F
ppm
-40
-60
-80
-100
-120
-140
-160
-180
-200
-220
-240
-260

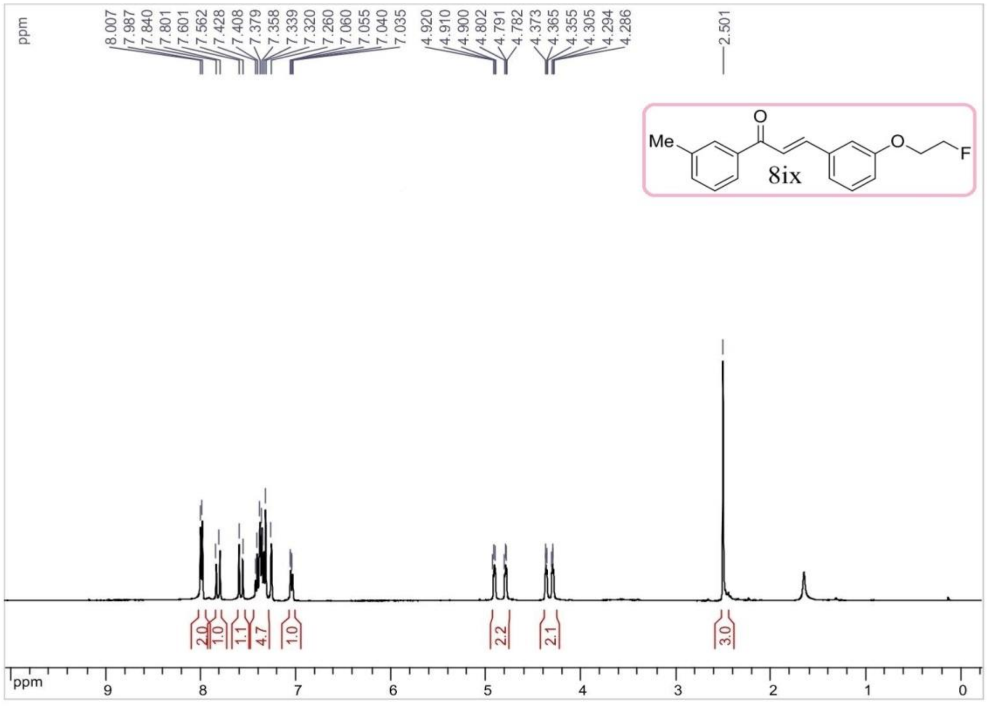
ppm
8.007
7.987
7.840
7.801
7.601
7.562
7.428
7.408
7.379
7.358
7.339
7.320
7.260
7.060
7.055
7.040
7.035
4.920
4.910
4.900
4.802
4.791
4.782
4.373
4.365
4.355
4.305
4.294
4.286
2.501
Me
O
F
8ix
2.0
1.0
1.1
4.7
1.0
2.2
2.1
3.0
ppm
9
8
7
6
5
4
3
2
1
0

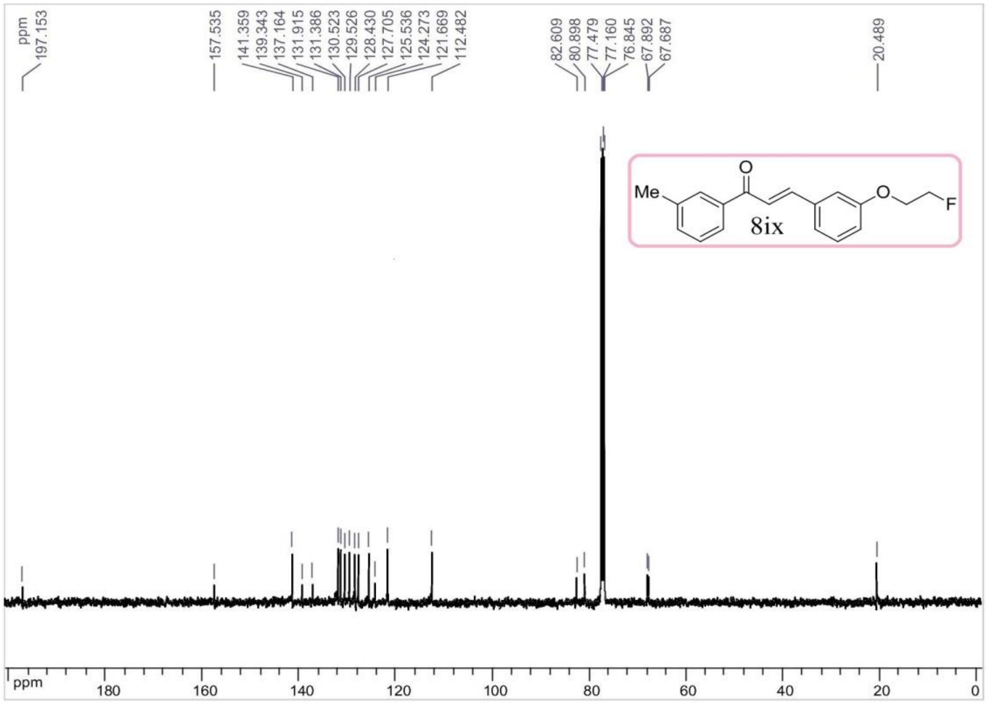
ppm
197.153
157.535
141.359
139.343
137.164
131.915
131.386
130.523
129.526
128.430
127.705
125.536
124.273
121.669
112.482
82.609
80.898
77.479
77.160
76.845
67.892
67.687
20.489
Me
O
F
8ix
ppm
180
160
140
120
100
80
60
40
20
0

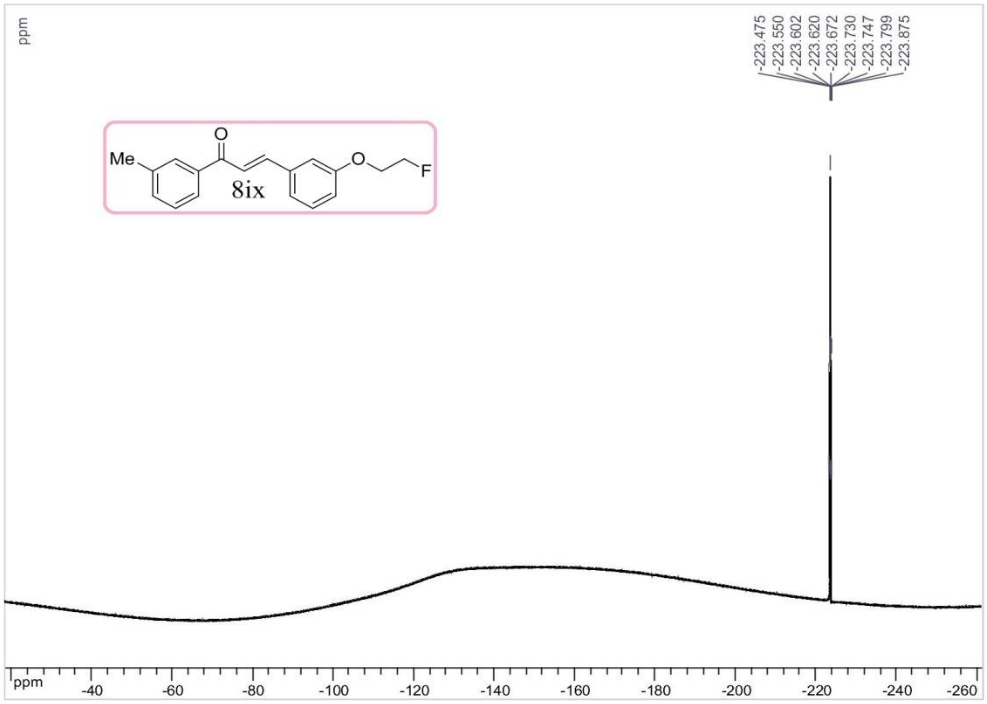
ppm
-223.475
-223.550
-223.602
-223.620
-223.672
-223.730
-223.747
-223.799
-223.875
Me
O
F
8ix
ppm
-40
-60
-80
-100
-120
-140
-160
-180
-200
-220
-240
-260

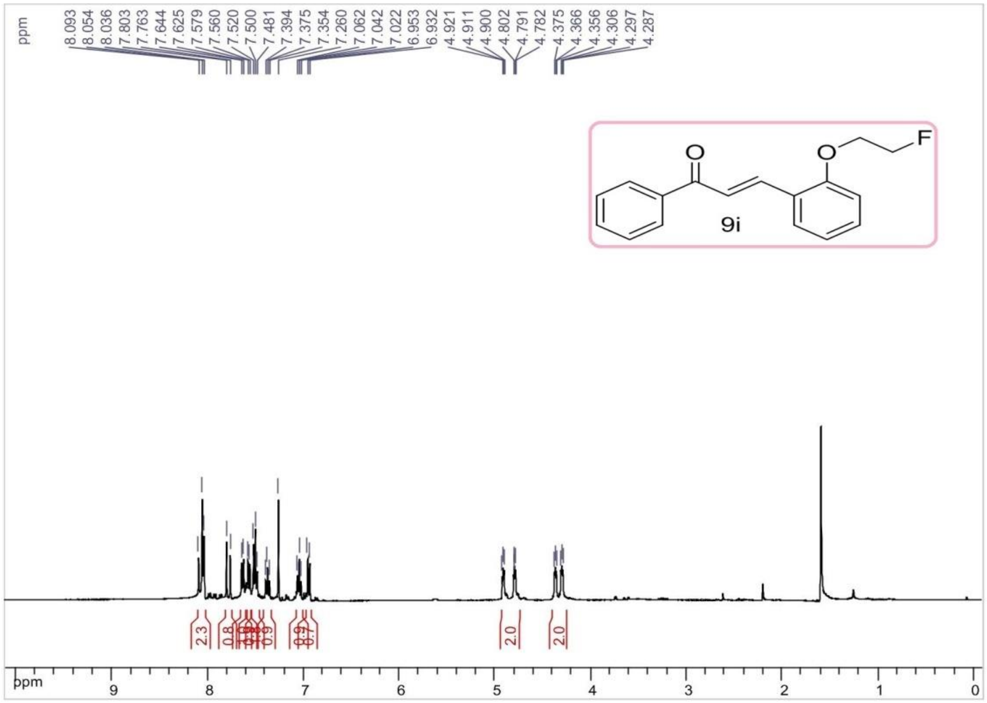
ppm
8.093
8.054
8.036
7.803
7.763
7.644
7.625
7.579
7.560
7.520
7.500
7.481
7.394
7.375
7.354
7.260
7.062
7.042
7.022
6.953
6.932
4.921
4.911
4.900
4.802
4.791
4.782
4.375
4.366
4.356
4.306
4.297
4.287
O
F
9i
2.3
0.8
0.9
0.9
0.7
2.0
2.0
ppm
9
8
7
6
5
4
3
2
1
0

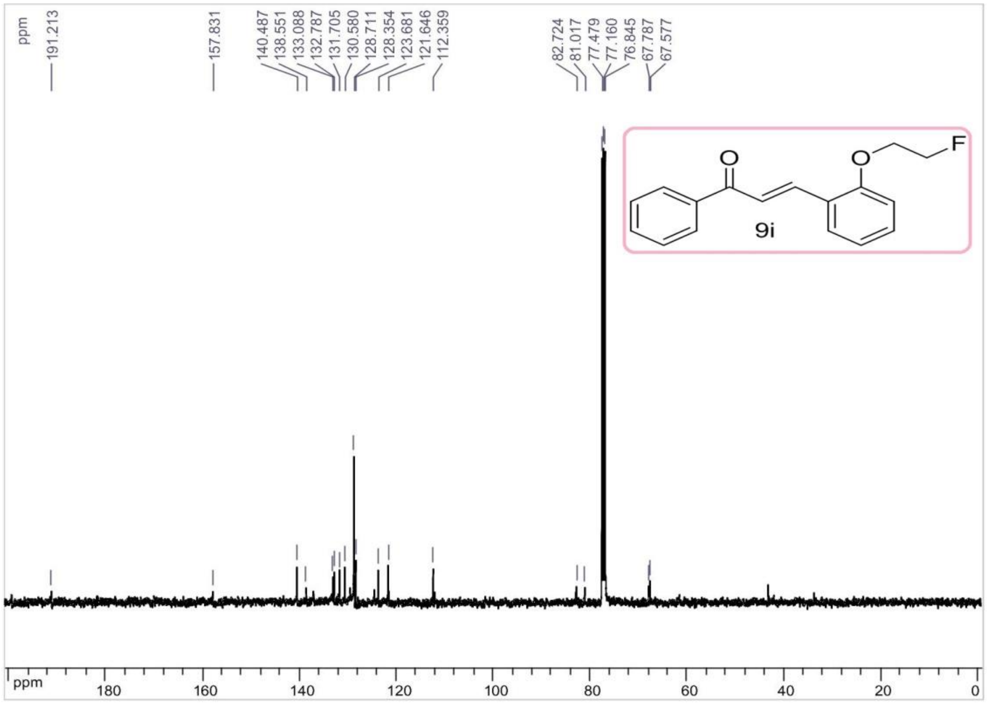
ppm
191.213
157.831
140.487
138.551
133.088
132.787
131.705
130.580
128.711
128.354
123.681
121.646
112.359
82.724
81.017
77.479
77.160
76.845
67.787
67.577
O
F
9i
ppm
180
160
140
120
100
80
60
40
20
0

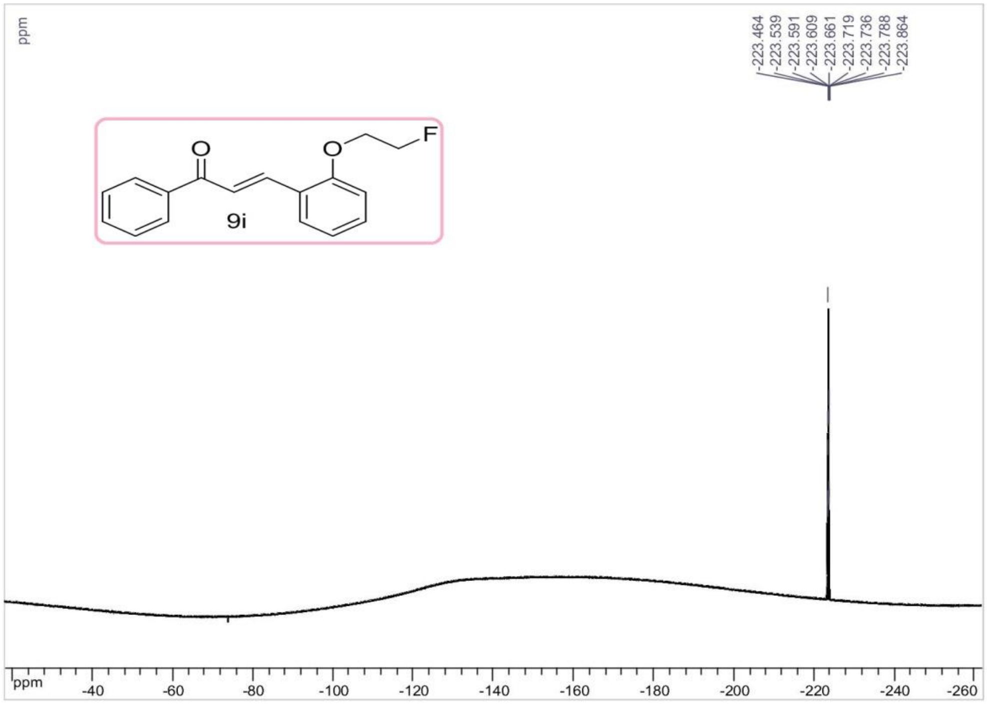
ppm
-223.464
-223.539
-223.591
-223.609
-223.661
-223.719
-223.736
-223.788
-223.864
O
F
9i
ppm -40 -60 -80 -100 -120 -140 -160 -180 -200 -220 -240 -260

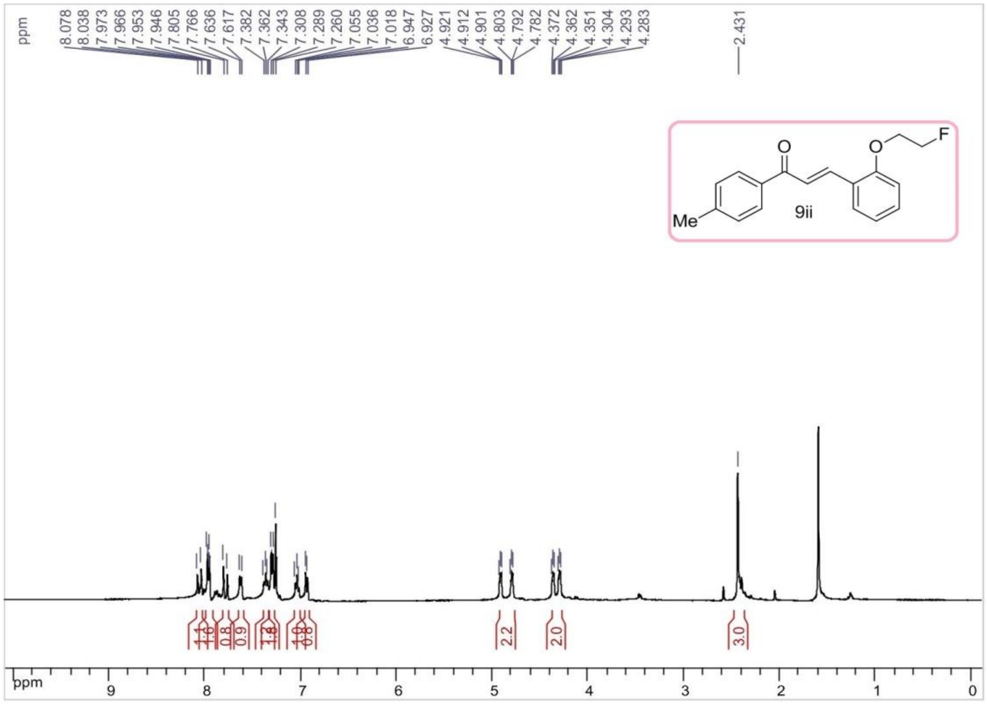
ppm
8.078
8.038
7.973
7.966
7.953
7.946
7.805
7.766
7.636
7.617
7.382
7.362
7.343
7.308
7.289
7.260
7.055
7.036
7.018
6.947
6.927
4.921
4.912
4.901
4.803
4.792
4.782
4.372
4.362
4.351
4.304
4.293
4.283
2.431
O
F
Me
9ii
2.2
2.0
3.0
ppm 9 8 7 6 5 4 3 2 1 0

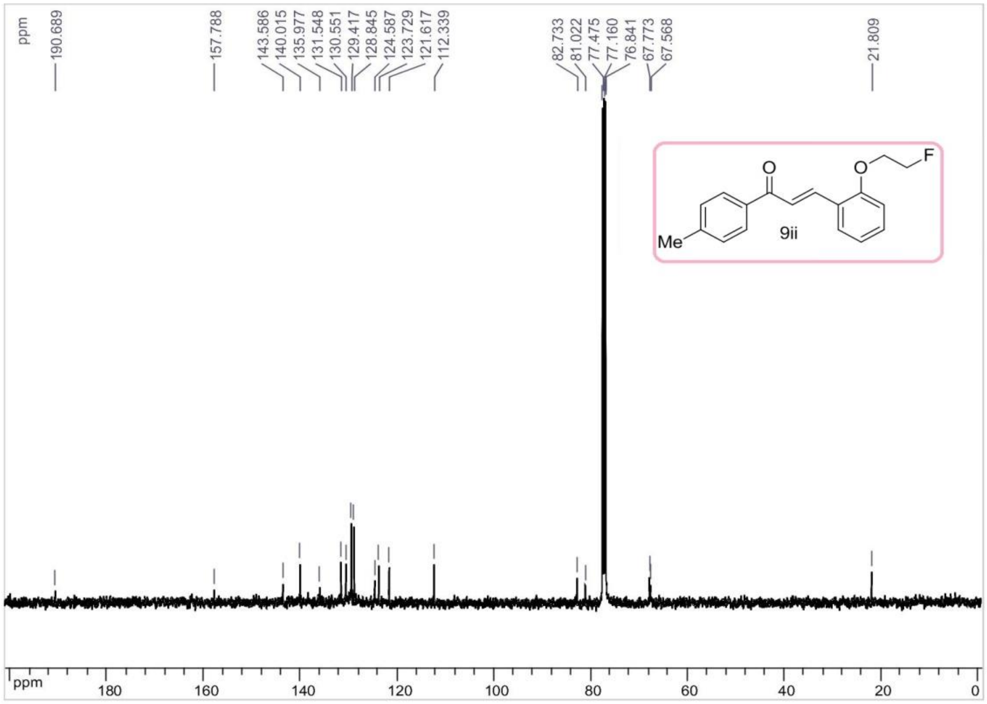
ppm
190.689
157.788
143.586
140.015
135.977
131.548
130.551
129.417
128.845
124.587
123.729
121.617
112.339
82.733
81.022
77.475
77.160
76.841
67.773
67.568
21.809
Me
9ii
ppm
180
160
140
120
100
80
60
40
20
0

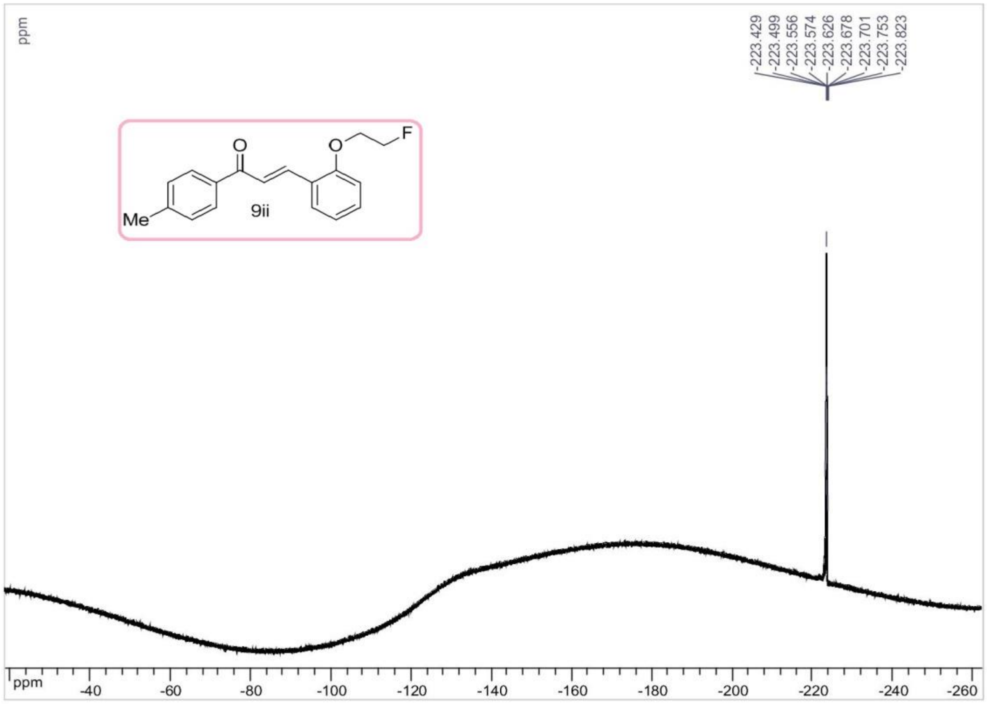
ppm
-223.429
-223.499
-223.556
-223.574
-223.626
-223.678
-223.701
-223.753
-223.823
Me
9ii
ppm
-40
-60
-80
-100
-120
-140
-160
-180
-200
-220
-240
-260

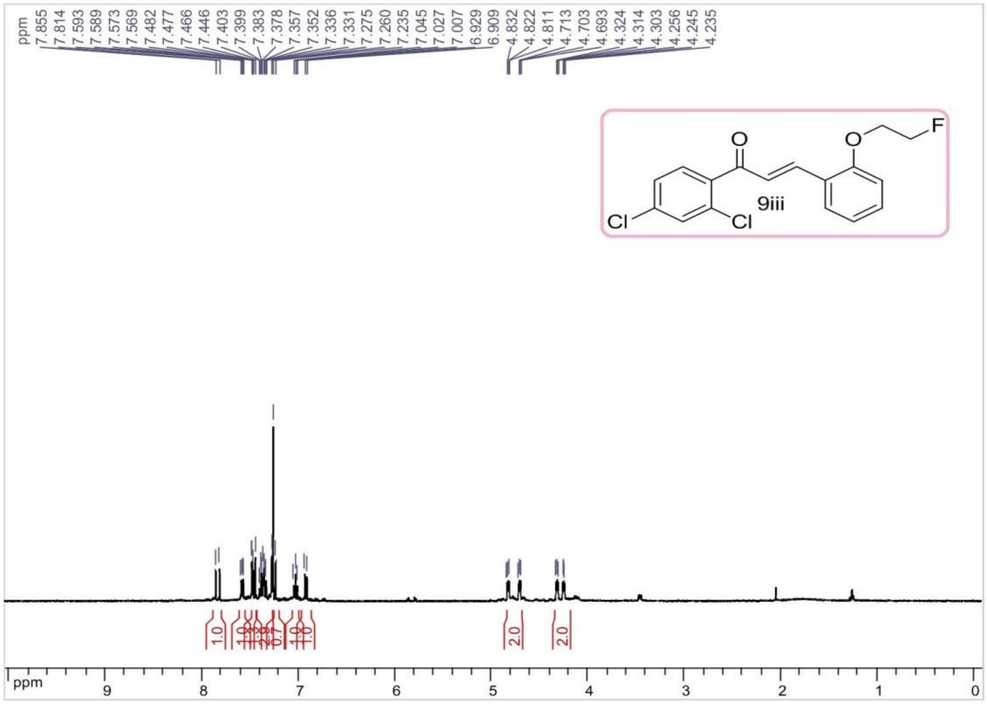
ppm
7.855
7.814
7.593
7.589
7.573
7.569
7.482
7.477
7.466
7.446
7.403
7.399
7.383
7.378
7.357
7.352
7.336
7.331
7.275
7.260
7.235
7.045
7.027
7.007
6.929
6.909
4.832
4.822
4.811
4.713
4.703
4.693
4.324
4.314
4.303
4.256
4.245
4.235
O
O
F
Cl
Cl
9iii
1.0
2.0
2.0
ppm
9
8
7
6
5
4
3
2
1
0

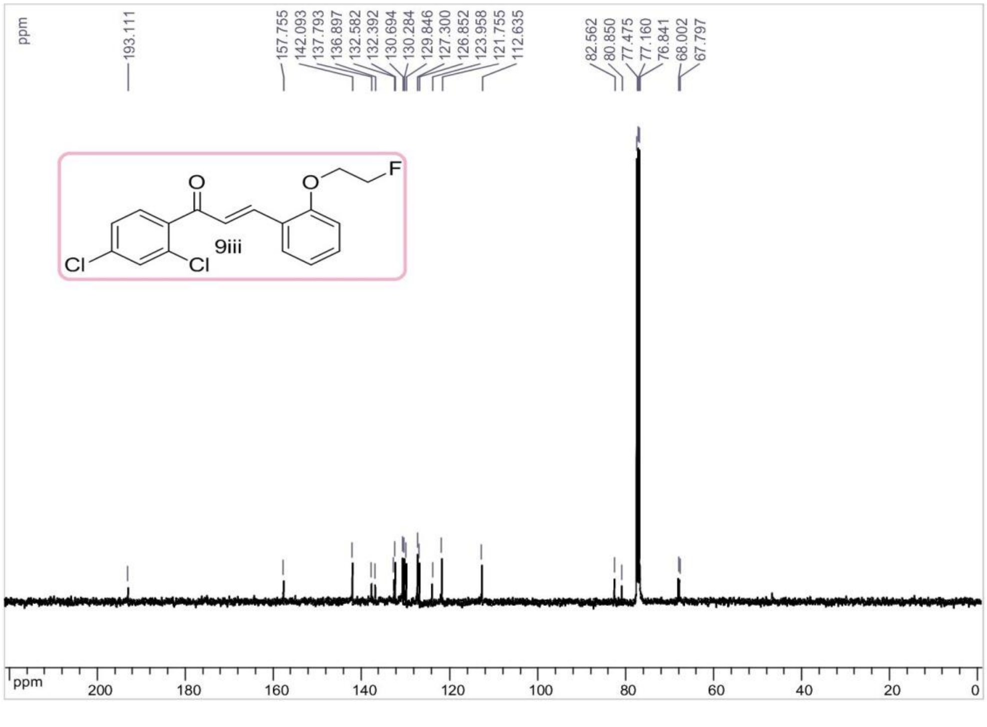
ppm
193.111
157.755
142.093
137.793
136.897
132.582
132.392
130.694
130.284
129.846
127.300
126.852
123.958
121.755
112.635
82.562
80.850
77.475
77.160
76.841
68.002
67.797
O
O
F
Cl
Cl
9iii
ppm
200
180
160
140
120
100
80
60
40
20
0

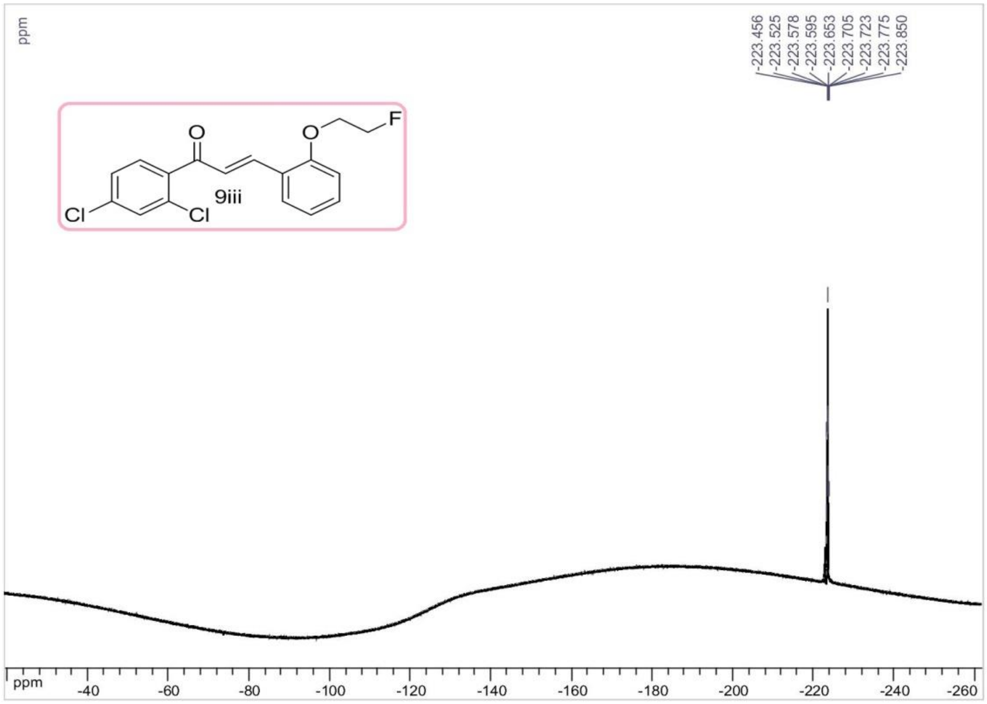
ppm
-223.456
-223.525
-223.578
-223.595
-223.653
-223.705
-223.723
-223.775
-223.850
O
F
Cl
Cl
9iii
ppm -40 -60 -80 -100 -120 -140 -160 -180 -200 -220 -240 -260

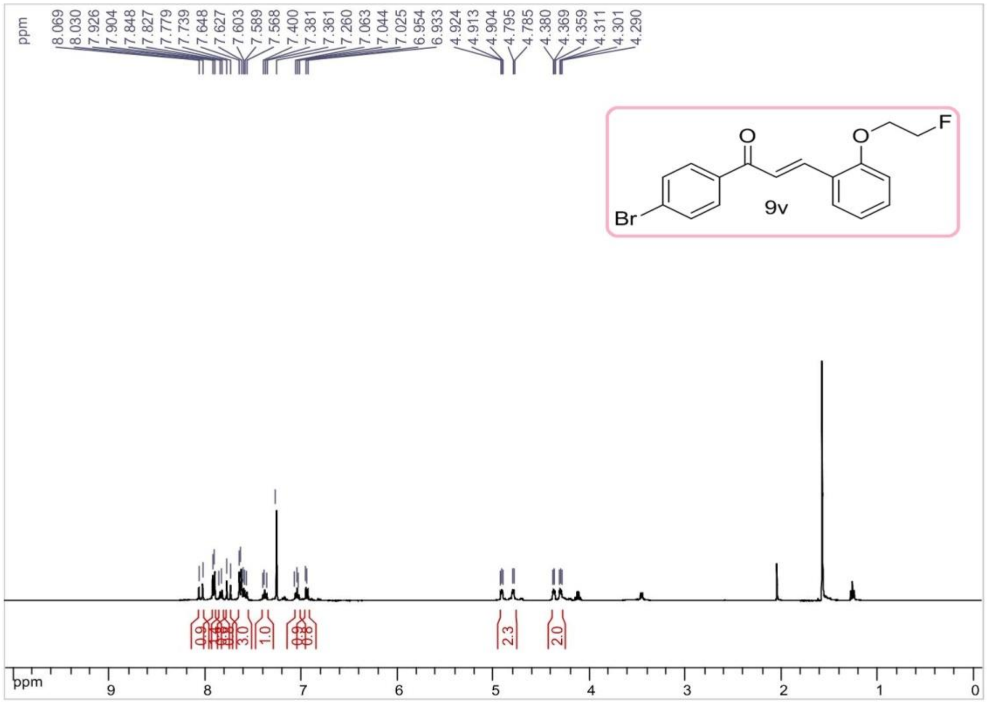
ppm
8.069
8.030
7.926
7.904
7.848
7.827
7.779
7.739
7.648
7.627
7.603
7.589
7.568
7.400
7.381
7.361
7.260
7.063
7.044
7.025
6.954
6.933
4.924
4.913
4.904
4.795
4.785
4.380
4.369
4.359
4.311
4.301
4.290
O
F
Br
9v
1.0
3.0
2.3
2.0
ppm 9 8 7 6 5 4 3 2 1 0

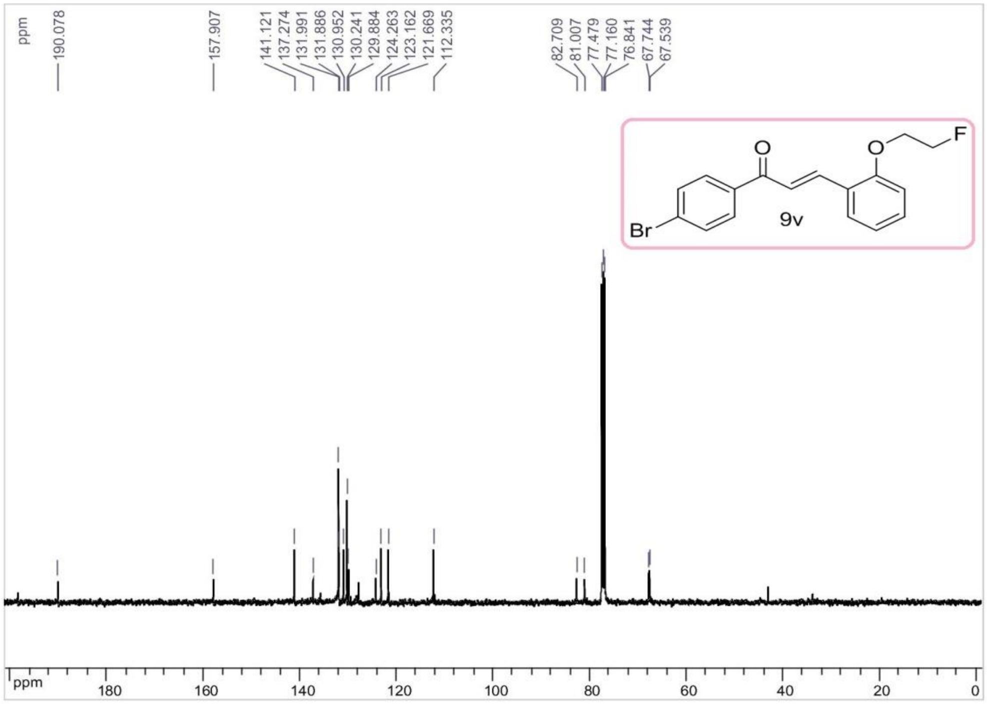

ppm
190.078
157.907
141.121
137.274
131.991
131.886
130.952
130.241
129.884
124.263
123.162
121.669
112.335
82.709
81.007
77.479
77.160
76.841
67.744
67.539
O
O
F
Br
9v
ppm
180
160
140
120
100
80
60
40
20
0

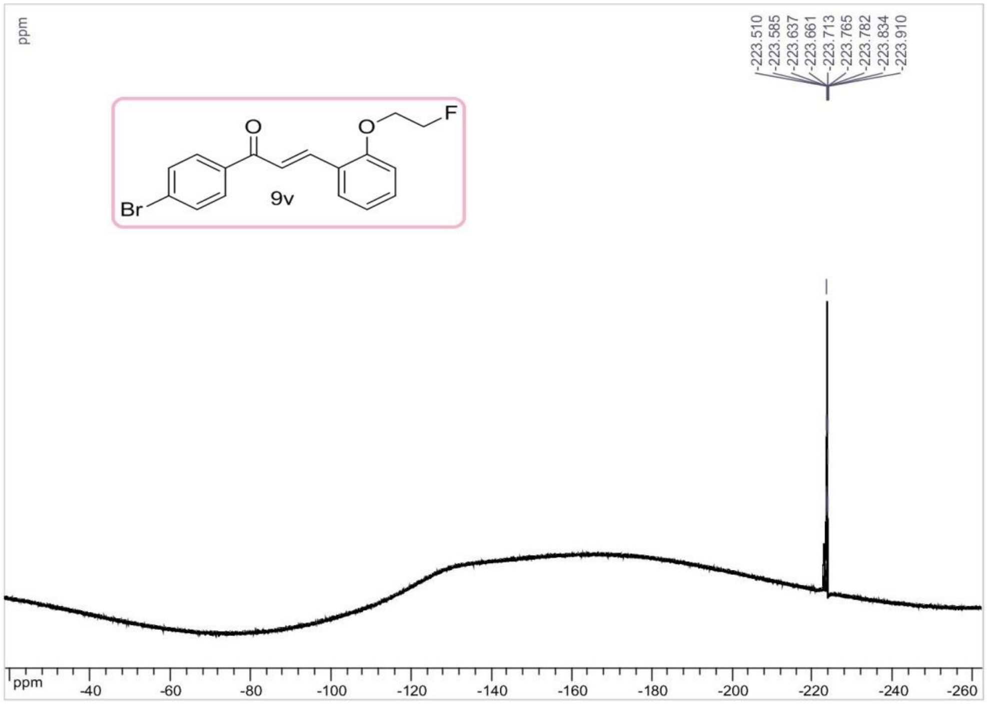

ppm
-223.510
-223.585
-223.637
-223.661
-223.713
-223.765
-223.782
-223.834
-223.910
O
O
F
Br
9v
ppm
-40
-60
-80
-100
-120
-140
-160
-180
-200
-220
-240
-260

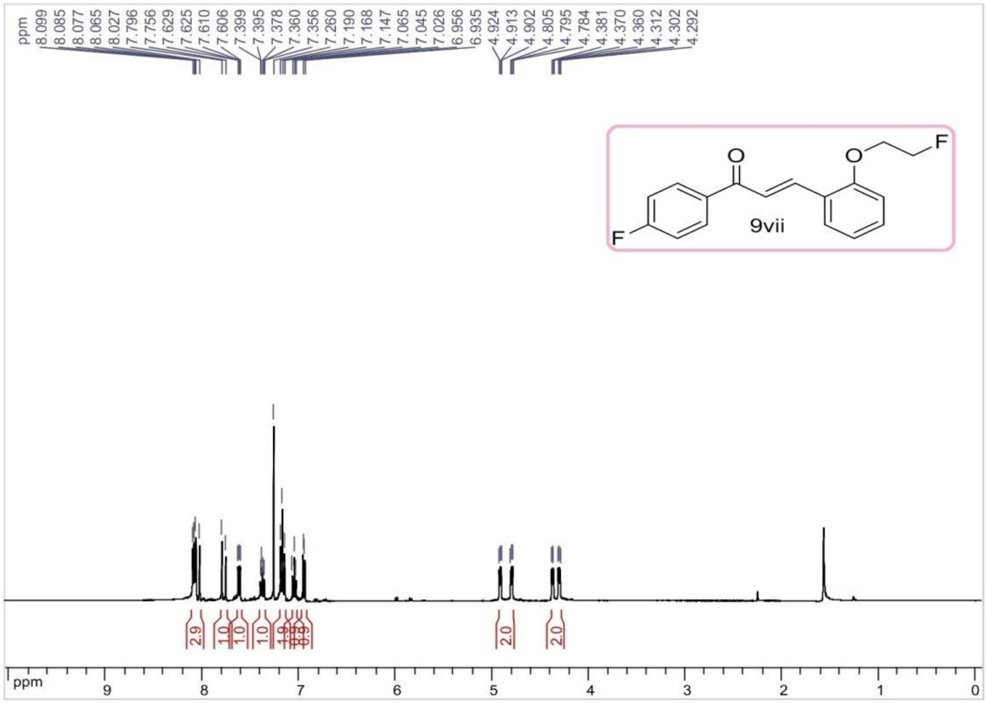
ppm
8.099
8.085
8.077
8.065
8.027
7.796
7.756
7.629
7.625
7.610
7.606
7.399
7.395
7.378
7.360
7.356
7.260
7.190
7.168
7.147
7.065
7.045
7.026
6.956
6.935
4.924
4.913
4.902
4.805
4.795
4.784
4.381
4.370
4.360
4.312
4.302
4.292
9vii
2.9
1.0
1.0
1.0
2.0
2.0
ppm
9
8
7
6
5
4
3
2
1
0

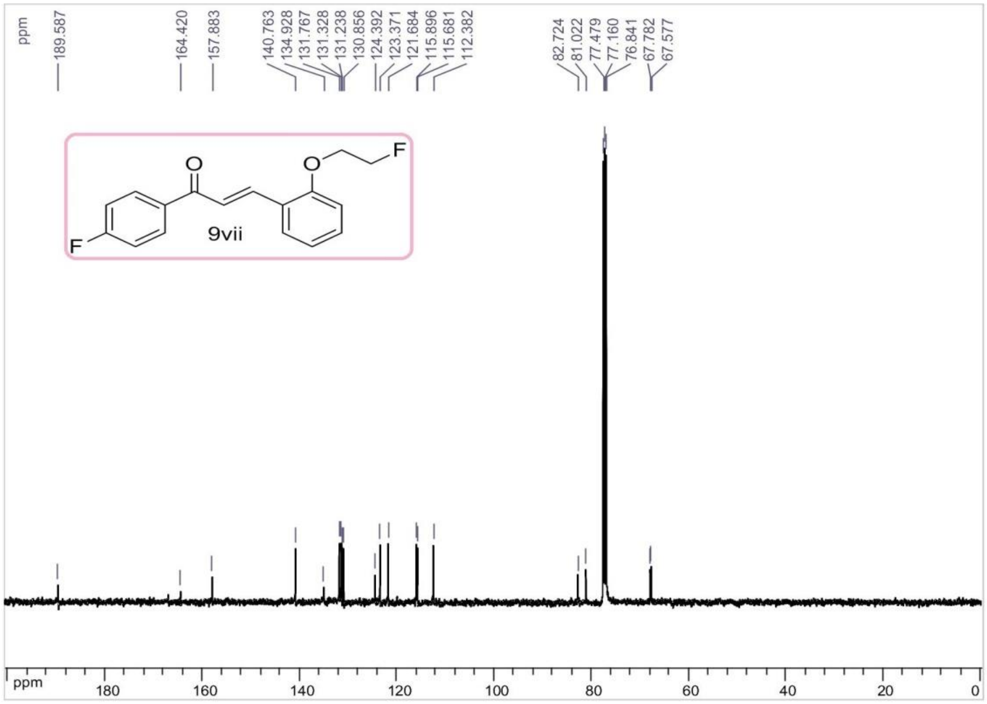
ppm
189.587
164.420
157.883
140.763
134.928
131.767
131.328
131.238
130.856
124.392
123.371
121.684
115.896
115.681
112.382
82.724
81.022
77.479
77.160
76.841
67.782
67.577
9vii
ppm
180
160
140
120
100
80
60
40
20
0

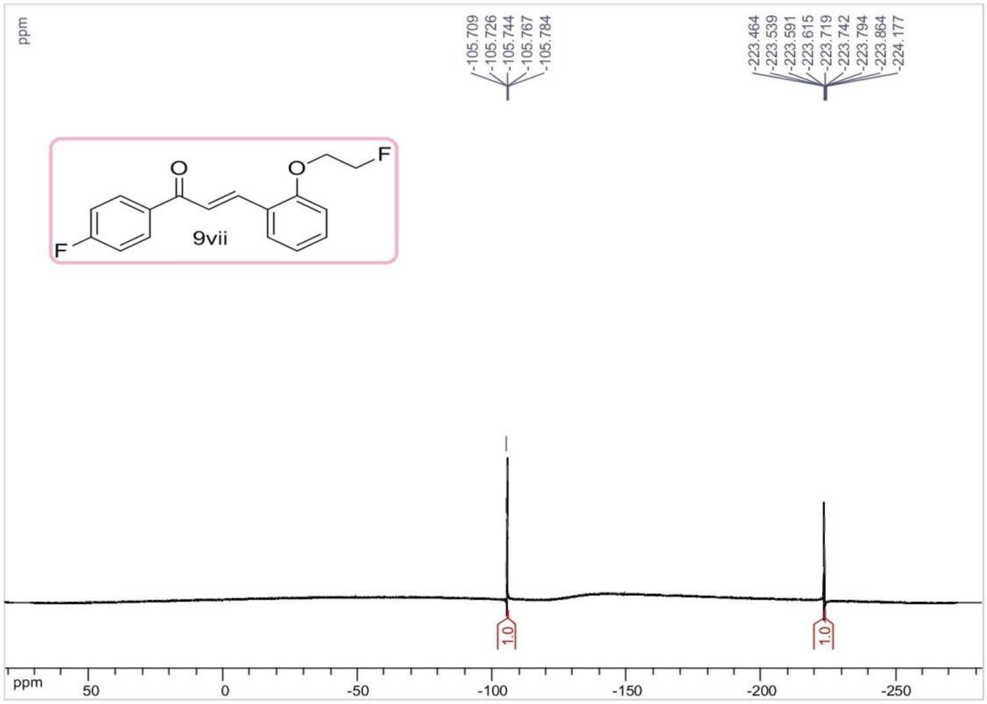

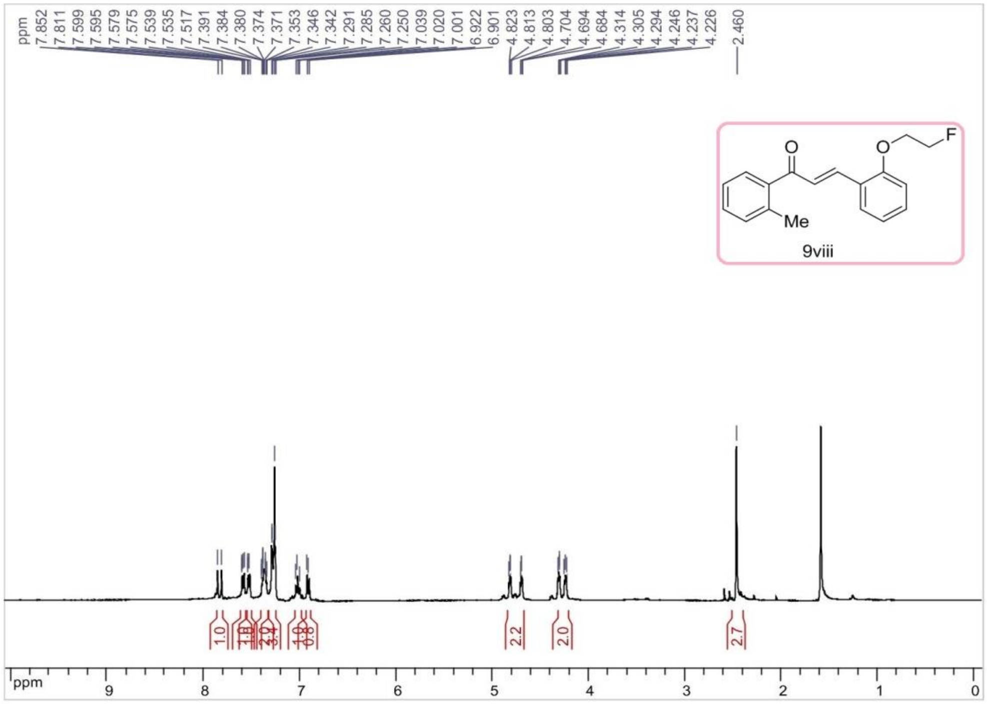

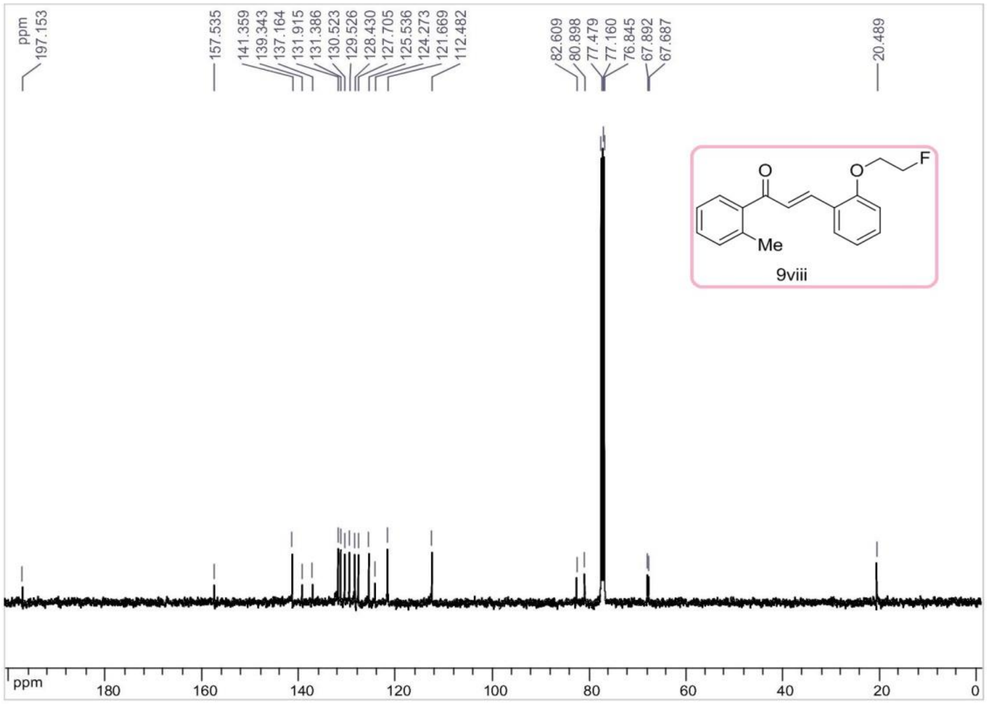
ppm
197.153
157.535
141.359
139.343
137.164
131.915
131.386
130.523
129.526
128.430
127.705
125.536
124.273
121.669
112.482
82.609
80.898
77.479
77.160
76.845
67.892
67.687
20.489
O
F
Me
9viii
ppm
180
160
140
120
100
80
60
40
20
0

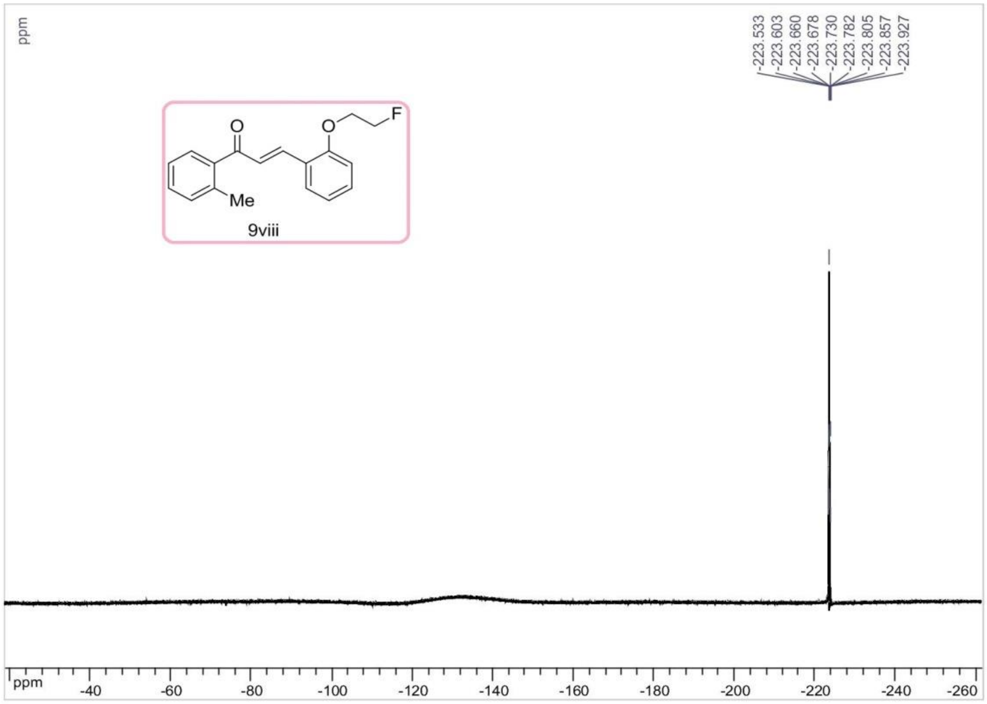
ppm
-223.533
-223.603
-223.660
-223.678
-223.730
-223.782
-223.805
-223.857
-223.927
O
F
Me
9viii
ppm
-40
-60
-80
-100
-120
-140
-160
-180
-200
-220
-240
-260
